# PPARγ promotes urothelial remodeling during urinary tract obstruction

**DOI:** 10.1038/s12276-025-01441-0

**Published:** 2025-05-01

**Authors:** Mohammad El-Harakeh, Felipe Rodriguez-Tirado, Andrew J. Hardman, Alexa Miehls, Oumoulkhairy Camara, Kelly M. Grounds, Glenis Tocaj, Macie Kercsmar, Birong Li, Xin Wang, Brian Becknell, Ashley R. Jackson

**Affiliations:** 1 Kidney and Urinary Tract Center, Abigail Wexner Research Institute, Nationwide Children’s Hospital, Columbus, OH USA; 2 Division of Nephrology and Hypertension, Nationwide Children’s Hospital, Columbus, OH USA; 3 Department of Pediatrics, The Ohio State University College of Medicine, Columbus, OH USA

**Keywords:** Obstructive nephropathy, Bioinformatics, Genetic models, Imaging

## Abstract

Urinary tract obstruction (UTO) is a common cause of kidney injury that can result in chronic kidney disease and end-stage renal disease. Heterogeneity in the extent of obstructive renal damage in humans with UTO implies the existence of unknown mechanisms that protect against or accelerate kidney injury. Prior studies show that congenital and acquired UTO initiate a conserved, protective program of renal urothelium remodeling that culminates in expansion of uroplakin (UPK)^+^ cells to promote renal structural integrity. However, the cellular and molecular mechanisms that regulate UPK expression in the renal urothelium are unknown. Peroxisome proliferator-activated receptor γ (PPARγ) drives urothelial differentiation and UPK expression in other tissues but has not been investigated in the renal urothelium. Here we demonstrate that activation of PPARγ in UPK^+^ cells is critical for UTO-induced renal urothelium remodeling. Conditional deletion of *Pparg* perturbs UPK expression and accelerates parenchymal thinning during UTO, while conditional activation of PPARγ increases UPK expression and results in parenchymal preservation. This study underscores the significance of renal urothelium during UTO and shows that UTO-induced renal urothelial remodeling is achieved through activation of PPARγ. These findings form the foundation for future studies that will determine the therapeutic utility of PPARγ agonists during congenital and acquired UTO.

## Renal urothelium remodeling protects against kidney injury

Urinary tract obstruction (UTO) is a major cause of kidney injury. Emerging evidence highlights the role of uroplakin (UPK)^+^ cells in protecting the kidney during UTO, but the regulatory mechanisms for the UPK expression were unclear. Using bioinformatics, researchers identified peroxisome proliferator-activated receptor γ (PPARγ) as a key upstream regulator, which they validated through conditional genetic manipulation of *Pparg* during UTO. Deleting PPARγ reduced the UPK expression and accelerated renal thinning, while activating it preserved renal parenchyma. These findings suggest that PPARγ pathways are crucial for protecting the renal urothelium during UTO, providing a potential therapeutic target with PPARγ agonists. The study also reveals significant differences between the upper tract and bladder urothelium, warranting further investigation.

This summary was initially drafted using artificial intelligence, then revised and fact-checked by the author.

## Introduction

Urinary tract obstruction (UTO) is a common cause of kidney injury that can lead to acute kidney injury and chronic kidney disease^1–3^. Acquired forms of UTO predominate in adults, while congenital etiologies predominate in children. Beyond interventions to relieve UTO, there are no available measures to prevent or reverse obstructive kidney injury. Research designed to improve UTO outcomes has been prioritized in the long-term strategic plans of major urological organizations^4,5^.

Prior studies show that congenital and acquired UTO initiate a conserved, protective program of renal urothelium remodeling that culminates in expansion of uroplakin (UPK)^+^ cells^6–8^. Renal UPK^+^ cells express UPK1A, UPK1B, UPK2 and UPK3A, which assemble immature urothelial plaques at the apical surface during homeostasis^9^. Following obstruction, UPK^+^ cells form mature, bladder-like urothelial plaques known to contribute to the formation of a compliant and impermeable urothelial barrier^10–12^. Depletion of UPK^+^ cells or ablation of the urothelial plaques using *Upk1b*-null mice accelerates parenchymal loss, renal dysfunction and mortality during UTO^7^. Therefore, UPK^+^ cells promote renal structural integrity during congenital and acquired UTO^7^. This suggests that the efforts to drive UPK expression may attenuate obstructive kidney disease, but the cellular and molecular mechanisms that regulate UPK expression in the renal urothelium are unclear.

Notably, renal urothelium is morphologically distinct from renal pelvis, ureter and bladder urothelium^13,14^, and renal urothelium cell types and distributions are unique^15^. Moreover, the developmental origin of UPK^+^ cells in renal urothelium is distinct from lineage relationships observed in bladder and ureteral urothelium^15–18^. In the kidney, keratin-5 (KRT5)^+^ cells exhibit an age-restricted potential to form UPK^+^ cells^15^. While, UTO-induced urothelial injury widens the window of multipotency, adult KRT5^+^ cells appear unable to escape lineage restriction and do not form the majority of UPK^+^ cells following UTO^15^. Thus, the adult renal urothelium repair progenitor remains unknown. In the bladder, adult intermediate UPK^+^ cells function as tissue repair progenitors^17,19,20^. The existence of an intermediate-like UPK^+^ cell in the kidney is uncertain, and a progenitor role for UPK^+^ cells in the kidney has not been investigated.

Peroxisome proliferator-activated receptor γ (PPARγ) is a nuclear receptor associated with transcription of genes linked to differentiation in numerous cell types, including urothelium^21–27^. Upon ligand binding, PPARγ heterodimerizes with a retinoid X receptor α (RXRα), associates with cofactors and binds to peroxisome proliferator response elements on target genes, such as fatty acid-binding protein 4 (FABP4)^22,28^. Pharmacologic activation of PPARγ directs urothelial differentiation in two-dimensional and three-dimensional cultures^23,26^. PPARγ indirectly promotes UPK (UPK1A, UPK2 and UPK3A) expression through regulation of intermediary transcription factors, forkhead box A1 (FOXA1), interferon regulatory factor-1 and Grainyhead-like 3 (GRHL3)^22,25,29^.

Recent in vivo studies demonstrate that conditional deletion of *Pparg* in urothelial cells abrogates the formation of terminally differentiated bladder urothelial cells, while conditional activation of PPARγ promotes differentiation of urothelial basal cells^21,22^. Although UTO engages a program of renal urothelium differentiation, a role for PPARγ in the renal urothelium or during UTO-induced renal urothelium remodeling has not been investigated.

In this study, we used lineage analysis to show that adult UPK^+^ cells serve as tissue repair progenitors during UTO and found that UTO induces PPARγ expression and activation in UPK^+^ cells. Conditional deletion of *Pparg* during UTO perturbs UPK expression and accelerates parenchymal thinning, while conditional activation of PPARγ enhances UPK expression and results in parenchymal preservation during UTO. These findings form the basis for the development of therapies aimed at bolstering the renal urothelium to mitigate obstructive kidney disease.

## Materials and methods

### Animals

The Public Health Service Animal Welfare Assurance number A3544-01 and Institutional Animal Care and Use Committee number AR16-00058 were used. Tg(Upk2-icre/ERT2)1Ccc/J (*Upk2*^CreERT2^, Jax #024768)^30^, B6.Cg-*Gt(ROSA)26Sor*^*tm14(CAG-tdTomato)Hze*^/J (*R26*^tdT^, Jax #007914)^31^, B6.129-*Pparg*^*tm2Rev*^/J (*Pparg*^fl/fl^, Jax #004584)^31^ and *ROSA26-CAG- STOP-VP16PPARG-IRES-EGFP* (*VP16-Pparg*^*fl/fl*^)^32^ mice were used (Supplementary Table 1). Tail DNA was isolated for genotyping by standard PCR (Supplementary Table 1). The order in which surgery, ultrasound and euthanasia were performed in control and mutant mouse groups was random to minimize potential confounders. The surgeon, ultrasonographer and downstream analytical team were blinded to mouse genotypes and group allocation to minimize bias.

### Histology and immunofluorescence

Formalin-fixed tissues were paraffin processed, sectioned at 4 µm and mounted on charged slides. Routine hematoxylin and eosin and Picrosirius Red (PSR) staining were performed. Immunolocalization was performed using anti-PPARγ (Cell Signaling Technology), anti-FABP4 (R&D Systems), anti-FOXA1 (also called HNF4, Santa Cruz Biotechnology), anti-GRHL3 (Abcam), anti-KRT14 (BioLegend), anti-KRT5 (BioLegend, Abcam), anti-KRT20 (Invitrogen), anti-RXRα (Cell signaling Technology), anti-tdTomato (TDT) (Rockland Immunochemicals; MyBioSource), anti-UPK1A (Santa Cruz Biotechnology), anti-UPK1B (Sigma-Aldrich) and anti-UPK3A (AbClonal, Fitzgerald), anti-KI67 (Abcam) and anti-P63 (Santa Cruz Biotechnology) primary antibodies (Supplementary Table 2). Cy3, Cy5 and Alexa Fluor 488 secondary antibodies (Jackson ImmunoResearch Laboratories) were used at 1:300. The coverslips were mounted using ProLong Antifade (Fisher Scientific). The images were captured using a Nikon Ti2-E microscope and ORCA-Fusion GenIII camera (fluorescent micrographs) or DS-Ri2 color camera (brightfield and polarized micrographs) (Nikon Instruments). The micrographs underwent equivalent brightness and contrast adjustments to enhance print view. The results were quantified using ImageJ v1.53k or QuPath v0.4.3 open source software^33^. For fluorescent image analysis, briefly, the renal urothelium was manually annotated using QuPath; cell detection or pixel thresholding tools were applied, and images were batch processed. PPARγ nuclear expression intensity was measured in arbitrary units, and thresholding levels were established using PPARγ-negative nuclei adjacent to the renal urothelium. For PSR image analysis, briefly, ImageJ was used to annotate regions of interest (ROIs) on PSR images collected using standard brightfield images (5–11, 20× images/kidney). The suburothelium compartment was annotated as a ROI spanning 100 μm beneath the renal urothelium. The renal cortex and medulla were captured as 20× images. The ROIs were transferred to polarized images, and the color threshold tool was used to annotate collagen fibers (spanning red, green and yellow hues). All measurements were exported to excel then added to GraphPad Prism v10.1.1 to apply appropriate statistical analyses as indicated in figure legends (GraphPad Software).

### Cre recombination

Tamoxifen (TMX, Sigma-Aldrich) was dosed via intraperitoneal administration at stages defined in each experiment to induce Cre-mediated recombination. For lineage experiments (using the R26^tdT^ line), a single dose of TMX (75 mg kg^−1^ body weight in corn oil) was administered. For conditional deletion or conditional activation of *Pparg* (using *Pparg*^fl/fl^ and *VP16-Pparg*^*fl/fl*^ lines), TMX (37.5 mg kg^−1^ body weight) was administered six times over 2 weeks leading up to surgical obstruction, followed by two doses following obstruction. Unexpected TMX effects were ruled out by using Cre-negative mice, and an inappropriate Cre;LoxP recombination was ruled out by using corn oil (carrier) treatment.

### UUO

A unilateral ureteral obstruction (UUO) was performed according to methods taught at the Mouse Kidney Injury Workshop (Vanderbilt University Medical Center) as previously described^7,15^. Briefly, following inhalation isoflurane induction, the right kidney was dorsally externalized, and the proximal ureter was ligated using a nonabsorbable silk suture. Ureteral ligation was omitted for sham surgery. The surgical site was closed using absorbable Polydioxanone (PDS) II suture and buprenorphine (0.05 mg kg^−1^ intraperitoneal) was administered for analgesia. A UUO was performed by the same experienced member to minimize variability. Every effort was made to reduce pain, suffering and distress. The humane endpoints established at the Institutional Animal Care and Use Committee (IACUC) review were strictly followed, and the mice were monitored daily. UUO was well tolerated, and none of the mice in this study met the endpoint criteria.

### Renal ultrasound

A renal ultrasound was performed, and hydronephrosis was graded as previously described^6,7,9,34^. Briefly, the isoflurane-anesthetized mice were shaved and depilated. Using a 40-mHz transducer, the longitudinal axis of each kidney was visualized. The largest longitudinal plane was imaged, and the percent parenchyma was calculated using the following equation: ((transverse renal width – renal pelvis diameter) + renal papilla width)/transverse renal width^34^.

### RNA extraction, cDNA synthesis and quantitative PCR

The RNA was extracted from decapsulated, snap-frozen whole kidneys using the mirVana kit (Life Technologies) as previously described^6,7,9^. The RNA was reverse transcribed to complementary DNA using the Thermo Scientific Verso cDNA Synthesis Kit (Fisher Scientific). SYBR green PCR master mix (Fischer Scientific) and gene-specific primers (Supplementary Table 3) were used to amplify 50 ng of cDNA on a 7500 real-time PCR system (Applied Biosystems). The results were expressed using the 2^−∆∆CT^ method normalized to the housekeeping gene *Gapdh* (Supplementary Table 3). The results were graphed and analyzed using GraphPad Prism.

### scRNA-seq data collection and analyses

The single-cell combinatorial indexing RNA sequencing (sci-RNA-seq3) datasets from mouse kidneys were obtained from the Gene Expression Omnibus (GEO) database under accession number GSE190887 (ref. ^35^). The datasets encompassed samples collected at six time points: 0, 2, 4, 6, 10 and 14 days post-UUO, representing both UUO-affected and healthy (called baseline) kidneys. A total of 162,611 high-quality cells were collected in the analysis, covering 19 distinct kidney cell types. To assess the effects of UUO on kidney cellular populations, we systematically compared the expression of key urothelial markers across all cell types between UUO and control conditions.

For tracing the UPK-lineage, we extracted cells belonging to the urothelial cell population expressing at least two read counts of any of the following UPK markers: *Upk1a*, *Upk1b*, *Upk2*, and *Upk3a*. This selection resulted in 1274 UPK^+^ cells, including 318 from baseline kidneys and 956 from UUO-affected kidneys. The extracted UPK^+^ cells were processed following the standard Seurat workflow. The cells were normalized using SCTransform, a method that normalizes and stabilizes variance across cells while accounting for sequencing depth. We identified the top 2000 most variable genes across the dataset, which were used for downstream analysis. To mitigate technical noise, including mitochondrial gene expression variability, we regressed out the percentage of mitochondrial gene content during the scaling process. A principal component analysis was performed on the scaled data to reduce dimensionality and identify key sources of variation within the dataset. The optimal number of principal components was determined using an elbow plot. To account for batch effects arising from different time points and sample conditions, we applied Harmony, a batch correction algorithm, to ensure robust alignment of cells across conditions without compromising biological variance. Dimensionality reduction was performed using a uniform manifold approximation and projection (UMAP). The clustering was conducted using the Louvain algorithm through Seurat’s FindNeighbors and FindClusters functions, with the clustering resolution empirically determined to balance granularity with biological interpretability. A similar strategy was also applied to analyze the KRT5 lineage. A total of 680 KRT5 urothelium cells were collected, including 30 cells from baseline kidneys and 650 cells from UUO-affected kidneys. The UMAP plots were generated to visualize the cellular landscape and identify distinct subpopulations within *Upk*^*+*^ and *Krt5*^*+*^ cells. To gain deeper insights into urothelium subpopulation dynamics in the *Upk*^+^ and *Krt5*^*+*^ lineage during UUO, we performed a detailed analysis in lineage proportions and gene expression changes across key urothelium markers. Superficial cell markers (*Clu*, *Uchl1* and *Krt20*), basal cell markers (*Trp63*, *Krt17*, *Krt14* and *Krt5*) and cycling cell markers (*Hmgb2*, *Birc5*, *Top2a* and *Miki67*) were systematically profiled to evaluate their expression patterns and cell proportional shifts.

To identify differentially expressed genes (DEGs) in UPK^+^ cells between baseline (postoperative day (POD)0) and UUO conditions, we utilized the ‘MAST’ implemented in Seurat’s FindMarkers function. The genes were considered differentially expressed if they met the following criteria: an adjusted *P* value ≤0.05 (Bonferroni correction) and a fold change ≥1.5. The DEGs were functionally annotated through Gene Ontology enrichment analysis using ClusterProfiler. In addition, we used ChIP-X Enrichment Analysis (ChEA3) to predict transcription factors regulating the observed DEGs in the UPK^+^ cells during UUO.

### Statistical analyses

The statistical analyses were performed using Prism Software. The normality was tested using the Shapiro–Wilk test. When appropriate, we applied a one-way analysis of variance (ANOVA), an unpaired two-tailed *t*-test or unpaired Mann–Whitney test. The differences between groups with a *P* < 0.05 were considered statistically significant. The data are presented as means ± standard deviation (s.d.). The figure legends indicate the sample size, sex, statistical tests and relevant multiple comparison correction used in the study.

## Results

### Adult UPK^+^ cells contribute to renal urothelium remodeling during UUO

UUO triggers a sequence of renal urothelium remodeling that includes (1) loss of UPK expression at POD1, (2) proliferation of KRT5^+^ cells at POD2 and (3) stratification and increased UPK expression at ≥POD7 (refs. ^7,8^). Prior work revealed that the KRT5 lineage does not account for a majority of renal UPK^+^ cells formed following UUO^15^, suggesting the existence of an alternative adult renal urothelium progenitor during UUO. Therefore, we performed lineage analysis during UUO using *Upk2*^CreERT2^;*R26*^tdT^ mice to determine whether renal UPK^+^ cells function as tissue repair progenitors. We bolstered our understanding of the renal urothelium using a bioinformatics examination of publicly available UUO single-cell RNA sequencing (scRNA-seq) data^35^, where *Upk*^*+*^ cells were defined by *Upk1a*, *Upk1b*, *Upk2* and *Upk3a* expression, and *Krt5*^+^ cells were identified by *Krt5* expression.

**Fig. 1:**
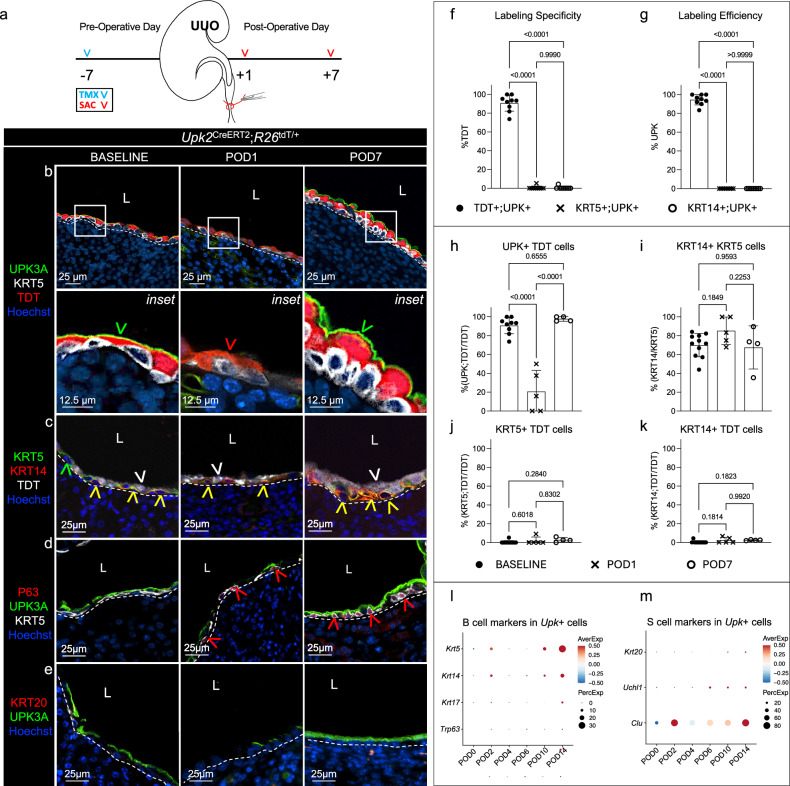
Adult UPK^+^ cells contribute to renal urothelium remodeling during UUO. **a**, A schematic that depicts the experimental plan. SAC, sacrifice. **b**, The representative micrographs and insets show anti-UPK3A, anti-KRT5, anti-TDT and Hoechst in the renal urothelium of *Upk2*^CreERT2^;*R26*^tdT/+^ mice at baseline, POD1 and POD7. The white dashed line is the renal urothelium basement membrane, the green arrowhead is the TDT^+^UPK^+^ cell and the red arrowhead is the TDT^+^ cell with an undetectable UPK3A expression. L, lumen. **c**, The representative micrographs show anti-KRT5, anti-KRT14, anti-TDT and Hoechst in the renal urothelium of *Upk2*^CreERT2^;*R26*^tdT/+^ mice at baseline, POD1 and POD7. The white dashed line is the renal urothelium basement membrane, the white arrowhead is the TDT^+^ cell, the green arrowhead is the KRT5^+^ cell and the yellow arrowhead is the KRT5^+^KRT14^+^ cell. **d**, The representative micrographs show anti-P63, anti-UPK3A, anti-KRT5 and Hoechst in the renal urothelium of *Upk2*^CreERT2^;*R26*^tdT/+^ mice at baseline, POD1 and POD7. The white dashed line is the renal urothelium basement membrane, and the red arrowhead is the P63^+^KRT5^+^ cell. **e**, The representative micrographs show anti-KRT20, anti-UPK3A and Hoechst in the renal urothelium of Upk2^CreERT2^;R26^tdT/+^ mice at baseline, POD1 and POD7. The white dashed line is the renal urothelium basement membrane. **f**, **g**, The graphs show the specificity (%TDT) (**f**) and efficiency (%UPK) (**g**) of reporter labeling in the renal urothelium at baseline. *Upk2*^CreERT2^;*R26*^tdT/+^ (*n* = 9 mice). The bars are the mean, and the error bars represent the s.d. The *P* values represent a one-way ANOVA with a Tukey’s multiple comparison correction. **h**–**k**, The graphs show the percent of TDT^+^ cells that express UPK (**h**), KRT5^+^ cells that express KRT14 (**i**), TDT^+^ cells that express KRT5 (**j**) and TDT^+^ cells that express KRT14 (**k**) in the renal urothelium of *Upk2*^CreERT2^;*R26*^tdT/+^ mice at baseline, POD1 and POD7. *Upk2*^CreERT2^;*R26*^tdT/+^ (*n* ≥ 4 mice). The bars are the mean, and the error bars represent the s.d. The *P* values represent a one-way ANOVA with a Tukey’s multiple comparison correction. **l**,**m**, The dot plots show the average marker expression and the percent of *Upk*^+^ (defined as expressing *Upk1a*, *Upk1b*, *Upk2* and *Upk3a*) cells that express each superficial (S) cell (**l**) and basal (B) cell (**m**) marker.

TMX was used to inducibly and permanently label the UPK-lineage with a TDT reporter 1 week before UUO (Fig. 1a). We confirmed a high specificity (90.73 ± 8.61%) (Fig. 1b, f) and efficiency (94.51 ± 5.57%) (Fig. 1b, g) for reporter labeling, indicating TDT marked UPK^+^ cells and not KRT5^+^ (0.48 ± 1.59%) nor KRT14^+^ (0.40 ± 1.31%) cells in the renal urothelium at baseline (Fig. 1b, f, g and Supplementary Fig. 1). We then mapped the fate of the UPK-lineage during UUO using TDT expression. Compared with baseline, UPK3A expression was faint and detected in only a fraction of the TDT^+^ cells at POD1 (Fig. 1b and Supplementary Fig. 1a), suggesting that despite downregulation of UPK expression, the UPK-lineage persisted. This finding was supported by scRNA-seq analysis, which identified a decrease in the proportion of *Upk*^*+*^ cells and a decrease in the expression of *Upk1a*, *Upk1b*, *Upk2* and *Upk3a* transcripts at POD2 compared with the baseline (Supplementary Fig. 1c, d). Importantly, while KRT5^+^ cells and KRT5^+^;KRT14^+^ cells are prominent at baseline, and the proportions of *Krt5*^+^ and *Krt14*^+^ cells and transcripts are increased following UUO, lineage analysis only rarely identified TDT^+^;KRT5^+^ (1.82 ± 4.07%) or TDT^+^;KRT14^+^ (2.42 ± 1.41%) cells at POD2 (Fig. 1c, i–k and Supplementary Fig. 1b–d), indicating the TDT^+^UPK-lineage did not contribute significantly to the increased proportion of KRT5^+^ or KRT14^+^ cells following UUO. Instead following the transient downregulation of UPK protein (lineage analysis) and mRNA (scRNA-seq), all *Upk* transcripts increased beginning at POD4 (Supplementary Fig. 1c, d), and our lineage analysis revealed that TDT^+^ cells prominently expressed UPK3A (97.81 ± 2.54%) but not KRT5 (2.82 ± 2.51%) or KRT14 (2.40 ± 0.45%) at POD7 (Fig. 1h, j, k).

**Fig. 2:**
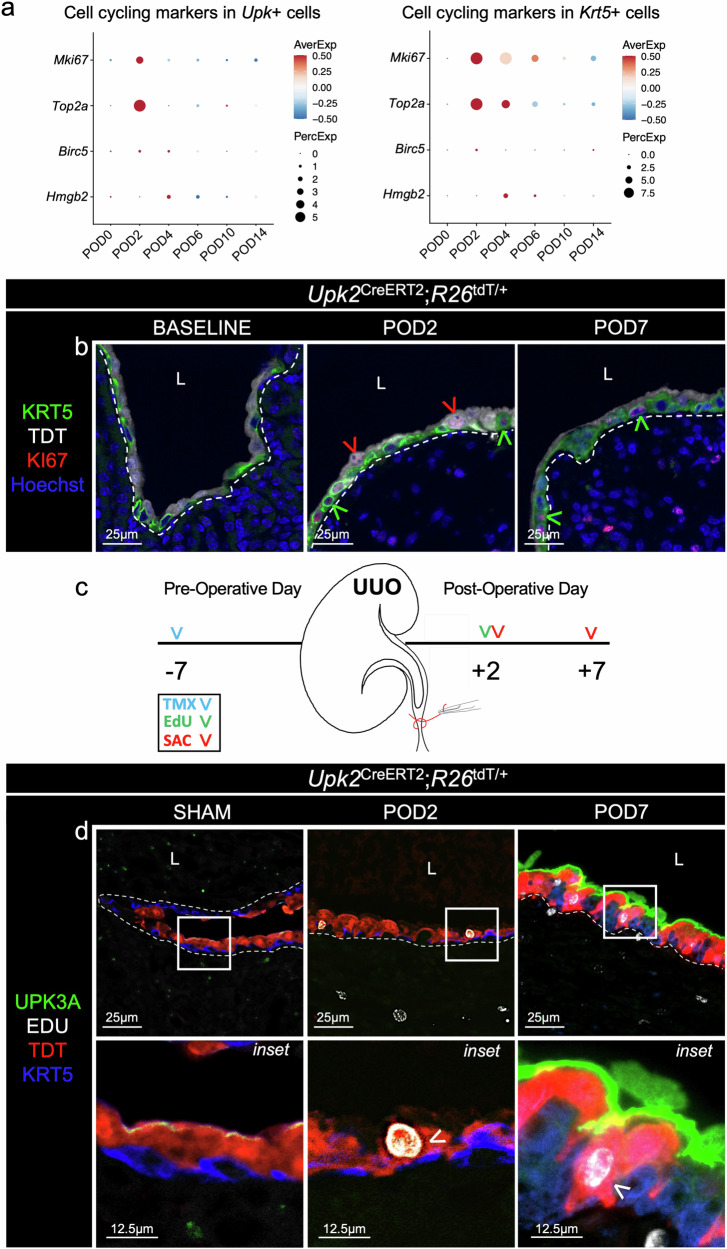
The adult UPK-lineage proliferates during UUO. **a**, The dot plots show the average maker expression and the percent of *Upk*^+^ or *Krt5*^+^ cells that express each cell cycling marker. **b**, The representative micrographs show anti-KRT5, anti-TDT, anti-KI67 and Hoechst in the renal urothelium of *Upk2*^CreERT2^;*R26*^tdT/+^ mice at baseline, POD2 and POD7. The white dashed line is the renal urothelium basement membrane, the red arrowhead is the TDT^+^;KI67^+^ cell and the green arrowhead is the KRT5^+^;KI67^+^ cell. L, lumen. **c**, A schematic depicting the experimental plan. SAC, sacrifice. **d**, The representative micrographs and insets show anti-UPK3A, anti-TDT, anti-KRT5 and EdU labeling in the renal urothelium of *Upk2*^CreERT2^;*R26*^tdT/+^ mice at baseline (sham injury), POD2 and POD7. Notably, EdU was dosed at POD2, and mice were killed 2 h later (POD2) or at POD7. The white dashed line is the renal urothelium basement membrane, and the white arrowhead is the EdU^+^;TDT^+^ cell (POD2) and EdU^+^;TDT^+^;UPK^+^ cell (POD7).

Next, we used bioinformatics to determine whether renal *Upk*^*+*^ cells exhibited features consistent with conventional basal or superficial cells and whether cycling cell markers were detected during obstruction-induced renal urothelium remodeling. We determined that the expression of basal cell markers (*Krt5*, *Krt14*, *Krt17* and *Trp63*) was rare in *Upk*^+^ cells and confirmed that basal cell transcription factor, P63, was expressed by KRT5^+^ but not UPK^+^ cells following UUO (Fig. 1d, l and Supplementary Fig. 1e), indicating renal UPK^+^ cells are not entirely consistent with conventional basal cells. We then identified increased expression of superficial cell markers (*Clu*, *Uchl1* and *Krt20*) following UUO, with a dramatic increase in the percent of *Clu*^+^ cells and expression of *Clu* beginning POD2 (Fig. 1m and Supplementary Fig. 1e). Notably, the proportion of *Upk*^+^ cells that expressed *Uchl1* or *Krt20* was rare (Supplementary Fig. 1e), and KRT20 could not be detected in the renal urothelium (Fig. 1e and Supplementary Fig. 2d), indicating renal UPK^+^ cells are not entirely consistent with conventional superficial cells. Interestingly, *Mki67* and *Top2a*, markers of cellular proliferation, were increased in *Upk*^*+*^ cells at POD2 (Fig. 2a), and we identified KI67^+^;TDT^+^ cells at POD2 (Fig. 2b). Therefore, we administered 5-ethynyl-2′-deoxyuridine (EdU) at POD2 to determine whether the TDT^+^ cells from the UPK-lineage proliferated following UUO (Fig. 2c). While renal urothelium is normally quiescent, we confirmed that TDT^+^;UPK cells incorporated EDU at POD2 (2 h after EDU administration), and that the EDU label was retained in a subset of TDT+ cells that expressed UPK3A at POD7 (Fig. 2d), indicating cells within the UPK-lineage have the capacity to both proliferate and differentiate following UUO. Interestingly, KI67 mRNA and protein levels indicate the window of proliferation among the UPK-lineage was limited to POD2, while KRT5^+^ cells were proliferative at both POD2 and POD7. Altogether these data demonstrate that UUO causes the UPK-lineage to downregulate UPK expression (POD1), proliferate (POD2) and reacquire UPK (POD7). Moreover, our findings indicate that renal UPK^+^ cells function as adult tissue repair progenitors during UUO, which aligns with roles for conventional intermediate cells in urothelial tissue repair^17,19,20^.

### scRNA-seq predicts PPARγ as a key transcription factor that regulates the UPK^+^ cell response to UUO

**Fig. 3:**
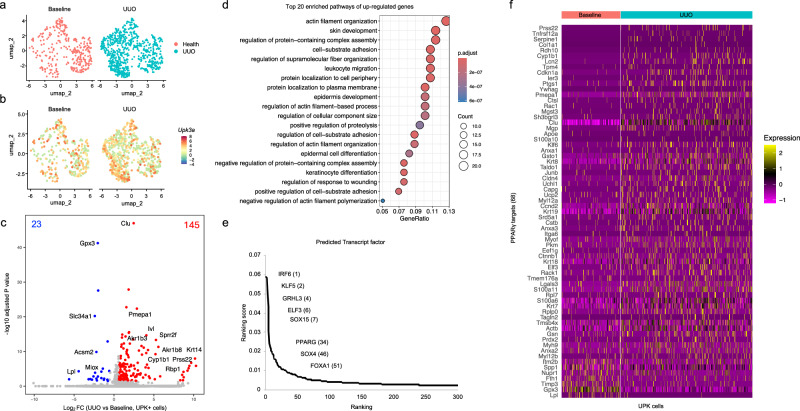
scRNA-seq demonstrates increased UPK cells and predicts PPARγ activation during UUO. **a**, UMAP plots of scRNA-seq data showing 1274 UPK cells in baseline (318 cells) and UUO (956 cells) kidneys clustered by their expression of *Upk1a*, *Upk1b*, *Upk2* and *Upk3a*. **b**, UMAP plots of UPK cells depicting *Upk3a* expression in single cells. **c**, A volcano plot showing the magnitude and significance of DEGs between baseline and UUO (POD6, POD10 and POD14) in *Upk*^*+*^ cells. Statistically significant downregulated genes in UUO (23) are shown in blue, and the statistically significant upregulated genes in UUO (145) are shown in red. **d**, The top 20 enriched biological processes of DEGs in UUO. **e**, The graph shows ChEA3-predicted transcription factors (TFs) ranked based on their predicted activity within DEGs in UUO compared with the baseline. **f**, A heat map that shows the expression of PPARγ target genes in baseline and UUO.

To better understand what regulates the increased expression of UPK following UUO, we compared *Upk*^+^ cells at baseline (POD0) to advanced UUO stages (≥POD6) (Fig. 3a, b). We identified 168 DEGs in *Upk*^*+*^ cells of UUO compared with the baseline kidneys, with 145 upregulated and 23 downregulated genes (Fig. 3c and Supplementary Table 4). The genes upregulated in *Upk*^*+*^ cells included *Lcn2*, *Clu*, *Krt5*, *Krt14* and *Sox4*, highlighting increases in markers of kidney injury and progenitor activation. Gene Ontology enrichment analysis revealed significant involvement in keratinocyte differentiation, epidermal cell differentiation, regulation of response to wounding and actin filament organization, supporting observations that renal urothelium undergoes significant remodeling in response to UUO (Fig. 3d and Supplementary Table 5).

Next, we used DEGs to predict transcription factors that regulate the UPK^+^ cell response to UUO (Supplementary Table 6). Among the top ranked transcription factors, we identified Kruppel-like transcription factor 5 (KLF5), GRHL3, ELF3, PPARγ and FOXA1 (Fig. 3e). Interestingly, KLF5 is an upstream regulator of *Ppar*γ, while *Grhl3, Elf3 and Foxa1* are downstream regulators of PPARγ signaling. Increased expression of PPARγ targets genes reveals increased activation of PPARγ during UUO, suggesting a key role for PPARγ in UPK^+^ cells. (Fig. 3f). Together these single-cell data validate that UUO triggers renal urothelium remodeling that culminates in increased *Upk* expression and links the transcription factor PPARγ to UPK^+^ cells.

### PPARγ is induced and activated in UPK^+^ cells during UUO

**Fig. 4:**
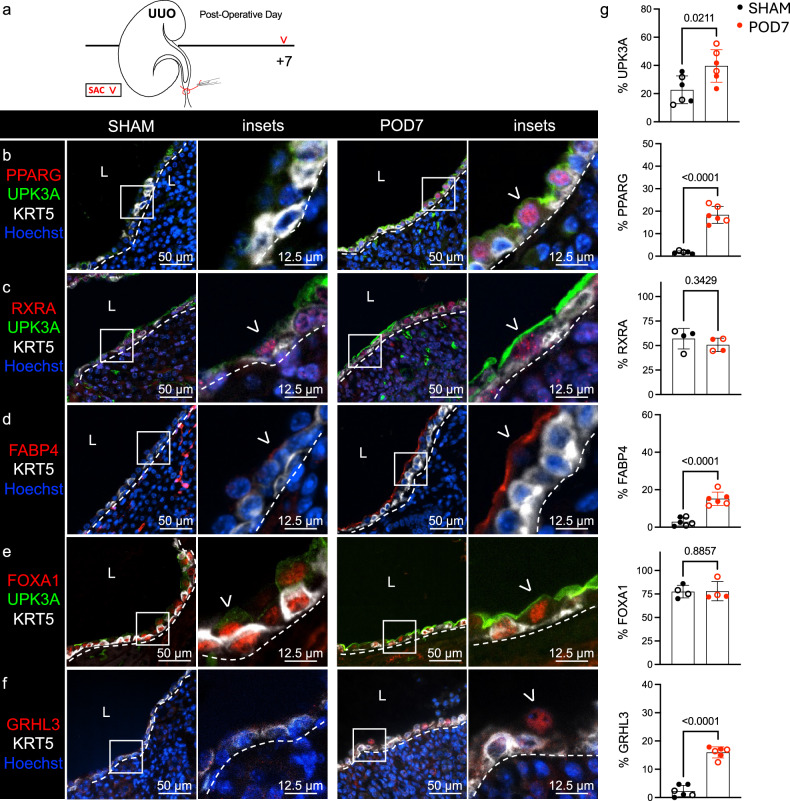
PPARγ is induced and activated in UPK^+^ cells during UUO. **a**, A schematic that depicts the experimental plan. SAC, sacrifice. **b**, The representative micrographs and insets show anti-PPARG, anti-UPK3A, anti-KRT5 and Hoechst labeling in the renal urothelium of sham and POD7 kidneys. The white dashed line is the renal urothelium basement membrane, and the white arrowhead is the PPARG^+^UPK^+^ cell. L, lumen. **c**, The representative micrographs and insets show anti-RXRA, anti-UPK3A, anti-KRT5 and Hoechst labeling in the renal urothelium of sham and POD7 kidneys. The white dashed line is the renal urothelium basement membrane, and the white arrowhead is the RXRA^+^ apical cell. **d**, The representative micrographs show anti-FABP4, anti-KRT5 and Hoechst labeling in the renal urothelium of sham and POD7 kidneys. The white dashed line is the renal urothelium basement membrane and the white arrowhead is the FABP4^+^ apical cell. **e**, The representative micrographs and insets show anti-FOXA1, anti-UPK3A and anti-KRT5 labeling in the renal urothelium of sham and POD7 kidneys. The white dashed line is the renal urothelium basement membrane, and the white arrowhead is the FOXA1^+^ apical cell. **f**, The representative micrographs and insets show anti-GRHL3, anti-KRT5 and Hoechst labeling in the renal urothelium of sham and POD7 kidneys. The white dashed line is the renal urothelium basement membrane, and the white arrowhead is the GRHL3^+^ apical cell. **g**, The graphs show (from top to bottom) the area of UPK3A expression as a percent of the total renal urothelium area, the PPARG index as a percent of the total renal urothelium nuclei count, the RXRA index as a percent of the total renal urothelium nuclei count, the area of FABP4 expression as a percent of the total renal urothelium area, the FOXA1 index as a percent of the total renal urothelium nuclei count and the GRHL3 index as a percent of the total renal urothelium nuclei count. Sham (*n* = 4–6 mice) and POD7 (*n* = 4–6 mice). The solid circle represents male, and the hollow circle represents female. The bars are the mean, and the error bars represent the s.d. The *P* values represent an unpaired two-tailed *t*-test, except for RXRA and FOXA1, where a Mann–Whitney test was used due to a nonparametric data distribution in the POD7 cohort.

PPARγ promotes urothelial differentiation and UPK expression^21,22,36^, but a role for PPARγ has not been investigated in the renal urothelium. To investigate whether PPARγ promotes UPK expression during UUO, we first profiled UPK expression (using UPK1A, UPK1B and UPK3A) and components of the *Pparγ* pathway in baseline (sham-operated kidneys) and obstructed kidneys at POD7 (Fig. 4a and Supplementary Figs. 2 and 3).

At baseline, the renal urothelium contains interspersed UPK^+^ cells, which express UPK1A, UPK1B, UPK2 and UPK3A^9^. scRNA-seq confirmed expression of *Upk1a*, *Upk1b*, *Upk2* and *Upk3a* in the renal urothelium at baseline (Supplementary Fig. 1), and we confirmed the expression of UPK1A, UPK1B and UPK3A (Supplementary Figs. 2 and 3); however, antibody availability limited our ability to detect UPK2 (data not shown). As expected, UPK3A protein and *Upk3a* mRNA expression was significantly increased during UUO (POD7) compared with the baseline (sham) (Fig. 4b, c, e, g and Supplementary Fig. 3)^7^. Interestingly, PPARγ expression was low or undetectable in the renal urothelium at baseline, which is in stark contrast to high levels of PPARγ observed in bladder urothelium at baseline^21,22^. Instead, nuclear expression of PPARγ was significantly increased during UUO and localized to UPK3A^+^ cells (Fig. 4b, g and Supplementary Fig. 3). Next, we assessed the PPARγ binding partner, RXRα, which was expressed in the renal urothelium at equivalent levels at baseline and during UUO (Fig. 4c, g). To determine whether PPARγ was activated during UUO, we assessed FABP4, a direct transcriptional target of PPARγ, and found significantly increased apical expression of FABP4 in UPK3A^+^ cells (Fig. 4d, g and Supplementary Fig. 3). FOXA1, a direct transcriptional target of PPARγ important for bladder urothelium differentiation, was expressed by KRT5^+^ and UPK3A^+^ cells at equivalent levels at baseline and during UUO (Fig. 4e, g). Notably, GRHL3, a downstream target of PPARγ and transcriptional driver of urothelial differentiation, was significantly increased in the renal urothelium during UUO compared with the baseline (Fig. 4f, g). Altogether, these data indicate that PPARγ is specifically expressed and activated in UPK^+^ cells during UUO and may regulate GRHL3 expression.

### Conditional deletion of *Pparg* limits UPK expression during UUO

**Fig. 5:**
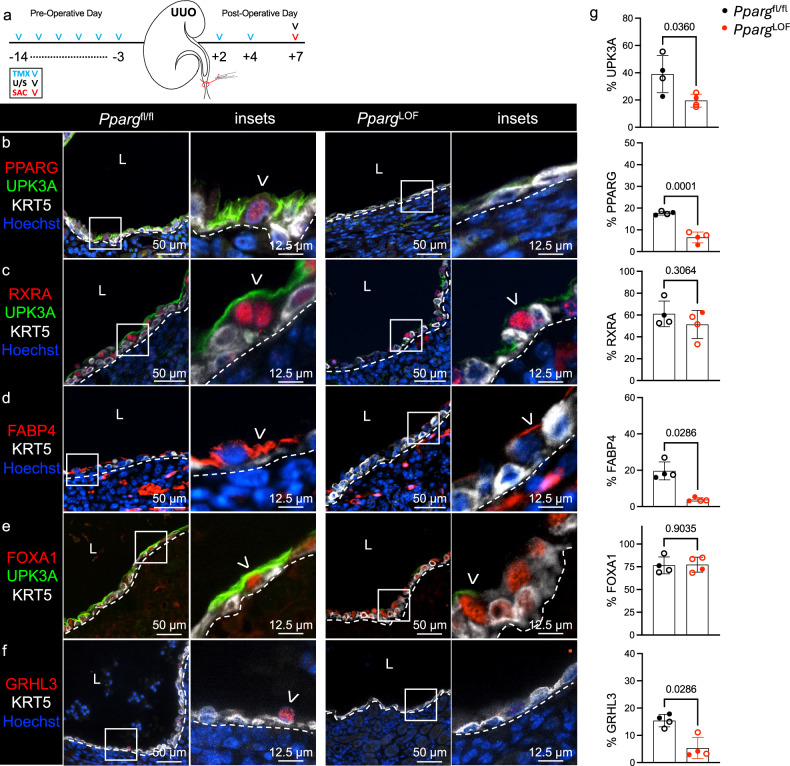
Conditional deletion of *Pparg* limits UPK expression during UUO. **a**, A schematic that depicts experimental plan. U/S, renal ultrasound; SAC, sacrifice. **b**, The representative micrographs and insets show anti-PPARG, anti-UPK3A, anti-KRT5 and Hoechst labeling in the renal urothelium of *Pparg*^fl/fl^ and *Upk2*^CreERT2^;*Pparg*^fl/fl^ (*Pparg*^LOF^) kidneys at POD7. The white dashed line is the renal urothelium basement membrane, and the white arrowhead is the PPARG^+^UPK^+^ cell. L, lumen. **c**, The representative micrographs and insets show anti-RXRA, anti-UPK3A, anti-KRT5 and Hoechst labeling in the renal urothelium of *Pparg*^fl/fl^ and *Pparg*^LOF^ kidneys at POD7. The white dashed line is the renal urothelium basement membrane, and the white arrowhead is the RXRA^+^ apical cell. **d**, The representative micrographs and insets show anti-FABP4, anti-KRT5 and Hoechst labeling in the renal urothelium of *Pparg*^fl/fl^ and *Pparg*^LOF^ kidneys at POD7. The white dashed line is the renal urothelium basement membrane, and the white arrowhead is the FABP4^+^ apical cell. **e**, The representative micrographs and insets show anti-FOXA1, anti-UPK3A and anti-KRT5 labeling in the renal urothelium of *Pparg*^fl/fl^ and *Pparg*^LOF^ kidneys at POD7. The white dashed line is the renal urothelium basement membrane and the white arrowhead is the FOXA1^+^ apical cell. **f**, The representative micrographs show anti-GRHL3, anti-KRT5 and Hoechst labeling in the renal urothelium of *Pparg*^fl/fl^ and *Pparg*^LOF^ kidneys at POD7. The white dashed line is the renal urothelium basement membrane, and the white arrowhead is the GRHL3^+^ apical cell. **g**, The graphs show (from top to bottom) the area of UPK3A expression as a percent of the total renal urothelium area, the PPARG index as a percent of the total renal urothelium nuclei count, the RXRA index as a percent of the total renal urothelium nuclei count, the area of FABP4 expression as a percent of the total renal urothelium area, the FOXA1 index as a percent of the total renal urothelium nuclei count and the GRHL3 index as a percent of the total renal urothelium nuclei count. *Pparg*^fl/fl^ (*n* = 4 mice), *Pparg*^LOF^ (*n* = 4 mice). The solid circle represents male, and the hollow circle represents female. The bars are the mean, and the error bars represent the s.d. The *P* values represent an unpaired two-tailed *t*-test, except for FABP4 and GRHL3, where a Mann–Whitney test was used due to a nonparametric data distribution in the *Pparg*^LOF^ cohort.

To functionally evaluate the role of PPARγ in the renal urothelium during UUO, we generated *Upk2*^CreERT2^;*Pparg*^fl/fl^ (hereafter called *Pparg*^LOF^) and *Pparg*^fl/fl^ control mice. The mice were treated with TMX, received UUO and were killed at POD7 (Fig. 5a). We observed a significant reduction in PPARγ in urothelium of *Pparg*^LOF^ mice during UUO (Fig. 5b, g and Supplementary Fig. 4a), while RXRα (PPARγ binding partner) remained unchanged (Fig. 5c, g). Next, we confirmed abrogation of *Pparg* signaling as evidenced by a significant decrease in FABP4 (direct transcriptional target of PPARγ) in the renal urothelium of *Pparg*^LOF^ mice compared with *Pparg*^fl/fl^ (Fig. 5d, g and Supplementary Fig. 3d). FOXA1 (direct transcriptional target of PPARγ) expression was not affected by *Pparg*^LOF^ (Fig. 5e, g). However, GRHL3 (PPARγ downstream target) expression was significantly decreased in the renal urothelium of *Pparg*^LOF^ mice (Fig. 5f, g). This coincided with decreased UPK1A and UPK1B (Supplementary Fig. 2b) and significant decreases in UPK3A protein and *Upk3a* mRNA expression (Fig. 5b, c, e, g and Supplementary Figs. 3a, d and 4a). Together these data indicate deletion of *Pparg* abrogates GRHL3 and UPK expression in the renal urothelium during UUO.

### Conditional activation of PPARγ increases UPK expression during UUO

**Fig. 6:**
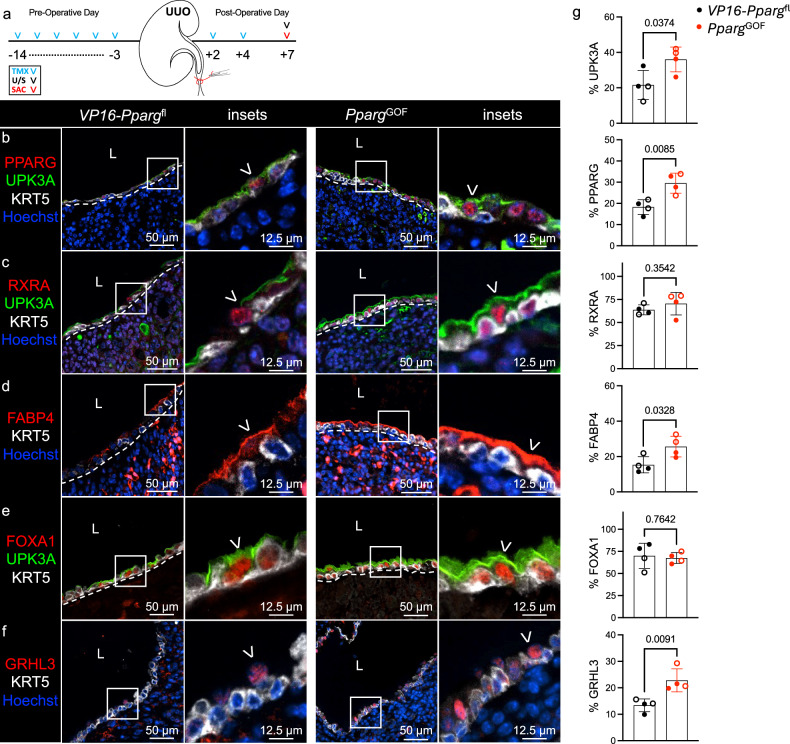
Conditional activation of PPARG increases UPK expression during UUO. **a**, A schematic that depicts the experimental plan. U/S, renal ultrasound; SAC, sacrifice. **b**, The representative micrographs and insets show anti-PPARG, anti-UPK3A, anti-KRT5 and Hoechst labeling in the renal urothelium of *VP16-Pparg*^fl^ (control) and *Upk2*^CreERT2^;*VP16-Pparg*^fl^ (*Pparg*^GOF^) kidneys at POD7. The white dashed line is the renal urothelium basement membrane, and the white arrowhead is the PPARG^+^UPK^+^ cell. L, lumen. **c**, The representative micrographs and insets show anti-RXRA, anti-UPK3A, anti-KRT5 and Hoechst labeling in the renal urothelium of *VP16-Pparg*^fl^ and *Pparg*^GOF^ kidneys at POD7. The white dashed line is the renal urothelium basement membrane, and the white arrowhead is the RXRA^+^ apical cell. **d**, The representative micrographs and insets show anti-FABP4, anti-KRT5 and Hoechst labeling in the renal urothelium of *VP16-Pparg*^fl^ and *Pparg*^GOF^ kidneys at POD7. The white dashed line is the renal urothelium basement membrane, and the white arrowhead is the FABP4^+^ apical cell. **e**, The representative micrographs and insets show anti-FOXA1, anti-UPK3A and anti-KRT5 labeling in the renal urothelium of *VP16-Pparg*^fl^ and *Pparg*^GOF^ kidneys at POD7. The white dashed line is the renal urothelium basement membrane, and the white arrowhead is the FOXA1^+^ apical cell. **f**, The representative micrographs and insets show anti-GRHL3, anti-KRT5 and Hoechst labeling in the renal urothelium of *VP16-Pparg*^fl^ and *Pparg*^GOF^ kidneys at POD7. The white dashed line is the renal urothelium basement membrane, and the white arrowhead is the GRHL3^+^ apical cell. **g**, The graphs show (from top to bottom) the area of UPK3A expression as a percent of the total renal urothelium area, the PPARG index as a percent of the total renal urothelium nuclei count, the RXRA index as a percent of the total renal urothelium nuclei count, the area of FABP4 expression as a percent of the total renal urothelium area, the FOXA1 index as a percent of the total renal urothelium nuclei count and the GRHL3 index as a percent of the total renal urothelium nuclei count. *VP16-Pparg*^fl^ (*n* = 4 mice), *Pparg*^GOF^ (*n* = 4 mice). The solid circle represents male, and the hollow circle represents female. The bars are the mean, and the error bars represent the s.d. The *P* values represent an unpaired two-tailed *t*-test.

To determine whether conditional activation of PPARγ could enhance UPK expression, we generated *Upk2*^CreERT2^;*VP16*-*Pparg*^fl^ (hereafter called *Pparg*^*GOF*^) mice. In *Pparg*^GOF^ mice, incorporation of a HSV *VP16* activator fused to the Pparg1 N-terminal renders PPARγ conditionally activated in a ligand-independent manner^21^. *Pparg*^*GOF*^ and *VP16*-*Pparg*^fl^ control mice were treated with TMX, received UUO and were killed at POD7 (Fig. 6a). We observed a significant increase in PPARγ in UPK3A^+^ cells of *Pparg*^*GOF*^ compared with *VP16*-*Pparg*^fl^ mice (Fig. 6b, g and Supplementary Fig. 4b). As was the case in *Pparg*^LOF^ experiments, RXRα (PPARγ binding partner) expression was unaffected by PPARγ activation (Fig. 6c, g). Next, we confirmed increased activation of PPARγ in the renal urothelium of *Pparg*^GOF^ mice as evidenced by a significant increase in apical FABP4 (direct transcriptional target of PPARγ) in UPK3A^+^ cells (Fig. 6d, g and Supplementary Fig. 3e). FOXA1 (direct transcriptional target of PPARγ) expression remained unchanged with PPARγ activation in the renal urothelium (Fig. 6e, g). Instead, GRHL3 (PPARγ transcriptional target) was significantly increased in the renal urothelium of *Pparg*^GOF^ mice compared with *VP16-Pparg*^fl^ during UUO (Fig. 6f, g). This coincided with increased UPK1A and UPK1B (Supplementary Fig. 2c) and significant increase in UPK3A protein and *Upk3a* mRNA expression (Fig. 6b, c, e, g and Supplementary Figs. 3a, e and 4b). These data indicate that conditional and constitutive activation of PPARγ increases GRHL3 and UPK expression in the renal urothelium during UUO.

### PPARγ activation in renal urothelium promotes renal structural integrity during UUO

**Fig. 7:**
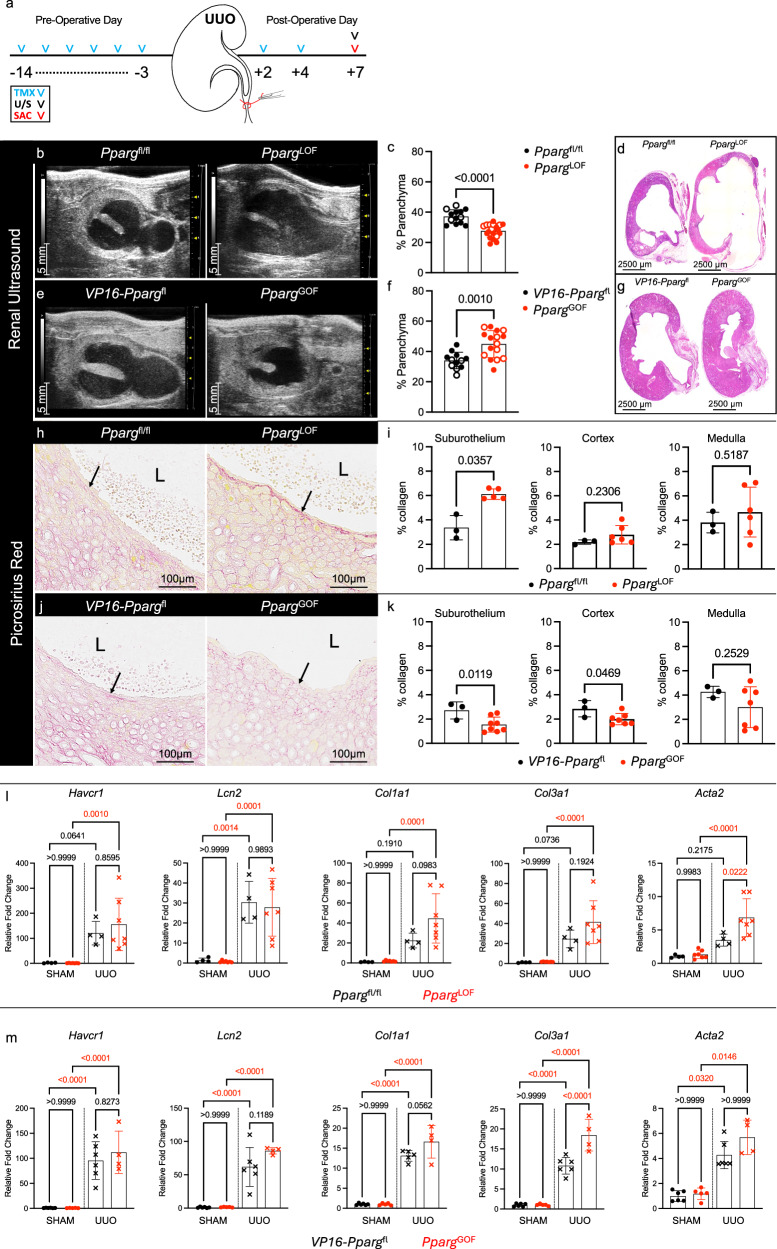
PPARγ activation in renal urothelium promotes renal structural integrity during UUO. **a**, A schematic that depicts the experimental plan. U/S, renal ultrasound; SAC, sacrifice. **b**, Representative sonograms of *Pparg*^fl/fl^ and *Pparg*^LOF^ kidneys at POD7. **c**, The graph shows the fractional parenchyma percentage. *Pparg*^fl/fl^ (*n* = 9 males, *n* = 4 females), *Pparg*^LOF^ (*n* = 13 males, *n* = 7 females). The solid circle represents male, and the hollow circle represents female. The bars are the mean, and the error bars represent the s.d. The *P* values represent an unpaired two-tailed *t*-test. **d**, The representative micrographs of hematoxylin and eosin-stained kidney sections from *Pparg*^fl/fl^ and *Pparg*^LOF^ POD7 kidneys. **e**, The representative sonograms of *VP16-Pparg*^fl^ and *Pparg*^GOF^ kidneys at POD7. **f**, The graph shows the fractional parenchyma percentage. *VP16-Pparg*^fl^ (*n* = 7 males, *n* = 5 females), *Pparg*^GOF^ (*n* = 8 males, *n* = 8 females). The solid circle represents male, and the hollow circle represents female. The bars are the mean, and the error bars represent the s.d. The *P* values represent an unpaired two-tailed *t*-test. **g**, The representative micrographs of hematoxylin and eosin-stained kidney sections from *VP16-Pparg*^fl^ and *Pparg*^GOF^ POD7 kidneys. **h**, The representative micrographs show PSR stain in *Pparg*^fl/fl^ and *Pparg*^LOF^ kidneys at POD7, captured using standard brightfield light. L, lumen. The arrows show the suburothelium collagen. **i**, The graphs show the collagen area (measured using polarized light micrographs) as a percent of the suburothelium compartment, renal cortex and renal medulla areas. *Pparg*^fl/fl^ (*n* = 3 mice), *Pparg*^LOF^ (*n* = 5 mice). The bars are the mean, and the error bars represent the s.d. The *P* value represents an unpaired two-tailed *t*-test, except the suburothelium graph, where a Mann–Whitney test was used due to a nonparametric data distribution in the *Pparg*^LOF^ cohort. **j**, The representative micrographs show PSR stain in *VP16-Pparg*^fl^ and *Pparg*^GOF^ kidneys at POD7, captured using standard brightfield light. The arrows show the suburothelium collagen. **k**, The graphs show the collagen area (measured using polarized light micrographs) as a percent of the suburothelium compartment, renal cortex and renal medulla areas. *VP16-Pparg*^fl^ (*n* = 3 mice), *Pparg*^GOF^ (*n* = 8 mice). The bars are the mean, and the error bars represent the s.d. The *P* values represent an unpaired two-tailed *t*-test. **l**, **m**, The graphs show the relative fold change of *Havcr1*, *Lcn2*, *Col1a1*, *Col3a1* and *Acta2* mRNA expression in sham and UUO (POD7) kidneys from *Pparg*^fl/fl^ and *Pparg*^LOF^ mice (**l**) and *VP16-Pparg*^fl^ and *Pparg*^GOF^ mice (**m**). The bars are the mean, and the error bars represent the s.d. The *P* values represent a one-way ANOVA with a Šídák’s multiple comparisons test.

Prior studies discovered that the urothelial plaque (formed by UPK proteins) promotes renal structural integrity^7^. Since our findings demonstrate that PPARγ regulates UPK expression, we evaluated the impact of conditional *Pparg* deletion and activation on kidney structure during UUO at POD7 (Fig. 7a). Using renal ultrasound to capture images of the longitudinal axis of the kidney and histologic examination, we observed that the renal parenchyma was significantly thinned in *Pparg*^LOF^ mice compared with *Pparg*^fl/fl^ mice at POD7 (Fig. 7b–d and Supplementary Fig. 5). In contrast, *Pparg*^GOF^ mice had significantly more renal parenchyma compared with *VP16-Pparg*^*fl*^ mice at POD7 (Fig. 7e–g and Supplementary Fig. 5). Importantly, we did not observe renal structural defects in these mice before UUO (Supplementary Fig. 4c, d).

Although the link between renal urothelium and parenchymal integrity remains unclear, we measured collagen deposition using PSR-stained sections to assess extracellular matrix accumulation in each experimental group. We observed a significant increase in collagen beneath the renal urothelium (suburothelium collagen) in *Pparg*^*LOF*^ (Fig. 7h, i) and a significant decrease in suburothelium collagen in *Pparg*^*GOF*^ mice (Fig. 7j, k). While the interstitium immediately beneath the urothelium was affected by *Pparg* manipulation, the collagen deposition in the cortical and medullary compartments was less affected (Fig. 7i, k). Since the UUO model is not directly amenable to renal functional measurements, we profiled markers of kidney injury *Havcr1* (encodes KIM-1) and *Lcn2* (encodes NGAL) and extracellular matrix markers *Col1a1* (encodes alpha-1 type I collagen), *Col3a1* (encodes alpha-1 type III collagen) and *Acta2* (encodes smooth muscle alpha (α)-2 actin) using reverse transcription quantitative PCR in whole kidneys. As expected, UUO kidneys expressed higher levels of *Havcr1*, *Lcn2*, *Col1a1*, *Col3a1* and *Acta2* compared with the sham-treated kidneys; however, we did not find significant exacerbation or protection with conditional *Pparg* deletion or activation, respectively (Fig. 7l, m). Together these data indicate that conditional activation of PPARγ in the renal urothelium promotes renal structural integrity during UUO but does not exert widespread protection for the obstructed kidney.

## Discussion

Congenital and acquired UTO cause bladder-like renal urothelium remodeling, which culminates in increased UPK^+^ cells and UPK expression^6–8^. The deletion of UPK^+^ cells or the UPK-containing urothelial plaque exacerbates parenchymal thinning and accelerates renal dysfunction in acquired and congenital UTO, respectively. Therefore, renal urothelium remodeling and increased UPK represent protective adaptations during UTO^7^, but the cellular and molecular bases underlying renal urothelial remodeling were unclear. In this study, we close key knowledge gaps. First, we established that renal UPK^+^ cells function as adult tissue repair progenitors during UUO. Second, we demonstrated that PPARγ promotes GRHL3 and UPK expression in the renal urothelium. Finally, we demonstrated that cell-specific activation of PPARγ promotes renal structural integrity during UTO. These findings enhance our understanding of the renal urothelium and form the foundation for the development of therapies aimed at increasing UPK expression to preserve renal parenchyma.

### UPK^+^ cells are tissue repair progenitors during UUO

Unlike high-turnover epithelia found in the skin and gut, urothelium is nearly quiescent during homeostasis^37–39^, which complicates fate mapping analysis. However, we previously reported that KRT5^+^ cells display a temporally restricted potential to form UPK^+^ cells, such that embryonic (E17-E18), neonatal (P1-P7) and juvenile (P14) KRT5^+^ cells give rise to UPK^+^ cells, while adult KRT5^+^ cells are lineage restricted^15,16^. Mechanical, chemical and bacterial injuries engage robust proliferation to quickly restore damaged urothelium^17,40–46^. In the kidney, congenital and acquired UTO engage a conserved program of renal urothelium remodeling that culminate in increased UPK expression. Here, we used UUO, a surgical UTO model, to precisely control the timing of obstruction and study the cellular and molecular basis of renal urothelium remodeling and publicly available single-cell RNAseq data that spans several UUO time points. Notably, UUO elicits (1) loss of UPK expression at POD1, (2) KRT5^+^ cell expansion at POD2 and (3) increased apical UPK expression and expansion of UPK^+^ cells at ≥POD7 (refs. ^7,8,15^). While loss of UPK expression and KRT5^+^ cell expansion suggests that KRT5^+^ cells may function as tissue repair progenitors during UTO, prior lineage analysis concluded that KRT5^+^ cells did not account for the majority of UPK^+^ cells following UUO^15^. Remarkably, while UPK3A expression is diminished at POD1, we observed that the UPK-lineage remains intact, proliferates and forms apical UPK^+^ cells at POD7. Consistent with bladder urothelium tissue repair studies, our findings demonstrate an important role for renal UPK^+^ cells during UUO^17,19,20^. However, the renal urothelium exhibits distinct differences from the bladder urothelium: apical cells are infrequently binucleated and do not express KRT20. Moreover, basal cells in the renal urothelium express minimal P63 at baseline and widely express KRT14, in contrast to the bladder urothelium.

### Activation of urothelial PPARγ promotes UPK expression during UUO

Our bioinformatics examination predicted activation of PPARγ in UPK^+^ cells during UUO. PPARγ promotes a program of urothelial differentiation in vitro and in vivo that culminates in increased UPK expression^21–26^. Upon ligand binding, the PPARγ–RXRα complex regulates downstream targets by binding to peroxisome proliferator response elements^22,28^. While RXRα expression remains consistent, we show that PPARγ is induced and activated in renal UPK^+^ cells only during UUO. We also determined that PPARγ is indispensable for UUO-induced urothelial differentiation since UPK was reduced in *Pparg*^*LOF*^ mice and increased in *Pparg*^*GOF*^ mice during UUO. These observations are consistent with recent in vivo studies in bladder urothelium, where conditional deletion of *Pparg* was shown to abrogate terminal differentiation, and conditional activation of PPARγ was shown to promote basal urothelial cell differentiation^21,22^.

We used FABP4, a direct transcriptional target of PPARγ, to assess PPARγ activation and deletion in our studies. While FABP4 is induced during UUO and affected by genetic manipulation of *Pparg*, it is unclear whether FABP4 orchestrates a program of urothelial differentiation. Additional direct downstream targets of PPARγ in urothelium include FOXA1 and IRF1, which are known to promote UPK1A, UPK2 and UPK3A expression^25^. While we were unable to detect IRF1 (data not shown), we found that FOXA1 was expressed by renal urothelium at baseline and following UUO and did not appear to be affected by genetic manipulation of *Pparg*. Therefore, it is unlikely that UUO-induced renal urothelium differentiation occurs through a PPARγ–FOXA1-dependent manner. GRHL3 is a PPARγ downstream regulator and is reduced in PPARγ-deficient bladder urothelium^22^. GRHL3 promotes urothelial differentiation and selectively binds to the *Upk2* promoter^29^. Our findings reveal that GRHL3 is induced during UUO and affected by genetic manipulation of *Pparg*. Together these findings support a role for PPARγ–GRHL3 in UUO-induced renal urothelial differentiation. While additional studies are required to functionally validate a role for GRHL3 in renal urothelium differentiation, additional PPARγ downstream regulators must also be investigated.

### Activation of urothelial PPARγ promotes parenchymal integrity during UUO

We observed parenchymal loss in UPK-deficient *Pparg*^*LOF*^ kidneys and parenchymal preservation in UPK-enhanced *Pparg*^*GOF*^ kidneys during UUO. These findings align with our prior work, which established a critical role for UPK in promoting structural integrity within the obstructed kidney^7^. However, the mechanism by which UPK expression impacts parenchymal integrity remain unclear. One possibility is that the UPK plaque promotes cellular compliance^12^; therefore, increased UPK may lead to better accommodation of increased urine volumes during UTO. A second possibility is that the UPK plaque promotes a water-tight urothelial barrier^10–12^; therefore, increased UPK may limit urine permeability during UTO. A third possibility stems from anti-inflammatory and antifibrotic roles for PPARγ in numerous organ systems, including the kidney (reviewed in ref. ^47^). Indeed, our findings reveal increased suburothelial collagen in obstructed *Pparg*^*LOF*^ kidneys, while *Pparg*^*GOF*^ kidneys were characterized by reduced suburothelial collagen. Pharmacologic activation of PPARγ by antidiabetic thiazolidinediones limit inflammation and fibrosis during UTO. Kawai et al. demonstrated that troglitazone-treated mice exhibit reduced transforming growth factor beta 1 (TGFb1), interstitial alpha smooth muscle actin (aSMA) and collagen I during UUO^48^, revealing that PPARγ activation suppresses interstitial inflammation and fibrosis. More recently, Wei et al. used ultrasound-guided delivery of rosiglitazone loaded nanoparticles to show that PPARγ activation reduced collagen deposition and attenuated interstitial fibrosis during UUO in rats^49^. Importantly, our findings, which reveal altered UPK and suburothelial collagen levels in obstructed *Pparg*^*LOF*^ and *Pparg*^*GOF*^ kidneys, highlight the significance of a urothelial-specific contribution to UTO-induced collagen deposition. Future studies are warranted to dissect the interplay between urothelial PPARγ activation and renal compliance, urothelial barrier function and inflammation during UTO.

### Endogenous regulation and activation of PPARγ

Several transcription factors regulate *Pparg* (reviewed in ref. ^50^). In urothelium, KLF5 and the transcriptional regulator Brahma‐related gene 1 (BRG1) are shown to be upstream of PPARγ, and their deficiency in vivo alters UPK expression in the bladder and ureter, respectively^27,36^. Additional studies are needed to clarify the transcriptional regulation of *Pparg* in renal urothelium following UTO.

PPARγ activation occurs predominantly through a ligand-dependent manner. Endogenous ligands include fatty acids and eicosanoids, such as the prostaglandin (PG) metabolite 15-deoxy-∆-^12,14^-prostaglandin J2 (15d-PGJ_2_)^51^. The metabolite 15d-PGJ2 is a terminal metabolite derived from cyclooxygenase (COX)2 metabolism of arachidonic acid (reviewed in ref. ^52^). In addition, 15d-PGJ2 is present in human urine^53^, but it is unclear whether urinary 15d-PGJ2 levels are increased in obstructed kidneys. Furthermore, COX2 is upregulated in bladder urothelium during tissue repair^54,55^, but it is unclear whether 15d-PGJ2 or COX2 are impacted or relevant to UTO-induced renal urothelium remodeling. Additional studies are needed to clarify how PPARγ is endogenously activated in the renal urothelium.

In conclusion, our data show that PPARγ promotes UPK expression in the renal urothelium and that activation of PPARγ in the renal urothelium promotes renal structural integrity during UUO. Further studies are required to delineate events upstream and downstream of PPARγ and whether activation of PPARγ in the obstructed kidney can serve as a therapeutic measure to mitigate parenchymal injury and preserve renal function after unobstruction. Finally, this study compels an investigation of PPARγ in reversible UUO and models of congenital UTO, such as the *Megabladder* mouse^56^, where the impact of urothelial *Pparg* manipulation on kidney function can be investigated.

## Supplementary information

Supplementary Figures and legends

Supplementary Tables 1–3

Supplementary Table 4

Supplementary Table 5

Supplementary Table 6

## References

[1] Chavez-Iniguez, J. S., Navarro-Gallardo, G. J., Medina-Gonzalez, R., Alcantar-Vallin, L. & Garcia-Garcia, G. Acute kidney injury caused by obstructive nephropathy. *Int. J. Nephrol.*10.1155/2020/8846622 (2020). 33312728 10.1155/2020/8846622 PMC7719507

[2] Yang, J. et al. Impact of acute kidney injury on long-term adverse outcomes in obstructive uropathy. *Sci. Rep.* **11**, 23639 (2021). 34880338 10.1038/s41598-021-03033-0 PMC8654816

[3] Chua, A. et al. Kidney transplant practice patterns and outcome benchmarks over 30 years: the 2018 report of the NAPRTCS. *Pediatr. Transplant.*10.1111/petr.13597 (2019). 31657095 10.1111/petr.13597

[4] Chevalier, R. L. & Peters, C. A. Congenital urinary tract obstruction: Proceedings of the State-Of-The-Art Strategic Planning Workshop—National Institutes of Health, Bethesda, Maryland, USA, 11–12 March 2002. *Pediatr. Nephrol.* **18**, 576–606 (2003). 12720078 10.1007/s00467-003-1074-8

[5] Schaeffer, A. J., Freeman, M. & Giambarresi, L. Introduction to the national urology research agenda: a roadmap for priorities in urological disease research. *J. Urol.*10.1016/j.juro.2010.06.046 (2010). 20643437 10.1016/j.juro.2010.06.046

[6] Becknell, B. et al. Molecular basis of renal adaptation in a murine model of congenital obstructive nephropathy. *PloS ONE*10.1371/journal.pone.0072762 (2013). 24023768 10.1371/journal.pone.0072762 PMC3762787

[7] Jackson, A. R. et al. The uroplakin plaque promotes renal structural integrity during congenital and acquired urinary tract obstruction. *Am. J. Physiol. Ren. Physiol.*10.1152/ajprenal.00173.2018 (2018). 10.1152/ajprenal.00173.2018 PMC6230727 29897287

[8] Girshovich, A. et al. Ureteral obstruction promotes proliferation and differentiation of the renal urothelium into a bladder-like phenotype. *Kidney Int.*10.1038/ki.2012.110 (2012). 22513823 10.1038/ki.2012.110

[9] Carpenter, A. R. et al. Uroplakin 1b is critical in urinary tract development and urothelial differentiation and homeostasis. *Kidney Int.*10.1016/j.kint.2015.11.017 (2016). 26880456 10.1016/j.kint.2015.11.017 PMC4757817

[10] Hu, P. et al. Role of membrane proteins in permeability barrier function: uroplakin ablation elevates urothelial permeability. *Am. J. Physiol. Ren. Physiol.*10.1152/ajprenal.00043.2002 (2002). 10.1152/ajprenal.00043.2002 12388410

[11] Hu, P. et al. Ablation of uroplakin III gene results in small urothelial plaques, urothelial leakage, and vesicoureteral reflux. *J. Cell Biol.* **151**, 961–972 (2000). 11085999 10.1083/jcb.151.5.961 PMC2174354

[12] Mathai, J. C. et al. Hypercompliant apical membranes of bladder umbrella cells. *Biophys. J.*10.1016/j.bpj.2014.07.047 (2014). 25229135 10.1016/j.bpj.2014.07.047 PMC4167298

[13] Romih, R., Korosec, P., de Mello, W. Jr & Jezernik, K. Differentiation of epithelial cells in the urinary tract. *Cell Tissue Res.*10.1007/s00441-004-1005-4 (2005). 15778856 10.1007/s00441-004-1005-4

[14] Jackson, A. R., Narla, S. T., Bates, C. M. & Becknell, B. Urothelial progenitors in development and repair. *Pediatr. Nephrol.*10.1007/s00467-021-05239-w (2022). 34471946 10.1007/s00467-021-05239-w PMC8942604

[15] Jackson, A. R. et al. Krt5^+^ urothelial cells are developmental and tissue repair progenitors in the kidney. *Am. J. Physiol. Ren. Physiol.*10.1152/ajprenal.00171.2019 (2019). 10.1152/ajprenal.00171.2019 PMC6766634 31322419

[16] Becknell, B. et al. Keratin 5 basal cells are temporally regulated developmental and tissue repair progenitors in bladder urothelium. Am. J. Physiol. 10.1152/ajprenal.00378.2023 (2024). 10.1152/ajprenal.00378.2023 PMC11386981 38634130

[17] Gandhi, D. et al. Retinoid signaling in progenitors controls specification and regeneration of the urothelium. *Dev. Cell* **26**, 469–482 (2013). 23993789 10.1016/j.devcel.2013.07.017 PMC4024836

[18] Bohnenpoll, T. et al. Diversification of cell lineages in ureter development. *J. Am. Soc. Nephrol.* **28**, 1792–1801 (2017). 28028137 10.1681/ASN.2016080849 PMC5461796

[19] Wang, J. et al. Polyploid superficial cells that maintain the urothelial barrier are produced via incomplete cytokinesis and endoreplication. *Cell Rep.* **25**, 464–477 e464 (2018). 30304685 10.1016/j.celrep.2018.09.042 PMC6351079

[20] Schafer, F. M. et al. Mode of surgical injury influences the source of urothelial progenitors during bladder defect repair. *Stem Cell Rep.*10.1016/j.stemcr.2017.10.025 (2017). 29173895 10.1016/j.stemcr.2017.10.025 PMC5785709

[21] Tate, T. et al. Pparg signaling controls bladder cancer subtype and immune exclusion. *Nat. Commun.* **12**, 6160 (2021). 34697317 10.1038/s41467-021-26421-6 PMC8545976

[22] Liu, C. et al. *Pparg* promotes differentiation and regulates mitochondrial gene expression in bladder epithelial cells. *Nat. Commun.* **10**, 4589 (2019). 31597917 10.1038/s41467-019-12332-0 PMC6785552

[23] Varley, C. L. et al. Role of PPARγ and EGFR signalling in the urothelial terminal differentiation programme. *J. Cell Sci.* **117**, 2029–2036 (2004). 15054105 10.1242/jcs.01042

[24] Varley, C. L., Stahlschmidt, J., Smith, B., Stower, M. & Southgate, J. Activation of peroxisome proliferator-activated receptor-gamma reverses squamous metaplasia and induces transitional differentiation in normal human urothelial cells. *Am. J. Pathol.* **164**, 1789–1798 (2004). 15111325 10.1016/s0002-9440(10)63737-6 PMC1615665

[25] Varley, C. L., Bacon, E. J., Holder, J. C. & Southgate, J. FOXA1 and IRF-1 intermediary transcriptional regulators of PPARgamma-induced urothelial cytodifferentiation. *Cell Death Differ.* **16**, 103–114 (2009). 18688264 10.1038/cdd.2008.116

[26] Santos, C. P. et al. Urothelial organoids originating from Cd49f^high^ mouse stem cells display Notch-dependent differentiation capacity. *Nat. Commun.* **10**, 4407 (2019). 31562298 10.1038/s41467-019-12307-1 PMC6764959

[27] Weiss, R. M. et al. Brg1 determines urothelial cell fate during ureter development. *J. Am. Soc. Nephrol.* **24**, 618–626 (2013). 23449535 10.1681/ASN.2012090902 PMC3609140

[28] Kliewer, S. A., Umesono, K., Noonan, D. J., Heyman, R. A. & Evans, R. M. Convergence of 9-*cis* retinoic acid and peroxisome proliferator signalling pathways through heterodimer formation of their receptors. *Nature* **358**, 771–774 (1992). 1324435 10.1038/358771a0 PMC6159883

[29] Yu, Z., Mannik, J., Soto, A., Lin, K. K. & Andersen, B. The epidermal differentiation-associated Grainyhead gene Get1/Grhl3 also regulates urothelial differentiation. *EMBO J.* **28**, 1890–1903 (2009). 19494835 10.1038/emboj.2009.142 PMC2711180

[30] Shen, T. H. et al. A BAC-based transgenic mouse specifically expresses an inducible Cre in the urothelium. *PloS ONE* **7**, e35243 (2012). 22496911 10.1371/journal.pone.0035243 PMC3322165

[31] Madisen, L. et al. A robust and high-throughput Cre reporting and characterization system for the whole mouse brain. *Nat. Neurosci.* **13**, 133–140 (2010). 20023653 10.1038/nn.2467 PMC2840225

[32] He, W. et al. Adipose-specific peroxisome proliferator-activated receptor gamma knockout causes insulin resistance in fat and liver but not in muscle. *Proc. Natl Acad. Sci. USA* **100**, 15712–15717 (2003). 14660788 10.1073/pnas.2536828100 PMC307633

[33] Bankhead, P. et al. QuPath: open source software for digital pathology image analysis. *Sci. Rep.* **7**, 16878 (2017). 29203879 10.1038/s41598-017-17204-5 PMC5715110

[34] Carpenter, A. R., Becknell, B., Ingraham, S. E. & McHugh, K. M. Ultrasound imaging of the murine kidney. *Methods Mol. Biol.* **886**, 403–410 (2012). 22639280 10.1007/978-1-61779-851-1_35

[35] Li, H., Dixon, E. E., Wu, H. & Humphreys, B. D. Comprehensive single-cell transcriptional profiling defines shared and unique epithelial injury responses during kidney fibrosis. *Cell Metab.* **34**, 1977–1998.e1979 (2022). 36265491 10.1016/j.cmet.2022.09.026 PMC9742301

[36] Bell, S. M. et al. Kruppel-like factor 5 is required for formation and differentiation of the bladder urothelium. *Dev. Biol.* **358**, 79–90 (2011). 21803035 10.1016/j.ydbio.2011.07.020 PMC3180904

[37] Hicks, R. M. The mammalian urinary bladder: an accommodating organ. *Biol. Rev. Camb. Philos. Soc.* **50**, 215–246 (1975). 1100129 10.1111/j.1469-185x.1975.tb01057.x

[38] Jost, S. P. Cell cycle of normal bladder urothelium in developing and adult mice. *Virchows Arch. B* **57**, 27–36 (1989). 2567547 10.1007/BF02899062

[39] Jost, S. P. & Potten, C. S. Urothelial proliferation in growing mice. *Cell Tissue Kinet.* **19**, 155–160 (1986). 3698072 10.1111/j.1365-2184.1986.tb00725.x

[40] Kullmann, F. A. et al. Urothelial proliferation and regeneration after spinal cord injury. *Am. J. Physiol. Ren. Physiol.* **313**, F85–F102 (2017). 10.1152/ajprenal.00592.2016 PMC5538841 28331065

[41] Kreft, M. E., Sterle, M., Veranic, P. & Jezernik, K. Urothelial injuries and the early wound healing response: tight junctions and urothelial cytodifferentiation. *Histochem. Cell Biol.* **123**, 529–539 (2005). 15868181 10.1007/s00418-005-0770-9

[42] Veranic, P. et al. Rapid differentiation of superficial urothelial cells after chitosan-induced desquamation. *Histochem. Cell Biol.* **131**, 129–139 (2009). 18797916 10.1007/s00418-008-0492-x

[43] Romih, R., Koprivec, D., Martincic, D. S. & Jezernik, K. Restoration of the rat urothelium after cyclophosphamide treatment. *Cell Biol. Int.* **25**, 531–537 (2001). 11407858 10.1006/cbir.2000.0658

[44] Shin, K. et al. Hedgehog/Wnt feedback supports regenerative proliferation of epithelial stem cells in bladder. *Nature* **472**, 110–114 (2011). 21389986 10.1038/nature09851 PMC3676169

[45] Papafotiou, G. et al. KRT14 marks a subpopulation of bladder basal cells with pivotal role in regeneration and tumorigenesis. *Nat. Commun.* **7**, 11914 (2016). 27320313 10.1038/ncomms11914 PMC4915139

[46] Balsara, Z. R. & Li, X. Sleeping beauty: awakening urothelium from its slumber. *Am. J. Physiol. Ren. Physiol.* **312**, F732–F743 (2017). 10.1152/ajprenal.00337.2016 PMC5407074 28122714

[47] Kokeny, G., Calvier, L. & Hansmann, G. PPARγ and TGFβ—major regulators of metabolism, inflammation, and fibrosis in the lungs and kidneys. Int. J. Mol. Sci. 10.3390/ijms221910431 (2021). 10.3390/ijms221910431 PMC8508998 34638771

[48] Kawai, T. et al. PPAR-γ agonist attenuates renal interstitial fibrosis and inflammation through reduction of TGF-β. *Lab. Investig.* **89**, 47–58 (2009). 19002105 10.1038/labinvest.2008.104

[49] Wei, S. et al. Ultrasound assisted a peroxisome proliferator-activated receptor (PPAR)gamma agonist-loaded nanoparticle-microbubble complex to attenuate renal interstitial fibrosis. *Int J. Nanomed.* **15**, 7315–7327 (2020). 10.2147/IJN.S262052 PMC7537998 33061383

[50] Lee, J. E. & Ge, K. Transcriptional and epigenetic regulation of PPARgamma expression during adipogenesis. *Cell Biosci.* **4**, 29 (2014). 24904744 10.1186/2045-3701-4-29 PMC4046494

[51] Forman, B. M. et al. 15-Deoxy-delta 12, 14-prostaglandin J2 is a ligand for the adipocyte determination factor PPAR gamma. *Cell* **83**, 803–812 (1995). 8521497 10.1016/0092-8674(95)90193-0

[52] Zhang, Y. et al. Arachidonic acid metabolism in health and disease. *Med. Comm.* **4**, e363 (2023). 10.1002/mco2.363 PMC10511835 37746665

[53] Bell-Parikh, L. C. et al. Biosynthesis of 15-deoxy-delta12,14-PGJ2 and the ligation of PPARgamma. *J. Clin. Investig.* **112**, 945–955 (2003). 12975479 10.1172/JCI18012 PMC193665

[54] Davies, S. S. et al. Oxidized alkyl phospholipids are specific, high affinity peroxisome proliferator-activated receptor gamma ligands and agonists. *J. Biol. Chem.* **276**, 16015–16023 (2001). 11279149 10.1074/jbc.M100878200

[55] Hannan, T. J. et al. Inhibition of cyclooxygenase-2 prevents chronic and recurrent cystitis. *EBioMedicine*10.1016/j.ebiom.2014.10.011 (2014). 26125048 10.1016/j.ebiom.2014.10.011 PMC4457352

[56] Singh, S. et al. Identification of a unique transgenic mouse line that develops megabladder, obstructive uropathy, and renal dysfunction. *J. Am. Soc. Nephrol.* **18**, 461–471 (2007). 17202422 10.1681/ASN.2006040405

