# Supplementary material for: PPARγ promotes urothelial remodeling during urinary tract obstruction
Source: Exp Mol Med. 2025 May 1;57(5):950–63. doi: 10.1038/s12276-025-01441-0 (PMC12130184; doi:10.1038/s12276-025-01441-0)
Supplement: Supplementary file 1 — Supplementary Figures and legends [file 12276_2025_1441_MOESM1_ESM.pdf]

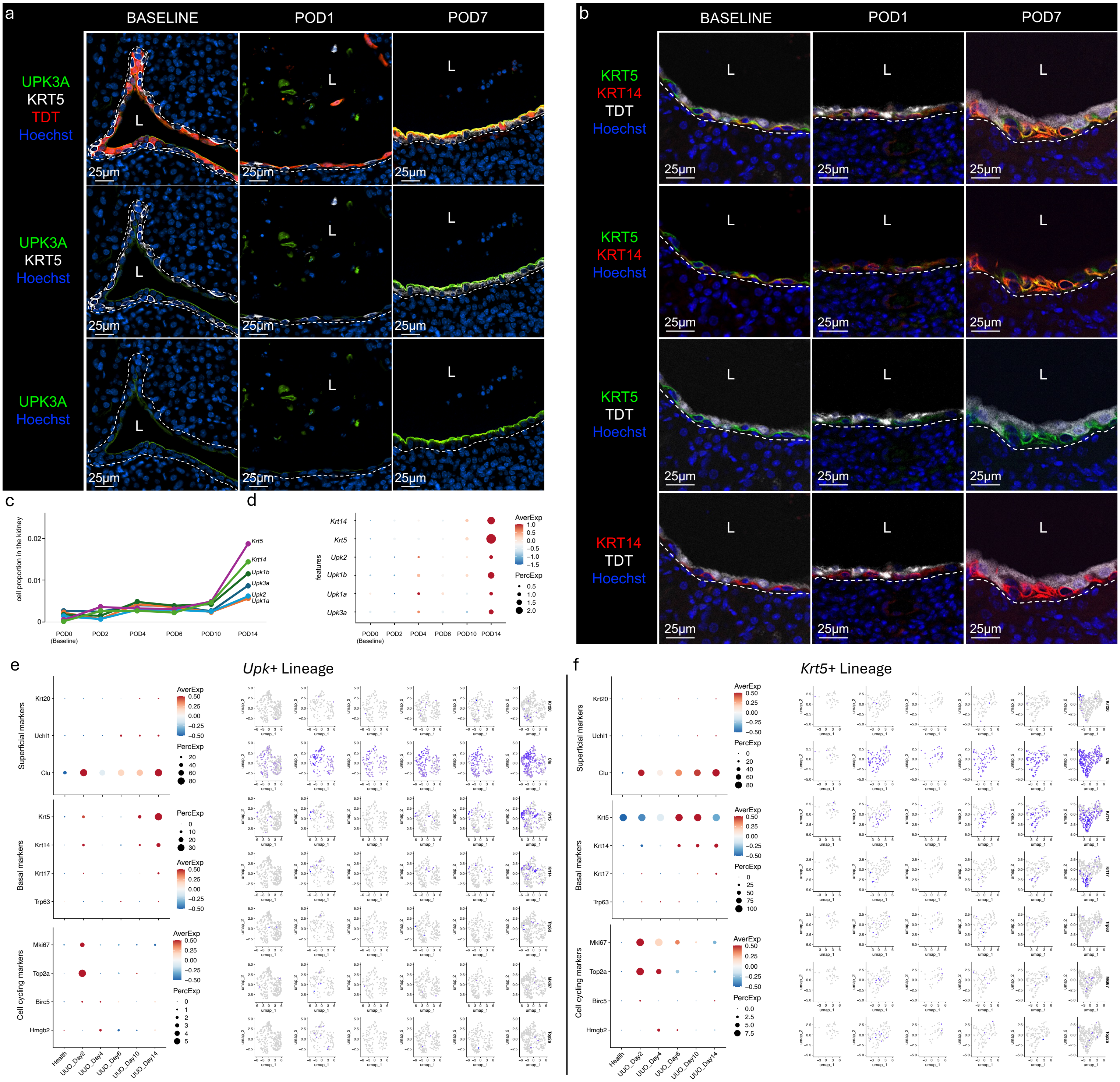

Supplementary Figure 1. Immunofluorescence localization and scRNA-seq analyses of renal urothelium subpopulations at baseline and during UUO.

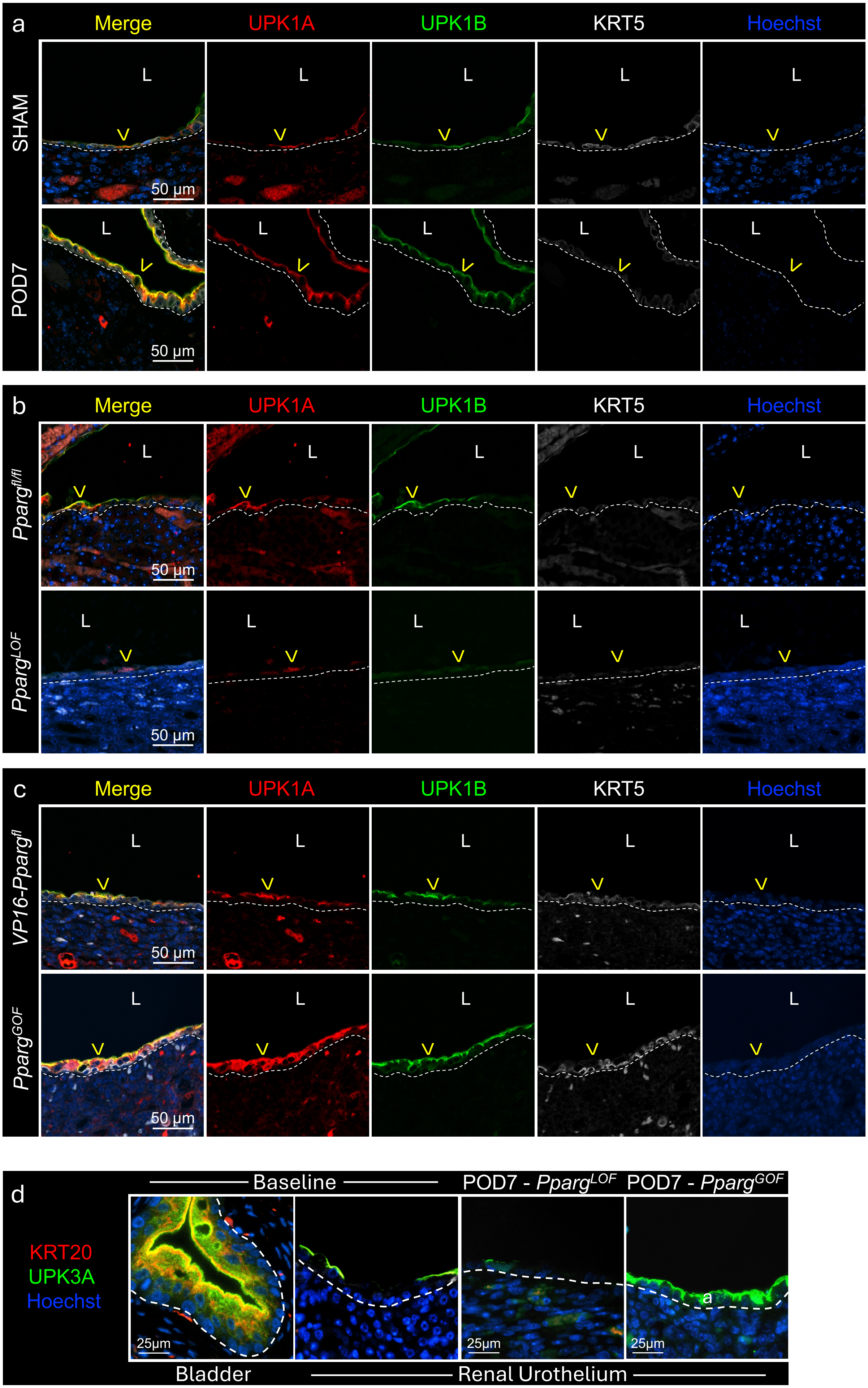

Supplementary Figure 2. UUO leads to increased UPK1A and UPK1B which are altered during conditional manipulation of PPARγ.

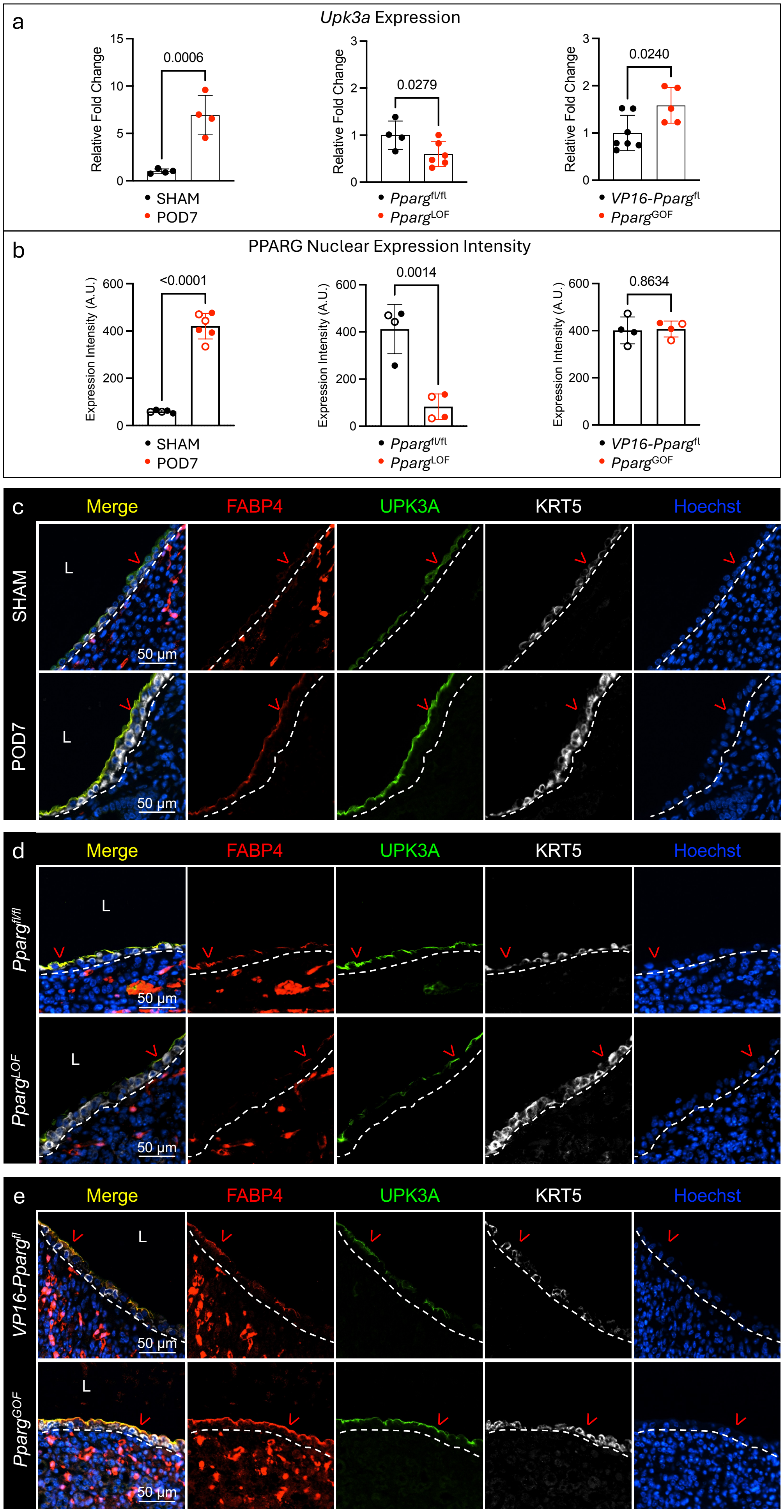

Supplementary Figure 3. UUO leads to increased UPK3A and FABP4 which are altered during conditional manipulation of PPARγ.

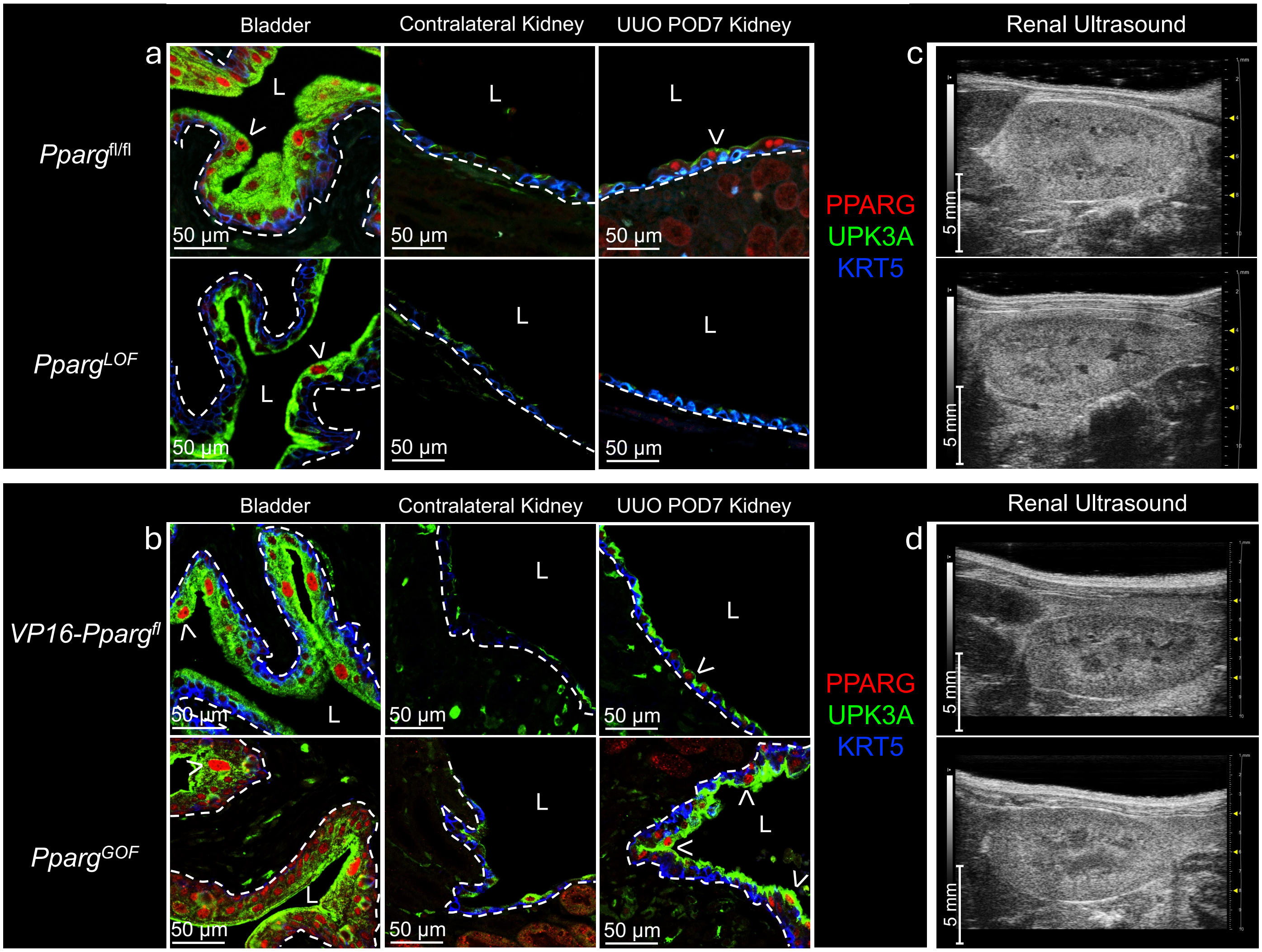

Supplementary Figure 4. Conditional manipulation of PPARγ alters both PPARG and UPK3A expression.

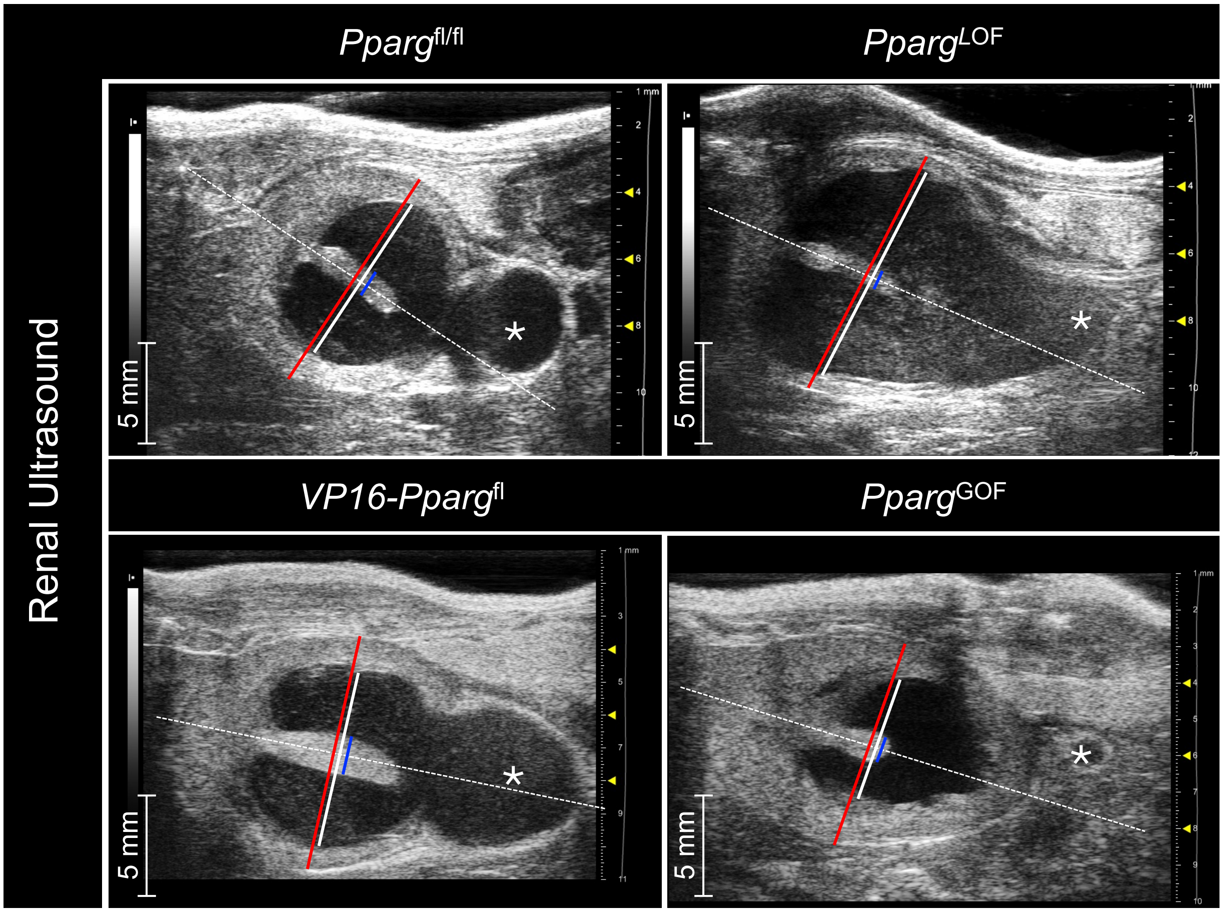

Supplementary Figure 5. Annotation of sonograms for renal parenchyma measurements.

SUPPLEMENTARY FIGURES LEGENDS

Supplementary Figure 1. Immunofluorescence localization and scRNA-seq analyses of renal urothelium subpopulations at baseline and during UUO.

a Representative micrographs show anti-UPK3A, -KRT5, -TDT, and Hoechst labeling in the renal urothelium of *Upk2*^{CreERT2};*R26*$^{tdT/+}$ mice at baseline, POD1, and POD7. White dashed line: renal urothelium basement membrane. L: lumen. **b** Representative micrographs show anti-KRT5, -KRT14, -TDT, and Hoechst labeling in the renal urothelium of *Upk2*^{CreERT2};*R26*$^{tdT/+}$ mice at baseline, POD1, and POD7. White dashed line: renal urothelium basement membrane. L: lumen. **c** Graph shows the proportion of *Upk1a*, *Upk1b*, *Upk2*, *Upk3a*, *Krt5*, and *Krt14* expressing urothelial cells at baseline (POD0) and at UUO POD2, POD4, POD6, POD10, and POD14. **d** Dotplot of canonical urothelial marker genes at baseline and UUO. **e, f** Dotplots and UMAP plots of Superficial, Basal, and Cell cycling markers in (**e**) *Upk*+ or (**f**) *Krt5*+ cells at baseline (health, POD0), and POD2, POD4, POD6, POD10, and POD14.

Supplementary Figure 2. UUO leads to increased UPK1A and UPK1B which are altered during conditional manipulation of PPARγ.

a-c Representative micrographs show anti-UPK1A, -UPK1B, -KRT5, and Hoechst labeling (and individual color channels) in the renal urothelium of SHAM and POD7 kidneys (**a**), *Pparg*$^{fl/fl}$ and *Upk2*^{CreERT2};*Pparg*$^{fl/fl}$ (*Pparg*^{LOF}) mice at POD7 (**b**), and *VP16-Pparg*^{fl} and *Upk2*^{CreERT2};*VP16-Pparg*^{fl} (*Pparg*^{GOF}) mice at POD7 (**c**). White dashed line: renal urothelium basement membrane; L: lumen; Yellow arrowhead: UPK1A+ UPK1B+ cell. SHAM (n=4 mice), POD7 (n=4 mice). **d** Representative micrographs show anti-KRT20, -UPK3A, and Hoechst labeling in the urothelium

of bladder and kidney at baseline, along with the renal urothelium of *Upk2*^{CreERT2};*Pparg*$^{fl/fl}$ (*Pparg*^{LOF}) and *Upk2*^{CreERT2};*VP16-Pparg*^{fl} (*Pparg*^{GOF}) mice at POD7.

Supplementary Figure 3. UUO leads to increased UPK3A and FABP4 which are altered during conditional manipulation of PPARγ.

a Graphs show the relative fold change of *Upk3a* mRNA levels in the kidneys of SHAM and POD7 mice, *Pparg*$^{fl/fl}$ and *Upk2*^{CreERT2};*Pparg*$^{fl/fl}$ (*Pparg*^{LOF}) mice at POD7, and *VP16-Pparg*^{fl} and *Upk2*^{CreERT2};*VP16-Pparg*^{fl} (*Pparg*^{GOF}) mice at POD7, normalized to *Gapdh*. Sham (n=4 mice), POD7 (n=4 mice), *Pparg*$^{fl/fl}$ (n=4 mice), *Pparg*^{LOF} (n=6 mice), *VP16-Pparg*^{fl} (n=7 mice), *Pparg*^{GOF} (n=5 mice). Bars: mean; Error bars: SD; P values: Unpaired Two-Tailed T test. For *VP16-Pparg*^{fl} and *Pparg*^{GOF} plot, Mann-Whitney test was used due to nonparametric data distribution in *VP16-Pparg*^{fl} cohort. **b** Graphs show PPARG nuclear expression intensity in the renal urothelium of SHAM and POD7 mice, *Pparg*$^{fl/fl}$ and *Upk2*^{CreERT2};*Pparg*$^{fl/fl}$ (*Pparg*^{LOF}) mice at POD7, and *VP16-Pparg*^{fl} and *Upk2*^{CreERT2};*VP16-Pparg*^{fl} (*Pparg*^{GOF}) mice at POD7. Sham (n=5 mice), POD7 (n=6 mice), *Pparg*$^{fl/fl}$ (n=4 mice), *Pparg*^{LOF} (n=4 mice), *VP16-Pparg*^{fl} (n=4 mice), *Pparg*^{GOF} (n=4 mice). solid circle: male; hollow circle: female. Bars: mean; Error bars: SD; P values: Unpaired Two-Tailed T test. **c-e** Representative micrographs show anti-FABP4, -UPK3A, -KRT5, and Hoechst labeling (and individual color channels) in the renal urothelium of (**c**) SHAM and POD7 kidneys, (**d**) *Pparg*$^{fl/fl}$ and *Upk2*^{CreERT2};*Pparg*$^{fl/fl}$ (*Pparg*^{LOF}) mice at POD7, and (**e**) *VP16-Pparg*^{fl} and *Upk2*^{CreERT2};*VP16-Pparg*^{fl} (*Pparg*^{GOF}) mice at POD7. White dashed line: renal urothelium basement membrane; L: lumen; Red arrowhead: FABP4+ UPK+ cell.

Supplementary Figure 4. Conditional manipulation of PPARγ alters both PPARG and UPK3A expression.

a Representative micrographs show anti-PPARG, -UPK3A, -KRT5 labeling in bladder, contralateral kidney (normal) renal urothelium and POD7 renal urothelium in *Pparg*$^{fl/fl}$ and *Upk2*^{CreERT2};*Pparg*$^{fl/fl}$ (*Pparg*^{LOF}) mice. White dashed line: urothelium basement membrane; L: lumen. White Arrowhead: PPARG+ UPK+ cell. **b** Representative micrographs show anti-PPARG, -UPK3A, -KRT5 labeling in bladder, contralateral kidney (normal) renal urothelium and POD7 renal urothelium in *VP16-Pparg*^{fl} and *Upk2*^{CreERT2};*VP16-Pparg*^{fl} (*Pparg*^{GOF}) mice. White dashed line: urothelium basement membrane; L: lumen. White Arrowhead: PPARG+ UPK+ cell. **c** Representative sonogram shows kidneys in *Pparg*$^{fl/fl}$ and *Pparg*^{LOF} mice without UUO. **d** Representative sonogram shows kidneys in *VP16-Pparg*^{fl} and *Pparg*^{GOF} mice without UUO.

Supplementary Figure 5. Annotation of sonograms for renal parenchyma measurements.

Annotated longitudinal images of POD7 kidneys. Images are captured at the largest longitudinal plane of the kidney. Dashed white line: longitudinal renal length (LRL – to which all measurements lines are perpendicular); red line: transverse renal width (TRW); white line: renal pelvis diameter (RPD); blue line: renal papilla width (RPW); Asterisk (*): renal pelvis. The fractional percentage of parenchyma is calculated as ([TRW − RPD] + RPW)/TRW.
