# Supplementary material for: PPARγ promotes urothelial remodeling during urinary tract obstruction
Source: Exp Mol Med. 2025 May 1;57(5):950–63. doi: 10.1038/s12276-025-01441-0 (PMC12130184; doi:10.1038/s12276-025-01441-0)
Supplement: Supplementary file 2 — Supplementary Tables 1–3 [file 12276_2025_1441_MOESM2_ESM.pdf]

Supplemental Table 1. Mouse Lines.

Allele	Name in Text	Source	Primers	Product Size
Tg(Upk2-icre/ERT2)1Ccc/J	$Upk2^{CreERT2}$	Jackson Laboratory (stock #024768) [1]	Common Forward:<br>5′-GCGGGAGTTCCAGAAAGAG-3′<br>Wild Type Reverse:<br>5′-AGGACAGCCAGCAGAATCAG-3′<br>Mutant Reverse:<br>5′-AGATCTCCTGTGCAGCATG-3	Wild Type:<br>338 bp<br><br>Mutant:<br>600 bp
B6.Cg-$Gt(ROSA)26Sor^{tm14(CAG-tdTomato)Hze}$/J	$R26^{tdT}$	Jackson Laboratory (stock #007914) [2]	Wild Type forward:<br>5′-AAGGGAGCTGCAGTGGAGTA-3′<br>Wild Type reverse:<br>5′-CCGAAAATCTGTGGGAAGTC-3′<br>Mutant forward:<br>5′-CTGTTCCTGTACGGCATGG-3′<br>Mutant reverse:<br>5′-GGCATTAAAGCAGCGTATCC-3′	Wild Type:<br>297 bp<br><br>Mutant:<br>315 bp
B6.129-$Pparg^{tm2Rev}$/J	$Pparg^{fl/fl}$	Jackson Laboratory (stock #004584) [3]	Mutant Forward<br>5′-TGTAATGGAAGGGCAAAAGG-3′<br>Mutant Reverse:<br>5′-TGGCTTCCAGTGCATAAGTT-3′<br>Wild Type forward:<br>5′-TGTAATGGAAGGGCAAAAGG-3′<br>Wild Type Reverse:<br>5′-TGGCTTCCAGTGCATAAGTT-3′	Wild Type:<br>184 bp<br><br>Mutant:<br>220 bp
ROSA26-CAG-STOP-VP16PPARG-IRES-EGFP	$VP16\text{-}Pparg^{fl/fl}$	Cathy L. Mendelsohn Laboratory, Columbia University [4]	Mutant Forward:<br>5'-CTGCATTCTAGTTGTGGTTTGTCCA-3'<br>Mutant Reverse:<br>5'-ATCGGTAAACATCTGCTCAAACTCG-3'<br>Wild Type Forward:<br>5'-CCCAAAGTCGCTCTGAGTTGTTATC-3'<br>Wild Type Reverse:<br>5'-AACTCGGGTGAGCATGTCTTTAATC-3'	Wild Type:<br>500 bp<br><br>Mutant:<br>300 bp

Supplemental Table 2. Primary Antibodies.

Name (host)	Catalogue #	Company	Concentration
FABP4 (goat)	AF1443-SP	R&D Systems	1:1000
FOXA1 (mouse)	Sc-101058	Santa Cruz Biotechnology	1:400
GRHL3 (rabbit)	Ab221058	Abcam	1:800
KI67 (rabbit)	Ab15580	Abcam	1:400
KRT14 (rabbit)	905304	BioLegend	1:400
KRT20 (mouse)	MA5-13263	Invitrogen	1:50
KRT5 (chicken)	905901	BioLegend	1:500
KRT5 (rabbit-488)	ab193894	Abcam	1:800
KRT5 (rabbit-647)	ab193895	Abcam	1:400
KRT5 (rabbit)	PRB-160P	Covance	1:800
P63 (rabbit)	Sc-8343	Santa Cruz Biotechnology	1:400
PPARG (rabbit)	2435S	Cell Signaling Technology	1:400
RXRA (rabbit)	D6H10	Cell Signaling Technology	1:400
TDT (goat)	MBS448092	MyBioSource	1:500
TDT (rabbit)	600-401-379	Rockland	1:500
UPK1A (goat)	sc-15173	Santa Cruz Biotechnology	1:400
UPK1B (mouse)	WH0007348M2	Sigma Aldrich	1:400
UPK3A (mouse)	10R-U103ax	Fitzgerald	1:200
UPK3A (rabbit)	A10034	AbClonal	1:800

Supplemental Table 3. Mouse Primers Used for RT-qPCR.

Gene	Name in Text	Primers	Product Size
Glyceraldehyde-3-phosphate dehydrogenase	*Gapdh*	Forward:<br>5'- CTGGAGAAACCTGCCAAGTA -3'<br>Reverse<br>5'- TGTTGCTGTAGCCGTATTCA -3'	223 bp
Uroplakin 3a	*Upk3a*	Forward:<br>5'- TCCCACTGAGCACCACTTTC -3'<br>Reverse<br>5'- CCACAAGGGGTCAGGTCAAA -3'	85 bp
Hepatitis A virus cellular receptor 1 encodes kidney injury molecule-1 (KIM-1)	*Havcr1*	Forward:<br>5'- TTTCAGGCCTCATACTGCTTC -3'<br>Reverse:<br>5'- TGTTGAGTAAGTACATGGAAGTGTGA -3'	101 bp
Lipocalin-2 (aka neutrophil gelatinase-associated lipocalin, Ngal)	*Lcn2*	Forward:<br>5'- GCCTCAAGGACGACAACATC -3'<br>Reverse:<br>5'- CTGAACCATTGGGTCTCTGC -3'	109 bp
Smooth muscle alpha (α)-2 actin	*Acta2*	Forward:<br>5'- ACACTGCTGACAGAGGCACCAC -3'<br>Reverse:<br>5'- GCCACATACATGGCGGGGACAT -3'	98 bp
Collagen, type I, alpha 1	*Col1a1*	Forward:<br>5'- TGGACGGCTGCACGAGTCAC -3'<br>Reverse:<br>5'- GCAGGCGGGAGGTCTTGGTG -3'	78 bp
Collagen, type III, alpha 1	*Col3a1*	Forward:<br>5'- CTGGTCAAGCTGGTCCGGCA -3'<br>Reverse:<br>5'- AGGTCCAGGCAGTCCACGCT -3'	125 bp

SUPPLEMENTAL REFERENCES

1 Shen, T. H. *et al.* A BAC-based transgenic mouse specifically expresses an inducible Cre in the urothelium. *PloS one* **7**, e35243, doi:10.1371/journal.pone.0035243 (2012).

2 Madisen, L. *et al.* A robust and high-throughput Cre reporting and characterization system for the whole mouse brain. *Nat Neurosci* **13**, 133-140, doi:10.1038/nn.2467 (2010).

3 He, W. *et al.* Adipose-specific peroxisome proliferator-activated receptor gamma knockout causes insulin resistance in fat and liver but not in muscle. *Proceedings of the National Academy of Sciences of the United States of America* **100**, 15712-15717, doi:10.1073/pnas.2536828100 (2003).

4 Tate, T. *et al.* Pparg signaling controls bladder cancer subtype and immune exclusion. *Nature communications* **12**, 6160, doi:10.1038/s41467-021-26421-6 (2021).
