# Supplementary material for: PPARγ promotes urothelial remodeling during urinary tract obstruction
Source: Exp Mol Med. 2025 May 1;57(5):950–63. doi: 10.1038/s12276-025-01441-0 (PMC12130184; doi:10.1038/s12276-025-01441-0)
Supplement: Supplementary file 3 — Supplementary Table 4 [file 12276_2025_1441_MOESM3_ESM.pdf]

Supplementary Table 4 - DEGs

	Gene	p_val	avg_log2FC	pct.1	pct.2	p_val_adj
1	Prss22	7.73E-11	10.28413993	0.08	0	1.41E-06
2	Krt14	6.01E-13	10.16151165	0.097	0	1.10E-08
3	Mmp7	1.03E-11	9.679579843	0.087	0	1.88E-07
4	Pdlim7	7.73E-11	9.454499028	0.08	0	1.41E-06
5	Trim16	3.85E-10	9.358420612	0.074	0	7.02E-06
6	Tnfrsf12a	1.91E-09	9.22119299	0.069	0	3.48E-05
7	Serpine1	1.51E-07	8.743586302	0.053	0	0.002760209
8	Rbp1	4.24E-09	8.715692453	0.066	0	7.73E-05
9	Col1a1	1.09E-06	8.66021302	0.046	0	0.019951021
10	Il33	3.10E-08	8.549852523	0.059	0	0.00056487
11	Cd200r3	1.09E-06	8.318324796	0.046	0	0.019951021
12	Rdh10	8.22E-08	5.793611816	0.074	0.003	0.001499568
13	Akr1b8	2.66E-16	5.567901855	0.145	0.003	4.85E-12
14	Sprr2f	2.61E-18	5.264839081	0.16	0.003	4.76E-14
15	Cyp1b1	3.27E-14	5.205460807	0.128	0.003	5.97E-10
16	Micu1	5.49E-07	5.120938215	0.067	0.003	0.010023416
17	Cadm4	2.63E-07	4.487189685	0.07	0.003	0.00479807
18	Eif6	8.45E-08	4.380300145	0.074	0.003	0.00154049
19	Tsc22d1	3.11E-10	4.33038836	0.105	0.006	5.67E-06
20	Sprr2a3	2.11E-15	4.268242102	0.174	0.016	3.85E-11
21	Actn1	3.46E-11	4.138268927	0.131	0.013	6.31E-07
22	Ivl	1.23E-19	4.059174792	0.201	0.013	2.24E-15
23	Lcn2	1.57E-14	4.044946269	0.129	0.003	2.87E-10
24	Clic4	3.01E-08	3.961937261	0.111	0.016	0.000549139
25	Flna	2.15E-10	3.960118127	0.124	0.013	3.91E-06
26	Krt15	4.34E-16	3.731637649	0.202	0.025	7.92E-12
27	Tpm4	1.84E-07	3.649489649	0.097	0.013	3.36E-03
28	Krt5	4.51E-14	3.512777731	0.191	0.028	8.22E-10
29	Cdkn1a	1.43E-16	3.448905162	0.192	0.019	2.61E-12
30	Cp	1.13E-08	3.341799405	0.108	0.013	2.07E-04
31	Ier3	2.70E-08	3.289610332	0.097	0.009	4.93E-04
32	Ptgs1	3.00E-15	3.127966408	0.188	0.022	5.47785E-11
33	Ywhag	2.02E-08	3.122510355	0.079	0.003	3.68E-04
34	Tubb5	1.58E-09	2.997785951	0.124	0.016	2.8781E-05
35	Prss27	1.02E-11	2.973515497	0.117	0.006	1.85251E-07
36	Map4	2.26E-08	2.90504538	0.105	0.013	0.000413149
37	Pmepa1	2.47E-27	2.87104115	0.344	0.05	4.50415E-23
38	Dstn	9.34E-08	2.805360434	0.114	0.019	1.70E-03
39	Ctsl	1.89E-06	2.79982562	0.094	0.016	3.45E-02
40	Rac1	6.66E-08	2.788164272	0.115	0.019	1.22E-03
41	Tmsb10	1.54E-06	2.721245801	0.072	0.006	0.02817588
42	Mgst3	1.22E-07	2.653087568	0.09	0.009	0.002224784
43	Sh3bgrl3	5.41E-10	2.602352393	0.142	0.022	9.86552E-06

44	S100a14	6.43E-11	2.498082676	0.135	0.016	1.17E-06
45	Clu	5.79E-52	2.466293582	0.761	0.349	1.06E-47
46	Mgp	1.19E-08	2.448058696	0.115	0.016	2.18E-04
47	Akr1b3	1.86E-18	2.408475375	0.226	0.028	3.39E-14
48	Apoe	3.56E-08	2.397515617	0.094	0.009	6.49E-04
49	Mmp14	1.01E-06	2.255149392	0.081	0.009	0.018503918
50	1110008P14Rik	2.68E-06	2.195783119	0.077	0.009	4.89E-02
51	S100a10	1.48E-06	2.192579662	0.08	0.009	2.70E-02
52	Klf6	3.95E-11	2.140604354	0.195	0.044	7.21E-07
53	Serpinb6a	6.05E-08	2.140547141	0.1	0.013	1.10E-03
54	Anxa1	9.73E-11	2.133701993	0.133	0.016	1.78E-06
55	Sfn	2.93E-17	1.990306017	0.274	0.057	5.34E-13
56	Sox4	1.81E-09	1.928281274	0.205	0.06	3.29E-05
57	Tmem176b	1.44E-10	1.902344336	0.237	0.072	2.63012E-06
58	Gsto1	1.51E-20	1.891605434	0.348	0.085	2.76E-16
59	Basp1	2.26E-20	1.858055766	0.257	0.038	4.12688E-16
60	Krt8	7.67E-33	1.835846553	0.5	0.123	1.39907E-28
61	Taldo1	2.55E-13	1.827981329	0.208	0.041	4.65519E-09
62	Junb	1.21E-06	1.784093685	0.125	0.031	0.022064021
63	Cldn4	6.17E-14	1.747627286	0.235	0.053	1.13E-09
64	Adam10	1.26E-06	1.71493923	0.1	0.019	2.29E-02
65	Uchl1	3.90E-07	1.6831284	0.164	0.05	7.11E-03
66	Capg	2.47E-11	1.653749574	0.143	0.019	4.49747E-07
67	Cfl1	1.97E-15	1.643344247	0.261	0.06	3.6003E-11
68	Fam129b	5.34E-07	1.62562788	0.152	0.044	0.009738888
69	Ucp2	4.35E-08	1.616474663	0.169	0.047	7.93E-04
70	Las1l	2.03E-07	1.60743582	0.121	0.025	3.70E-03
71	Myl12a	4.80E-08	1.599402611	0.167	0.047	8.76E-04
72	Gsta4	2.30E-14	1.590641509	0.372	0.138	4.19E-10
73	Ccnd2	6.44E-15	1.576694078	0.386	0.145	1.17E-10
74	Ctr9	2.23E-09	1.570237258	0.139	0.025	4.07289E-05
75	Krt19	9.58E-28	1.552345275	0.617	0.255	1.75E-23
76	Srd5a1	1.36E-08	1.504793399	0.157	0.038	0.000248858
77	Cstb	5.08E-08	1.479891958	0.125	0.025	0.000926127
78	Cd74	5.13E-08	1.461926722	0.17	0.05	0.000935822
79	Capzb	1.76E-10	1.454991552	0.169	0.035	3.22E-06
80	Sprr1a	2.59E-19	1.450352269	0.626	0.336	4.73E-15
81	Cers3	1.64E-07	1.442349613	0.107	0.019	3.00E-03
82	Anxa3	1.52E-12	1.411420022	0.181	0.031	2.77E-08
83	Mgat4a	2.34E-08	1.391235749	0.164	0.044	4.26E-04
84	Itga6	1.05E-06	1.390512696	0.101	0.019	1.91E-02
85	Rtn4	1.19E-07	1.366739381	0.202	0.072	2.18E-03
86	Myof	2.33E-11	1.363948532	0.302	0.11	4.26E-07
87	Txn1	8.57E-07	1.34965896	0.184	0.066	0.015632943
88	Eif5a	4.30E-07	1.338258643	0.167	0.053	7.84E-03

89	Pkm	7.00E-10	1.333602721	0.277	0.104	1.277E-05
90	Idh1	2.30E-06	1.324639961	0.121	0.031	0.041955232
91	Sdc1	2.74E-08	1.300270432	0.157	0.041	0.000499205
92	Cd24a	5.46E-08	1.269631794	0.129	0.028	0.000996764
93	Eef1g	4.40E-07	1.252283359	0.166	0.053	8.03E-03
94	Serpinb6b	2.13E-08	1.252033449	0.191	0.06	3.88E-04
95	Wfdc2	2.68E-17	1.247324135	0.535	0.248	4.89E-13
96	Eef1b2	1.91E-07	1.216933729	0.173	0.057	0.003485629
97	Rpl18a	1.24E-08	1.205540332	0.135	0.028	0.000226004
98	Capns1	1.43E-11	1.205317714	0.236	0.069	2.61231E-07
99	Arf1	1.51E-06	1.204598591	0.128	0.035	2.76E-02
100	Calm1	9.20E-11	1.202849398	0.258	0.088	1.68E-06
101	Ctnnb1	1.46E-07	1.190621774	0.237	0.097	2.67E-03
102	Krt18	7.48E-16	1.188874883	0.475	0.208	1.36E-11
103	Elf3	1.32E-10	1.154910334	0.185	0.044	2.40E-06
104	Fam25c	6.49E-07	1.135035258	0.162	0.053	0.011838068
105	Arpc2	9.86E-11	1.07593653	0.24	0.079	1.80E-06
106	Arpc1b	7.35E-08	1.057777535	0.171	0.053	1.34E-03
107	Rack1	3.03E-11	1.051503362	0.239	0.075	5.52E-07
108	Tmem176a	4.59E-08	1.046919723	0.169	0.05	8.37E-04
109	Lgals3	7.52E-20	1.040082869	0.365	0.104	1.37E-15
110	Jup	3.91E-09	1.03520183	0.162	0.041	7.13E-05
111	S100a11	5.41E-19	1.00821146	0.399	0.132	9.87E-15
112	Rab11a	1.30E-06	1.004391203	0.121	0.031	0.023703413
113	Isoc1	4.81E-08	0.996451178	0.149	0.038	8.78E-04
114	Rpl7	9.94E-07	0.992878499	0.133	0.038	0.018131914
115	Rps13	1.15E-06	0.981764673	0.129	0.035	0.021047398
116	S100a6	5.56E-17	0.960451493	0.64	0.352	1.01353E-12
117	Fmo5	3.27E-08	0.945416359	0.389	0.208	0.000597298
118	Ldhb	5.82E-07	0.942741881	0.204	0.082	1.06E-02
119	Cdc42	5.16E-07	0.936087857	0.187	0.069	9.41E-03
120	Krt7	2.41E-16	0.916703538	0.497	0.226	4.39E-12
121	Rpl11	1.90E-06	0.893223284	0.146	0.047	0.034569068
122	Rplp0	5.52E-07	0.890269168	0.219	0.091	0.010061502
123	Tagln2	3.35E-07	0.874859214	0.097	0.016	0.006119299
124	Clic1	4.60E-08	0.872576322	0.133	0.031	8.40E-04
125	Tmsb4x	1.09E-13	0.859732177	0.447	0.204	1.98E-09
126	Actb	5.11E-13	0.834646631	0.604	0.352	9.32E-09
127	Ywhae	6.05E-11	0.822695801	0.194	0.05	1.10E-06
128	Gsn	1.95E-10	0.822690843	0.235	0.079	3.57E-06
129	Cldn7	9.94E-10	0.81623366	0.162	0.041	1.81265E-05
130	Ly6a	1.34E-07	0.794354001	0.209	0.079	2.45E-03
131	Prdx2	1.37E-06	0.76530838	0.17	0.063	2.51E-02
132	Bsg	3.97E-09	0.762823436	0.277	0.116	7.25E-05
133	Myh9	1.32E-11	0.752180635	0.368	0.167	2.41E-07

134	Pea15a	2.41E-06	0.688736848	0.084	0.016	4.39E-02
135	Rpl14	8.20E-09	0.666948752	0.226	0.088	1.50E-04
136	Rab10	2.89E-07	0.665318649	0.153	0.047	5.27E-03
137	Rps9	6.63E-07	0.655410462	0.18	0.066	0.012090465
138	Hspa8	1.47E-07	0.650808043	0.282	0.138	2.68E-03
139	Vcp	3.21E-07	0.642514714	0.242	0.11	0.005849135
140	Rps5	1.06E-08	0.64069192	0.235	0.091	0.000193254
141	Iqgap1	2.36E-06	0.628253244	0.271	0.142	0.043129317
142	Anxa2	5.57E-11	0.61705443	0.301	0.119	1.01664E-06
143	Ly6d	1.03E-07	0.599109447	0.268	0.123	1.88E-03
144	Rpsa	9.20E-09	0.595451697	0.256	0.11	1.68E-04
145	Tmem123	1.28E-06	0.583934509	0.183	0.079	2.34E-02
146	Itm2b	2.33E-09	-0.59296992	0.347	0.242	4.24627E-05
147	Atp1b1	1.78E-06	-0.76939681	0.35	0.396	0.032397321
148	Spp1	7.06E-18	-0.79581392	0.604	0.447	1.28866E-13
149	Sptbn1	7.62E-07	-0.83272918	0.292	0.283	1.39E-02
150	Nupr1	3.85E-07	-1.22785677	0.253	0.261	7.02E-03
151	Fth1	8.18E-07	-1.26975374	0.206	0.261	1.49E-02
152	Pdzk1ip1	4.93E-10	-1.3989	0.295	0.352	9.00E-06
153	Fxyd2	2.63E-07	-1.58137152	0.124	0.233	4.81E-03
154	Lars2	3.60E-09	-1.58287917	0.213	0.258	6.57216E-05
155	Kap	1.48E-32	-1.97999352	0.395	0.613	2.70E-28
156	Timp3	9.14E-08	-2.01531125	0.136	0.22	0.001667725
157	Gpx3	3.13E-46	-2.03021706	0.437	0.692	5.71391E-42
158	Lrp2	1.21E-06	-2.14225296	0.02	0.094	0.022119808
159	Slc12a2	3.61E-07	-2.20496603	0.101	0.195	6.58E-03
160	Acsm2	7.69E-15	-2.20755714	0.118	0.302	1.40E-10
161	Miox	7.41E-09	-2.36741248	0.07	0.192	0.000135224
162	Slc34a1	3.31E-25	-2.4041251	0.133	0.412	6.03176E-21
163	Slc12a1	8.63E-08	-2.57029437	0.045	0.148	1.57E-03
164	Fut9	7.53E-07	-2.75746509	0.055	0.148	1.37E-02
165	Egf	4.95E-07	-2.95850153	0.017	0.091	0.009030532
166	S100g	5.66E-07	-3.18629323	0.024	0.104	1.03E-02
167	Lpl	2.80E-09	-4.4290153	0.015	0.101	5.11566E-05
168	Slc6a19	5.67E-07	-5.62683359	0.001	0.047	0.010351705
