# Supplementary material for: PPARγ promotes urothelial remodeling during urinary tract obstruction
Source: Exp Mol Med. 2025 May 1;57(5):950–63. doi: 10.1038/s12276-025-01441-0 (PMC12130184; doi:10.1038/s12276-025-01441-0)
Supplement: Supplementary file 4 — Supplementary Table 5 [file 12276_2025_1441_MOESM4_ESM.pdf]

Supplementary Table 5 - GO Terms

Gene Ontology ID	Description	GeneRatio	BgRatio	pvalue	p.adjust	qvalue	geneID	Count
GO:0043588	skin development	18/158	340/28891	5.79E-13	1.93E-09	1.24E-09	Ivl/Sprr1a/Sprr2f/Sfn/Cdkn1a/Krt7/Ptgs1/Krt5/Cldn4/Krt14/Anxa1/Jup/Clic4/Ctnnb1/Cers3/Itga6/Col1a1/Ctsl	18
GO:0007015	actin filament organization	20/158	471/28891	1.64E-12	2.73E-09	1.76E-09	Cfl1/Tmsb4x/Myh9/Capg/Actn1/Arpc2/Capzb/Gsn/Flna/Rac1/Arpc1b/Dstn/Tpm4/Tagln2/Cdc42/Sptbn1/S100a10/Arf1/Tmsb10/Iqgap1	20
GO:0072659	protein localization to plasma membrane	16/158	315/28891	2.22E-11	1.90E-08	1.22E-08	Lgals3/Krt18/Actb/Rack1/Anxa2/Flna/Jup/Bsg/Myl12a/Rac1/Rab10/Sptbn1/Mmp14/Rab11a/S100a10/Atp1b1	16
GO:0031589	cell-substrate adhesion	17/158	368/28891	2.28E-11	1.90E-08	1.22E-08	Spp1/Cfl1/Myh9/Actn1/Arpc2/Flna/Tnfrsf12a/Jup/Rac1/Ctnnb1/Serpine1/Cdc42/Mmp14/Itga6/Col1a1/S100a10/Iqgap1	17
GO:0010810	regulation of cell-substrate adhesion	14/158	228/28891	3.42E-11	2.09E-08	1.34E-08	Spp1/Cfl1/Myh9/Arpc2/Flna/Jup/Rac1/Serpine1/Cdc42/Mmp14/Itga6/Col1a1/S100a10/Iqgap1	14
GO:0043254	regulation of protein-containing complex assembly	18/158	437/28891	3.75E-11	2.09E-08	1.34E-08	Clu/Lgals3/Cfl1/Tmsb4x/Capg/Rack1/Arpc2/Capzb/Gsn/Cldn7/Apoe/Cd24a/Rac1/Hspa8/Vcp/Cdc42/Sptbn1/Tmsb10	18
GO:1902903	regulation of supramolecular fiber organization	17/158	393/28891	6.35E-11	2.79E-08	1.79E-08	Clu/Cfl1/Tmsb4x/Capg/Arpc2/Capzb/Gsn/Flna/Apoe/Rac1/Dstn/Hspa8/Cdc42/Sptbn1/S100a10/Arf1/Tmsb10	17
GO:0031333	negative regulation of protein-containing complex assembly	12/158	157/28891	7.22E-11	2.79E-08	1.79E-08	Clu/Cfl1/Tmsb4x/Capg/Arpc2/Capzb/Gsn/Cldn7/Hspa8/Cdc42/Sptbn1/Tmsb10	12
GO:0050900	leukocyte migration	17/158	398/28891	7.72E-11	2.79E-08	1.79E-08	Lgals3/Spp1/S100a14/Anxa1/Bsg/Il33/Cd74/Cd24a/Rac1/Rtn4/Serpine1/Slc12a2/Cdc42/Fut9/Mmp14/Itga6/Adam10	17
GO:1990778	protein localization to cell periphery	17/158	400/28891	8.34E-11	2.79E-08	1.79E-08	Lgals3/Krt18/Actb/Rack1/Anxa2/Flna/Jup/Bsg/Myl12a/Rac1/Rab10/Sptbn1/Mmp14/Adam10/Rab11a/S100a10/Atp1b1	17
GO:0030216	keratinocyte differentiation	12/158	163/28891	1.12E-10	3.39E-08	2.18E-08	Ivl/Sprr1a/Sprr2f/Sfn/Cdkn1a/Krt7/Ptgs1/Krt5/Krt14/Anxa1/Clic4/Cers3	12
GO:1903034	regulation of response to wounding	12/158	174/28891	2.39E-10	6.65E-08	4.27E-08	Cldn4/Anxa2/Anxa1/Flna/Tnfrsf12a/Il33/Apoe/Cd24a/Serpine1/Cadm4/Slc12a2/Prdx2	12
GO:0010811	positive regulation of cell-substrate adhesion	11/158	140/28891	3.41E-10	8.75E-08	5.62E-08	Spp1/Cfl1/Myh9/Arpc2/Flna/Jup/Rac1/Cdc42/Itga6/S100a10/Iqgap1	11
GO:0110053	regulation of actin filament organization	14/158	280/28891	5.14E-10	1.23E-07	7.87E-08	Cfl1/Tmsb4x/Capg/Arpc2/Capzb/Gsn/Flna/Rac1/Dstn/Cdc42/Sptbn1/S100a10/Arf1/Tmsb10	14
GO:0008544	epidermis development	16/158	397/28891	6.67E-10	1.48E-07	9.53E-08	Ivl/Sprr1a/Sprr2f/Sfn/Cdkn1a/Krt7/Ptgs1/Krt5/Cldn4/Krt14/Anxa1/Clic4/Rac1/Ctnnb1/Cers3/Ctsl	16
GO:0032970	regulation of actin filament-based process	16/158	402/28891	7.99E-10	1.67E-07	1.07E-07	Cfl1/Tmsb4x/Myh9/Capg/Arpc2/Capzb/Gsn/Flna/Jup/Rac1/Dstn/Cdc42/Sptbn1/S100a10/Arf1/Tmsb10	16
GO:0032535	regulation of cellular component size	16/158	411/28891	1.10E-09	2.16E-07	1.39E-07	Cfl1/Tmsb4x/Capg/Arpc2/Capzb/Gsn/Tnfrsf12a/Apoe/Rac1/Slc12a1/Dstn/Rtn4/Slc12a2/Sptbn1/Rab11a/Tmsb10	16
GO:0009913	epidermal cell differentiation	13/158	252/28891	1.50E-09	2.78E-07	1.78E-07	Ivl/Sprr1a/Sprr2f/Sfn/Cdkn1a/Krt7/Ptgs1/Krt5/Krt14/Anxa1/Clic4/Rac1/Cers3	13
GO:0045862	positive regulation of proteolysis	15/158	372/28891	2.32E-09	4.08E-07	2.62E-07	Clu/Cfl1/Cldn4/Myh9/Rack1/Prss22/Gsn/Il33/Apoe/Vcp/Nupr1/Egf/Mmp14/S100a10/Ctsl	15
GO:0030837	negative regulation of actin filament polymerization	8/158	68/28891	3.75E-09	6.25E-07	4.02E-07	Cfl1/Tmsb4x/Capg/Arpc2/Capzb/Gsn/Sptbn1/Tmsb10	8
GO:1902904	negative regulation of supramolecular fiber organization	11/158	178/28891	4.37E-09	6.95E-07	4.46E-07	Clu/Cfl1/Tmsb4x/Capg/Arpc2/Capzb/Gsn/Apoe/Hspa8/Sptbn1/Tmsb10	11
GO:0001667	ameboidal-type cell migration	16/158	471/28891	7.64E-09	1.16E-06	7.44E-07	Cfl1/Lcn2/Cyp1b1/Anxa3/Myh9/Anxa1/Jup/Bsg/Apoe/Rac1/Rtn4/Serpine1/Egf/Cdc42/Rab11a/Iqgap1	16
GO:0032956	regulation of actin cytoskeleton organization	14/158	351/28891	9.34E-09	1.33E-06	8.56E-07	Cfl1/Tmsb4x/Capg/Arpc2/Capzb/Gsn/Flna/Rac1/Dstn/Cdc42/Sptbn1/S100a10/Arf1/Tmsb10	14
GO:0002181	cytoplasmic translation	10/158	148/28891	9.58E-09	1.33E-06	8.56E-07	Pkm/Rpl14/Rpsa/Rps5/Rpl18a/Rplp0/Rps9/Rpl7/Rps13/Rpl11	10
GO:0042060	wound healing	14/158	370/28891	1.81E-08	2.40E-06	1.54E-06	Cdkn1a/Cldn4/Myh9/Myof/Anxa2/Anxa1/Flna/Tnfrsf12a/Apoe/Serpine1/Cadm4/Slc12a2/Prdx2/S100a10	14
GO:0051014	actin filament severing	5/158	16/28891	1.91E-08	2.40E-06	1.54E-06	Cfl1/Myh9/Capg/Gsn/Dstn	5
GO:2001233	regulation of apoptotic signaling pathway	15/158	437/28891	2.01E-08	2.40E-06	1.54E-06	Clu/Lgals3/Rack1/Tnfrsf12a/Ier3/Cd74/Timp3/Ctnnb1/Serpine1/Nupr1/Itga6/Prdx2/Ctsl/Rpl11/Pea15a	15
GO:0032272	negative regulation of protein polymerization	8/158	84/28891	2.05E-08	2.40E-06	1.54E-06	Cfl1/Tmsb4x/Capg/Arpc2/Capzb/Gsn/Sptbn1/Tmsb10	8
GO:0030834	regulation of actin filament depolymerization	7/158	55/28891	2.08E-08	2.40E-06	1.54E-06	Cfl1/Capg/Arpc2/Capzb/Gsn/Dstn/Sptbn1	7
GO:0008064	regulation of actin polymerization or depolymerization	10/158	166/28891	2.87E-08	3.20E-06	2.05E-06	Cfl1/Tmsb4x/Capg/Arpc2/Capzb/Gsn/Rac1/Dstn/Sptbn1/Tmsb10	10
GO:0030832	regulation of actin filament length	10/158	169/28891	3.41E-08	3.52E-06	2.26E-06	Cfl1/Tmsb4x/Capg/Arpc2/Capzb/Gsn/Rac1/Dstn/Sptbn1/Tmsb10	10
GO:0030042	actin filament depolymerization	7/158	59/28891	3.44E-08	3.52E-06	2.26E-06	Cfl1/Capg/Arpc2/Capzb/Gsn/Dstn/Sptbn1	7
GO:0010631	epithelial cell migration	13/158	328/28891	3.48E-08	3.52E-06	2.26E-06	Lcn2/Cyp1b1/Anxa3/Myh9/Anxa1/Jup/Bsg/Apoe/Rac1/Rtn4/Serpine1/Egf/Rab11a	13
GO:0090132	epithelium migration	13/158	330/28891	3.73E-08	3.63E-06	2.33E-06	Lcn2/Cyp1b1/Anxa3/Myh9/Anxa1/Jup/Bsg/Apoe/Rac1/Rtn4/Serpine1/Egf/Rab11a	13
GO:0061041	regulation of wound healing	9/158	128/28891	3.81E-08	3.63E-06	2.33E-06	Cldn4/Anxa2/Anxa1/Tnfrsf12a/Apoe/Serpine1/Cadm4/Slc12a2/Prdx2	9
GO:0090130	tissue migration	13/158	332/28891	4.00E-08	3.71E-06	2.39E-06	Lcn2/Cyp1b1/Anxa3/Myh9/Anxa1/Jup/Bsg/Apoe/Rac1/Rtn4/Serpine1/Egf/Rab11a	13
GO:0051222	positive regulation of protein transport	13/158	346/28891	6.48E-08	5.85E-06	3.75E-06	Sfn/Myh9/Rack1/Ywhae/Arpc2/Flna/Sox4/Jup/Bsg/Rac1/Cdc42/Lrp2/Arf1	13
GO:0007160	cell-matrix adhesion	11/158	235/28891	7.68E-08	6.75E-06	4.34E-06	Cfl1/Myh9/Actn1/Jup/Rac1/Ctnnb1/Serpine1/Mmp14/Itga6/S100a10/Iqgap1	11
GO:0051693	actin filament capping	6/158	40/28891	8.00E-08	6.85E-06	4.40E-06	Cfl1/Capg/Arpc2/Capzb/Gsn/Sptbn1	6
GO:0097191	extrinsic apoptotic signaling pathway	11/158	242/28891	1.03E-07	8.63E-06	5.54E-06	Krt8/Lgals3/Krt18/Lcn2/Tnfrsf12a/Il33/Timp3/Serpine1/Itga6/Prdx2/Pea15a	11
GO:1904951	positive regulation of establishment of protein localization	13/158	362/28891	1.09E-07	8.90E-06	5.72E-06	Sfn/Myh9/Rack1/Ywhae/Arpc2/Flna/Sox4/Jup/Bsg/Rac1/Cdc42/Lrp2/Arf1	13
GO:0030833	regulation of actin filament polymerization	9/158	146/28891	1.19E-07	9.43E-06	6.06E-06	Cfl1/Tmsb4x/Capg/Arpc2/Capzb/Gsn/Rac1/Sptbn1/Tmsb10	9
GO:0032984	protein-containing complex disassembly	11/158	248/28891	1.32E-07	1.03E-05	6.60E-06	Cfl1/Capg/Calm1/Arpc2/Capzb/Gsn/Dstn/Hspa8/Vcp/Eif5a/Sptbn1	11
GO:0002685	regulation of leukocyte migration	11/158	249/28891	1.38E-07	1.05E-05	6.71E-06	Lgals3/S100a14/Anxa1/Il33/Cd74/Rac1/Rtn4/Serpine1/Fut9/Mmp14/Adam10	11
GO:0010632	regulation of epithelial cell migration	11/158	250/28891	1.44E-07	1.05E-05	6.71E-06	Lcn2/Anxa3/Anxa1/Jup/Bsg/Apoe/Rac1/Rtn4/Serpine1/Egf/Rab11a	11
GO:0008154	actin polymerization or depolymerization	10/158	197/28891	1.44E-07	1.05E-05	6.71E-06	Cfl1/Tmsb4x/Capg/Arpc2/Capzb/Gsn/Rac1/Dstn/Sptbn1/Tmsb10	10
GO:0007162	negative regulation of cell adhesion	12/158	315/28891	1.83E-07	1.30E-05	8.33E-06	Lgals3/Cfl1/Cyp1b1/Anxa1/Cldn7/Cd74/Cd24a/Serpine1/Mmp14/Col1a1/Adam10/Prdx2	12
GO:0030835	negative regulation of actin filament depolymerization	6/158	46/28891	1.90E-07	1.32E-05	8.49E-06	Cfl1/Capg/Arpc2/Capzb/Gsn/Sptbn1	6
GO:1903706	regulation of hemopoiesis	14/158	452/28891	2.13E-07	1.45E-05	9.32E-06	Actb/Anxa1/Tmem176b/Tsc22d1/Sox4/Ctr9/Rbp1/Tmem176a/Cd74/Cd24a/Eif6/Ctnnb1/Mmp14/Prdx2	14
GO:0046394	carboxylic acid biosynthetic process	12/158	321/28891	2.23E-07	1.49E-05	9.58E-06	Gsto1/Ptgs1/Acsm2/Anxa1/Pkm/Lpl/Rbp1/Cd74/Rdh10/Eif6/Mgst3/Ldhb	12
GO:0016053	organic acid biosynthetic process	12/158	324/28891	2.47E-07	1.62E-05	1.04E-05	Gsto1/Ptgs1/Acsm2/Anxa1/Pkm/Lpl/Rbp1/Cd74/Rdh10/Eif6/Mgst3/Ldhb	12
GO:0010634	positive regulation of epithelial cell migration	9/158	162/28891	2.88E-07	1.85E-05	1.19E-05	Lcn2/Anxa3/Anxa1/Bsg/Rac1/Rtn4/Serpine1/Egf/Rab11a	9
GO:0051016	barbed-end actin filament capping	5/158	27/28891	3.36E-07	2.12E-05	1.36E-05	Cfl1/Capg/Arpc2/Capzb/Gsn	5
GO:0030041	actin filament polymerization	9/158	170/28891	4.33E-07	2.68E-05	1.72E-05	Cfl1/Tmsb4x/Capg/Arpc2/Capzb/Gsn/Rac1/Sptbn1/Tmsb10	9
GO:0030100	regulation of endocytosis	12/158	344/28891	4.67E-07	2.83E-05	1.82E-05	Clu/Lgals3/Actb/Myh9/Rack1/Anxa2/Apoe/Serpine1/Egf/Cdc42/Lrp2/Arf1	12
GO:0051258	protein polymerization	11/158	282/28891	4.77E-07	2.84E-05	1.83E-05	Cfl1/Krt5/Tmsb4x/Capg/Arpc2/Capzb/Gsn/Map4/Rac1/Sptbn1/Tmsb10	11
GO:1903319	positive regulation of protein maturation	5/158	29/28891	4.90E-07	2.87E-05	1.84E-05	Myh9/Gsn/Sox4/Mmp14/S100a10	5
GO:0048041	focal adhesion assembly	7/158	87/28891	5.18E-07	2.98E-05	1.92E-05	Cfl1/Myh9/Actn1/Rac1/Mmp14/S100a10/Iqgap1	7
GO:0072330	monocarboxylic acid biosynthetic process	10/158	227/28891	5.33E-07	3.01E-05	1.93E-05	Ptgs1/Acsm2/Anxa1/Pkm/Lpl/Rbp1/Cd74/Rdh10/Eif6/Ldhb	10
GO:0001952	regulation of cell-matrix adhesion	8/158	128/28891	5.49E-07	3.01E-05	1.93E-05	Cfl1/Myh9/Jup/Rac1/Serpine1/Mmp14/S100a10/Iqgap1	8
GO:0051261	protein depolymerization	8/158	128/28891	5.49E-07	3.01E-05	1.93E-05	Cfl1/Capg/Arpc2/Capzb/Gsn/Dstn/Hspa8/Sptbn1	8
GO:0051894	positive regulation of focal adhesion assembly	5/158	30/28891	5.86E-07	3.15E-05	2.03E-05	Cfl1/Myh9/Rac1/S100a10/Iqgap1	5
GO:0043542	endothelial cell migration	10/158	231/28891	6.26E-07	3.31E-05	2.13E-05	Lcn2/Cyp1b1/Anxa3/Myh9/Anxa1/Jup/Bsg/Apoe/Rac1/Egf	10
GO:0014812	muscle cell migration	8/158	131/28891	6.56E-07	3.42E-05	2.20E-05	S100a11/Cyp1b1/Tmsb4x/Myh9/Anxa1/Arpc2/Serpine1/Iqgap1	8
GO:0043244	regulation of protein-containing complex disassembly	8/158	134/28891	7.79E-07	4.00E-05	2.57E-05	Cfl1/Capg/Arpc2/Capzb/Gsn/Dstn/Eif5a/Sptbn1	8
GO:0030595	leukocyte chemotaxis	10/158	240/28891	8.86E-07	4.48E-05	2.88E-05	Lgals3/Spp1/S100a14/Anxa1/Bsg/Cd74/Rac1/Serpine1/Slc12a2/Adam10	10
GO:1901879	regulation of protein depolymerization	7/158	95/28891	9.45E-07	4.68E-05	3.01E-05	Cfl1/Capg/Arpc2/Capzb/Gsn/Dstn/Sptbn1	7
GO:0031424	keratinization	6/158	60/28891	9.54E-07	4.68E-05	3.01E-05	Sprr1a/Sprr2f/Sfn/Krt7/Krt5/Cers3	6
GO:0000910	cytokinesis	9/158	188/28891	1.01E-06	4.87E-05	3.13E-05	Cfl1/Myh9/Calm1/Dstn/Cdc42/Sptbn1/Rab11a/Arf1/Iqgap1	9
GO:0007044	cell-substrate junction assembly	7/158	98/28891	1.17E-06	5.57E-05	3.58E-05	Cfl1/Myh9/Actn1/Rac1/Mmp14/S100a10/Iqgap1	7
GO:1901342	regulation of vasculature development	11/158	311/28891	1.25E-06	5.86E-05	3.76E-05	Lgals3/Cyp1b1/Anxa3/Pkm/Tnfrsf12a/Jup/Rtn4/Ctnnb1/Serpine1/Slc12a2/Adam10	11
GO:0150117	positive regulation of cell-substrate junction organization	5/158	35/28891	1.31E-06	6.05E-05	3.89E-05	Cfl1/Myh9/Rac1/S100a10/Iqgap1	5
GO:0051893	regulation of focal adhesion assembly	6/158	64/28891	1.40E-06	6.27E-05	4.03E-05	Cfl1/Myh9/Rac1/Mmp14/S100a10/Iqgap1	6
GO:0090109	regulation of cell-substrate junction assembly	6/158	64/28891	1.40E-06	6.27E-05	4.03E-05	Cfl1/Myh9/Rac1/Mmp14/S100a10/Iqgap1	6
GO:0097529	myeloid leukocyte migration	10/158	253/28891	1.43E-06	6.27E-05	4.03E-05	Lgals3/Spp1/S100a14/Anxa1/Bsg/Cd74/Rac1/Rtn4/Serpine1/Mmp14	10
GO:2001234	negative regulation of apoptotic signaling pathway	10/158	253/28891	1.43E-06	6.27E-05	4.03E-05	Clu/Lgals3/Rack1/Ier3/Cd74/Ctnnb1/Serpine1/Itga6/Prdx2/Pea15a	10

GO:0052547	regulation of peptidase activity	13/158	457/28891	1.52E-06	6.58E-05	4.22E-05	Wfdc2/Sfn/Cldn4/Rack1/Prss22/Gsn/Serpinb6b/Cstb/Serpinb6a/Timp3/Serpine1/Vcp/Ctsl	13
GO:0072593	reactive oxygen species metabolic process	10/158	257/28891	1.64E-06	7.03E-05	4.52E-05	Gpx3/Cdkn1a/Lcn2/Cyp1b1/Ier3/Ucp2/Eif6/Eif5a/Txn1/Prdx2	10
GO:0001954	positive regulation of cell-matrix adhesion	6/158	66/28891	1.69E-06	7.05E-05	4.53E-05	Cfl1/Myh9/Jup/Rac1/S100a10/Iqgap1	6
GO:0006979	response to oxidative stress	12/158	389/28891	1.69E-06	7.05E-05	4.53E-05	Gpx3/Cfl1/Ptgs1/Lcn2/Cyp1b1/Rack1/Anxa1/Apoe/Ucp2/Txn1/Prdx2/Idh1	12
GO:0022411	cellular component disassembly	13/158	462/28891	1.71E-06	7.05E-05	4.53E-05	Cfl1/Capg/Calm1/Arpc2/Capzb/Gsn/Map4/Cd24a/Dstn/Hspa8/Vcp/Eif5a/Sptbn1	13
GO:1903532	positive regulation of secretion by cell	12/158	390/28891	1.74E-06	7.07E-05	4.54E-05	Lgals3/Spp1/Myh9/Anxa2/Sox4/Bsg/Sdc1/Rac1/Rtn4/Slc12a2/S100a10/Arf1	12
GO:0014910	regulation of smooth muscle cell migration	7/158	105/28891	1.86E-06	7.39E-05	4.75E-05	S100a11/Cyp1b1/Tmsb4x/Myh9/Arpc2/Serpine1/Iqgap1	7
GO:0150115	cell-substrate junction organization	7/158	105/28891	1.86E-06	7.39E-05	4.75E-05	Cfl1/Myh9/Actn1/Rac1/Mmp14/S100a10/Iqgap1	7
GO:0045807	positive regulation of endocytosis	9/158	204/28891	1.98E-06	7.77E-05	4.99E-05	Clu/Myh9/Anxa2/Apoe/Serpine1/Egf/Cdc42/Lrp2/Arf1	9
GO:0032271	regulation of protein polymerization	9/158	206/28891	2.15E-06	8.33E-05	5.35E-05	Cfl1/Tmsb4x/Capg/Arpc2/Capzb/Gsn/Rac1/Sptbn1/Tmsb10	9
GO:0150116	regulation of cell-substrate junction organization	6/158	69/28891	2.19E-06	8.42E-05	5.41E-05	Cfl1/Myh9/Rac1/Mmp14/S100a10/Iqgap1	6
GO:0140694	non-membrane-bounded organelle assembly	12/158	402/28891	2.37E-06	9.01E-05	5.79E-05	Krt8/Krt19/Arpc2/Gsn/Flna/Tubb5/Rpsa/Rps5/Eif6/Rplp0/Rab11a/Rpl11	12
GO:1903729	regulation of plasma membrane organization	4/158	18/28891	2.48E-06	9.31E-05	5.98E-05	Myh9/Anxa2/Gsn/S100a10	4
GO:0007548	sex differentiation	11/158	335/28891	2.56E-06	9.40E-05	6.04E-05	Myh9/Flna/Srd5a1/Serpinb6b/Serpinb6a/Rac1/Rdh10/Ctnnb1/Serpine1/Nupr1/Lrp2	11
GO:0051098	regulation of binding	12/158	405/28891	2.56E-06	9.40E-05	6.04E-05	Cdkn1a/Actb/Rack1/Anxa2/Calm1/Apoe/Ctnnb1/Egf/Cdc42/Txn1/S100a10/Rpl11	12
GO:0034329	cell junction assembly	13/158	481/28891	2.66E-06	9.65E-05	6.20E-05	Cfl1/Cldn4/Actb/Myh9/Actn1/Cldn7/Jup/Rac1/Ctnnb1/Cdc42/Mmp14/S100a10/Iqgap1	13
GO:1905475	regulation of protein localization to membrane	9/158	212/28891	2.72E-06	9.75E-05	6.26E-05	Lgals3/Actb/Rack1/Arpc2/Gsn/Sptbn1/Mmp14/Adam10/Rab11a	9
GO:0010591	regulation of lamellipodium assembly	5/158	41/28891	2.93E-06	0.000103111	6.62E-05	Cfl1/Arpc2/Capzb/Rac1/Cdc42	5
GO:1900026	positive regulation of substrate adhesion-dependent cell spreading	5/158	41/28891	2.93E-06	0.000103111	6.62E-05	Arpc2/Flna/Rac1/Cdc42/S100a10	5
GO:0048771	tissue remodeling	9/158	215/28891	3.05E-06	0.000105287	6.76E-05	Slc34a1/Spp1/Klf6/Elf3/Flna/Cd24a/Rac1/Ctnnb1/Mmp14	9
GO:1903317	regulation of protein maturation	6/158	73/28891	3.06E-06	0.000105287	6.76E-05	Myh9/Gsn/Sox4/Serpine1/Mmp14/S100a10	6
GO:0048608	reproductive structure development	11/158	343/28891	3.20E-06	0.000109166	7.01E-05	Myh9/Anxa1/Flna/Serpinb6b/Serpinb6a/Rac1/Rdh10/Ctnnb1/Serpine1/Nupr1/Lrp2	11
GO:0032386	regulation of intracellular transport	11/158	346/28891	3.48E-06	0.000117439	7.54E-05	Sfn/Anxa2/Ywhae/Arpc2/Flna/Jup/Ier3/Cdc42/Txn1/Rab11a/Arf1	11
GO:0061458	reproductive system development	11/158	347/28891	3.58E-06	0.000119512	7.68E-05	Myh9/Anxa1/Flna/Serpinb6b/Serpinb6a/Rac1/Rdh10/Ctnnb1/Serpine1/Nupr1/Lrp2	11
GO:0014909	smooth muscle cell migration	7/158	116/28891	3.62E-06	0.000119768	7.69E-05	S100a11/Cyp1b1/Tmsb4x/Myh9/Arpc2/Serpine1/Iqgap1	7
GO:0042273	ribosomal large subunit biogenesis	6/158	76/28891	3.88E-06	0.000126862	8.15E-05	Rpl14/Eif6/Las1l/Rplp0/Rpl7/Rpl11	6
GO:0097530	granulocyte migration	8/158	167/28891	4.06E-06	0.000131685	8.46E-05	Lgals3/Spp1/S100a14/Anxa1/Bsg/Cd74/Rac1/Rtn4	8
GO:0006509	membrane protein ectodomain proteolysis	5/158	45/28891	4.70E-06	0.000150873	9.69E-05	Mmp7/Myh9/Apoe/Timp3/Adam10	5
GO:0006913	nucleocytoplasmic transport	11/158	359/28891	4.95E-06	0.00015579	0.000100074	Sfn/Cdkn1a/Cfl1/Ywhae/Flna/Jup/Ier3/Il33/Eif6/Txn1/Adam10	11
GO:0051169	nuclear transport	11/158	359/28891	4.95E-06	0.00015579	0.000100074	Sfn/Cdkn1a/Cfl1/Ywhae/Flna/Jup/Ier3/Il33/Eif6/Txn1/Adam10	11
GO:1903036	positive regulation of response to wounding	6/158	80/28891	5.23E-06	0.000163264	0.000104875	Cldn4/Anxa1/Flna/Cd24a/Serpine1/Prdx2	6
GO:0045216	cell-cell junction organization	9/158	231/28891	5.46E-06	0.000168264	0.000108087	Cldn4/Actb/Flna/Cldn7/Jup/Rac1/Ctnnb1/Cdc42/Adam10	9
GO:1902105	regulation of leukocyte differentiation	11/158	363/28891	5.49E-06	0.000168264	0.000108087	Actb/Anxa1/Tmem176b/Sox4/Rbp1/Tmem176a/Cd74/Cd24a/Ctnnb1/Mmp14/Prdx2	11
GO:0051047	positive regulation of secretion	12/158	437/28891	5.56E-06	0.000168849	0.000108462	Lgals3/Spp1/Myh9/Anxa2/Sox4/Bsg/Sdc1/Rac1/Rtn4/Slc12a2/S100a10/Arf1	12
GO:1901880	negative regulation of protein depolymerization	6/158	81/28891	5.63E-06	0.000169208	0.000108693	Cfl1/Capg/Arpc2/Capzb/Gsn/Sptbn1	6
GO:0002687	positive regulation of leukocyte migration	8/158	176/28891	5.99E-06	0.00017681	0.000113576	Lgals3/S100a14/Cd74/Rac1/Rtn4/Serpine1/Mmp14/Adam10	8
GO:0051494	negative regulation of cytoskeleton organization	8/158	176/28891	5.99E-06	0.00017681	0.000113576	Cfl1/Tmsb4x/Capg/Arpc2/Capzb/Gsn/Sptbn1/Tmsb10	8
GO:0010594	regulation of endothelial cell migration	8/158	177/28891	6.24E-06	0.000180409	0.000115888	Lcn2/Anxa3/Anxa1/Jup/Bsg/Apoe/Rac1/Egf	8
GO:2001235	positive regulation of apoptotic signaling pathway	8/158	177/28891	6.24E-06	0.000180409	0.000115888	Clu/Rack1/Tnfrsf12a/Timp3/Nupr1/Ctsl/Rpl11/Pea15a	8
GO:0048145	regulation of fibroblast proliferation	7/158	126/28891	6.27E-06	0.000180409	0.000115888	Cdkn1a/Anxa2/Cd74/Serpine1/Nupr1/Egf/Fth1	7
GO:0042176	regulation of protein catabolic process	11/158	369/28891	6.41E-06	0.000183011	0.00011756	Clu/Rack1/Anxa2/Flna/Ier3/Il33/Apoe/Timp3/Vcp/Nupr1/Rpl11	11
GO:1903707	negative regulation of hemopoiesis	7/158	129/28891	7.32E-06	0.000207096	0.000133031	Anxa1/Tmem176b/Tsc22d1/Tmem176a/Cd74/Ctnnb1/Prdx2	7
GO:0045765	regulation of angiogenesis	10/158	307/28891	7.93E-06	0.000221635	0.000142371	Lgals3/Cyp1b1/Anxa3/Pkm/Tnfrsf12a/Jup/Rtn4/Ctnnb1/Serpine1/Slc12a2	10
GO:1902905	positive regulation of supramolecular fiber organization	8/158	183/28891	7.97E-06	0.000221635	0.000142371	Cfl1/Arpc2/Gsn/Flna/Rac1/Dstn/Cdc42/S100a10	8
GO:1902743	regulation of lamellipodium organization	5/158	51/28891	8.80E-06	0.000242823	0.000155981	Cfl1/Arpc2/Capzb/Rac1/Cdc42	5
GO:0010959	regulation of metal ion transport	12/158	461/28891	9.52E-06	0.000260552	0.000167369	Gsto1/Lgals3/Lcn2/Ywhae/Calm1/Flna/Ctnnb1/Serpine1/Fxyd2/Egf/Arf1/Atp1b1	12
GO:0071621	granulocyte chemotaxis	7/158	135/28891	9.87E-06	0.000267673	0.000171944	Lgals3/Spp1/S100a14/Anxa1/Bsg/Cd74/Rac1	7
GO:0031346	positive regulation of cell projection organization	12/158	463/28891	9.94E-06	0.000267673	0.000171944	Cfl1/Lcn2/Arpc2/Flna/Tnfrsf12a/Apoe/Cd24a/Rac1/Cdc42/Fut9/Itga6/Iqgap1	12
GO:0010639	negative regulation of organelle organization	11/158	387/28891	1.00E-05	0.000267836	0.000172048	Cfl1/Tmsb4x/Capg/Arpc2/Capzb/Gsn/Map4/Ier3/Nupr1/Sptbn1/Tmsb10	11
GO:0062197	cellular response to chemical stress	10/158	316/28891	1.02E-05	0.000270132	0.000173523	Cfl1/Lcn2/Cyp1b1/Rack1/Anxa1/Serpinb6b/Serpinb6a/Micu1/Txn1/Prdx2	10
GO:0051651	maintenance of location in cell	9/158	250/28891	1.03E-05	0.000270988	0.000174073	Gsto1/Tmsb4x/Calm1/Gsn/Flna/Apoe/Fth1/Txn1/Tmsb10	9
GO:0043242	negative regulation of protein-containing complex disassembly	6/158	91/28891	1.10E-05	0.000288125	0.000185081	Cfl1/Capg/Arpc2/Capzb/Gsn/Sptbn1	6
GO:0032388	positive regulation of intracellular transport	8/158	192/28891	1.13E-05	0.000292583	0.000187945	Sfn/Anxa2/Ywhae/Arpc2/Flna/Jup/Cdc42/Arf1	8
GO:0042254	ribosome biogenesis	10/158	322/28891	1.20E-05	0.000308138	0.000197937	Rpl14/Rpsa/Rps5/Eif6/Las1l/Rplp0/Rps9/Rpl7/Rps13/Rpl11	10
GO:0060562	epithelial tube morphogenesis	11/158	395/28891	1.21E-05	0.000309254	0.000198654	Cfl1/Lcn2/Bsg/Mgp/Clic4/Rdh10/Ctnnb1/Slc12a2/Egf/Mmp14/Lrp2	11
GO:0010721	negative regulation of cell development	10/158	323/28891	1.23E-05	0.000311712	0.000200232	Cfl1/Anxa1/Tmem176b/Tsc22d1/Tmem176a/Cd74/Cd24a/Rtn4/Ctnnb1/Prdx2	10
GO:0000302	response to reactive oxygen species	8/158	195/28891	1.27E-05	0.000315216	0.000202484	Cfl1/Lcn2/Cyp1b1/Rack1/Anxa1/Ucp2/Txn1/Prdx2	8
GO:0051495	positive regulation of cytoskeleton organization	8/158	195/28891	1.27E-05	0.000315216	0.000202484	Cfl1/Arpc2/Gsn/Flna/Rac1/Dstn/Cdc42/S100a10	8
GO:1903035	negative regulation of response to wounding	6/158	94/28891	1.33E-05	0.000326833	0.000209946	Anxa2/Il33/Apoe/Cd24a/Serpine1/Slc12a2	6
GO:2000146	negative regulation of cell motility	10/158	326/28891	1.34E-05	0.000326833	0.000209946	Cfl1/Cyp1b1/Myh9/Jup/Clic4/Il33/Apoe/Cd74/Rac1/Serpine1	10
GO:0051099	positive regulation of binding	8/158	197/28891	1.36E-05	0.000326833	0.000209946	Anxa2/Calm1/Apoe/Ctnnb1/Egf/Txn1/S100a10/Rpl11	8
GO:0008406	gonad development	9/158	259/28891	1.37E-05	0.000326833	0.000209946	Myh9/Flna/Serpinb6b/Serpinb6a/Rac1/Rdh10/Serpine1/Nupr1/Lrp2	9
GO:0010954	positive regulation of protein processing	4/158	27/28891	1.37E-05	0.000326833	0.000209946	Myh9/Gsn/Mmp14/S100a10	4
GO:0097193	intrinsic apoptotic signaling pathway	10/158	327/28891	1.37E-05	0.000326833	0.000209946	Clu/Sfn/Cdkn1a/Cyp1b1/Rack1/Ier3/Cd74/Ctnnb1/Nupr1/Rpl11	10
GO:0045104	intermediate filament cytoskeleton organization	6/158	96/28891	1.50E-05	0.000355692	0.000228483	Krt19/Krt7/Krt15/Krt18/Krt5/Krt14	6
GO:0060326	cell chemotaxis	10/158	331/28891	1.52E-05	0.000356687	0.000229123	Lgals3/Spp1/S100a14/Anxa1/Bsg/Cd74/Rac1/Serpine1/Slc12a2/Adam10	10
GO:0070509	calcium ion import	5/158	57/28891	1.53E-05	0.000356687	0.000229123	Lgals3/Ctnnb1/Serpine1/Egf/Micu1	5
GO:0045137	development of primary sexual characteristics	9/158	263/28891	1.54E-05	0.00035773	0.000229793	Myh9/Flna/Serpinb6b/Serpinb6a/Rac1/Rdh10/Serpine1/Nupr1/Lrp2	9
GO:0034614	cellular response to reactive oxygen species	7/158	145/28891	1.57E-05	0.000361848	0.000232438	Cfl1/Lcn2/Cyp1b1/Rack1/Anxa1/Txn1/Prdx2	7
GO:0045103	intermediate filament-based process	6/158	97/28891	1.59E-05	0.000364518	0.000234153	Krt19/Krt7/Krt15/Krt18/Krt5/Krt14	6
GO:0033619	membrane protein proteolysis	5/158	59/28891	1.81E-05	0.000408461	0.00026238	Mmp7/Myh9/Apoe/Timp3/Adam10	5
GO:1900024	regulation of substrate adhesion-dependent cell spreading	5/158	59/28891	1.81E-05	0.000408461	0.00026238	Arpc2/Flna/Rac1/Cdc42/S100a10	5
GO:0048144	fibroblast proliferation	7/158	150/28891	1.96E-05	0.000438572	0.000281723	Cdkn1a/Anxa2/Cd74/Serpine1/Nupr1/Egf/Fth1	7
GO:0001778	plasma membrane repair	4/158	30/28891	2.11E-05	0.000466899	0.000299919	Myh9/Myof/Anxa2/S100a10	4
GO:0010592	positive regulation of lamellipodium assembly	4/158	30/28891	2.11E-05	0.000466899	0.000299919	Cfl1/Arpc2/Rac1/Cdc42	4
GO:0070301	cellular response to hydrogen peroxide	5/158	62/28891	2.31E-05	0.000506841	0.000325576	Cfl1/Lcn2/Cyp1b1/Anxa1/Txn1	5
GO:0043270	positive regulation of monoatomic ion transport	9/158	277/28891	2.32E-05	0.000506841	0.000325576	Gsto1/Lgals3/Cfl1/Lcn2/Calm1/Flna/Serpine1/Arf1/Atp1b1	9
GO:1904375	regulation of protein localization to cell periphery	7/158	156/28891	2.52E-05	0.000546364	0.000350965	Lgals3/Actb/Rack1/Sptbn1/Mmp14/Adam10/Rab11a	7

GO:0006066	alcohol metabolic process	10/158	354/28891	2.70E-05	0.000581836	0.00037375	Cyp1b1/Myh9/Myof/Rbp1/Miox/Fmo5/Apoe/Rdh10/Lrp2/Idh1	10
GO:0040013	negative regulation of locomotion	10/158	358/28891	2.97E-05	0.000635855	0.00040845	Cfl1/Cyp1b1/Myh9/Jup/Clic4/Il33/Apoe/Cd74/Rac1/Serpine1	10
GO:0042255	ribosome assembly	5/158	66/28891	3.13E-05	0.000666508	0.000428141	Rpsa/Rps5/Eif6/Rplp0/Rpl11	5
GO:0010595	positive regulation of endothelial cell migration	6/158	110/28891	3.26E-05	0.000688682	0.000442385	Lcn2/Anxa3/Anxa1/Bsg/Rac1/Egf	6
GO:0042989	sequestering of actin monomers	3/158	12/28891	3.40E-05	0.000714834	0.000459183	Tmsb4x/Gsn/Tmsb10	3
GO:0014911	positive regulation of smooth muscle cell migration	5/158	68/28891	3.62E-05	0.000756168	0.000485735	S100a11/Cyp1b1/Tmsb4x/Arpc2/Iqgap1	5
GO:0008630	intrinsic apoptotic signaling pathway in response to DNA damage	6/158	113/28891	3.79E-05	0.000786581	0.000505271	Clu/Sfn/Cdkn1a/Ier3/Cd74/Nupr1	6
GO:0070613	regulation of protein processing	5/158	69/28891	3.89E-05	0.000801647	0.000514949	Myh9/Gsn/Serpine1/Mmp14/S100a10	5
GO:0090279	regulation of calcium ion import	4/158	35/28891	3.95E-05	0.000808985	0.000519663	Lgals3/Ctnnb1/Serpine1/Egf	4
GO:2000377	regulation of reactive oxygen species metabolic process	7/158	168/28891	4.05E-05	0.000824186	0.000529428	Cdkn1a/Lcn2/Cyp1b1/Ier3/Eif6/Eif5a/Prdx2	7
GO:0051235	maintenance of location	10/158	373/28891	4.20E-05	0.000840521	0.00053992	Gsto1/Tmsb4x/Calm1/Gsn/Flna/Lpl/Apoe/Fth1/Txn1/Tmsb10	10
GO:0007009	plasma membrane organization	7/158	169/28891	4.21E-05	0.000840521	0.00053992	Clu/Myh9/Myof/Anxa2/Gsn/Sptbn1/S100a10	7
GO:0035725	sodium ion transmembrane transport	7/158	169/28891	4.21E-05	0.000840521	0.00053992	Slc34a1/Slc12a1/Fxyd2/Slc12a2/Slc6a19/Arf1/Atp1b1	7
GO:1903050	regulation of proteolysis involved in protein catabolic process	8/158	232/28891	4.39E-05	0.000870786	0.000559362	Clu/Rack1/Il33/Apoe/Vcp/Nupr1/Egf/Rpl11	8
GO:0017038	protein import	3/158	13/28891	4.41E-05	0.000870786	0.000559362	Clu/Apoe/Lrp2	3
GO:0034250	positive regulation of amide metabolic process	7/158	171/28891	4.53E-05	0.000884568	0.000568215	Clu/Cyp1b1/Pkm/Sox4/Apoe/Eif6/Eif5a	7
GO:2001236	regulation of extrinsic apoptotic signaling pathway	7/158	171/28891	4.53E-05	0.000884568	0.000568215	Lgals3/Tnfrsf12a/Timp3/Serpine1/Itga6/Prdx2/Pea15a	7
GO:0097242	amyloid-beta clearance	4/158	37/28891	4.94E-05	0.000953297	0.000612363	Clu/Apoe/Lrp2/Rab11a	4
GO:1902745	positive regulation of lamellipodium organization	4/158	37/28891	4.94E-05	0.000953297	0.000612363	Cfl1/Arpc2/Rac1/Cdc42	4
GO:0051017	actin filament bundle assembly	7/158	174/28891	5.06E-05	0.000968824	0.000622337	Cfl1/Myh9/Actn1/Flna/Rac1/Cdc42/S100a10	7
GO:0006935	chemotaxis	11/158	462/28891	5.08E-05	0.000968824	0.000622337	Lgals3/Spp1/S100a14/Anxa1/Bsg/Cd74/Rac1/Serpine1/Slc12a2/Lrp2/Adam10	11
GO:0030032	lamellipodium assembly	5/158	73/28891	5.11E-05	0.000968998	0.000622449	Cfl1/Arpc2/Capzb/Rac1/Cdc42	5
GO:0042330	taxis	11/158	464/28891	5.28E-05	0.000995687	0.000639593	Lgals3/Spp1/S100a14/Anxa1/Bsg/Cd74/Rac1/Serpine1/Slc12a2/Lrp2/Adam10	11
GO:1903076	regulation of protein localization to plasma membrane	6/158	120/28891	5.32E-05	0.000996783	0.000640298	Lgals3/Actb/Rack1/Sptbn1/Mmp14/Rab11a	6
GO:0007163	establishment or maintenance of cell polarity	8/158	239/28891	5.41E-05	0.001008567	0.000647867	Cfl1/Actb/Myh9/Gsn/Map4/Rac1/Rab10/Cdc42	8
GO:0099024	plasma membrane invagination	5/158	74/28891	5.46E-05	0.001011667	0.000649858	Myh9/Gsn/Rac1/Cdc42/Arf1	5
GO:0061572	actin filament bundle organization	7/158	177/28891	5.64E-05	0.001039662	0.000667841	Cfl1/Myh9/Actn1/Flna/Rac1/Cdc42/S100a10	7
GO:0042692	muscle cell differentiation	11/158	468/28891	5.70E-05	0.001045634	0.000671677	Krt8/Krt19/Ccnd2/Tmsb4x/Myh9/Myof/Actn1/Ctnnb1/Uchl1/Eif5a/Mmp14	11
GO:0062012	regulation of small molecule metabolic process	10/158	389/28891	5.96E-05	0.001087837	0.000698787	Myh9/Anxa1/Ier3/Fmo5/Apoe/Cd74/Rdh10/Eif6/Vcp/Nupr1	10
GO:0030336	negative regulation of cell migration	9/158	313/28891	6.00E-05	0.001088021	0.000698905	Cyp1b1/Myh9/Jup/Clic4/Il33/Apoe/Cd74/Rac1/Serpine1	9
GO:1902106	negative regulation of leukocyte differentiation	6/158	123/28891	6.10E-05	0.001100817	0.000707125	Anxa1/Tmem176b/Tmem176a/Cd74/Ctnnb1/Prdx2	6
GO:0045109	intermediate filament organization	5/158	76/28891	6.20E-05	0.001113014	0.00071496	Krt19/Krt7/Krt15/Krt5/Krt14	5
GO:0031647	regulation of protein stability	9/158	316/28891	6.45E-05	0.001145625	0.000735908	Clu/Gsn/Flna/Sox4/Cd74/Rtn4/Hspa8/Atp1b1/Rpl11	9
GO:0060485	mesenchyme development	9/158	316/28891	6.45E-05	0.001145625	0.000735908	Cfl1/Flna/Sox4/Rdh10/Timp3/Rtn4/Ctnnb1/Cdc42/Col1a1	9
GO:0042982	amyloid precursor protein metabolic process	5/158	77/28891	6.60E-05	0.001160141	0.000745233	Clu/Itm2b/Apoe/Rtn4/Adam10	5
GO:0046847	filopodium assembly	5/158	77/28891	6.60E-05	0.001160141	0.000745233	Arpc2/Capzb/Rac1/Cdc42/Itga6	5
GO:0045766	positive regulation of angiogenesis	7/158	182/28891	6.72E-05	0.001168246	0.000750439	Lgals3/Cyp1b1/Anxa3/Pkm/Jup/Rtn4/Serpine1	7
GO:1904018	positive regulation of vasculature development	7/158	182/28891	6.72E-05	0.001168246	0.000750439	Lgals3/Cyp1b1/Anxa3/Pkm/Jup/Rtn4/Serpine1	7
GO:0030099	myeloid cell differentiation	11/158	480/28891	7.14E-05	0.00123551	0.000793647	Myh9/Actn1/Flna/Ctr9/Rbp1/Il33/Ucp2/Cd74/Eif6/Ctnnb1/Junb	11
GO:0001763	morphogenesis of a branching structure	8/158	249/28891	7.20E-05	0.0012397	0.000796339	Mgp/Clic4/Rdh10/Rtn4/Ctnnb1/Slc12a2/Egf/Mmp14	8
GO:0048146	positive regulation of fibroblast proliferation	5/158	79/28891	7.47E-05	0.001278153	0.00082104	Cdkn1a/Anxa2/Cd74/Serpine1/Egf	5
GO:0045927	positive regulation of growth	9/158	324/28891	7.81E-05	0.001327014	0.000852426	Sfn/Cfl1/Ccnd2/Tnfrsf12a/Apoe/Serpine1/Cdc42/Mmp14/Adam10	9
GO:0099173	postsynapse organization	8/158	252/28891	7.83E-05	0.001327014	0.000852426	Cfl1/Actb/Apoe/Rac1/Hspa8/Cdc42/Adam10/Arf1	8
GO:0055078	sodium ion homeostasis	4/158	42/28891	8.20E-05	0.001381925	0.000887698	Spp1/Slc12a1/Slc12a2/Atp1b1	4
GO:0048762	mesenchymal cell differentiation	8/158	254/28891	8.27E-05	0.001387892	0.000891532	Cfl1/Flna/Rdh10/Timp3/Rtn4/Ctnnb1/Cdc42/Col1a1	8
GO:0046496	nicotinamide nucleotide metabolic process	5/158	81/28891	8.41E-05	0.001404348	0.000902102	Taldo1/Fmo5/Vcp/Ldhb/Idh1	5
GO:0009612	response to mechanical stimulus	7/158	189/28891	8.51E-05	0.001414019	0.000908315	Krt5/Gsn/Jup/Rac1/Ctnnb1/Slc12a2/Col1a1	7
GO:0034765	regulation of monoatomic ion transmembrane transport	11/158	490/28891	8.57E-05	0.001416922	0.00091018	Gsto1/Cfl1/Lcn2/Ywhae/Calm1/Flna/Clic4/Clic1/Fxyd2/Arf1/Atp1b1	11
GO:0010324	membrane invagination	5/158	82/28891	8.92E-05	0.001466984	0.000942338	Myh9/Gsn/Rac1/Cdc42/Arf1	5
GO:0048754	branching morphogenesis of an epithelial tube	7/158	191/28891	9.09E-05	0.001487964	0.000955814	Mgp/Clic4/Rdh10/Ctnnb1/Slc12a2/Egf/Mmp14	7
GO:0050708	regulation of protein secretion	9/158	331/28891	9.19E-05	0.001495868	0.000960892	Myh9/Anxa1/Sox4/Bsg/Apoe/Ucp2/Rac1/Slc12a2/Arf1	9
GO:1903052	positive regulation of proteolysis involved in protein catabolic process	6/158	133/28891	9.41E-05	0.001516825	0.000974354	Clu/Rack1/Il33/Vcp/Nupr1/Egf	6
GO:0019362	pyridine nucleotide metabolic process	5/158	83/28891	9.45E-05	0.001516825	0.000974354	Taldo1/Fmo5/Vcp/Ldhb/Idh1	5
GO:0048844	artery morphogenesis	5/158	83/28891	9.45E-05	0.001516825	0.000974354	Sox4/Apoe/Rtn4/Ctnnb1/Lrp2	5
GO:0060996	dendritic spine development	6/158	134/28891	9.81E-05	0.001566761	0.001006431	Cfl1/Apoe/Rac1/Cdc42/Adam10/Arf1	6
GO:0034504	protein localization to nucleus	9/158	335/28891	0.000100615	0.001599292	0.001027327	Cdkn1a/Cfl1/Ywhae/Flna/Jup/Il33/Txn1/Col1a1/Rpl11	9
GO:0046822	regulation of nucleocytoplasmic transport	6/158	135/28891	0.000102202	0.001616833	0.001038595	Sfn/Ywhae/Flna/Jup/Ier3/Txn1	6
GO:0061136	regulation of proteasomal protein catabolic process	7/158	195/28891	0.00010348	0.001629315	0.001046613	Clu/Rack1/Il33/Apoe/Vcp/Nupr1/Rpl11	7
GO:0009636	response to toxic substance	6/158	136/28891	0.000106442	0.001668084	0.001071517	Gpx3/Lcn2/Cyp1b1/Nupr1/Txn1/Prdx2	6
GO:0006090	pyruvate metabolic process	6/158	137/28891	0.000110819	0.001728573	0.001110373	Pkm/Ier3/Ucp2/Eif6/Nupr1/Ldhb	6
GO:0071214	cellular response to abiotic stimulus	9/158	340/28891	0.000112521	0.001738874	0.00111699	Cdkn1a/Ccnd2/Actb/Serpinb6b/Serpinb6a/Rac1/Ctnnb1/Micu1/Col1a1	9
GO:0104004	cellular response to environmental stimulus	9/158	340/28891	0.000112521	0.001738874	0.00111699	Cdkn1a/Ccnd2/Actb/Serpinb6b/Serpinb6a/Rac1/Ctnnb1/Micu1/Col1a1	9
GO:1990266	neutrophil migration	6/158	138/28891	0.000115339	0.001774197	0.00113968	Lgals3/Spp1/Bsg/Cd74/Rac1/Rtn4	6
GO:0071674	mononuclear cell migration	7/158	199/28891	0.000117396	0.001790851	0.001150378	Lgals3/S100a14/Anxa1/Serpine1/Slc12a2/Cdc42/Adam10	7
GO:0044788	modulation by host of viral process	4/158	46/28891	0.000117494	0.001790851	0.001150378	Cfl1/Apoe/Hspa8/Cdc42	4
GO:0071900	regulation of protein serine/threonine kinase activity	9/158	343/28891	0.000120216	0.001824012	0.001171679	Sfn/Cdkn1a/Ccnd2/Actb/Apoe/Cd24a/Uchl1/Egf/Iqgap1	9
GO:0070661	leukocyte proliferation	10/158	425/28891	0.000123494	0.001865269	0.001198181	Clu/Lgals3/Cdkn1a/Anxa1/Il33/Cd74/Cd24a/Ctnnb1/Junb/Prdx2	10
GO:0090150	establishment of protein localization to membrane	8/158	270/28891	0.000126177	0.001897199	0.001218692	Krt18/Rack1/Arpc2/Cd24a/Slc12a1/Rab10/Sptbn1/Rab11a	8
GO:0009410	response to xenobiotic stimulus	9/158	346/28891	0.000128347	0.001919376	0.001232937	Gsto1/Lcn2/Gsta4/Cyp1b1/Mmp7/Srd5a1/Fmo5/Serpine1/Uchl1	9
GO:0050821	protein stabilization	7/158	202/28891	0.000128802	0.001919376	0.001232937	Clu/Flna/Sox4/Cd74/Rtn4/Atp1b1/Rpl11	7
GO:0006898	receptor-mediated endocytosis	8/158	272/28891	0.000132732	0.00196915	0.001264911	Clu/Anxa2/Sdc1/Apoe/Serpine1/Egf/Lrp2/Arf1	8
GO:0003159	morphogenesis of an endothelium	3/158	19/28891	0.000145812	0.002134745	0.001371283	Lcn2/Bsg/Ctnnb1	3
GO:0061154	endothelial tube morphogenesis	3/158	19/28891	0.000145812	0.002134745	0.001371283	Lcn2/Bsg/Ctnnb1	3
GO:1902430	negative regulation of amyloid-beta formation	3/158	19/28891	0.000145812	0.002134745	0.001371283	Clu/Apoe/Rtn4	3
GO:0055074	calcium ion homeostasis	9/158	353/28891	0.000149124	0.002173696	0.001396304	Slc34a1/Gsto1/Spp1/Ywhae/Calm1/Flna/Apoe/Micu1/Atp1b1	9
GO:0034205	amyloid-beta formation	4/158	49/28891	0.000150622	0.002185977	0.001404193	Clu/Apoe/Rtn4/Adam10	4
GO:0042542	response to hydrogen peroxide	5/158	92/28891	0.000153893	0.002201284	0.001414025	Cfl1/Lcn2/Cyp1b1/Anxa1/Txn1	5
GO:0072524	pyridine-containing compound metabolic process	5/158	92/28891	0.000153893	0.002201284	0.001414025	Taldo1/Fmo5/Vcp/Ldhb/Idh1	5

GO:0097581	lamellipodium organization	5/158	92/28891	0.000153893	0.002201284	0.001414025	Cfl1/Arpc2/Capzb/Rac1/Cdc42	5
GO:0045732	positive regulation of protein catabolic process	7/158	208/28891	0.000154314	0.002201284	0.001414025	Clu/Rack1/Ier3/Il33/Apoe/Vcp/Nupr1	7
GO:0034767	positive regulation of monoatomic ion transmembrane transport	7/158	209/28891	0.000158939	0.002257603	0.001450203	Gsto1/Cfl1/Lcn2/Calm1/Flna/Arf1/Atp1b1	7
GO:0022613	ribonucleoprotein complex biogenesis	10/158	440/28891	0.000163548	0.002305256	0.001480813	Rpl14/Rpsa/Rps5/Eif6/Las1l/Rplp0/Rps9/Rpl7/Rps13/Rpl11	10
GO:0030307	positive regulation of cell growth	7/158	210/28891	0.000163675	0.002305256	0.001480813	Sfn/Cfl1/Tnfrsf12a/Apoe/Cdc42/Mmp14/Adam10	7
GO:0034764	positive regulation of transmembrane transport	8/158	282/28891	0.000169872	0.002382489	0.001530425	Slc34a1/Gsto1/Cfl1/Lcn2/Calm1/Flna/Arf1/Atp1b1	8
GO:0120032	regulation of plasma membrane bounded cell projection assembly	7/158	212/28891	0.00017349	0.002423046	0.001556477	Cfl1/Arpc2/Capzb/Map4/Rac1/Cdc42/Rab11a	7
GO:1902041	regulation of extrinsic apoptotic signaling pathway via death domain receptors	4/158	51/28891	0.000176151	0.002449968	0.001573771	Lgals3/Timp3/Serpine1/Pea15a	4
GO:0090316	positive regulation of intracellular protein transport	6/158	150/28891	0.000181884	0.002519202	0.001618245	Sfn/Ywhae/Arpc2/Flna/Jup/Cdc42	6
GO:0031334	positive regulation of protein-containing complex assembly	7/158	214/28891	0.000183775	0.002534886	0.001628319	Clu/Lgals3/Rack1/Arpc2/Cd24a/Rac1/Vcp	7
GO:0060491	regulation of cell projection assembly	7/158	215/28891	0.0001891	0.002597598	0.001668603	Cfl1/Arpc2/Capzb/Map4/Rac1/Cdc42/Rab11a	7
GO:0072594	establishment of protein localization to organelle	10/158	452/28891	0.000203011	0.002777261	0.001784012	Clu/Cdkn1a/Cfl1/Flna/Jup/Il33/Hspa8/Rab10/Lrp2/Rab11a	10
GO:0046456	icosanoid biosynthetic process	4/158	53/28891	0.000204664	0.002788447	0.001791197	Ptgs1/Anxa1/Cd74/Mgst3	4
GO:0031032	actomyosin structure organization	7/158	218/28891	0.000205827	0.00279289	0.001794052	Krt8/Krt19/Myh9/Rac1/Cdc42/S100a10/Iqgap1	7
GO:0006970	response to osmotic stress	5/158	98/28891	0.000207001	0.002797453	0.001796983	Serpinb6b/Serpinb6a/Rac1/Slc12a2/Micu1	5
GO:0060560	developmental growth involved in morphogenesis	8/158	292/28891	0.000215176	0.002896196	0.001860412	Cfl1/Tnfrsf12a/Apoe/Rdh10/Rtn4/Ctnnb1/Slc12a2/Iqgap1	8
GO:0015698	inorganic anion transport	6/158	155/28891	0.000217249	0.002912357	0.001870793	Slc34a1/Cldn4/Clic4/Clic1/Slc12a1/Slc12a2	6
GO:0044794	positive regulation by host of viral process	3/158	22/28891	0.00022896	0.003034119	0.001949009	Cfl1/Apoe/Hspa8	3
GO:1902992	negative regulation of amyloid precursor protein catabolic process	3/158	22/28891	0.00022896	0.003034119	0.001949009	Clu/Apoe/Rtn4	3
GO:0001558	regulation of cell growth	10/158	459/28891	0.000229525	0.003034119	0.001949009	Sfn/Cdkn1a/Cfl1/Rack1/Tnfrsf12a/Apoe/Rtn4/Cdc42/Mmp14/Adam10	10
GO:0008361	regulation of cell size	7/158	222/28891	0.000229968	0.003034119	0.001949009	Cfl1/Tnfrsf12a/Apoe/Rac1/Slc12a1/Rtn4/Slc12a2	7
GO:0016055	Wnt signaling pathway	10/158	460/28891	0.00023354	0.003069125	0.001971495	Sox4/Ctr9/Jup/Sdc1/Apoe/Rac1/Ctnnb1/Vcp/Egf/Col1a1	10
GO:0051924	regulation of calcium ion transport	8/158	296/28891	0.000235873	0.003087627	0.00198338	Gsto1/Lgals3/Ywhae/Calm1/Ctnnb1/Serpine1/Egf/Atp1b1	8
GO:0198738	cell-cell signaling by wnt	10/158	462/28891	0.000241747	0.003152159	0.002024833	Sox4/Ctr9/Jup/Sdc1/Apoe/Rac1/Ctnnb1/Vcp/Egf/Col1a1	10
GO:0045619	regulation of lymphocyte differentiation	7/158	224/28891	0.000242866	0.003154424	0.002026288	Actb/Anxa1/Sox4/Cd74/Cd24a/Mmp14/Prdx2	7
GO:0034446	substrate adhesion-dependent cell spreading	5/158	102/28891	0.000249474	0.003215235	0.002065351	Arpc2/Flna/Rac1/Cdc42/S100a10	5
GO:2001237	negative regulation of extrinsic apoptotic signaling pathway	5/158	102/28891	0.000249474	0.003215235	0.002065351	Lgals3/Serpine1/Itga6/Prdx2/Pea15a	5
GO:0098974	postsynaptic actin cytoskeleton organization	3/158	23/28891	0.000262249	0.003353979	0.002154475	Actb/Rac1/Arf1	3
GO:0099188	postsynaptic cytoskeleton organization	3/158	23/28891	0.000262249	0.003353979	0.002154475	Actb/Rac1/Arf1	3
GO:0006633	fatty acid biosynthetic process	6/158	161/28891	0.000266609	0.003396716	0.002181927	Ptgs1/Acsm2/Anxa1/Lpl/Cd74/Eif6	6
GO:0043393	regulation of protein binding	7/158	228/28891	0.000270412	0.003413556	0.002192745	Cdkn1a/Actb/Rack1/Anxa2/Apoe/Cdc42/Rpl11	7
GO:0050435	amyloid-beta metabolic process	4/158	57/28891	0.000271441	0.003413556	0.002192745	Clu/Apoe/Rtn4/Adam10	4
GO:0006816	calcium ion transport	10/158	469/28891	0.000272404	0.003413556	0.002192745	Gsto1/Lgals3/Ywhae/Calm1/Flna/Ctnnb1/Serpine1/Egf/Micu1/Atp1b1	10
GO:0006096	glycolytic process	5/158	104/28891	0.000273044	0.003413556	0.002192745	Pkm/Ier3/Ucp2/Eif6/Nupr1	5
GO:0045185	maintenance of protein location	5/158	104/28891	0.000273044	0.003413556	0.002192745	Tmsb4x/Gsn/Flna/Txn1/Tmsb10	5
GO:0061138	morphogenesis of a branching epithelium	7/158	229/28891	0.000277677	0.003458524	0.002221631	Mgp/Clic4/Rdh10/Ctnnb1/Slc12a2/Egf/Mmp14	7
GO:0060070	canonical Wnt signaling pathway	8/158	305/28891	0.000288454	0.003536331	0.002271611	Sox4/Jup/Sdc1/Apoe/Ctnnb1/Vcp/Egf/Col1a1	8
GO:0006692	prostanoid metabolic process	4/158	58/28891	0.000290314	0.003536331	0.002271611	Ptgs1/Anxa1/Cd74/Mgst3	4
GO:0034332	adherens junction organization	4/158	58/28891	0.000290314	0.003536331	0.002271611	Actb/Ctnnb1/Cdc42/Adam10	4
GO:0043388	positive regulation of DNA binding	4/158	58/28891	0.000290314	0.003536331	0.002271611	Calm1/Ctnnb1/Egf/Txn1	4
GO:0045010	actin nucleation	4/158	58/28891	0.000290314	0.003536331	0.002271611	Arpc2/Gsn/Arpc1b/Arf1	4
GO:0051489	regulation of filopodium assembly	4/158	58/28891	0.000290314	0.003536331	0.002271611	Arpc2/Capzb/Rac1/Cdc42	4
GO:0031400	negative regulation of protein modification process	10/158	473/28891	0.000291339	0.003536331	0.002271611	Cdkn1a/Actb/Rack1/Ywhae/Sox4/Apoe/Ctnnb1/Cadm4/Uchl1/Rpl11	10
GO:0006706	steroid catabolic process	3/158	24/28891	0.000298513	0.003597248	0.002310742	Spp1/Srd5a1/Apoe	3
GO:0097284	hepatocyte apoptotic process	3/158	24/28891	0.000298513	0.003597248	0.002310742	Krt8/Krt18/Gsn	3
GO:0033157	regulation of intracellular protein transport	7/158	232/28891	0.000300418	0.003607171	0.002317116	Sfn/Ywhae/Arpc2/Flna/Jup/Cdc42/Txn1	7
GO:0050807	regulation of synapse organization	8/158	309/28891	0.000314714	0.003765292	0.002418688	Cfl1/Tubb5/Apoe/Ctnnb1/Hspa8/Vcp/Cdc42/Adam10	8
GO:0043523	regulation of neuron apoptotic process	8/158	310/28891	0.000321576	0.003833641	0.002462593	Clu/Lcn2/Apoe/Ucp2/Ctnnb1/Nupr1/Cdc42/Prdx2	8
GO:0031099	regeneration	6/158	167/28891	0.000324383	0.003835936	0.002464066	Cdkn1a/Anxa1/Flna/Pkm/Rtn4/Serpine1	6
GO:0097061	dendritic spine organization	5/158	108/28891	0.000325216	0.003835936	0.002464066	Cfl1/Apoe/Cdc42/Adam10/Arf1	5
GO:2000060	positive regulation of ubiquitin-dependent protein catabolic process	5/158	108/28891	0.000325216	0.003835936	0.002464066	Clu/Rack1/Il33/Vcp/Egf	5
GO:0006893	Golgi to plasma membrane transport	4/158	60/28891	0.000330857	0.003888733	0.002497982	Krt18/Rack1/Rab10/Sptbn1	4
GO:0055064	chloride ion homeostasis	3/158	25/28891	0.000337862	0.003957131	0.002541918	Spp1/Slc12a1/Slc12a2	3
GO:0006887	exocytosis	9/158	395/28891	0.000341646	0.00398746	0.0025614	Anxa3/Myh9/Anxa2/Anxa1/Sdc1/Rab10/Rab11a/S100a10/Arf1	9
GO:0043434	response to peptide hormone	9/158	396/28891	0.000347983	0.004047273	0.002599822	Cfl1/Anxa1/Pkm/Lpl/Ywhag/Rac1/Eif6/Ctnnb1/Rab10	9
GO:0042987	amyloid precursor protein catabolic process	4/158	61/28891	0.000352578	0.004074322	0.002617197	Clu/Apoe/Rtn4/Adam10	4
GO:0006821	chloride transport	5/158	110/28891	0.00035397	0.004074322	0.002617197	Cldn4/Clic4/Clic1/Slc12a1/Slc12a2	5
GO:0030593	neutrophil chemotaxis	5/158	110/28891	0.00035397	0.004074322	0.002617197	Lgals3/Spp1/Bsg/Cd74/Rac1	5
GO:0030010	establishment of cell polarity	6/158	170/28891	0.000356727	0.004091937	0.002628513	Cfl1/Myh9/Gsn/Map4/Rab10/Cdc42	6
GO:0030098	lymphocyte differentiation	10/158	486/28891	0.000360661	0.004122901	0.002648403	Actb/Anxa1/Sox4/Cd74/Cd24a/Ly6d/Ctnnb1/Mmp14/Prdx2/Ctsl	10
GO:0002690	positive regulation of leukocyte chemotaxis	5/158	111/28891	0.000369049	0.004204387	0.002700746	S100a14/Cd74/Rac1/Serpine1/Adam10	5
GO:0050803	regulation of synapse structure or activity	8/158	317/28891	0.000373104	0.004236123	0.002721133	Cfl1/Tubb5/Apoe/Ctnnb1/Hspa8/Vcp/Cdc42/Adam10	8
GO:0010165	response to X-ray	3/158	26/28891	0.000380402	0.004296376	0.002759836	Ccnd2/Anxa1/Lrp2	3
GO:0015850	organic hydroxy compound transport	8/158	318/28891	0.000380985	0.004296376	0.002759836	Lgals3/Spp1/Ptgs1/Actb/Anxa2/Apoe/Rtn4/Egf	8
GO:0006909	phagocytosis	7/158	242/28891	0.00038725	0.004352325	0.002795776	Anxa3/Myh9/Rack1/Anxa1/Gsn/Rac1/Cdc42	7
GO:0042391	regulation of membrane potential	10/158	491/28891	0.000390754	0.004362327	0.002802201	Slc34a1/Rack1/Ywhae/Calm1/Flna/Jup/Ucp2/Clic1/Vcp/Atp1b1	10
GO:0051656	establishment of organelle localization	10/158	491/28891	0.000390754	0.004362327	0.002802201	Cfl1/Myh9/Map4/Sdc1/Eif6/Ctnnb1/Uchl1/Cdc42/Rab11a/Arf1	10
GO:0034599	cellular response to oxidative stress	7/158	243/28891	0.000396934	0.004381682	0.002814634	Cfl1/Lcn2/Cyp1b1/Rack1/Anxa1/Txn1/Prdx2	7
GO:0010038	response to metal ion	8/158	320/28891	0.000397154	0.004381682	0.002814634	Slc34a1/Calm1/Gsn/Cp/Clic4/Slc12a2/Junb/Iqgap1	8
GO:0030858	positive regulation of epithelial cell differentiation	4/158	63/28891	0.000399051	0.004381682	0.002814634	Sfn/Cd24a/Ctnnb1/Serpine1	4
GO:0090303	positive regulation of wound healing	4/158	63/28891	0.000399051	0.004381682	0.002814634	Cldn4/Anxa1/Serpine1/Prdx2	4
GO:1903078	positive regulation of protein localization to plasma membrane	4/158	63/28891	0.000399051	0.004381682	0.002814634	Lgals3/Rack1/Sptbn1/Rab11a	4
GO:2000058	regulation of ubiquitin-dependent protein catabolic process	6/158	175/28891	0.000416189	0.004536282	0.002913944	Clu/Rack1/Il33/Vcp/Egf/Rpl11	6
GO:1901800	positive regulation of proteasomal protein catabolic process	5/158	114/28891	0.000417207	0.004536282	0.002913944	Clu/Rack1/Il33/Vcp/Nupr1	5
GO:1905477	positive regulation of protein localization to membrane	5/158	114/28891	0.000417207	0.004536282	0.002913944	Lgals3/Rack1/Arpc2/Sptbn1/Rab11a	5
GO:0006749	glutathione metabolic process	4/158	64/28891	0.000423855	0.004555402	0.002926226	Gpx3/Gsto1/Gsta4/Idh1	4
GO:0048260	positive regulation of receptor-mediated endocytosis	4/158	64/28891	0.000423855	0.004555402	0.002926226	Clu/Anxa2/Serpine1/Egf	4
GO:0006883	intracellular sodium ion homeostasis	3/158	27/28891	0.00042624	0.004555402	0.002926226	Spp1/Slc12a2/Atp1b1	3

GO:0070841	inclusion body assembly	3/158	27/28891	0.00042624	0.004555402	0.002926226	Clu/Apoe/Vcp	3
GO:0042445	hormone metabolic process	7/158	246/28891	0.000427154	0.004555402	0.002926226	Spp1/Cyp1b1/Rbp1/Srd5a1/Rdh10/Adam10/Ctsl	7
GO:1901888	regulation of cell junction assembly	7/158	246/28891	0.000427154	0.004555402	0.002926226	Cfl1/Myh9/Rac1/Ctnnb1/Mmp14/S100a10/Iqgap1	7
GO:1904064	positive regulation of cation transmembrane transport	6/158	176/28891	0.000428958	0.004560071	0.002929224	Gsto1/Lcn2/Calm1/Flna/Arf1/Atp1b1	6
GO:0032231	regulation of actin filament bundle assembly	5/158	115/28891	0.000434268	0.004601864	0.002956071	Cfl1/Flna/Rac1/Cdc42/S100a10	5
GO:0002548	monocyte chemotaxis	4/158	65/28891	0.000449741	0.004735757	0.003042079	Lgals3/S100a14/Anxa1/Serpine1	4
GO:0006911	phagocytosis, engulfment	4/158	65/28891	0.000449741	0.004735757	0.003042079	Myh9/Gsn/Rac1/Cdc42	4
GO:0050727	regulation of inflammatory response	9/158	411/28891	0.000455317	0.004779392	0.003070109	Anxa1/Lpl/Ier3/Il33/Apoe/Cd24a/Serpine1/Nupr1/Cd200r3	9
GO:0006874	intracellular calcium ion homeostasis	8/158	327/28891	0.000458204	0.004794627	0.003079895	Gsto1/Spp1/Ywhae/Calm1/Flna/Apoe/Micu1/Atp1b1	8
GO:0055081	monoatomic anion homeostasis	3/158	28/28891	0.000475479	0.004942043	0.00317459	Spp1/Slc12a1/Slc12a2	3
GO:0030193	regulation of blood coagulation	4/158	66/28891	0.000476734	0.004942043	0.00317459	Anxa2/Apoe/Serpine1/Prdx2	4
GO:0060999	positive regulation of dendritic spine development	4/158	66/28891	0.000476734	0.004942043	0.00317459	Cfl1/Apoe/Rac1/Arf1	4
GO:0106027	neuron projection organization	5/158	118/28891	0.000488616	0.005049538	0.00324364	Cfl1/Apoe/Cdc42/Adam10/Arf1	5
GO:0003015	heart process	7/158	252/28891	0.000493108	0.005080225	0.003263353	Calm1/Gsn/Flna/Jup/Rac1/Cdc42/Atp1b1	7
GO:0071826	protein-RNA complex organization	6/158	183/28891	0.000527133	0.005408589	0.003474282	Rpsa/Rps5/Eif6/Vcp/Rplp0/Rpl11	6
GO:0060571	morphogenesis of an epithelial fold	3/158	29/28891	0.00052822	0.005408589	0.003474282	Cfl1/Rdh10/Ctnnb1	3
GO:0032233	positive regulation of actin filament bundle assembly	4/158	68/28891	0.00053415	0.005419428	0.003481245	Flna/Rac1/Cdc42/S100a10	4
GO:0061045	negative regulation of wound healing	4/158	68/28891	0.00053415	0.005419428	0.003481245	Anxa2/Apoe/Serpine1/Slc12a2	4
GO:1900046	regulation of hemostasis	4/158	68/28891	0.00053415	0.005419428	0.003481245	Anxa2/Apoe/Serpine1/Prdx2	4
GO:0071496	cellular response to external stimulus	8/158	335/28891	0.000537091	0.005432757	0.003489807	Slc34a1/Cdkn1a/Lpl/Ywhag/Rac1/Ctnnb1/Itga6/Col1a1	8
GO:0001822	kidney development	8/158	336/28891	0.000547681	0.005509019	0.003538795	Basp1/Spp1/Sox4/Cd24a/Rdh10/Slc12a1/Ctnnb1/Lrp2	8
GO:0060840	artery development	5/158	121/28891	0.000547931	0.005509019	0.003538795	Sox4/Apoe/Rtn4/Ctnnb1/Lrp2	5
GO:0010466	negative regulation of peptidase activity	7/158	257/28891	0.00055406	0.005553906	0.003567629	Wfdc2/Sfn/Serpinb6b/Cstb/Serpinb6a/Timp3/Serpine1	7
GO:0061951	establishment of protein localization to plasma membrane	4/158	69/28891	0.000564626	0.005642882	0.003624784	Krt18/Rack1/Sptbn1/Rab11a	4
GO:0062013	positive regulation of small molecule metabolic process	6/158	186/28891	0.000574201	0.005721442	0.003675247	Myh9/Anxa1/Apoe/Cd74/Rdh10/Vcp	6
GO:0000470	maturation of LSU-rRNA	3/158	30/28891	0.000584565	0.005755981	0.003697434	Eif6/Las1l/Rpl7	3
GO:0045730	respiratory burst	3/158	30/28891	0.000584565	0.005755981	0.003697434	Cd24a/Rac1/Prdx2	3
GO:0098869	cellular oxidant detoxification	3/158	30/28891	0.000584565	0.005755981	0.003697434	Gpx3/Txn1/Prdx2	3
GO:1990000	amyloid fibril formation	3/158	30/28891	0.000584565	0.005755981	0.003697434	Clu/Gsn/Apoe	3
GO:0050678	regulation of epithelial cell proliferation	9/158	426/28891	0.000588534	0.005778015	0.003711588	Sfn/Ccnd2/Apoe/Rtn4/Ctnnb1/Nupr1/Egf/Cdc42/Ctsl	9
GO:0032963	collagen metabolic process	5/158	123/28891	0.000590375	0.005779094	0.003712281	Mmp7/Serpine1/Mmp14/Col1a1/Ctsl	5
GO:0000079	regulation of cyclin-dependent protein serine/threonine kinase activity	4/158	70/28891	0.000596318	0.005803234	0.003727788	Sfn/Cdkn1a/Ccnd2/Actb	4
GO:0097006	regulation of plasma lipoprotein particle levels	4/158	70/28891	0.000596318	0.005803234	0.003727788	Anxa2/Lpl/Apoe/Arf1	4
GO:0043534	blood vessel endothelial cell migration	5/158	124/28891	0.000612499	0.005943374	0.003817809	Cyp1b1/Myh9/Anxa1/Jup/Apoe	5
GO:0006814	sodium ion transport	7/158	262/28891	0.000620863	0.006007075	0.003858728	Slc34a1/Slc12a1/Fxyd2/Slc12a2/Slc6a19/Arf1/Atp1b1	7
GO:1902074	response to salt	8/158	343/28891	0.00062664	0.006045449	0.003883378	Krt8/Slc34a1/Calm1/Clic4/Ly6a/Slc12a2/Junb/Iqgap1	8
GO:0050818	regulation of coagulation	4/158	71/28891	0.000629252	0.006053147	0.003888323	Anxa2/Apoe/Serpine1/Prdx2	4
GO:0098659	inorganic cation import across plasma membrane	5/158	125/28891	0.000635239	0.00607572	0.003902823	Slc34a1/Lcn2/Slc12a1/Slc12a2/Atp1b1	5
GO:0099587	inorganic ion import across plasma membrane	5/158	125/28891	0.000635239	0.00607572	0.003902823	Slc34a1/Lcn2/Slc12a1/Slc12a2/Atp1b1	5
GO:0044403	biological process involved in symbiotic interaction	7/158	264/28891	0.000649311	0.006182084	0.003971147	Cfl1/Gsn/Bsg/Apoe/Cd74/Hspa8/Cdc42	7
GO:0042886	amide transport	9/158	432/28891	0.000650063	0.006182084	0.003971147	Mmp7/Myh9/Anxa1/Sox4/Ucp2/Cd74/Rac1/Slc12a1/Lrp2	9
GO:0071466	cellular response to xenobiotic stimulus	6/158	191/28891	0.000659836	0.006255965	0.004018606	Gsto1/Gsta4/Cyp1b1/Fmo5/Serpine1/Uchl1	6
GO:0032507	maintenance of protein location in cell	4/158	72/28891	0.000663455	0.006255965	0.004018606	Tmsb4x/Gsn/Txn1/Tmsb10	4
GO:0034394	protein localization to cell surface	4/158	72/28891	0.000663455	0.006255965	0.004018606	Flna/Ctnnb1/Egf/Rab11a	4
GO:0009306	protein secretion	9/158	434/28891	0.000671706	0.006315929	0.004057124	Myh9/Anxa1/Sox4/Bsg/Apoe/Ucp2/Rac1/Slc12a2/Arf1	9
GO:0035592	establishment of protein localization to extracellular region	9/158	435/28891	0.000682746	0.006401706	0.004112225	Myh9/Anxa1/Sox4/Bsg/Apoe/Ucp2/Rac1/Slc12a2/Arf1	9
GO:0051090	regulation of DNA-binding transcription factor activity	9/158	436/28891	0.000693935	0.006488386	0.004167905	Clu/Cyp1b1/Anxa3/Flna/Jup/Ctnnb1/Nupr1/Prdx2/Rpl11	9
GO:0010952	positive regulation of peptidase activity	6/158	193/28891	0.000696733	0.006496356	0.004173024	Cldn4/Rack1/Prss22/Gsn/Vcp/Ctsl	6
GO:2001244	positive regulation of intrinsic apoptotic signaling pathway	4/158	73/28891	0.000698954	0.006498909	0.004174664	Clu/Rack1/Nupr1/Rpl11	4
GO:0001516	prostaglandin biosynthetic process	3/158	32/28891	0.000708451	0.00653262	0.004196319	Ptgs1/Anxa1/Cd74	3
GO:0042572	retinol metabolic process	3/158	32/28891	0.000708451	0.00653262	0.004196319	Cyp1b1/Rbp1/Rdh10	3
GO:0046457	prostanoid biosynthetic process	3/158	32/28891	0.000708451	0.00653262	0.004196319	Ptgs1/Anxa1/Cd74	3
GO:0007051	spindle organization	6/158	194/28891	0.000715771	0.006563855	0.004216383	Myh9/Flna/Tubb5/Map4/Vcp/Rab11a	6
GO:2001242	regulation of intrinsic apoptotic signaling pathway	6/158	194/28891	0.000715771	0.006563855	0.004216383	Clu/Rack1/Cd74/Ctnnb1/Nupr1/Rpl11	6
GO:1901890	positive regulation of cell junction assembly	5/158	129/28891	0.000732576	0.006699554	0.004303552	Cfl1/Myh9/Rac1/S100a10/Iqgap1	5
GO:1904029	regulation of cyclin-dependent protein kinase activity	4/158	74/28891	0.000735777	0.006710445	0.004310548	Sfn/Cdkn1a/Ccnd2/Actb	4
GO:0072001	renal system development	8/158	352/28891	0.000741421	0.006743495	0.004331777	Basp1/Spp1/Sox4/Cd24a/Rdh10/Slc12a1/Ctnnb1/Lrp2	8
GO:0071692	protein localization to extracellular region	9/158	441/28891	0.000752148	0.006822472	0.004382509	Myh9/Anxa1/Sox4/Bsg/Apoe/Ucp2/Rac1/Slc12a2/Arf1	9
GO:1904377	positive regulation of protein localization to cell periphery	4/158	75/28891	0.00077395	0.007001211	0.004497325	Lgals3/Rack1/Sptbn1/Rab11a	4
GO:0035987	endodermal cell differentiation	3/158	33/28891	0.000776182	0.007002421	0.004498102	Myh9/Ctr9/Ctnnb1	3
GO:0051346	negative regulation of hydrolase activity	8/158	356/28891	0.000797593	0.007176184	0.004609721	Wfdc2/Sfn/Anxa1/Serpinb6b/Cstb/Serpinb6a/Timp3/Serpine1	8
GO:0048259	regulation of receptor-mediated endocytosis	5/158	132/28891	0.000812586	0.007280081	0.004676461	Clu/Anxa2/Serpine1/Egf/Arf1	5
GO:0046824	positive regulation of nucleocytoplasmic transport	4/158	76/28891	0.000813502	0.007280081	0.004676461	Sfn/Ywhae/Flna/Jup	4
GO:0002828	regulation of type 2 immune response	3/158	34/28891	0.000847896	0.007507366	0.00482246	Anxa1/Il33/Cd74	3
GO:0006972	hyperosmotic response	3/158	34/28891	0.000847896	0.007507366	0.00482246	Rac1/Slc12a2/Micu1	3
GO:0018149	peptide cross-linking	3/158	34/28891	0.000847896	0.007507366	0.00482246	Ivl/Sprr1a/Anxa1	3
GO:0055075	potassium ion homeostasis	3/158	34/28891	0.000847896	0.007507366	0.00482246	Slc12a1/Slc12a2/Atp1b1	3
GO:0042246	tissue regeneration	4/158	77/28891	0.000854459	0.007545463	0.004846933	Cdkn1a/Anxa1/Pkm/Serpine1	4
GO:0052548	regulation of endopeptidase activity	8/158	360/28891	0.00085714	0.007549162	0.004849309	Sfn/Rack1/Gsn/Serpinb6b/Serpinb6a/Timp3/Serpine1/Vcp	8
GO:0022604	regulation of cell morphogenesis	7/158	277/28891	0.000860399	0.007557927	0.004854939	Cfl1/Cldn4/Myh9/Anxa1/Capzb/Myl12a/Rac1	7
GO:0060048	cardiac muscle contraction	5/158	134/28891	0.000869432	0.007617227	0.004893031	Calm1/Gsn/Flna/Jup/Atp1b1	5
GO:0045861	negative regulation of proteolysis	8/158	362/28891	0.000888224	0.007761501	0.004985708	Wfdc2/Sfn/Serpinb6b/Cstb/Serpinb6a/Timp3/Serpine1/Rpl11	8
GO:0050867	positive regulation of cell activation	9/158	454/28891	0.000922371	0.008029305	0.005157735	Cdkn1a/Actb/Anxa1/Flna/Sox4/Il33/Cd74/Cd24a/Mmp14	9
GO:1901020	negative regulation of calcium ion transmembrane transporter activity	3/158	35/28891	0.000923683	0.008029305	0.005157735	Gsto1/Ywhae/Calm1	3
GO:0014009	glial cell proliferation	4/158	79/28891	0.000940699	0.00815598	0.005239107	Clu/Sox4/Il33/Ctnnb1	4
GO:0002688	regulation of leukocyte chemotaxis	5/158	137/28891	0.000960188	0.008281928	0.005320011	S100a14/Cd74/Rac1/Serpine1/Adam10	5
GO:2000177	regulation of neural precursor cell proliferation	5/158	137/28891	0.000960188	0.008281928	0.005320011	Flna/Cd24a/Ctnnb1/Egf/Lrp2	5
GO:1904062	regulation of monoatomic cation transmembrane transport	8/158	367/28891	0.000969911	0.008344232	0.005360033	Gsto1/Lcn2/Ywhae/Calm1/Flna/Fxyd2/Arf1/Atp1b1	8

GO:0006081	cellular aldehyde metabolic process	4/158	80/28891	0.000986036	0.008461149	0.005435136	Cyp1b1/Taldo1/Rdh10/Idh1	4
GO:0050868	negative regulation of T cell activation	5/158	138/28891	0.000991949	0.008490064	0.00545371	Lgals3/Anxa1/Cd74/Cd24a/Prdx2	5
GO:0098739	import across plasma membrane	6/158	207/28891	0.001001789	0.008551282	0.005493034	Slc34a1/Lcn2/Slc12a1/Slc12a2/Lrp2/Atp1b1	6
GO:0051402	neuron apoptotic process	8/158	369/28891	0.001004225	0.008551282	0.005493034	Clu/Lcn2/Apoe/Ucp2/Ctnnb1/Nupr1/Cdc42/Prdx2	8
GO:0099175	regulation of postsynapse organization	5/158	139/28891	0.001024484	0.008701598	0.005589592	Cfl1/Apoe/Hspa8/Cdc42/Adam10	5
GO:0000281	mitotic cytokinesis	4/158	81/28891	0.001032887	0.008728551	0.005606905	Cfl1/Sptbn1/Arf1/Iqgap1	4
GO:0032413	negative regulation of ion transmembrane transporter activity	4/158	81/28891	0.001032887	0.008728551	0.005606905	Gsto1/Ywhae/Calm1/Fxyd2	4
GO:0033559	unsaturated fatty acid metabolic process	5/158	140/28891	0.001057805	0.008916549	0.005727669	Ptgs1/Cyp1b1/Anxa1/Cd74/Mgst3	5
GO:1901652	response to peptide	9/158	464/28891	0.001073592	0.008977441	0.005766784	Cfl1/Anxa1/Pkm/Lpl/Ywhag/Rac1/Eif6/Ctnnb1/Rab10	9
GO:0043524	negative regulation of neuron apoptotic process	6/158	210/28891	0.001078763	0.008977441	0.005766784	Clu/Lcn2/Apoe/Ucp2/Ctnnb1/Prdx2	6
GO:0051048	negative regulation of secretion	6/158	210/28891	0.001078763	0.008977441	0.005766784	Ptgs1/Anxa1/Apoe/Ucp2/Cd74/Egf	6
GO:0008625	extrinsic apoptotic signaling pathway via death domain receptors	4/158	82/28891	0.001081281	0.008977441	0.005766784	Lgals3/Timp3/Serpine1/Pea15a	4
GO:0051851	modulation by host of symbiont process	4/158	82/28891	0.001081281	0.008977441	0.005766784	Cfl1/Apoe/Hspa8/Cdc42	4
GO:0071677	positive regulation of mononuclear cell migration	4/158	82/28891	0.001081281	0.008977441	0.005766784	Lgals3/S100a14/Serpine1/Adam10	4
GO:0051491	positive regulation of filopodium assembly	3/158	37/28891	0.001087827	0.008977441	0.005766784	Arpc2/Rac1/Cdc42	3
GO:0071624	positive regulation of granulocyte chemotaxis	3/158	37/28891	0.001087827	0.008977441	0.005766784	S100a14/Cd74/Rac1	3
GO:0045621	positive regulation of lymphocyte differentiation	5/158	141/28891	0.001091924	0.008977441	0.005766784	Actb/Anxa1/Sox4/Cd74/Mmp14	5
GO:0045727	positive regulation of translation	5/158	141/28891	0.001091924	0.008977441	0.005766784	Cyp1b1/Pkm/Sox4/Eif6/Eif5a	5
GO:0008360	regulation of cell shape	5/158	142/28891	0.001126851	0.009219192	0.005922075	Cfl1/Myh9/Anxa1/Myl12a/Rac1	5
GO:0032434	regulation of proteasomal ubiquitin-dependent protein catabolic process	5/158	142/28891	0.001126851	0.009219192	0.005922075	Clu/Rack1/Il33/Vcp/Rpl11	5
GO:2000179	positive regulation of neural precursor cell proliferation	4/158	83/28891	0.001131243	0.00922059	0.005922974	Flna/Ctnnb1/Egf/Lrp2	4
GO:0071805	potassium ion transmembrane transport	6/158	212/28891	0.001132547	0.00922059	0.005922974	Ywhae/Flna/Slc12a1/Fxyd2/Slc12a2/Atp1b1	6
GO:0043243	positive regulation of protein-containing complex disassembly	3/158	38/28891	0.001176356	0.009507694	0.006107399	Cfl1/Dstn/Eif5a	3
GO:0046825	regulation of protein export from nucleus	3/158	38/28891	0.001176356	0.009507694	0.006107399	Sfn/Ywhae/Txn1	3
GO:0071364	cellular response to epidermal growth factor stimulus	3/158	38/28891	0.001176356	0.009507694	0.006107399	Cfl1/Arpc2/Iqgap1	3
GO:0003007	heart morphogenesis	7/158	294/28891	0.00121437	0.009767633	0.006274374	Flna/Sox4/Rac1/Rtn4/Ctnnb1/Cdc42/Lrp2	7
GO:0022898	regulation of transmembrane transporter activity	7/158	294/28891	0.00121437	0.009767633	0.006274374	Gsto1/Cfl1/Actb/Ywhae/Calm1/Fxyd2/Atp1b1	7
GO:0150076	neuroinflammatory response	4/158	85/28891	0.001235986	0.009893812	0.006355427	Clu/Il33/Nupr1/Cd200r3	4
GO:2000379	positive regulation of reactive oxygen species metabolic process	4/158	85/28891	0.001235986	0.009893812	0.006355427	Cdkn1a/Lcn2/Cyp1b1/Eif5a	4
GO:0071375	cellular response to peptide hormone stimulus	7/158	296/28891	0.001262562	0.010082375	0.006476553	Cfl1/Pkm/Lpl/Ywhag/Rac1/Ctnnb1/Rab10	7
GO:0120033	negative regulation of plasma membrane bounded cell projection assembly	3/158	39/28891	0.001269302	0.010087929	0.006480121	Cfl1/Capzb/Map4	3
GO:1902003	regulation of amyloid-beta formation	3/158	39/28891	0.001269302	0.010087929	0.006480121	Clu/Apoe/Rtn4	3
GO:0060998	regulation of dendritic spine development	4/158	86/28891	0.001290819	0.010210321	0.006558741	Cfl1/Apoe/Rac1/Arf1	4
GO:0072332	intrinsic apoptotic signaling pathway by p53 class mediator	4/158	86/28891	0.001290819	0.010210321	0.006558741	Cdkn1a/Cd74/Nupr1/Rpl11	4
GO:0034372	very-low-density lipoprotein particle remodeling	2/158	10/28891	0.001299414	0.010229824	0.006571269	Lpl/Apoe	2
GO:0060982	coronary artery morphogenesis	2/158	10/28891	0.001299414	0.010229824	0.006571269	Ctnnb1/Lrp2	2
GO:0006690	icosanoid metabolic process	5/158	147/28891	0.001314046	0.01032067	0.006629625	Ptgs1/Cyp1b1/Anxa1/Cd74/Mgst3	5
GO:0072521	purine-containing compound metabolic process	9/158	479/28891	0.001337562	0.010480706	0.006732427	Spp1/Acsm2/Taldo1/Fmo5/Hspa8/Vcp/Ldhb/Atp1b1/Idh1	9
GO:0021799	cerebral cortex radially oriented cell migration	3/158	40/28891	0.001366745	0.010585141	0.006799512	Rac1/Rtn4/Ctnnb1	3
GO:0030574	collagen catabolic process	3/158	40/28891	0.001366745	0.010585141	0.006799512	Mmp7/Mmp14/Ctsl	3
GO:0035850	epithelial cell differentiation involved in kidney development	3/158	40/28891	0.001366745	0.010585141	0.006799512	Basp1/Cd24a/Ctnnb1	3
GO:0043001	Golgi to plasma membrane protein transport	3/158	40/28891	0.001366745	0.010585141	0.006799512	Krt18/Rack1/Sptbn1	3
GO:1905332	positive regulation of morphogenesis of an epithelium	3/158	40/28891	0.001366745	0.010585141	0.006799512	Lcn2/Ctnnb1/Egf	3
GO:0034248	regulation of amide metabolic process	9/158	482/28891	0.001396159	0.010787916	0.006929768	Clu/Cyp1b1/Rack1/Pkm/Sox4/Apoe/Eif6/Rtn4/Eif5a	9
GO:0031345	negative regulation of cell projection organization	6/158	221/28891	0.001400473	0.010796258	0.006935126	Cfl1/Capzb/Flna/Map4/Apoe/Rtn4	6
GO:0046890	regulation of lipid biosynthetic process	6/158	222/28891	0.001432987	0.011021455	0.007079784	Anxa1/Apoe/Cd74/Rdh10/Eif6/Idh1	6
GO:0034341	response to type II interferon	5/158	150/28891	0.001436838	0.011025666	0.007082489	Capg/Calm1/Gsn/Cd74/Cdc42	5
GO:0032102	negative regulation of response to external stimulus	9/158	485/28891	0.00145681	0.011153283	0.007164466	Anxa2/Ier3/Il33/Apoe/Cd24a/Hspa8/Serpine1/Slc12a2/Prdx2	9
GO:0032410	negative regulation of transporter activity	4/158	89/28891	0.001465499	0.011167977	0.007173904	Gsto1/Ywhae/Calm1/Fxyd2	4
GO:0032506	cytokinetic process	3/158	41/28891	0.001468766	0.011167977	0.007173904	Myh9/Arf1/Iqgap1	3
GO:0070849	response to epidermal growth factor	3/158	41/28891	0.001468766	0.011167977	0.007173904	Cfl1/Arpc2/Iqgap1	3
GO:0034763	negative regulation of transmembrane transport	5/158	151/28891	0.001479587	0.011199231	0.007193981	Gsto1/Ywhae/Calm1/Fxyd2/Pea15a	5
GO:0051928	positive regulation of calcium ion transport	5/158	151/28891	0.001479587	0.011199231	0.007193981	Gsto1/Lgals3/Calm1/Serpine1/Atp1b1	5
GO:0031016	pancreas development	4/158	90/28891	0.001527209	0.011506669	0.007391468	Clu/Anxa1/Sox4/Ctnnb1	4
GO:0033627	cell adhesion mediated by integrin	4/158	90/28891	0.001527209	0.011506669	0.007391468	Cyp1b1/Cd24a/Serpine1/Itga6	4
GO:0050866	negative regulation of cell activation	6/158	225/28891	0.001533993	0.011506669	0.007391468	Lgals3/Anxa1/Apoe/Cd74/Cd24a/Prdx2	6
GO:0061351	neural precursor cell proliferation	6/158	225/28891	0.001533993	0.011506669	0.007391468	Flna/Cd24a/Ctnnb1/Rab10/Egf/Lrp2	6
GO:0032409	regulation of transporter activity	7/158	307/28891	0.001555033	0.011583997	0.007441141	Gsto1/Cfl1/Actb/Ywhae/Calm1/Fxyd2/Atp1b1	7
GO:0072331	signal transduction by p53 class mediator	5/158	153/28891	0.001567886	0.011583997	0.007441141	Cdkn1a/Sox4/Cd74/Nupr1/Rpl11	5
GO:1903038	negative regulation of leukocyte cell-cell adhesion	5/158	153/28891	0.001567886	0.011583997	0.007441141	Lgals3/Anxa1/Cd74/Cd24a/Prdx2	5
GO:0071827	plasma lipoprotein particle organization	3/158	42/28891	0.001575443	0.011583997	0.007441141	Lpl/Apoe/Arf1	3
GO:0030643	intracellular phosphate ion homeostasis	2/158	11/28891	0.001582475	0.011583997	0.007441141	Slc34a1/Spp1	2
GO:0033629	negative regulation of cell adhesion mediated by integrin	2/158	11/28891	0.001582475	0.011583997	0.007441141	Cyp1b1/Serpine1	2
GO:0034370	triglyceride-rich lipoprotein particle remodeling	2/158	11/28891	0.001582475	0.011583997	0.007441141	Lpl/Apoe	2
GO:0048664	neuron fate determination	2/158	11/28891	0.001582475	0.011583997	0.007441141	Ctnnb1/Cdc42	2
GO:0061043	regulation of vascular wound healing	2/158	11/28891	0.001582475	0.011583997	0.007441141	Serpine1/Slc12a2	2
GO:0061299	retina vasculature morphogenesis in camera-type eye	2/158	11/28891	0.001582475	0.011583997	0.007441141	Cyp1b1/Clic4	2
GO:2001280	positive regulation of unsaturated fatty acid biosynthetic process	2/158	11/28891	0.001582475	0.011583997	0.007441141	Anxa1/Cd74	2
GO:0032436	positive regulation of proteasomal ubiquitin-dependent protein catabolic process	4/158	91/28891	0.001590705	0.011618758	0.00746347	Clu/Rack1/Il33/Vcp	4
GO:0019216	regulation of lipid metabolic process	8/158	398/28891	0.001620561	0.011810987	0.007586951	Anxa1/Fmo5/Apoe/Cd74/Rdh10/Eif6/Arf1/Idh1	8
GO:0050767	regulation of neurogenesis	9/158	494/28891	0.001651604	0.011967777	0.007687667	Anxa2/Tnfrsf12a/Il33/Apoe/Cd24a/Rtn4/Ctnnb1/Lrp2/S100a10	9
GO:0051881	regulation of mitochondrial membrane potential	4/158	92/28891	0.001656014	0.011967777	0.007687667	Rack1/Ucp2/Clic1/Vcp	4
GO:1901019	regulation of calcium ion transmembrane transporter activity	4/158	92/28891	0.001656014	0.011967777	0.007687667	Gsto1/Ywhae/Calm1/Atp1b1	4
GO:0046660	female sex differentiation	5/158	155/28891	0.00166	0.011967777	0.007687667	Myh9/Rac1/Serpine1/Nupr1/Lrp2	5
GO:0098876	vesicle-mediated transport to the plasma membrane	5/158	155/28891	0.00166	0.011967777	0.007687667	Krt18/Rack1/Rab10/Sptbn1/Rab11a	5
GO:1902115	regulation of organelle assembly	6/158	229/28891	0.001676996	0.012057194	0.007745106	Arpc2/Gsn/Map4/Sdc1/Nupr1/Rab11a	6
GO:0001706	endoderm formation	3/158	43/28891	0.001686851	0.012057194	0.007745106	Myh9/Ctr9/Ctnnb1	3
GO:0006636	unsaturated fatty acid biosynthetic process	3/158	43/28891	0.001686851	0.012057194	0.007745106	Ptgs1/Anxa1/Cd74	3

GO:0007616	long-term memory	3/158	43/28891	0.001686851	0.012057194	0.007745106	Ccnd2/Lcn2/Apoe	3
GO:0001649	osteoblast differentiation	6/158	230/28891	0.001714279	0.01222706	0.007854221	Spp1/Pdlim7/Clic1/Ctnnb1/Col1a1/Junb	6
GO:0070663	regulation of leukocyte proliferation	7/158	313/28891	0.001735423	0.012351477	0.007934142	Lgals3/Cdkn1a/Anxa1/Il33/Cd74/Cd24a/Ctnnb1	7
GO:0006820	monoatomic anion transport	5/158	157/28891	0.001756029	0.012471541	0.008011267	Cldn4/Clic4/Clic1/Slc12a1/Slc12a2	5
GO:0043525	positive regulation of neuron apoptotic process	4/158	94/28891	0.001792182	0.012688206	0.008150445	Lcn2/Ctnnb1/Nupr1/Cdc42	4
GO:0010559	regulation of glycoprotein biosynthetic process	3/158	44/28891	0.001803067	0.012688206	0.008150445	Itm2b/Il33/Ctnnb1	3
GO:0034314	Arp2/3 complex-mediated actin nucleation	3/158	44/28891	0.001803067	0.012688206	0.008150445	Arpc2/Arpc1b/Arf1	3
GO:1901021	positive regulation of calcium ion transmembrane transporter activity	3/158	44/28891	0.001803067	0.012688206	0.008150445	Gsto1/Calm1/Atp1b1	3
GO:0071333	cellular response to glucose stimulus	5/158	158/28891	0.001805542	0.012688206	0.008150445	Myh9/Rack1/Sox4/Ucp2/Rac1	5
GO:1903037	regulation of leukocyte cell-cell adhesion	8/158	407/28891	0.001863063	0.012995949	0.008348128	Lgals3/Actb/Anxa1/Sox4/Cd74/Cd24a/Fut9/Prdx2	8
GO:1901379	regulation of potassium ion transmembrane transport	4/158	95/28891	0.001863093	0.012995949	0.008348128	Ywhae/Flna/Fxyd2/Atp1b1	4
GO:0002778	antibacterial peptide production	2/158	12/28891	0.00189216	0.012995949	0.008348128	Ivl/Mmp7	2
GO:0031392	regulation of prostaglandin biosynthetic process	2/158	12/28891	0.00189216	0.012995949	0.008348128	Anxa1/Cd74	2
GO:0034333	adherens junction assembly	2/158	12/28891	0.00189216	0.012995949	0.008348128	Actb/Ctnnb1	2
GO:0061517	macrophage proliferation	2/158	12/28891	0.00189216	0.012995949	0.008348128	Clu/Il33	2
GO:0061684	chaperone-mediated autophagy	2/158	12/28891	0.00189216	0.012995949	0.008348128	Clu/Hspa8	2
GO:0072182	regulation of nephron tubule epithelial cell differentiation	2/158	12/28891	0.00189216	0.012995949	0.008348128	Cd24a/Ctnnb1	2
GO:0106015	negative regulation of inflammatory response to wounding	2/158	12/28891	0.00189216	0.012995949	0.008348128	Il33/Cd24a	2
GO:1902947	regulation of tau-protein kinase activity	2/158	12/28891	0.00189216	0.012995949	0.008348128	Clu/Apoe	2
GO:1905907	negative regulation of amyloid fibril formation	2/158	12/28891	0.00189216	0.012995949	0.008348128	Clu/Apoe	2
GO:0071331	cellular response to hexose stimulus	5/158	160/28891	0.001907626	0.013075271	0.008399081	Myh9/Rack1/Sox4/Ucp2/Rac1	5
GO:0042771	intrinsic apoptotic signaling pathway in response to DNA damage by p53 class mediator	3/158	45/28891	0.001924162	0.013161582	0.008454524	Cdkn1a/Cd74/Nupr1	3
GO:0006414	translational elongation	4/158	96/28891	0.001935925	0.013214966	0.008488817	Rack1/Eef1b2/Eif5a/Eef1g	4
GO:0071326	cellular response to monosaccharide stimulus	5/158	161/28891	0.001960223	0.01335352	0.008577819	Myh9/Rack1/Sox4/Ucp2/Rac1	5
GO:0050863	regulation of T cell activation	8/158	411/28891	0.001979644	0.013458353	0.00864516	Lgals3/Actb/Anxa1/Sox4/Cd74/Cd24a/Ctnnb1/Prdx2	8
GO:0051054	positive regulation of DNA metabolic process	7/158	321/28891	0.002000796	0.013574505	0.008719771	Cyp1b1/Actb/Anxa3/Rac1/Ctnnb1/Egf/Cdc42	7
GO:0001824	blastocyst development	5/158	162/28891	0.002013873	0.013635511	0.008758959	Elf3/Ctr9/Rtn4/Junb/Atp1b1	5
GO:0030198	extracellular matrix organization	7/158	322/28891	0.002036061	0.013757835	0.008837536	Lgals3/Cyp1b1/Mmp7/Anxa2/Elf3/Mmp14/Col1a1	7
GO:0030195	negative regulation of blood coagulation	3/158	46/28891	0.002050208	0.01376981	0.008845228	Anxa2/Apoe/Serpine1	3
GO:0071825	protein-lipid complex organization	3/158	46/28891	0.002050208	0.01376981	0.008845228	Lpl/Apoe/Arf1	3
GO:1902991	regulation of amyloid precursor protein catabolic process	3/158	46/28891	0.002050208	0.01376981	0.008845228	Clu/Apoe/Rtn4	3
GO:1904019	epithelial cell apoptotic process	5/158	163/28891	0.002068587	0.013850606	0.008897129	Krt8/Krt18/Gsn/Serpine1/Nupr1	5
GO:0045229	external encapsulating structure organization	7/158	323/28891	0.002071805	0.013850606	0.008897129	Lgals3/Cyp1b1/Mmp7/Anxa2/Elf3/Mmp14/Col1a1	7
GO:0010976	positive regulation of neuron projection development	6/158	239/28891	0.002078836	0.013850606	0.008897129	Flna/Apoe/Cd24a/Fut9/Itga6/Iqgap1	6
GO:0060047	heart contraction	6/158	239/28891	0.002078836	0.013850606	0.008897129	Calm1/Gsn/Flna/Jup/Cdc42/Atp1b1	6
GO:0006766	vitamin metabolic process	4/158	98/28891	0.002087458	0.013880349	0.008916234	Gsto1/Rbp1/Rdh10/Lrp2	4
GO:0006936	muscle contraction	7/158	324/28891	0.002108034	0.013961541	0.008968389	Ptgs1/Calm1/Gsn/Flna/Jup/Tpm4/Atp1b1	7
GO:0043062	extracellular structure organization	7/158	324/28891	0.002108034	0.013961541	0.008968389	Lgals3/Cyp1b1/Mmp7/Anxa2/Elf3/Mmp14/Col1a1	7
GO:0006892	post-Golgi vesicle-mediated transport	4/158	99/28891	0.002166212	0.014248728	0.009152868	Krt18/Rack1/Rab10/Sptbn1	4
GO:0050921	positive regulation of chemotaxis	5/158	165/28891	0.002181263	0.014248728	0.009152868	S100a14/Cd74/Rac1/Serpine1/Adam10	5
GO:0042789	mRNA transcription by RNA polymerase II	3/158	47/28891	0.002181276	0.014248728	0.009152868	Anxa2/Flna/S100a10	3
GO:0060563	neuroepithelial cell differentiation	3/158	47/28891	0.002181276	0.014248728	0.009152868	Tubb5/Sox4/Serpine1	3
GO:0140236	translation at presynapse	3/158	47/28891	0.002181276	0.014248728	0.009152868	Rpl14/Rps5/Rpl7	3
GO:1900047	negative regulation of hemostasis	3/158	47/28891	0.002181276	0.014248728	0.009152868	Anxa2/Apoe/Serpine1	3
GO:1990573	potassium ion import across plasma membrane	3/158	47/28891	0.002181276	0.014248728	0.009152868	Slc12a1/Slc12a2/Atp1b1	3
GO:0016358	dendrite development	7/158	327/28891	0.002219668	0.014470124	0.009295084	Cfl1/Apoe/Rac1/Cdc42/Adam10/Arf1/Iqgap1	7
GO:0030836	positive regulation of actin filament depolymerization	2/158	13/28891	0.002228174	0.014470124	0.009295084	Cfl1/Dstn	2
GO:0042447	hormone catabolic process	2/158	13/28891	0.002228174	0.014470124	0.009295084	Spp1/Srd5a1	2
GO:0002790	peptide secretion	7/158	329/28891	0.002296592	0.014810597	0.009513792	Mmp7/Myh9/Anxa1/Sox4/Ucp2/Cd74/Rac1	7
GO:0016032	viral process	7/158	329/28891	0.002296592	0.014810597	0.009513792	Cfl1/Gsn/Bsg/Apoe/Cd74/Hspa8/Vcp	7
GO:0016052	carbohydrate catabolic process	5/158	167/28891	0.002298349	0.014810597	0.009513792	Pkm/Ier3/Ucp2/Eif6/Nupr1	5
GO:0050729	positive regulation of inflammatory response	5/158	167/28891	0.002298349	0.014810597	0.009513792	Lpl/Il33/Cd24a/Serpine1/Nupr1	5
GO:0086009	membrane repolarization	3/158	48/28891	0.002317434	0.014847588	0.009537554	Ywhae/Flna/Atp1b1	3
GO:0140241	translation at synapse	3/158	48/28891	0.002317434	0.014847588	0.009537554	Rpl14/Rps5/Rpl7	3
GO:0140242	translation at postsynapse	3/158	48/28891	0.002317434	0.014847588	0.009537554	Rpl14/Rps5/Rpl7	3
GO:0007043	cell-cell junction assembly	5/158	168/28891	0.002358578	0.015082246	0.009688289	Cldn4/Actb/Cldn7/Jup/Ctnnb1	5
GO:1904063	negative regulation of cation transmembrane transport	4/158	102/28891	0.002414738	0.015386251	0.009883571	Gsto1/Ywhae/Calm1/Fxyd2	4
GO:0043433	negative regulation of DNA-binding transcription factor activity	5/158	169/28891	0.002419947	0.015386251	0.009883571	Cyp1b1/Flna/Nupr1/Prdx2/Rpl11	5
GO:0071322	cellular response to carbohydrate stimulus	5/158	169/28891	0.002419947	0.015386251	0.009883571	Myh9/Rack1/Sox4/Ucp2/Rac1	5
GO:0045740	positive regulation of DNA replication	3/158	49/28891	0.002458747	0.015514743	0.00996611	Rac1/Egf/Cdc42	3
GO:0050819	negative regulation of coagulation	3/158	49/28891	0.002458747	0.015514743	0.00996611	Anxa2/Apoe/Serpine1	3
GO:1902624	positive regulation of neutrophil migration	3/158	49/28891	0.002458747	0.015514743	0.00996611	Cd74/Rac1/Rtn4	3
GO:1903018	regulation of glycoprotein metabolic process	3/158	49/28891	0.002458747	0.015514743	0.00996611	Itm2b/Il33/Ctnnb1	3
GO:0045921	positive regulation of exocytosis	4/158	103/28891	0.002501755	0.015756336	0.0101213	Anxa2/Sdc1/S100a10/Arf1	4
GO:0006813	potassium ion transport	6/158	249/28891	0.002549541	0.015895284	0.010210556	Ywhae/Flna/Slc12a1/Fxyd2/Slc12a2/Atp1b1	6
GO:0002775	antimicrobial peptide production	2/158	14/28891	0.002590222	0.015895284	0.010210556	Ivl/Mmp7	2
GO:0043589	skin morphogenesis	2/158	14/28891	0.002590222	0.015895284	0.010210556	Itga6/Col1a1	2
GO:0046598	positive regulation of viral entry into host cell	2/158	14/28891	0.002590222	0.015895284	0.010210556	Bsg/Cd74	2
GO:0051280	negative regulation of release of sequestered calcium ion into cytosol	2/158	14/28891	0.002590222	0.015895284	0.010210556	Gsto1/Calm1	2
GO:0071895	odontoblast differentiation	2/158	14/28891	0.002590222	0.015895284	0.010210556	Tubb5/Serpine1	2
GO:0072160	nephron tubule epithelial cell differentiation	2/158	14/28891	0.002590222	0.015895284	0.010210556	Cd24a/Ctnnb1	2
GO:0075294	positive regulation by symbiont of entry into host	2/158	14/28891	0.002590222	0.015895284	0.010210556	Bsg/Cd74	2
GO:0106014	regulation of inflammatory response to wounding	2/158	14/28891	0.002590222	0.015895284	0.010210556	Il33/Cd24a	2
GO:1904948	midbrain dopaminergic neuron differentiation	2/158	14/28891	0.002590222	0.015895284	0.010210556	Rac1/Ctnnb1	2
GO:2000644	regulation of receptor catabolic process	2/158	14/28891	0.002590222	0.015895284	0.010210556	Anxa2/Apoe	2
GO:2001279	regulation of unsaturated fatty acid biosynthetic process	2/158	14/28891	0.002590222	0.015895284	0.010210556	Anxa1/Cd74	2
GO:0042274	ribosomal small subunit biogenesis	4/158	104/28891	0.002590903	0.015895284	0.010210556	Rpsa/Rps5/Rps9/Rps13	4
GO:0019058	viral life cycle	6/158	250/28891	0.00260067	0.015895284	0.010210556	Gsn/Bsg/Apoe/Cd74/Hspa8/Vcp	6

GO:0006739	NADP metabolic process	3/158	50/28891	0.002605283	0.015895284	0.010210556	Taldo1/Fmo5/Idh1	3
GO:0033628	regulation of cell adhesion mediated by integrin	3/158	50/28891	0.002605283	0.015895284	0.010210556	Cyp1b1/Cd24a/Serpine1	3
GO:0042092	type 2 immune response	3/158	50/28891	0.002605283	0.015895284	0.010210556	Anxa1/Il33/Cd74	3
GO:0010563	negative regulation of phosphorus metabolic process	8/158	430/28891	0.002614293	0.015895284	0.010210556	Cdkn1a/Actb/Rack1/Ywhae/Apoe/Cadm4/Uchl1/Iqgap1	8
GO:0045936	negative regulation of phosphate metabolic process	8/158	430/28891	0.002614293	0.015895284	0.010210556	Cdkn1a/Actb/Rack1/Ywhae/Apoe/Cadm4/Uchl1/Iqgap1	8
GO:0060828	regulation of canonical Wnt signaling pathway	6/158	251/28891	0.002652566	0.016098663	0.010341199	Sox4/Apoe/Ctnnb1/Vcp/Egf/Col1a1	6
GO:0032675	regulation of interleukin-6 production	5/158	173/28891	0.002677077	0.016188556	0.010398943	Lpl/Bsg/Il33/Cd74/Cd24a	5
GO:0120254	olefinic compound metabolic process	5/158	173/28891	0.002677077	0.016188556	0.010398943	Ptgs1/Cyp1b1/Rbp1/Srd5a1/Rdh10	5
GO:0002696	positive regulation of leukocyte activation	8/158	432/28891	0.002689408	0.016233714	0.010427951	Cdkn1a/Actb/Anxa1/Sox4/Il33/Cd74/Cd24a/Mmp14	8
GO:0030217	T cell differentiation	7/158	339/28891	0.002712577	0.016344009	0.0104988	Actb/Anxa1/Sox4/Cd74/Ctnnb1/Prdx2/Ctsl	7
GO:0051250	negative regulation of lymphocyte activation	5/158	174/28891	0.002744336	0.016505574	0.010602584	Lgals3/Anxa1/Cd74/Cd24a/Prdx2	5
GO:0016601	Rac protein signal transduction	3/158	51/28891	0.002757104	0.016522822	0.010613664	Rac1/Rtn4/Cadm4	3
GO:0061005	cell differentiation involved in kidney development	3/158	51/28891	0.002757104	0.016522822	0.010613664	Basp1/Cd24a/Ctnnb1	3
GO:0007596	blood coagulation	5/158	175/28891	0.002812811	0.016796356	0.010789372	Anxa2/Flna/Apoe/Serpine1/Prdx2	5
GO:0022618	protein-RNA complex assembly	5/158	175/28891	0.002812811	0.016796356	0.010789372	Rpsa/Rps5/Eif6/Rplp0/Rpl11	5
GO:0001678	intracellular glucose homeostasis	5/158	176/28891	0.002882514	0.017181844	0.011036995	Myh9/Rack1/Sox4/Ucp2/Rac1	5
GO:0015833	peptide transport	7/158	343/28891	0.002894289	0.017221275	0.011062324	Mmp7/Myh9/Anxa1/Sox4/Ucp2/Cd74/Rac1	7
GO:0009299	mRNA transcription	3/158	52/28891	0.002914272	0.017247942	0.011079455	Anxa2/Flna/S100a10	3
GO:0034605	cellular response to heat	3/158	52/28891	0.002914272	0.017247942	0.011079455	Myof/Ywhae/Vcp	3
GO:0042304	regulation of fatty acid biosynthetic process	3/158	52/28891	0.002914272	0.017247942	0.011079455	Anxa1/Cd74/Eif6	3
GO:0019751	polyol metabolic process	4/158	108/28891	0.002969323	0.017348356	0.011143957	Myh9/Myof/Miox/Lrp2	4
GO:0046849	bone remodeling	4/158	108/28891	0.002969323	0.017348356	0.011143957	Slc34a1/Spp1/Rac1/Ctnnb1	4
GO:0061640	cytoskeleton-dependent cytokinesis	4/158	108/28891	0.002969323	0.017348356	0.011143957	Cfl1/Sptbn1/Arf1/Iqgap1	4
GO:0001711	endodermal cell fate commitment	2/158	15/28891	0.002978013	0.017348356	0.011143957	Ctr9/Ctnnb1	2
GO:0030007	intracellular potassium ion homeostasis	2/158	15/28891	0.002978013	0.017348356	0.011143957	Slc12a2/Atp1b1	2
GO:0032372	negative regulation of sterol transport	2/158	15/28891	0.002978013	0.017348356	0.011143957	Apoe/Egf	2
GO:0032375	negative regulation of cholesterol transport	2/158	15/28891	0.002978013	0.017348356	0.011143957	Apoe/Egf	2
GO:1905048	regulation of metallopeptidase activity	2/158	15/28891	0.002978013	0.017348356	0.011143957	Cldn4/Timp3	2
GO:2000343	positive regulation of chemokine (C-X-C motif) ligand 2 production	2/158	15/28891	0.002978013	0.017348356	0.011143957	Lpl/Cd74	2
GO:0006941	striated muscle contraction	5/158	178/28891	0.003025656	0.017564593	0.011282859	Calm1/Gsn/Flna/Jup/Atp1b1	5
GO:0050817	coagulation	5/158	178/28891	0.003025656	0.017564593	0.011282859	Anxa2/Flna/Apoe/Serpine1/Prdx2	5
GO:1901653	cellular response to peptide	7/158	346/28891	0.003036592	0.017597471	0.011303979	Cfl1/Pkm/Lpl/Ywhag/Rac1/Ctnnb1/Rab10	7
GO:0090263	positive regulation of canonical Wnt signaling pathway	4/158	109/28891	0.003069512	0.017707793	0.011374846	Sox4/Vcp/Egf/Col1a1	4
GO:0030865	cortical cytoskeleton organization	3/158	53/28891	0.003076848	0.017707793	0.011374846	Actn1/Rac1/Iqgap1	3
GO:0046596	regulation of viral entry into host cell	3/158	53/28891	0.003076848	0.017707793	0.011374846	Gsn/Bsg/Cd74	3
GO:1990748	cellular detoxification	3/158	53/28891	0.003076848	0.017707793	0.011374846	Gpx3/Txn1/Prdx2	3
GO:0007599	hemostasis	5/158	179/28891	0.00309912	0.017805273	0.011437464	Anxa2/Flna/Apoe/Serpine1/Prdx2	5
GO:2001243	negative regulation of intrinsic apoptotic signaling pathway	4/158	110/28891	0.003171985	0.018185803	0.011681903	Clu/Rack1/Cd74/Ctnnb1	4
GO:0006163	purine nucleotide metabolic process	8/158	444/28891	0.00317625	0.018185803	0.011681903	Acsm2/Taldo1/Fmo5/Hspa8/Vcp/Ldhb/Atp1b1/Idh1	8
GO:0021795	cerebral cortex cell migration	3/158	54/28891	0.003244892	0.018452215	0.011853036	Rac1/Rtn4/Ctnnb1	3
GO:0031018	endocrine pancreas development	3/158	54/28891	0.003244892	0.018452215	0.011853036	Clu/Anxa1/Sox4	3
GO:0032355	response to estradiol	3/158	54/28891	0.003244892	0.018452215	0.011853036	Sprr2f/Anxa1/Ctnnb1	3
GO:0045581	negative regulation of T cell differentiation	3/158	54/28891	0.003244892	0.018452215	0.011853036	Anxa1/Cd74/Prdx2	3
GO:0007159	leukocyte cell-cell adhesion	8/158	447/28891	0.003308058	0.018779419	0.01206322	Lgals3/Actb/Anxa1/Sox4/Cd74/Cd24a/Fut9/Prdx2	8
GO:0006606	protein import into nucleus	5/158	182/28891	0.003327235	0.018824256	0.012092022	Cdkn1a/Cfl1/Flna/Jup/Il33	5
GO:0032635	interleukin-6 production	5/158	182/28891	0.003327235	0.018824256	0.012092022	Lpl/Bsg/Il33/Cd74/Cd24a	5
GO:0030644	intracellular chloride ion homeostasis	2/158	16/28891	0.003391259	0.018898204	0.012139523	Spp1/Slc12a2	2
GO:0032488	Cdc42 protein signal transduction	2/158	16/28891	0.003391259	0.018898204	0.012139523	Apoe/Cdc42	2
GO:0051284	positive regulation of sequestering of calcium ion	2/158	16/28891	0.003391259	0.018898204	0.012139523	Gsto1/Calm1	2
GO:0055119	relaxation of cardiac muscle	2/158	16/28891	0.003391259	0.018898204	0.012139523	Gsn/Atp1b1	2
GO:0071801	regulation of podosome assembly	2/158	16/28891	0.003391259	0.018898204	0.012139523	Arpc2/Gsn	2
GO:0086103	G protein-coupled receptor signaling pathway involved in heart process	2/158	16/28891	0.003391259	0.018898204	0.012139523	Rac1/Cdc42	2
GO:1902043	positive regulation of extrinsic apoptotic signaling pathway via death domain receptors	2/158	16/28891	0.003391259	0.018898204	0.012139523	Timp3/Pea15a	2
GO:1902950	regulation of dendritic spine maintenance	2/158	16/28891	0.003391259	0.018898204	0.012139523	Cfl1/Apoe	2
GO:1905906	regulation of amyloid fibril formation	2/158	16/28891	0.003391259	0.018898204	0.012139523	Clu/Apoe	2
GO:0061462	protein localization to lysosome	3/158	55/28891	0.003418461	0.01901804	0.012216501	Clu/Rtn4/Hspa8	3
GO:0001890	placenta development	5/158	184/28891	0.003485873	0.019264645	0.012374912	Krt8/Krt19/Serpine1/Junb/Ctsl	5
GO:0010951	negative regulation of endopeptidase activity	5/158	184/28891	0.003485873	0.019264645	0.012374912	Sfn/Serpinb6b/Serpinb6a/Timp3/Serpine1	5
GO:0055088	lipid homeostasis	5/158	184/28891	0.003485873	0.019264645	0.012374912	Lpl/Rbp1/Apoe/Cd24a/Rtn4	5
GO:0071897	DNA biosynthetic process	5/158	184/28891	0.003485873	0.019264645	0.012374912	Cdkn1a/Cyp1b1/Ctnnb1/Vcp/Egf	5
GO:0032755	positive regulation of interleukin-6 production	4/158	113/28891	0.003493361	0.019274113	0.012380994	Lpl/Bsg/Il33/Cd74	4
GO:0016485	protein processing	6/158	266/28891	0.003527923	0.019400673	0.012462291	Myh9/Gsn/Serpine1/Mmp14/Adam10/S100a10	6
GO:0051896	regulation of phosphatidylinositol 3-kinase/protein kinase B signal transduction	6/158	266/28891	0.003527923	0.019400673	0.012462291	Rack1/Rac1/Rtn4/Egf/Cdc42/Lrp2	6
GO:0046889	positive regulation of lipid biosynthetic process	4/158	114/28891	0.003605221	0.019793138	0.012714396	Anxa1/Apoe/Cd74/Rdh10	4
GO:0034766	negative regulation of monoatomic ion transmembrane transport	4/158	115/28891	0.00371949	0.020353535	0.013074375	Gsto1/Ywhae/Calm1/Fxyd2	4
GO:0051702	biological process involved in interaction with symbiont	4/158	115/28891	0.00371949	0.020353535	0.013074375	Cfl1/Apoe/Hspa8/Cdc42	4
GO:0051170	import into nucleus	5/158	187/28891	0.003733924	0.020365752	0.013082223	Cdkn1a/Cfl1/Flna/Jup/Il33	5
GO:0071248	cellular response to metal ion	5/158	187/28891	0.003733924	0.020365752	0.013082223	Gsn/Clic4/Slc12a2/Junb/Iqgap1	5
GO:0006693	prostaglandin metabolic process	3/158	57/28891	0.003782399	0.020554102	0.013203212	Ptgs1/Anxa1/Cd74	3
GO:0051293	establishment of spindle localization	3/158	57/28891	0.003782399	0.020554102	0.013203212	Cfl1/Myh9/Map4	3
GO:0050714	positive regulation of protein secretion	5/158	188/28891	0.00381935	0.020554102	0.013203212	Myh9/Sox4/Bsg/Rac1/Arf1	5
GO:0010755	regulation of plasminogen activation	2/158	17/28891	0.003829674	0.020554102	0.013203212	Serpine1/S100a10	2
GO:0030002	intracellular monoatomic anion homeostasis	2/158	17/28891	0.003829674	0.020554102	0.013203212	Spp1/Slc12a2	2
GO:0061318	renal filtration cell differentiation	2/158	17/28891	0.003829674	0.020554102	0.013203212	Basp1/Cd24a	2
GO:0072112	podocyte differentiation	2/158	17/28891	0.003829674	0.020554102	0.013203212	Basp1/Cd24a	2
GO:0072311	glomerular epithelial cell differentiation	2/158	17/28891	0.003829674	0.020554102	0.013203212	Basp1/Cd24a	2
GO:2000696	regulation of epithelial cell differentiation involved in kidney development	2/158	17/28891	0.003829674	0.020554102	0.013203212	Cd24a/Ctnnb1	2
GO:0032642	regulation of chemokine production	4/158	116/28891	0.003836191	0.020554102	0.013203212	Lpl/Il33/Cd74/Cd24a	4

GO:0043266	regulation of potassium ion transport	4/158	116/28891	0.003836191	0.020554102	0.013203212	Ywhae/Flna/Fxyd2/Atp1b1	4
GO:0046942	carboxylic acid transport	7/158	362/28891	0.003888179	0.020799266	0.013360696	Anxa1/Rbp1/Apoe/Ucp2/Slc12a2/Slc6a19/Lrp2	7
GO:0009991	response to extracellular stimulus	8/158	460/28891	0.003929111	0.020917134	0.01343641	Slc34a1/Spp1/Cdkn1a/Lpl/Rbp1/Ywhag/Apoe/Itga6	8
GO:0002573	myeloid leukocyte differentiation	6/158	272/28891	0.003932331	0.020917134	0.01343641	Rbp1/Il33/Ucp2/Cd74/Ctnnb1/Junb	6
GO:0015844	monoamine transport	4/158	117/28891	0.00395535	0.020917134	0.01343641	Lgals3/Ptgs1/Actb/Rtn4	4
GO:0042177	negative regulation of protein catabolic process	4/158	117/28891	0.00395535	0.020917134	0.01343641	Anxa2/Flna/Timp3/Rpl11	4
GO:0006110	regulation of glycolytic process	3/158	58/28891	0.003972877	0.020917134	0.01343641	Ier3/Eif6/Nupr1	3
GO:0008631	intrinsic apoptotic signaling pathway in response to oxidative stress	3/158	58/28891	0.003972877	0.020917134	0.01343641	Cyp1b1/Rack1/Ctnnb1	3
GO:0042743	hydrogen peroxide metabolic process	3/158	58/28891	0.003972877	0.020917134	0.01343641	Gpx3/Txn1/Prdx2	3
GO:0051496	positive regulation of stress fiber assembly	3/158	58/28891	0.003972877	0.020917134	0.01343641	Rac1/Cdc42/S100a10	3
GO:0052372	modulation by symbiont of entry into host	3/158	58/28891	0.003972877	0.020917134	0.01343641	Gsn/Bsg/Cd74	3
GO:1903170	negative regulation of calcium ion transmembrane transport	3/158	58/28891	0.003972877	0.020917134	0.01343641	Gsto1/Ywhae/Calm1	3
GO:0045580	regulation of T cell differentiation	5/158	190/28891	0.003994389	0.020964262	0.013466684	Actb/Anxa1/Sox4/Cd74/Prdx2	5
GO:1905952	regulation of lipid localization	5/158	190/28891	0.003994389	0.020964262	0.013466684	Spp1/Anxa2/Lpl/Apoe/Egf	5
GO:0015849	organic acid transport	7/158	364/28891	0.004006201	0.020993247	0.013485303	Anxa1/Rbp1/Apoe/Ucp2/Slc12a2/Slc6a19/Lrp2	7
GO:0120034	positive regulation of plasma membrane bounded cell projection assembly	4/158	118/28891	0.004076989	0.021330706	0.013702074	Cfl1/Arpc2/Rac1/Cdc42	4
GO:0006631	fatty acid metabolic process	8/158	463/28891	0.004084455	0.021336324	0.013705683	Ptgs1/Acsm2/Cyp1b1/Anxa1/Lpl/Cd74/Eif6/Mgst3	8
GO:0001825	blastocyst formation	3/158	59/28891	0.004169096	0.021744443	0.013967844	Ctr9/Rtn4/Junb	3
GO:0051251	positive regulation of lymphocyte activation	7/158	367/28891	0.00418831	0.021810574	0.014010324	Cdkn1a/Actb/Anxa1/Sox4/Cd74/Cd24a/Mmp14	7
GO:0051897	positive regulation of phosphatidylinositol 3-kinase/protein kinase B signal transduction	5/158	193/28891	0.004267605	0.02211411	0.014205305	Rac1/Rtn4/Egf/Cdc42/Lrp2	5
GO:0002830	positive regulation of type 2 immune response	2/158	18/28891	0.004292973	0.02211411	0.014205305	Il33/Cd74	2
GO:0060572	morphogenesis of an epithelial bud	2/158	18/28891	0.004292973	0.02211411	0.014205305	Rdh10/Ctnnb1	2
GO:0061042	vascular wound healing	2/158	18/28891	0.004292973	0.02211411	0.014205305	Serpine1/Slc12a2	2
GO:0071243	cellular response to arsenic-containing substance	2/158	18/28891	0.004292973	0.02211411	0.014205305	Gsto1/Vcp	2
GO:1900221	regulation of amyloid-beta clearance	2/158	18/28891	0.004292973	0.02211411	0.014205305	Clu/Apoe	2
GO:1901881	positive regulation of protein depolymerization	2/158	18/28891	0.004292973	0.02211411	0.014205305	Cfl1/Dstn	2
GO:0021987	cerebral cortex development	4/158	120/28891	0.004327808	0.022259204	0.014298508	Ywhae/Rac1/Rtn4/Ctnnb1	4
GO:0006826	iron ion transport	3/158	60/28891	0.004371109	0.022344199	0.014353106	Lcn2/Cp/Fth1	3
GO:0086001	cardiac muscle cell action potential	3/158	60/28891	0.004371109	0.022344199	0.014353106	Calm1/Flna/Jup	3
GO:0097237	cellular response to toxic substance	3/158	60/28891	0.004371109	0.022344199	0.014353106	Gpx3/Txn1/Prdx2	3
GO:1902622	regulation of neutrophil migration	3/158	60/28891	0.004371109	0.022344199	0.014353106	Cd74/Rac1/Rtn4	3
GO:0010812	negative regulation of cell-substrate adhesion	3/158	61/28891	0.004578964	0.023314367	0.014976307	Serpine1/Mmp14/Col1a1	3
GO:0030199	collagen fibril organization	3/158	61/28891	0.004578964	0.023314367	0.014976307	Cyp1b1/Anxa2/Col1a1	3
GO:0008585	female gonad development	4/158	122/28891	0.004588837	0.023314367	0.014976307	Myh9/Rac1/Serpine1/Nupr1	4
GO:0071346	cellular response to type II interferon	4/158	122/28891	0.004588837	0.023314367	0.014976307	Capg/Calm1/Gsn/Cdc42	4
GO:0048193	Golgi vesicle transport	6/158	281/28891	0.004602074	0.023346082	0.014996679	Krt18/Rack1/Rab10/Vcp/Sptbn1/Arf1	6
GO:0043405	regulation of MAP kinase activity	5/158	197/28891	0.00465231	0.023565116	0.015137379	Apoe/Cd24a/Uchl1/Egf/Iqgap1	5
GO:0051101	regulation of DNA binding	4/158	123/28891	0.00472324	0.023854346	0.01532317	Calm1/Ctnnb1/Egf/Txn1	4
GO:0001894	tissue homeostasis	6/158	283/28891	0.004761658	0.023854346	0.01532317	Spp1/Ptgs1/Bsg/Rac1/Ctnnb1/Slc12a2	6
GO:0032412	regulation of monoatomic ion transmembrane transporter activity	6/158	283/28891	0.004761658	0.023854346	0.01532317	Gsto1/Cfl1/Ywhae/Calm1/Fxyd2/Atp1b1	6
GO:0060249	anatomical structure homeostasis	6/158	283/28891	0.004761658	0.023854346	0.01532317	Spp1/Ptgs1/Bsg/Rac1/Ctnnb1/Slc12a2	6
GO:0002138	retinoic acid biosynthetic process	2/158	19/28891	0.004780874	0.023854346	0.01532317	Rbp1/Rdh10	2
GO:0016102	diterpenoid biosynthetic process	2/158	19/28891	0.004780874	0.023854346	0.01532317	Rbp1/Rdh10	2
GO:0042574	retinal metabolic process	2/158	19/28891	0.004780874	0.023854346	0.01532317	Cyp1b1/Rdh10	2
GO:0072010	glomerular epithelium development	2/158	19/28891	0.004780874	0.023854346	0.01532317	Basp1/Cd24a	2
GO:0090083	regulation of inclusion body assembly	2/158	19/28891	0.004780874	0.023854346	0.01532317	Clu/Apoe	2
GO:0090280	positive regulation of calcium ion import	2/158	19/28891	0.004780874	0.023854346	0.01532317	Lgals3/Serpine1	2
GO:0051653	spindle localization	3/158	62/28891	0.00479271	0.023877709	0.015338177	Cfl1/Myh9/Map4	3
GO:2000106	regulation of leukocyte apoptotic process	4/158	124/28891	0.004860264	0.024178185	0.015531192	Lgals3/Anxa1/Cd74/Cd24a	4
GO:0045582	positive regulation of T cell differentiation	4/158	125/28891	0.004999934	0.024750542	0.015898854	Actb/Anxa1/Sox4/Cd74	4
GO:0046545	development of primary female sexual characteristics	4/158	125/28891	0.004999934	0.024750542	0.015898854	Myh9/Rac1/Serpine1/Nupr1	4
GO:0050670	regulation of lymphocyte proliferation	6/158	286/28891	0.005008626	0.024750542	0.015898854	Lgals3/Cdkn1a/Anxa1/Cd74/Cd24a/Ctnnb1	6
GO:0045620	negative regulation of lymphocyte differentiation	3/158	63/28891	0.005012393	0.024750542	0.015898854	Anxa1/Cd74/Prdx2	3
GO:2001238	positive regulation of extrinsic apoptotic signaling pathway	3/158	63/28891	0.005012393	0.024750542	0.015898854	Tnfrsf12a/Timp3/Pea15a	3
GO:0032602	chemokine production	4/158	126/28891	0.005142273	0.025316973	0.016262708	Lpl/Il33/Cd74/Cd24a	4
GO:1905954	positive regulation of lipid localization	4/158	126/28891	0.005142273	0.025316973	0.016262708	Spp1/Anxa2/Lpl/Apoe	4
GO:1902075	cellular response to salt	5/158	202/28891	0.00516712	0.025401835	0.01631722	Clic4/Ly6a/Slc12a2/Junb/Iqgap1	5
GO:0043616	keratinocyte proliferation	3/158	64/28891	0.005238058	0.025712704	0.016516912	Sfn/Cdkn1a/Ctsl	3
GO:0046651	lymphocyte proliferation	7/158	383/28891	0.005267671	0.02571814	0.016520403	Lgals3/Cdkn1a/Anxa1/Cd74/Cd24a/Ctnnb1/Prdx2	7
GO:0007052	mitotic spindle organization	4/158	127/28891	0.005287303	0.02571814	0.016520403	Flna/Map4/Vcp/Rab11a	4
GO:0035633	maintenance of blood-brain barrier	2/158	20/28891	0.005293098	0.02571814	0.016520403	Ptgs1/Slc12a2	2
GO:0036120	cellular response to platelet-derived growth factor stimulus	2/158	20/28891	0.005293098	0.02571814	0.016520403	Arpc2/Iqgap1	2
GO:0046827	positive regulation of protein export from nucleus	2/158	20/28891	0.005293098	0.02571814	0.016520403	Sfn/Ywhae	2
GO:0060314	regulation of ryanodine-sensitive calcium-release channel activity	2/158	20/28891	0.005293098	0.02571814	0.016520403	Gsto1/Calm1	2
GO:1902004	positive regulation of amyloid-beta formation	2/158	20/28891	0.005293098	0.02571814	0.016520403	Clu/Apoe	2
GO:0001503	ossification	8/158	484/28891	0.005308835	0.025719725	0.016521421	Spp1/Pdlim7/Mgp/Clic1/Ctnnb1/Mmp14/Col1a1/Junb	8
GO:0006091	generation of precursor metabolites and energy	8/158	484/28891	0.005308835	0.025719725	0.016521421	Taldo1/Pkm/Ier3/Ucp2/Eif6/Vcp/Nupr1/Idh1	8
GO:0032944	regulation of mononuclear cell proliferation	6/158	290/28891	0.005352404	0.025893225	0.016632872	Lgals3/Cdkn1a/Anxa1/Cd74/Cd24a/Ctnnb1	6
GO:0009749	response to glucose	5/158	204/28891	0.005383915	0.026007972	0.016706581	Myh9/Rack1/Sox4/Ucp2/Rac1	5
GO:0007041	lysosomal transport	4/158	128/28891	0.005435046	0.026179198	0.01681657	Clu/Hspa8/Vcp/Arf1	4
GO:2000278	regulation of DNA biosynthetic process	4/158	128/28891	0.005435046	0.026179198	0.01681657	Cdkn1a/Cyp1b1/Ctnnb1/Egf	4
GO:0071260	cellular response to mechanical stimulus	3/158	65/28891	0.005469751	0.026270546	0.016875249	Rac1/Ctnnb1/Col1a1	3
GO:0071622	regulation of granulocyte chemotaxis	3/158	65/28891	0.005469751	0.026270546	0.016875249	S100a14/Cd74/Rac1	3
GO:0006805	xenobiotic metabolic process	4/158	129/28891	0.005585525	0.026749617	0.017182987	Gsto1/Gsta4/Cyp1b1/Fmo5	4
GO:0032414	positive regulation of ion transmembrane transporter activity	4/158	129/28891	0.005585525	0.026749617	0.017182987	Gsto1/Cfl1/Calm1/Atp1b1	4
GO:0002695	negative regulation of leukocyte activation	5/158	206/28891	0.005607069	0.026814323	0.017224551	Lgals3/Anxa1/Cd74/Cd24a/Prdx2	5
GO:0048732	gland development	8/158	489/28891	0.005637891	0.026923147	0.017294456	Anxa1/Elf3/Pkm/Rtn4/Ctnnb1/Slc12a2/Egf/Prdx2	8
GO:0061077	chaperone-mediated protein folding	3/158	66/28891	0.005707514	0.027208517	0.017477767	Clu/Cd74/Hspa8	3

GO:0009746	response to hexose	5/158	207/28891	0.005721061	0.027208517	0.017477767	Myh9/Rack1/Sox4/Ucp2/Rac1	5
GO:0032943	mononuclear cell proliferation	7/158	389/28891	0.005722103	0.027208517	0.017477767	Lgals3/Cdkn1a/Anxa1/Cd74/Cd24a/Ctnnb1/Prdx2	7
GO:0030048	actin filament-based movement	4/158	130/28891	0.005738762	0.02721021	0.017478855	Myh9/Gsn/Flna/Jup	4
GO:0071675	regulation of mononuclear cell migration	4/158	130/28891	0.005738762	0.02721021	0.017478855	Lgals3/S100a14/Serpine1/Adam10	4
GO:0000028	ribosomal small subunit assembly	2/158	21/28891	0.005829368	0.027363534	0.017577345	Rpsa/Rps5	2
GO:0034377	plasma lipoprotein particle assembly	2/158	21/28891	0.005829368	0.027363534	0.017577345	Apoe/Arf1	2
GO:0046931	pore complex assembly	2/158	21/28891	0.005829368	0.027363534	0.017577345	Rtn4/Adam10	2
GO:0071786	endoplasmic reticulum tubular network organization	2/158	21/28891	0.005829368	0.027363534	0.017577345	Rtn4/Rab10	2
GO:1990182	exosomal secretion	2/158	21/28891	0.005829368	0.027363534	0.017577345	Sdc1/Rab11a	2
GO:0006109	regulation of carbohydrate metabolic process	5/158	208/28891	0.00583668	0.027363534	0.017577345	Myh9/Ier3/Eif6/Nupr1/Egf	5
GO:0007259	receptor signaling pathway via JAK-STAT	5/158	208/28891	0.00583668	0.027363534	0.017577345	Cyp1b1/Calm1/Ctr9/Rac1/Egf	5
GO:0034284	response to monosaccharide	5/158	208/28891	0.00583668	0.027363534	0.017577345	Myh9/Rack1/Sox4/Ucp2/Rac1	5
GO:0002040	sprouting angiogenesis	4/158	131/28891	0.005894779	0.027597156	0.017727416	Anxa1/Pkm/Rtn4/Cdc42	4
GO:0071470	cellular response to osmotic stress	3/158	67/28891	0.005951388	0.027718603	0.017805428	Serpinb6b/Serpinb6a/Micu1	3
GO:1901016	regulation of potassium ion transmembrane transporter activity	3/158	67/28891	0.005951388	0.027718603	0.017805428	Ywhae/Fxyd2/Atp1b1	3
GO:1905517	macrophage migration	3/158	67/28891	0.005951388	0.027718603	0.017805428	Lgals3/Rtn4/Mmp14	3
GO:0022408	negative regulation of cell-cell adhesion	5/158	209/28891	0.005953936	0.027718603	0.017805428	Lgals3/Anxa1/Cd74/Cd24a/Prdx2	5
GO:0010212	response to ionizing radiation	4/158	132/28891	0.006053598	0.028104185	0.018053112	Cdkn1a/Ccnd2/Anxa1/Lrp2	4
GO:0042476	odontogenesis	4/158	132/28891	0.006053598	0.028104185	0.018053112	Ctnnb1/Serpine1/Itga6/Col1a1	4
GO:1990138	neuron projection extension	5/158	211/28891	0.006193412	0.028591605	0.018366213	Tnfrsf12a/Apoe/Rtn4/Ctnnb1/Iqgap1	5
GO:0009117	nucleotide metabolic process	8/158	497/28891	0.006196511	0.028591605	0.018366213	Acsm2/Taldo1/Fmo5/Hspa8/Vcp/Ldhb/Atp1b1/Idh1	8
GO:0022029	telencephalon cell migration	3/158	68/28891	0.006201415	0.028591605	0.018366213	Rac1/Rtn4/Ctnnb1	3
GO:0030330	DNA damage response, signal transduction by p53 class mediator	3/158	68/28891	0.006201415	0.028591605	0.018366213	Cdkn1a/Sox4/Cd74	3
GO:0043903	regulation of biological process involved in symbiotic interaction	3/158	68/28891	0.006201415	0.028591605	0.018366213	Gsn/Bsg/Cd74	3
GO:1902414	protein localization to cell junction	4/158	133/28891	0.006215239	0.028615819	0.018381768	Actb/Flna/Adam10/Rab11a	4
GO:0042063	gliogenesis	7/158	397/28891	0.006372956	0.028938735	0.018589197	Clu/Anxa1/Sox4/Il33/Rtn4/Ctnnb1/Lrp2	7
GO:0001767	establishment of lymphocyte polarity	2/158	22/28891	0.006389409	0.028938735	0.018589197	Myh9/Gsn	2
GO:0001768	establishment of T cell polarity	2/158	22/28891	0.006389409	0.028938735	0.018589197	Myh9/Gsn	2
GO:0010224	response to UV-B	2/158	22/28891	0.006389409	0.028938735	0.018589197	Ivl/Cdkn1a	2
GO:0030220	platelet formation	2/158	22/28891	0.006389409	0.028938735	0.018589197	Myh9/Actn1	2
GO:0042730	fibrinolysis	2/158	22/28891	0.006389409	0.028938735	0.018589197	Anxa2/Serpine1	2
GO:0044851	hair cycle phase	2/158	22/28891	0.006389409	0.028938735	0.018589197	Ctnnb1/Ctsl	2
GO:0045056	transcytosis	2/158	22/28891	0.006389409	0.028938735	0.018589197	Lrp2/Rab11a	2
GO:0045820	negative regulation of glycolytic process	2/158	22/28891	0.006389409	0.028938735	0.018589197	Ier3/Nupr1	2
GO:0097734	extracellular exosome biogenesis	2/158	22/28891	0.006389409	0.028938735	0.018589197	Sdc1/Rab11a	2
GO:0098719	sodium ion import across plasma membrane	2/158	22/28891	0.006389409	0.028938735	0.018589197	Slc34a1/Slc12a2	2
GO:2000114	regulation of establishment of cell polarity	2/158	22/28891	0.006389409	0.028938735	0.018589197	Cfl1/Gsn	2
GO:1902107	positive regulation of leukocyte differentiation	5/158	213/28891	0.006439584	0.029087051	0.01868447	Actb/Anxa1/Sox4/Cd74/Mmp14	5
GO:1903708	positive regulation of hemopoiesis	5/158	213/28891	0.006439584	0.029087051	0.01868447	Actb/Anxa1/Sox4/Cd74/Mmp14	5
GO:0001523	retinoid metabolic process	3/158	69/28891	0.006457632	0.029089847	0.018686266	Cyp1b1/Rbp1/Rdh10	3
GO:0006611	protein export from nucleus	3/158	69/28891	0.006457632	0.029089847	0.018686266	Sfn/Ywhae/Txn1	3
GO:0006605	protein targeting	6/158	303/28891	0.006589522	0.029643967	0.019042212	Clu/Ywhae/Arpc2/Ywhag/Hspa8/Rpl11	6
GO:0006364	rRNA processing	5/158	215/28891	0.006692548	0.030066926	0.019313906	Rpl14/Eif6/Las1l/Rpl7/Rpl11	5
GO:0032411	positive regulation of transporter activity	4/158	136/28891	0.006717317	0.03013764	0.01935933	Gsto1/Cfl1/Calm1/Atp1b1	4
GO:0097696	receptor signaling pathway via STAT	5/158	216/28891	0.006821607	0.030564462	0.019633506	Cyp1b1/Calm1/Ctr9/Rac1/Egf	5
GO:0003158	endothelium development	4/158	137/28891	0.006890464	0.030655175	0.019691776	Lcn2/Bsg/Clic4/Ctnnb1	4
GO:0000303	response to superoxide	2/158	23/28891	0.006972948	0.030655175	0.019691776	Ucp2/Prdx2	2
GO:0010818	T cell chemotaxis	2/158	23/28891	0.006972948	0.030655175	0.019691776	Slc12a2/Adam10	2
GO:0010880	regulation of release of sequestered calcium ion into cytosol by sarcoplasmic reticulum	2/158	23/28891	0.006972948	0.030655175	0.019691776	Gsto1/Calm1	2
GO:0031579	membrane raft organization	2/158	23/28891	0.006972948	0.030655175	0.019691776	Gsn/S100a10	2
GO:0036119	response to platelet-derived growth factor	2/158	23/28891	0.006972948	0.030655175	0.019691776	Arpc2/Iqgap1	2
GO:0051043	regulation of membrane protein ectodomain proteolysis	2/158	23/28891	0.006972948	0.030655175	0.019691776	Apoe/Timp3	2
GO:0055062	phosphate ion homeostasis	2/158	23/28891	0.006972948	0.030655175	0.019691776	Slc34a1/Spp1	2
GO:0071800	podosome assembly	2/158	23/28891	0.006972948	0.030655175	0.019691776	Arpc2/Gsn	2
GO:0016101	diterpenoid metabolic process	3/158	71/28891	0.006988792	0.030655175	0.019691776	Cyp1b1/Rbp1/Rdh10	3
GO:0021885	forebrain cell migration	3/158	71/28891	0.006988792	0.030655175	0.019691776	Rac1/Rtn4/Ctnnb1	3
GO:0032371	regulation of sterol transport	3/158	71/28891	0.006988792	0.030655175	0.019691776	Anxa2/Apoe/Egf	3
GO:0032374	regulation of cholesterol transport	3/158	71/28891	0.006988792	0.030655175	0.019691776	Anxa2/Apoe/Egf	3
GO:0098754	detoxification	3/158	71/28891	0.006988792	0.030655175	0.019691776	Gpx3/Txn1/Prdx2	3
GO:0098900	regulation of action potential	3/158	71/28891	0.006988792	0.030655175	0.019691776	Calm1/Flna/Jup	3
GO:1902305	regulation of sodium ion transmembrane transport	3/158	71/28891	0.006988792	0.030655175	0.019691776	Fxyd2/Arf1/Atp1b1	3
GO:0000086	G2/M transition of mitotic cell cycle	4/158	138/28891	0.007066539	0.030914404	0.019858296	Cdkn1a/Calm1/Ier3/Rab11a	4
GO:0046425	regulation of receptor signaling pathway via JAK-STAT	4/158	138/28891	0.007066539	0.030914404	0.019858296	Cyp1b1/Calm1/Rac1/Egf	4
GO:0048638	regulation of developmental growth	7/158	405/28891	0.007077533	0.030914404	0.019858296	Cdkn1a/Cfl1/Ccnd2/Tnfrsf12a/Apoe/Rtn4/Serpine1	7
GO:0001101	response to acid chemical	5/158	218/28891	0.007084937	0.030914404	0.019858296	Krt8/Cfl1/Anxa2/Col1a1/S100a10	5
GO:0031638	zymogen activation	3/158	72/28891	0.007263807	0.031489074	0.020227443	Serpine1/Mmp14/S100a10	3
GO:0043470	regulation of carbohydrate catabolic process	3/158	72/28891	0.007263807	0.031489074	0.020227443	Ier3/Eif6/Nupr1	3
GO:0055117	regulation of cardiac muscle contraction	3/158	72/28891	0.007263807	0.031489074	0.020227443	Calm1/Jup/Atp1b1	3
GO:0060425	lung morphogenesis	3/158	72/28891	0.007263807	0.031489074	0.020227443	Rdh10/Ctnnb1/Cdc42	3
GO:0086003	cardiac muscle cell contraction	3/158	72/28891	0.007263807	0.031489074	0.020227443	Gsn/Flna/Jup	3
GO:0030888	regulation of B cell proliferation	3/158	73/28891	0.007545158	0.032437293	0.020836544	Cdkn1a/Cd74/Cd24a	3
GO:1905330	regulation of morphogenesis of an epithelium	3/158	73/28891	0.007545158	0.032437293	0.020836544	Lcn2/Ctnnb1/Egf	3
GO:0000305	response to oxygen radical	2/158	24/28891	0.007579715	0.032437293	0.020836544	Ucp2/Prdx2	2
GO:0010560	positive regulation of glycoprotein biosynthetic process	2/158	24/28891	0.007579715	0.032437293	0.020836544	Il33/Ctnnb1	2
GO:0016114	terpenoid biosynthetic process	2/158	24/28891	0.007579715	0.032437293	0.020836544	Rbp1/Rdh10	2
GO:0036344	platelet morphogenesis	2/158	24/28891	0.007579715	0.032437293	0.020836544	Myh9/Actn1	2
GO:0048485	sympathetic nervous system development	2/158	24/28891	0.007579715	0.032437293	0.020836544	Sox4/Ctnnb1	2
GO:0060055	angiogenesis involved in wound healing	2/158	24/28891	0.007579715	0.032437293	0.020836544	Serpine1/Slc12a2	2

GO:0140112	extracellular vesicle biogenesis	2/158	24/28891	0.007579715	0.032437293	0.020836544	Sdc1/Rab11a	2
GO:0150105	protein localization to cell-cell junction	2/158	24/28891	0.007579715	0.032437293	0.020836544	Actb/Flna	2
GO:0001776	leukocyte homeostasis	4/158	141/28891	0.007612537	0.032536044	0.020899978	Anxa1/Cd74/Cd24a/Prdx2	4
GO:0009743	response to carbohydrate	5/158	223/28891	0.00777421	0.033184544	0.021316551	Myh9/Rack1/Sox4/Ucp2/Rac1	5
GO:0030177	positive regulation of Wnt signaling pathway	4/158	142/28891	0.007800528	0.03325436	0.021361399	Sox4/Vcp/Egf/Col1a1	4
GO:0048662	negative regulation of smooth muscle cell proliferation	3/158	74/28891	0.007832878	0.033349677	0.021422627	Cdkn1a/Apoe/Timp3	3
GO:0014013	regulation of gliogenesis	4/158	143/28891	0.007991548	0.033981894	0.02182874	Il33/Rtn4/Ctnnb1/Lrp2	4
GO:0032677	regulation of interleukin-8 production	3/158	75/28891	0.008127	0.034470046	0.022142311	Anxa1/Cd74/Serpine1	3
GO:0043113	receptor clustering	3/158	75/28891	0.008127	0.034470046	0.022142311	Gsn/Flna/Apoe	3
GO:0043491	phosphatidylinositol 3-kinase/protein kinase B signal transduction	6/158	317/28891	0.008141288	0.034486827	0.022153091	Rack1/Rac1/Rtn4/Egf/Cdc42/Lrp2	6
GO:0001829	trophectodermal cell differentiation	2/158	25/28891	0.008209441	0.03451281	0.022169781	Ctr9/Junb	2
GO:0046697	decidualization	2/158	25/28891	0.008209441	0.03451281	0.022169781	Junb/Ctsl	2
GO:0051788	response to misfolded protein	2/158	25/28891	0.008209441	0.03451281	0.022169781	Clu/Vcp	2
GO:1902307	positive regulation of sodium ion transmembrane transport	2/158	25/28891	0.008209441	0.03451281	0.022169781	Arf1/Atp1b1	2
GO:1902410	mitotic cytokinetic process	2/158	25/28891	0.008209441	0.03451281	0.022169781	Arf1/Iqgap1	2
GO:0017157	regulation of exocytosis	5/158	226/28891	0.008209458	0.03451281	0.022169781	Anxa2/Anxa1/Sdc1/S100a10/Arf1	5
GO:0032637	interleukin-8 production	3/158	76/28891	0.008427555	0.035252102	0.022644676	Anxa1/Cd74/Serpine1	3
GO:0048645	animal organ formation	3/158	76/28891	0.008427555	0.035252102	0.022644676	Rdh10/Ctnnb1/Lrp2	3
GO:0051926	negative regulation of calcium ion transport	3/158	76/28891	0.008427555	0.035252102	0.022644676	Gsto1/Ywhae/Calm1	3
GO:0060997	dendritic spine morphogenesis	3/158	76/28891	0.008427555	0.035252102	0.022644676	Cfl1/Cdc42/Adam10	3
GO:1904892	regulation of receptor signaling pathway via STAT	4/158	146/28891	0.008582972	0.035857271	0.023033415	Cyp1b1/Calm1/Rac1/Egf	4
GO:0045669	positive regulation of osteoblast differentiation	3/158	77/28891	0.008734572	0.036399502	0.023381724	Pdlim7/Clic1/Ctnnb1	3
GO:2000573	positive regulation of DNA biosynthetic process	3/158	77/28891	0.008734572	0.036399502	0.023381724	Cyp1b1/Ctnnb1/Egf	3
GO:0051592	response to calcium ion	4/158	147/28891	0.008786298	0.036429671	0.023401103	Calm1/Clic4/Junb/Iqgap1	4
GO:0000027	ribosomal large subunit assembly	2/158	26/28891	0.008861861	0.036429671	0.023401103	Rplp0/Rpl11	2
GO:0014808	release of sequestered calcium ion into cytosol by sarcoplasmic reticulum	2/158	26/28891	0.008861861	0.036429671	0.023401103	Gsto1/Calm1	2
GO:0030194	positive regulation of blood coagulation	2/158	26/28891	0.008861861	0.036429671	0.023401103	Serpine1/Prdx2	2
GO:0045838	positive regulation of membrane potential	2/158	26/28891	0.008861861	0.036429671	0.023401103	Slc34a1/Vcp	2
GO:0065005	protein-lipid complex assembly	2/158	26/28891	0.008861861	0.036429671	0.023401103	Apoe/Arf1	2
GO:1900048	positive regulation of hemostasis	2/158	26/28891	0.008861861	0.036429671	0.023401103	Serpine1/Prdx2	2
GO:1902176	negative regulation of oxidative stress-induced intrinsic apoptotic signaling pathway	2/158	26/28891	0.008861861	0.036429671	0.023401103	Rack1/Ctnnb1	2
GO:1902230	negative regulation of intrinsic apoptotic signaling pathway in response to DNA damage	2/158	26/28891	0.008861861	0.036429671	0.023401103	Clu/Cd74	2
GO:1902993	positive regulation of amyloid precursor protein catabolic process	2/158	26/28891	0.008861861	0.036429671	0.023401103	Clu/Apoe	2
GO:1903020	positive regulation of glycoprotein metabolic process	2/158	26/28891	0.008861861	0.036429671	0.023401103	Il33/Ctnnb1	2
GO:0030111	regulation of Wnt signaling pathway	6/158	323/28891	0.008880867	0.036462897	0.023422447	Sox4/Apoe/Ctnnb1/Vcp/Egf/Col1a1	6
GO:0048706	embryonic skeletal system development	4/158	148/28891	0.008992749	0.036831651	0.023659321	Rdh10/Ctnnb1/Mmp14/Col1a1	4
GO:0050864	regulation of B cell activation	4/158	148/28891	0.008992749	0.036831651	0.023659321	Cdkn1a/Cd74/Cd24a/Mmp14	4
GO:0006721	terpenoid metabolic process	3/158	78/28891	0.009048081	0.037012861	0.023775724	Cyp1b1/Rbp1/Rdh10	3
GO:0033674	positive regulation of kinase activity	7/158	426/28891	0.009201568	0.037594655	0.024149447	Clu/Cdkn1a/Ccnd2/Cd74/Cd24a/Egf/Iqgap1	7
GO:0031667	response to nutrient levels	7/158	427/28891	0.009313182	0.038004158	0.024412498	Slc34a1/Spp1/Cdkn1a/Lpl/Rbp1/Ywhag/Apoe	7
GO:0007492	endoderm development	3/158	79/28891	0.009368109	0.038135059	0.024496583	Myh9/Ctr9/Ctnnb1	3
GO:0007584	response to nutrient	3/158	79/28891	0.009368109	0.038135059	0.024496583	Spp1/Lpl/Rbp1	3
GO:0002065	columnar/cuboidal epithelial cell differentiation	4/158	150/28891	0.009415095	0.038169713	0.024518844	Tubb5/Sox4/Ctnnb1/Serpine1	4
GO:0002082	regulation of oxidative phosphorylation	2/158	27/28891	0.009536711	0.038169713	0.024518844	Vcp/Nupr1	2
GO:0002523	leukocyte migration involved in inflammatory response	2/158	27/28891	0.009536711	0.038169713	0.024518844	Cd24a/Rtn4	2
GO:0010575	positive regulation of vascular endothelial growth factor production	2/158	27/28891	0.009536711	0.038169713	0.024518844	Cyp1b1/Bsg	2
GO:0010839	negative regulation of keratinocyte proliferation	2/158	27/28891	0.009536711	0.038169713	0.024518844	Sfn/Ctsl	2
GO:0031639	plasminogen activation	2/158	27/28891	0.009536711	0.038169713	0.024518844	Serpine1/S100a10	2
GO:0032878	regulation of establishment or maintenance of cell polarity	2/158	27/28891	0.009536711	0.038169713	0.024518844	Cfl1/Gsn	2
GO:0034368	protein-lipid complex remodeling	2/158	27/28891	0.009536711	0.038169713	0.024518844	Lpl/Apoe	2
GO:0034369	plasma lipoprotein particle remodeling	2/158	27/28891	0.009536711	0.038169713	0.024518844	Lpl/Apoe	2
GO:0046685	response to arsenic-containing substance	2/158	27/28891	0.009536711	0.038169713	0.024518844	Gsto1/Vcp	2
GO:0098901	regulation of cardiac muscle cell action potential	2/158	27/28891	0.009536711	0.038169713	0.024518844	Calm1/Jup	2
GO:1901857	positive regulation of cellular respiration	2/158	27/28891	0.009536711	0.038169713	0.024518844	Vcp/Nupr1	2
GO:1903514	release of sequestered calcium ion into cytosol by endoplasmic reticulum	2/158	27/28891	0.009536711	0.038169713	0.024518844	Gsto1/Calm1	2
GO:1904030	negative regulation of cyclin-dependent protein kinase activity	2/158	27/28891	0.009536711	0.038169713	0.024518844	Cdkn1a/Actb	2
GO:0050679	positive regulation of epithelial cell proliferation	5/158	235/28891	0.009616247	0.038441954	0.024693721	Ccnd2/Rtn4/Ctnnb1/Egf/Cdc42	5
GO:0051224	negative regulation of protein transport	4/158	151/28891	0.009631028	0.038454988	0.024702094	Anxa1/Apoe/Ucp2/Txn1	4
GO:0042742	defense response to bacterium	7/158	430/28891	0.009653986	0.038500602	0.024731395	Ivl/Wfdc2/Sprr2a3/Lcn2/Anxa3/Mmp7/Serpine1	7
GO:0050769	positive regulation of neurogenesis	6/158	329/28891	0.009667432	0.038508219	0.024736288	Tnfrsf12a/Il33/Apoe/Rtn4/Ctnnb1/Lrp2	6
GO:0055001	muscle cell development	5/158	236/28891	0.009782153	0.038918745	0.024999995	Krt8/Krt19/Myof/Actn1/Uchl1	5
GO:0045637	regulation of myeloid cell differentiation	5/158	237/28891	0.009950019	0.03953948	0.025398732	Ctr9/Rbp1/Cd74/Eif6/Ctnnb1	5
GO:0045453	bone resorption	3/158	81/28891	0.01002783	0.039754032	0.025536553	Spp1/Rac1/Ctnnb1	3
GO:0072665	protein localization to vacuole	3/158	81/28891	0.01002783	0.039754032	0.025536553	Clu/Rtn4/Hspa8	3
GO:0016197	endosomal transport	5/158	238/28891	0.010119855	0.040000218	0.025694694	Rab10/Vcp/Cdc42/Lrp2/Rab11a	5
GO:0008209	androgen metabolic process	2/158	28/28891	0.010233729	0.040000218	0.025694694	Spp1/Srd5a1	2
GO:0015740	C4-dicarboxylate transport	2/158	28/28891	0.010233729	0.040000218	0.025694694	Ucp2/Slc12a2	2
GO:0021801	cerebral cortex radial glia-guided migration	2/158	28/28891	0.010233729	0.040000218	0.025694694	Rtn4/Ctnnb1	2
GO:0022030	telencephalon glial cell migration	2/158	28/28891	0.010233729	0.040000218	0.025694694	Rtn4/Ctnnb1	2
GO:0035020	regulation of Rac protein signal transduction	2/158	28/28891	0.010233729	0.040000218	0.025694694	Rtn4/Cadm4	2
GO:0045723	positive regulation of fatty acid biosynthetic process	2/158	28/28891	0.010233729	0.040000218	0.025694694	Anxa1/Cd74	2
GO:0048820	hair follicle maturation	2/158	28/28891	0.010233729	0.040000218	0.025694694	Ctnnb1/Ctsl	2
GO:0090026	positive regulation of monocyte chemotaxis	2/158	28/28891	0.010233729	0.040000218	0.025694694	S100a14/Serpine1	2
GO:0097062	dendritic spine maintenance	2/158	28/28891	0.010233729	0.040000218	0.025694694	Cfl1/Apoe	2
GO:1903902	positive regulation of viral life cycle	2/158	28/28891	0.010233729	0.040000218	0.025694694	Bsg/Cd74	2
GO:2000647	negative regulation of stem cell proliferation	2/158	28/28891	0.010233729	0.040000218	0.025694694	Sfn/Tsc22d1	2
GO:0015711	organic anion transport	7/158	436/28891	0.010362864	0.040381511	0.025939623	Anxa1/Rbp1/Apoe/Ucp2/Slc12a2/Slc6a19/Lrp2	7
GO:0003208	cardiac ventricle morphogenesis	3/158	82/28891	0.010367572	0.040381511	0.025939623	Sox4/Ctnnb1/Lrp2	3

GO:0051149	positive regulation of muscle cell differentiation	3/158	82/28891	0.010367572	0.040381511	0.025939623	Tmsb4x/Eif5a/Mmp14	3
GO:1904950	negative regulation of establishment of protein localization	4/158	155/28891	0.010526897	0.04095429	0.026307555	Anxa1/Apoe/Ucp2/Txn1	4
GO:1904888	cranial skeletal system development	3/158	83/28891	0.010713934	0.041633425	0.026743807	Rdh10/Ctnnb1/Mmp14	3
GO:0044839	cell cycle G2/M phase transition	4/158	156/28891	0.010758985	0.041759876	0.026825035	Cdkn1a/Calm1/Ier3/Rab11a	4
GO:0046883	regulation of hormone secretion	6/158	337/28891	0.010791934	0.041839111	0.026875933	Spp1/Myh9/Anxa1/Sox4/Ucp2/Rac1	6
GO:0006622	protein targeting to lysosome	2/158	29/28891	0.010952655	0.042152148	0.027077016	Clu/Hspa8	2
GO:0034367	protein-containing complex remodeling	2/158	29/28891	0.010952655	0.042152148	0.027077016	Lpl/Apoe	2
GO:0050820	positive regulation of coagulation	2/158	29/28891	0.010952655	0.042152148	0.027077016	Serpine1/Prdx2	2
GO:0072567	chemokine (C-X-C motif) ligand 2 production	2/158	29/28891	0.010952655	0.042152148	0.027077016	Lpl/Cd74	2
GO:2000341	regulation of chemokine (C-X-C motif) ligand 2 production	2/158	29/28891	0.010952655	0.042152148	0.027077016	Lpl/Cd74	2
GO:0043271	negative regulation of monoatomic ion transport	4/158	157/28891	0.010994357	0.042152148	0.027077016	Gsto1/Ywhae/Calm1/Fxyd2	4
GO:0071887	leukocyte apoptotic process	4/158	157/28891	0.010994357	0.042152148	0.027077016	Lgals3/Anxa1/Cd74/Cd24a	4
GO:0030324	lung development	5/158	243/28891	0.010998958	0.042152148	0.027077016	Mgp/Rdh10/Ctnnb1/Cdc42/Mmp14	5
GO:0034249	negative regulation of amide metabolic process	5/158	243/28891	0.010998958	0.042152148	0.027077016	Clu/Rack1/Apoe/Eif6/Rtn4	5
GO:0050920	regulation of chemotaxis	5/158	243/28891	0.010998958	0.042152148	0.027077016	S100a14/Cd74/Rac1/Serpine1/Adam10	5
GO:1903828	negative regulation of protein localization	5/158	244/28891	0.011180837	0.042800038	0.027493197	Anxa1/Apoe/Ucp2/Vcp/Txn1	5
GO:0032368	regulation of lipid transport	4/158	158/28891	0.011233027	0.042950567	0.027589892	Spp1/Anxa2/Apoe/Egf	4
GO:0070228	regulation of lymphocyte apoptotic process	3/158	85/28891	0.011426608	0.043590877	0.028001204	Lgals3/Cd74/Cd24a	3
GO:1901796	regulation of signal transduction by p53 class mediator	3/158	85/28891	0.011426608	0.043590877	0.028001204	Sox4/Cd74/Rpl11	3
GO:0001933	negative regulation of protein phosphorylation	6/158	342/28891	0.011540154	0.04396392	0.028240833	Cdkn1a/Actb/Rack1/Apoe/Cadm4/Uchl1	6
GO:0030323	respiratory tube development	5/158	246/28891	0.011550736	0.04396392	0.028240833	Mgp/Rdh10/Ctnnb1/Cdc42/Mmp14	5
GO:0003012	muscle system process	7/158	446/28891	0.011627632	0.044206191	0.028396459	Ptgs1/Calm1/Gsn/Flna/Jup/Tpm4/Atp1b1	7
GO:0042744	hydrogen peroxide catabolic process	2/158	30/28891	0.011693233	0.04420709	0.028397036	Gpx3/Prdx2	2
GO:0060907	positive regulation of macrophage cytokine production	2/158	30/28891	0.011693233	0.04420709	0.028397036	Cd74/Rtn4	2
GO:0061298	retina vasculature development in camera-type eye	2/158	30/28891	0.011693233	0.04420709	0.028397036	Cyp1b1/Clic4	2
GO:1902042	negative regulation of extrinsic apoptotic signaling pathway via death domain receptors	2/158	30/28891	0.011693233	0.04420709	0.028397036	Serpine1/Pea15a	2
GO:1901990	regulation of mitotic cell cycle phase transition	6/158	343/28891	0.011694086	0.04420709	0.028397036	Cdkn1a/Ccnd2/Actb/Anxa1/Ier3/Rab11a	6
GO:1902850	microtubule cytoskeleton organization involved in mitosis	4/158	160/28891	0.011720331	0.044256182	0.028428571	Flna/Map4/Vcp/Rab11a	4
GO:0032722	positive regulation of chemokine production	3/158	86/28891	0.011792962	0.044480121	0.028572421	Lpl/Il33/Cd74	3
GO:0043467	regulation of generation of precursor metabolites and energy	4/158	161/28891	0.011968996	0.045093125	0.028966194	Ier3/Eif6/Vcp/Nupr1	4
GO:0016042	lipid catabolic process	6/158	345/28891	0.012006294	0.045182649	0.0290237	Spp1/Cyp1b1/Lpl/Srd5a1/Apoe/Idh1	6
GO:0019221	cytokine-mediated signaling pathway	7/158	450/28891	0.01216361	0.045578199	0.029277787	Krt8/Krt18/Klf6/Ctr9/Il33/Cd74/Cd24a	7
GO:0006809	nitric oxide biosynthetic process	3/158	87/28891	0.01216602	0.045578199	0.029277787	Clu/Cyp1b1/Rac1	3
GO:0014032	neural crest cell development	3/158	87/28891	0.01216602	0.045578199	0.029277787	Cfl1/Rdh10/Cdc42	3
GO:0034103	regulation of tissue remodeling	3/158	87/28891	0.01216602	0.045578199	0.029277787	Spp1/Klf6/Cd24a	3
GO:0098773	skin epidermis development	4/158	162/28891	0.012221025	0.045732937	0.029377186	Sfn/Cldn4/Ctnnb1/Ctsl	4
GO:0015874	norepinephrine transport	2/158	31/28891	0.012455207	0.046349478	0.029773229	Ptgs1/Actb	2
GO:0032801	receptor catabolic process	2/158	31/28891	0.012455207	0.046349478	0.029773229	Anxa2/Apoe	2
GO:0045454	cell redox homeostasis	2/158	31/28891	0.012455207	0.046349478	0.029773229	Txn1/Prdx2	2
GO:0060795	cell fate commitment involved in formation of primary germ layer	2/158	31/28891	0.012455207	0.046349478	0.029773229	Ctr9/Ctnnb1	2
GO:1905523	positive regulation of macrophage migration	2/158	31/28891	0.012455207	0.046349478	0.029773229	Rtn4/Mmp14	2
GO:0051147	regulation of muscle cell differentiation	4/158	163/28891	0.012476432	0.046376759	0.029790753	Ccnd2/Tmsb4x/Eif5a/Mmp14	4
GO:0003151	outflow tract morphogenesis	3/158	88/28891	0.012545801	0.046582742	0.02992307	Ctnnb1/Cdc42/Lrp2	3
GO:0009895	negative regulation of catabolic process	6/158	349/28891	0.012648271	0.04691103	0.03013395	Anxa2/Flna/Ier3/Timp3/Nupr1/Rpl11	6
GO:0016072	rRNA metabolic process	5/158	252/28891	0.012710299	0.046972606	0.030173504	Rpl14/Eif6/Las1l/Rpl7/Rpl11	5
GO:0044772	mitotic cell cycle phase transition	7/158	454/28891	0.012717237	0.046972606	0.030173504	Cdkn1a/Ccnd2/Actb/Calm1/Anxa1/Ier3/Rab11a	7
GO:0001837	epithelial to mesenchymal transition	4/158	164/28891	0.012735233	0.046972606	0.030173504	Flna/Rtn4/Ctnnb1/Col1a1	4
GO:0007613	memory	4/158	164/28891	0.012735233	0.046972606	0.030173504	Ptgs1/Ccnd2/Lcn2/Apoe	4
GO:0009266	response to temperature stimulus	4/158	164/28891	0.012735233	0.046972606	0.030173504	Myof/Ywhae/Ucp2/Vcp	4
GO:0034308	primary alcohol metabolic process	3/158	89/28891	0.012932324	0.047646905	0.030606649	Cyp1b1/Rbp1/Rdh10	3
GO:0007034	vacuolar transport	4/158	165/28891	0.012997444	0.047781351	0.030693012	Clu/Hspa8/Vcp/Arf1	4
GO:0030879	mammary gland development	4/158	165/28891	0.012997444	0.047781351	0.030693012	Elf3/Rtn4/Slc12a2/Egf	4
GO:0060977	coronary vasculature morphogenesis	2/158	32/28891	0.013238324	0.048506615	0.031158896	Ctnnb1/Lrp2	2
GO:0071354	cellular response to interleukin-6	2/158	32/28891	0.013238324	0.048506615	0.031158896	Cfl1/Ctr9	2
GO:0150077	regulation of neuroinflammatory response	2/158	32/28891	0.013238324	0.048506615	0.031158896	Nupr1/Cd200r3	2
GO:2000045	regulation of G1/S transition of mitotic cell cycle	4/158	166/28891	0.013263078	0.04850694	0.031159105	Cdkn1a/Ccnd2/Actb/Anxa1	4
GO:0042180	cellular ketone metabolic process	5/158	255/28891	0.013318622	0.04850694	0.031159105	Anxa1/Srd5a1/Cd74/Rdh10/Eif6	5
GO:0001738	morphogenesis of a polarized epithelium	3/158	90/28891	0.013325603	0.04850694	0.031159105	Actb/Rac1/Cdc42	3
GO:0006942	regulation of striated muscle contraction	3/158	90/28891	0.013325603	0.04850694	0.031159105	Calm1/Jup/Atp1b1	3
GO:0009408	response to heat	3/158	90/28891	0.013325603	0.04850694	0.031159105	Myof/Ywhae/Vcp	3
GO:0098661	inorganic anion transmembrane transport	3/158	90/28891	0.013325603	0.04850694	0.031159105	Slc34a1/Slc12a1/Slc12a2	3
GO:0009100	glycoprotein metabolic process	6/158	354/28891	0.013484222	0.048924274	0.031427185	Itm2b/Mgat4a/Il33/Ctnnb1/Fut9/Ctsl	6
GO:0022409	positive regulation of cell-cell adhesion	6/158	354/28891	0.013484222	0.048924274	0.031427185	Actb/Anxa1/Sox4/Cd74/Cd24a/Itga6	6
GO:0070588	calcium ion transmembrane transport	6/158	354/28891	0.013484222	0.048924274	0.031427185	Gsto1/Ywhae/Calm1/Flna/Micu1/Atp1b1	6
GO:0006937	regulation of muscle contraction	4/158	167/28891	0.013532151	0.049044865	0.031504648	Ptgs1/Calm1/Jup/Atp1b1	4
GO:0061178	regulation of insulin secretion involved in cellular response to glucose stimulus	3/158	91/28891	0.013725657	0.049638399	0.031885913	Myh9/Ucp2/Rac1	3
GO:0071277	cellular response to calcium ion	3/158	91/28891	0.013725657	0.049638399	0.031885913	Clic4/Junb/Iqgap1	3
GO:0072175	epithelial tube formation	4/158	168/28891	0.013804677	0.049870144	0.032034777	Cfl1/Ctnnb1/Egf/Lrp2	4
