# Supplementary material for: PPARγ promotes urothelial remodeling during urinary tract obstruction
Source: Exp Mol Med. 2025 May 1;57(5):950–63. doi: 10.1038/s12276-025-01441-0 (PMC12130184; doi:10.1038/s12276-025-01441-0)
Supplement: Supplementary file 5 — Supplementary Table 6 [file 12276_2025_1441_MOESM5_ESM.pdf]

Supplementary Table 6 - TFs

Rank	TF	Score	Library	Overlapping_Genes
1	IRF6	17	ARCHS4 Coexpression,3;Enrichr Queries,7;GTEx Coexpression,41	[illegible]
2	KLF5	17.2	Literature ChIP-seq,13;ARCHS4 Coexpression,14;Enrichr Queries,12;ReMap ChIP-seq,9;GTEx Coexpression,38	[illegible]
3	NFE2L2	20	Literature ChIP-seq,28;ARCHS4 Coexpression,9;Enrichr Queries,18;ReMap ChIP-seq,42;GTEx Coexpression,3	[illegible]
4	GRHL3	23	ARCHS4 Coexpression,2;Enrichr Queries,63;GTEx Coexpression,4	[illegible]
5	RARG	32.5	Literature ChIP-seq,8;ARCHS4 Coexpression,7;Enrichr Queries,82;GTEx Coexpression,33	[illegible]
6	ELF3	41.5	ARCHS4 Coexpression,78;Enrichr Queries,24;ReMap ChIP-seq,10;GTEx Coexpression,54	[illegible]
7	SOX15	44.67	ARCHS4 Coexpression,55;Enrichr Queries,70;GTEx Coexpression,9	[illegible]
8	HIF1A	46.6	Literature ChIP-seq,81;ARCHS4 Coexpression,13;Enrichr Queries,23;ReMap ChIP-seq,40;GTEx Coexpression,76	[illegible]
9	FOSL1	49.6	ARCHS4 Coexpression,1;ENCODE ChIP-seq,6;Enrichr Queries,170;ReMap ChIP-seq,11;GTEx Coexpression,60	[illegible]
10	TFCP2L1	49.67	ARCHS4 Coexpression,10;Enrichr Queries,38;GTEx Coexpression,101	[illegible]
11	FOXD1	53.5	ARCHS4 Coexpression,12;GTEx Coexpression,95	[illegible]
12	EHF	54.25	ARCHS4 Coexpression,35;Enrichr Queries,10;ReMap ChIP-seq,167;GTEx Coexpression,5	[illegible]
13	ELK3	55	Literature ChIP-seq,18;ARCHS4 Coexpression,31;Enrichr Queries,80;GTEx Coexpression,91	[illegible]
14	ATF3	62.67	Literature ChIP-seq,9;ARCHS4 Coexpression,91;ENCODE ChIP-seq,27;Enrichr Queries,50;ReMap ChIP-seq,27;GTEx Coexpression,172	[illegible]
15	HNF1B	63.33	ARCHS4 Coexpression,15;Enrichr Queries,139;ReMap ChIP-seq,36	[illegible]
16	TFAP2C	64.17	Literature ChIP-seq,64;ARCHS4 Coexpression,135;ENCODE ChIP-seq,36;Enrichr Queries,58;ReMap ChIP-seq,41;GTEx Coexpression,51	[illegible]
17	AHR	68.2	Literature ChIP-seq,71;ARCHS4 Coexpression,53;Enrichr Queries,61;ReMap ChIP-seq,84;GTEx Coexpression,72	[illegible]
18	ZNF750	70.75	ARCHS4 Coexpression,20;Enrichr Queries,75;ReMap ChIP-seq,139;GTEx Coexpression,49	[illegible]
19	KLF4	76.8	Literature ChIP-seq,7;ARCHS4 Coexpression,50;Enrichr Queries,35;ReMap ChIP-seq,12;GTEx Coexpression,280	[illegible]
20	OVOL1	80	ARCHS4 Coexpression,16;Enrichr Queries,177;GTEx Coexpression,47	[illegible]
21	TEAD3	81.33	ARCHS4 Coexpression,26;Enrichr Queries,89;GTEx Coexpression,129	[illegible]
22	BHLHE40	82.5	ARCHS4 Coexpression,104;ENCODE ChIP-seq,40;ReMap ChIP-seq,154;GTEx Coexpression,32	[illegible]
23	FOSL2	83.4	ARCHS4 Coexpression,192;ENCODE ChIP-seq,1;Enrichr Queries,31;ReMap ChIP-seq,1;GTEx Coexpression,192	[illegible]
24	SNAPC2	88.5	ARCHS4 Coexpression,51;GTEx Coexpression,126	[illegible]
25	TP63	92	Literature ChIP-seq,33;ARCHS4 Coexpression,21;Enrichr Queries,281;ReMap ChIP-seq,73;GTEx Coexpression,52	[illegible]
26	OSR1	93	ARCHS4 Coexpression,107;GTEx Coexpression,79	[illegible]
27	SNAI2	93	ARCHS4 Coexpression,37;Enrichr Queries,121;ReMap ChIP-seq,55;GTEx Coexpression,159	[illegible]
28	TP53	93.8	Literature ChIP-seq,67;ARCHS4 Coexpression,52;Enrichr Queries,237;ReMap ChIP-seq,107;GTEx Coexpression,6	[illegible]
29	ZNF469	95	ARCHS4 Coexpression,40;GTEx Coexpression,150	[illegible]
30	VDR	95	Literature ChIP-seq,73;ARCHS4 Coexpression,198;Enrichr Queries,36;ReMap ChIP-seq,25;GTEx Coexpression,143	[illegible]
31	BNC1	96.33	ARCHS4 Coexpression,5;Enrichr Queries,240;GTEx Coexpression,44	[illegible]
32	MTERF2	102	ARCHS4 Coexpression,43;GTEx Coexpression,161	[illegible]
33	FOS	105.4	ARCHS4 Coexpression,210;ENCODE ChIP-seq,25;Enrichr Queries,26;ReMap ChIP-seq,8;GTEx Coexpression,258	[illegible]
34	AEBP1	107.5	ARCHS4 Coexpression,96;GTEx Coexpression,119	[illegible]
35	PITX1	111.3	ARCHS4 Coexpression,142;Enrichr Queries,172;GTEx Coexpression,20	[illegible]
36	GRHL2	112	Literature ChIP-seq,125;ARCHS4 Coexpression,194;Enrichr Queries,171;ReMap ChIP-seq,5;GTEx Coexpression,65	[illegible]
37	GRHL1	112.3	ARCHS4 Coexpression,170;Enrichr Queries,149;GTEx Coexpression,18	[illegible]
38	PPARG	112.6	Literature ChIP-seq,54;ARCHS4 Coexpression,165;Enrichr Queries,95;ReMap ChIP-seq,26;GTEx Coexpression,223	[illegible]
39	EGR1	115.3	Literature ChIP-seq,63;ARCHS4 Coexpression,254;ENCODE ChIP-seq,78;Enrichr Queries,25;ReMap ChIP-seq,159;GTEx Coexpression,113	[illegible]
40	NR1H2	118.8	ARCHS4 Coexpression,126;Enrichr Queries,157;ReMap ChIP-seq,6;GTEx Coexpression,186	[illegible]
41	RUNX1	119.4	Literature ChIP-seq,48;ARCHS4 Coexpression,369;Enrichr Queries,79;ReMap ChIP-seq,45;GTEx Coexpression,56	[illegible]
42	FOXQ1	120.7	ARCHS4 Coexpression,140;Enrichr Queries,43;GTEx Coexpression,179	[illegible]
43	BARX2	122	ARCHS4 Coexpression,77;Enrichr Queries,287;GTEx Coexpression,2	[illegible]
44	ZNF436	122.3	ARCHS4 Coexpression,209;Enrichr Queries,142;GTEx Coexpression,16	[illegible]
45	FOXN1	122.7	ARCHS4 Coexpression,32;Enrichr Queries,123;GTEx Coexpression,213	[illegible]
46	SOX4	123	ARCHS4 Coexpression,323;Enrichr Queries,21;GTEx Coexpression,25	[illegible]
47	PPARD	127.5	Literature ChIP-seq,85;ARCHS4 Coexpression,256;Enrichr Queries,127;GTEx Coexpression,42	[illegible]
48	TCF7L2	130.3	Literature ChIP-seq,138;ARCHS4 Coexpression,305;ENCODE ChIP-seq,31;Enrichr Queries,192;ReMap ChIP-seq,23;GTEx Coexpression,93	[illegible]
49	GLIS2	135.7	ARCHS4 Coexpression,89;Enrichr Queries,196;GTEx Coexpression,122	[illegible]
50	ARNTL2	142	ARCHS4 Coexpression,4;Enrichr Queries,399;GTEx Coexpression,23	[illegible]
51	FOXA1	142.2	Literature ChIP-seq,116;ARCHS4 Coexpression,285;ENCODE ChIP-seq,69;Enrichr Queries,57;ReMap ChIP-seq,61;GTEx Coexpression,265	[illegible]
52	NR2F6	143	ARCHS4 Coexpression,146;Enrichr Queries,20;ReMap ChIP-seq,94;GTEx Coexpression,312	[illegible]
53	NR1H3	143.8	Literature ChIP-seq,90;ARCHS4 Coexpression,101;Enrichr Queries,106;ReMap ChIP-seq,254;GTEx Coexpression,168	[illegible]
54	SMAD3	147	Literature ChIP-seq,2;ARCHS4 Coexpression,25;Enrichr Queries,102;ReMap ChIP-seq,4;GTEx Coexpression,602	[illegible]
55	GLMP	147.5	ARCHS4 Coexpression,98;GTEx Coexpression,197	[illegible]
56	SRF	150.7	Literature ChIP-seq,103;ARCHS4 Coexpression,390;ENCODE ChIP-seq,20;Enrichr Queries,132;ReMap ChIP-seq,96;GTEx Coexpression,163	[illegible]
57	JUN	151.8	Literature ChIP-seq,10;ARCHS4 Coexpression,750;ENCODE ChIP-seq,22;Enrichr Queries,11;ReMap ChIP-seq,2;GTEx Coexpression,116	[illegible]
58	PRRX2	152.3	ARCHS4 Coexpression,122;Enrichr Queries,277;GTEx Coexpression,58	[illegible]
59	MAFB	152.8	ARCHS4 Coexpression,202;Enrichr Queries,73;ReMap ChIP-seq,50;GTEx Coexpression,286	[illegible]
60	TWIST2	158	ARCHS4 Coexpression,161;GTEx Coexpression,155	[illegible]
61	ZNF217	158.7	Literature ChIP-seq,11;ARCHS4 Coexpression,514;ENCODE ChIP-seq,13;Enrichr Queries,32;ReMap ChIP-seq,80;GTEx Coexpression,302	[illegible]
62	ZNF581	161.3	ARCHS4 Coexpression,74;Enrichr Queries,138;GTEx Coexpression,272	[illegible]
63	NME2	164.3	ARCHS4 Coexpression,204;ReMap ChIP-seq,275;GTEx Coexpression,14	[illegible]
64	MAFK	169.6	ARCHS4 Coexpression,464;ENCODE ChIP-seq,12;Enrichr Queries,86;ReMap ChIP-seq,97;GTEx Coexpression,189	[illegible]
65	ELF1	175.6	Literature ChIP-seq,86;ARCHS4 Coexpression,368;ENCODE ChIP-seq,110;ReMap ChIP-seq,110;GTEx Coexpression,204	[illegible]
66	RUNX2	177.4	Literature ChIP-seq,1;ARCHS4 Coexpression,550;Enrichr Queries,191;ReMap ChIP-seq,83;GTEx Coexpression,62	[illegible]
67	RBPJ	181.6	Literature ChIP-seq,113;ARCHS4 Coexpression,115;Enrichr Queries,76;ReMap ChIP-seq,233;GTEx Coexpression,371	[illegible]
68	GATA3	182.2	Literature ChIP-seq,42;ARCHS4 Coexpression,664;ENCODE ChIP-seq,17;Enrichr Queries,46;ReMap ChIP-seq,14;GTEx Coexpression,310	[illegible]
69	CREB3	182.8	ARCHS4 Coexpression,100;Enrichr Queries,6;ReMap ChIP-seq,144;GTEx Coexpression,481	[illegible]
70	ZHX1	183.3	ARCHS4 Coexpression,190;Enrichr Queries,444;ReMap ChIP-seq,51;GTEx Coexpression,48	[illegible]
71	ETV4	186.3	ARCHS4 Coexpression,480;Enrichr Queries,129;ReMap ChIP-seq,56;GTEx Coexpression,80	[illegible]
72	CEBPD	188.6	Literature ChIP-seq,147;ARCHS4 Coexpression,529;ENCODE ChIP-seq,21;ReMap ChIP-seq,140;GTEx Coexpression,106	[illegible]
73	MBNL2	189.3	ARCHS4 Coexpression,278;Enrichr Queries,59;GTEx Coexpression,231	[illegible]
74	FOXC2	194	ARCHS4 Coexpression,23;Enrichr Queries,467;GTEx Coexpression,92	[illegible]
75	NR1D1	196	ARCHS4 Coexpression,226;Enrichr Queries,47;GTEx Coexpression,315	[illegible]
76	CREB3L1	196.5	ARCHS4 Coexpression,36;Enrichr Queries,16;ReMap ChIP-seq,195;GTEx Coexpression,539	[illegible]
77	TCF12	197.6	ARCHS4 Coexpression,465;ENCODE ChIP-seq,11;Enrichr Queries,260;ReMap ChIP-seq,35;GTEx Coexpression,217	[illegible]
78	MSC	198	ARCHS4 Coexpression,105;Enrichr Queries,253;GTEx Coexpression,236	[illegible]
79	PAX9	199	ARCHS4 Coexpression,48;Enrichr Queries,522;GTEx Coexpression,27	[illegible]
80	JUND	199.5	Literature ChIP-seq,5;ARCHS4 Coexpression,768;ENCODE ChIP-seq,3;Enrichr Queries,1;ReMap ChIP-seq,3;GTEx Coexpression,417	[illegible]
81	ETS2	200.3	Literature ChIP-seq,136;ARCHS4 Coexpression,404;Enrichr Queries,49;GTEx Coexpression,212	[illegible]
82	IRX4	201.3	ARCHS4 Coexpression,33;Enrichr Queries,534;GTEx Coexpression,37	[illegible]
83	DLX3	202.3	ARCHS4 Coexpression,47;Enrichr Queries,286;GTEx Coexpression,274	[illegible]
84	NR2F2	203	ARCHS4 Coexpression,227;ENCODE ChIP-seq,18;Enrichr Queries,140;ReMap ChIP-seq,34;GTEx Coexpression,596	[illegible]
85	TRPS1	203.3	ARCHS4 Coexpression,230;Enrichr Queries,321;GTEx Coexpression,59	[illegible]
86	ESR1	204	Literature ChIP-seq,57;ARCHS4 Coexpression,334;ENCODE ChIP-seq,28;Enrichr Queries,248;ReMap ChIP-seq,47;GTEx Coexpression,510	[illegible]
87	HNF4A	208.7	Literature ChIP-seq,89;ARCHS4 Coexpression,383;ENCODE ChIP-seq,89;Enrichr Queries,126;ReMap ChIP-seq,117;GTEx Coexpression,448	[illegible]
88	PGR	210.8	ARCHS4 Coexpression,127;Enrichr Queries,527;ReMap ChIP-seq,22;GTEx Coexpression,167	[illegible]
89	CREB1	211	Literature ChIP-seq,65;ARCHS4 Coexpression,163;ENCODE ChIP-seq,67;Enrichr Queries,479;ReMap ChIP-seq,141;GTEx Coexpression,351	[illegible]
90	HES1	216	ARCHS4 Coexpression,479;Enrichr Queries,33;ReMap ChIP-seq,289;GTEx Coexpression,63	[illegible]
91	ATF4	217.8	ARCHS4 Coexpression,485;Enrichr Queries,9;ReMap ChIP-seq,122;GTEx Coexpression,255	[illegible]
92	GTF2B	219.3	ARCHS4 Coexpression,601;ENCODE ChIP-seq,32;ReMap ChIP-seq,112;GTEx Coexpression,132	[illegible]
93	RFX8	220.5	ARCHS4 Coexpression,364;GTEx Coexpression,77	[illegible]
94	CREB3L4	223	ARCHS4 Coexpression,275;Enrichr Queries,283;GTEx Coexpression,111	[illegible]
95	HOXB5	223.7	ARCHS4 Coexpression,68;Enrichr Queries,143;GTEx Coexpression,460	[illegible]
96	ZEB1	225.6	ARCHS4 Coexpression,391;ENCODE ChIP-seq,45;Enrichr Queries,280;ReMap ChIP-seq,76;GTEx Coexpression,336	[illegible]
97	HOXB7	227.2	Literature ChIP-seq,87;ARCHS4 Coexpression,577;Enrichr Queries,71;ReMap ChIP-seq,191;GTEx Coexpression,210	[illegible]
98	HOXA1	228	ARCHS4 Coexpression,136;Enrichr Queries,529;GTEx Coexpression,19	[illegible]
99	GATA2	228.3	Literature ChIP-seq,27;ARCHS4 Coexpression,344;ENCODE ChIP-seq,35;Enrichr Queries,150;ReMap ChIP-seq,95;GTEx Coexpression,719	[illegible]
100	FOXA2	228.7	Literature ChIP-seq,70;ARCHS4 Coexpression,501;ENCODE ChIP-seq,46;Enrichr Queries,99;ReMap ChIP-seq,64;GTEx Coexpression,592	[illegible]
101	ZBTB10	229	ARCHS4 Coexpression,19;Enrichr Queries,544;GTEx Coexpression,124	[illegible]
102	NPAS2	229.3	ARCHS4 Coexpression,402;Enrichr Queries,265;GTEx Coexpression,21	[illegible]
103	HMG20B	229.3	ARCHS4 Coexpression,144;Enrichr Queries,104;GTEx Coexpression,440	[illegible]
104	RELA	232	Literature ChIP-seq,6;ARCHS4 Coexpression,579;ENCODE ChIP-seq,7;Enrichr Queries,87;ReMap ChIP-seq,58;GTEx Coexpression,655	[illegible]
105	TCF4	232.6	Literature ChIP-seq,78;ARCHS4 Coexpression,328;Enrichr Queries,92;ReMap ChIP-seq,79;GTEx Coexpression,586	[illegible]
106	TWIST1	233	ARCHS4 Coexpression,315;Enrichr Queries,361;ReMap ChIP-seq,102;GTEx Coexpression,154	[illegible]
107	ELF5	235.8	Literature ChIP-seq,68;ARCHS4 Coexpression,743;Enrichr Queries,145;ReMap ChIP-seq,166;GTEx Coexpression,57	[illegible]
108	TBX3	236	Literature ChIP-seq,107;ARCHS4 Coexpression,329;Enrichr Queries,168;GTEx Coexpression,340	[illegible]
109	FOXL1	236.7	ARCHS4 Coexpression,11;Enrichr Queries,624;GTEx Coexpression,75	[illegible]
110	SPDEF	237	ARCHS4 Coexpression,269;Enrichr Queries,200;GTEx Coexpression,242	[illegible]
111	FOSB	237	ARCHS4 Coexpression,473;Enrichr Queries,53;GTEx Coexpression,185	[illegible]
112	ETS1	238.5	Literature ChIP-seq,118;ARCHS4 Coexpression,807;ENCODE ChIP-seq,4;Enrichr Queries,72;ReMap ChIP-seq,49;GTEx Coexpression,381	[illegible]
113	STAT3	239	Literature ChIP-seq,23;ARCHS4 Coexpression,696;ENCODE ChIP-seq,19;Enrichr Queries,29;ReMap ChIP-seq,15;GTEx Coexpression,652	[illegible]
114	MSANTD3	245.5	ARCHS4 Coexpression,121;GTEx Coexpression,370	[illegible]
115	MYC	245.7	Literature ChIP-seq,19;ARCHS4 Coexpression,1125;ENCODE ChIP-seq,59;Enrichr Queries,56;ReMap ChIP-seq,165;GTEx Coexpression,50	[illegible]
116	TFAP2A	247.2	Literature ChIP-seq,104;ARCHS4 Coexpression,1055;ENCODE ChIP-seq,5;Enrichr Queries,116;ReMap ChIP-seq,69;GTEx Coexpression,134	[illegible]
117	TEAD4	248.2	Literature ChIP-seq,112;ARCHS4 Coexpression,376;ENCODE ChIP-seq,2;Enrichr Queries,124;ReMap ChIP-seq,7;GTEx Coexpression,868	[illegible]
118	ZNF395	249	ARCHS4 Coexpression,455;Enrichr Queries,257;GTEx Coexpression,35	[illegible]
119	MXI1	249.2	ARCHS4 Coexpression,801;ENCODE ChIP-seq,73;Enrichr Queries,101;ReMap ChIP-seq,156;GTEx Coexpression,115	[illegible]
120	JUNB	250.5	ARCHS4 Coexpression,765;Enrichr Queries,22;ReMap ChIP-seq,70;GTEx Coexpression,145	[illegible]
121	ZNF277	251	ARCHS4 Coexpression,85;Enrichr Queries,400;GTEx Coexpression,268	[illegible]
122	KLF6	252.5	ARCHS4 Coexpression,528;Enrichr Queries,3;ReMap ChIP-seq,13;GTEx Coexpression,466	[illegible]
123	HMGA2	252.7	ARCHS4 Coexpression,339;Enrichr Queries,325;GTEx Coexpression,94	[illegible]
124	KLF7	256.3	ARCHS4 Coexpression,382;Enrichr Queries,223;GTEx Coexpression,164	[illegible]
125	EEA1	259	ARCHS4 Coexpression,179;Enrichr Queries,293;GTEx Coexpression,305	[illegible]
126	HHEX	259.5	ARCHS4 Coexpression,243;Enrichr Queries,103;ReMap ChIP-seq,268;GTEx Coexpression,424	[illegible]
127	ZNF697	260.7	ARCHS4 Coexpression,79;Enrichr Queries,323;GTEx Coexpression,380	[illegible]
128	BCL11A	261.6	ARCHS4 Coexpression,611;ENCODE ChIP-seq,29;Enrichr Queries,434;ReMap ChIP-seq,149;GTEx Coexpression,85	[illegible]
129	MAFF	261.6	ARCHS4 Coexpression,925;ENCODE ChIP-seq,37;Enrichr Queries,117;ReMap ChIP-seq,67;GTEx Coexpression,162	[illegible]
130	CDX2	264.6	Literature ChIP-seq,45;ARCHS4 Coexpression,679;Enrichr Queries,379;ReMap ChIP-seq,24;GTEx Coexpression,196	[illegible]
131	MKX	265	ARCHS4 Coexpression,118;Enrichr Queries,528;GTEx Coexpression,149	[illegible]
132	PRRX1	265.7	ARCHS4 Coexpression,158;Enrichr Queries,415;GTEx Coexpression,224	[illegible]
133	MLX	266.3	ARCHS4 Coexpression,145;Enrichr Queries,105;GTEx Coexpression,549	[illegible]
134	ZNF410	267	ARCHS4 Coexpression,433;Enrichr Queries,131;GTEx Coexpression,237	[illegible]
135	PRDM1	267.4	ARCHS4 Coexpression,713;ENCODE ChIP-seq,96;Enrichr Queries,273;ReMap ChIP-seq,227;GTEx Coexpression,28	[illegible]
136	CENPB	269	ARCHS4 Coexpression,426;Enrichr Queries,44;GTEx Coexpression,337	[illegible]
137	PAX8	269	ARCHS4 Coexpression,93;Enrichr Queries,97;ReMap ChIP-seq,241;GTEx Coexpression,645	[illegible]
138	NR4A1	269.8	ARCHS4 Coexpression,94;Enrichr Queries,34;ReMap ChIP-seq,255;GTEx Coexpression,696	[illegible]
139	HOXC13	270.7	ARCHS4 Coexpression,82;Enrichr Queries,460;GTEx Coexpression,270	[illegible]
140	EPAS1	273.8	ARCHS4 Coexpression,71;Enrichr Queries,48;ReMap ChIP-seq,71;GTEx Coexpression,905	[illegible]
141	TGIF1	274	ARCHS4 Coexpression,588;Enrichr Queries,27;GTEx Coexpression,207	[illegible]
142	GATA6	275.2	Literature ChIP-seq,15;ARCHS4 Coexpression,519;Enrichr Queries,147;ReMap ChIP-seq,18;GTEx Coexpression,677	[illegible]
143	ZBTB7C	275.3	ARCHS4 Coexpression,286;Enrichr Queries,533;GTEx Coexpression,7	[illegible]
144	NFIC	275.8	ARCHS4 Coexpression,416;ENCODE ChIP-seq,14;Enrichr Queries,160;ReMap ChIP-seq,17;GTEx Coexpression,772	[illegible]
145	CREB3L2	279.7	ARCHS4 Coexpression,90;Enrichr Queries,247;GTEx Coexpression,502	[illegible]
146	KLF9	281.3	ARCHS4 Coexpression,725;Enrichr Queries,62;ReMap ChIP-seq,127;GTEx Coexpression,211	[illegible]
147	KLF3	281.8	ARCHS4 Coexpression,602;Enrichr Queries,204;ReMap ChIP-seq,252;GTEx Coexpression,69	[illegible]
148	EGR2	282	ARCHS4 Coexpression,682;Enrichr Queries,83;GTEx Coexpression,81	[illegible]
149	CEBPB	282.8	Literature ChIP-seq,92;ARCHS4 Coexpression,544;ENCODE ChIP-seq,34;Enrichr Queries,17;ReMap ChIP-seq,29;GTEx Coexpression,981	[illegible]
150	SREBF1	284.7	Literature ChIP-seq,96;ARCHS4 Coexpression,905;ENCODE ChIP-seq,97;Enrichr Queries,42;ReMap ChIP-seq,202;GTEx Coexpression,366	[illegible]
151	ZNF274	286.7	Literature ChIP-seq,153;ARCHS4 Coexpression,281;ENCODE ChIP-seq,95;Enrichr Queries,372;ReMap ChIP-seq,277;GTEx Coexpression,542	[illegible]
152	TAL1	287.7	Literature ChIP-seq,16;ARCHS4 Coexpression,809;ENCODE ChIP-seq,64;Enrichr Queries,258;ReMap ChIP-seq,90;GTEx Coexpression,489	[illegible]
153	SP6	287.7	ARCHS4 Coexpression,187;Enrichr Queries,363;GTEx Coexpression,313	[illegible]
154	SNAI1	288	ARCHS4 Coexpression,371;Enrichr Queries,242;GTEx Coexpression,251	[illegible]
155	UBP1	289	ARCHS4 Coexpression,671;Enrichr Queries,96;GTEx Coexpression,100	[illegible]
156	MYB	294.3	Literature ChIP-seq,25;ARCHS4 Coexpression,821;ENCODE ChIP-seq,117;Enrichr Queries,543;ReMap ChIP-seq,153;GTEx Coexpression,107	[illegible]
157	SOX9	294.8	Literature ChIP-seq,39;ARCHS4 Coexpression,628;Enrichr Queries,39;ReMap ChIP-seq,33;GTEx Coexpression,735	[illegible]
158	SIX5	297	ARCHS4 Coexpression,517;ENCODE ChIP-seq,101;Enrichr Queries,424;ReMap ChIP-seq,210;GTEx Coexpression,233	[illegible]
159	RFX5	297.2	ARCHS4 Coexpression,691;ENCODE ChIP-seq,54;Enrichr Queries,299;ReMap ChIP-seq,119;GTEx Coexpression,323	[illegible]
160	HOXA3	298.7	ARCHS4 Coexpression,197;Enrichr Queries,521;GTEx Coexpression,178	[illegible]
161	ATOH8	299.7	ARCHS4 Coexpression,24;Enrichr Queries,389;GTEx Coexpression,486	[illegible]
162	HOXA9	299.8	ARCHS4 Coexpression,428;Enrichr Queries,381;ReMap ChIP-seq,244;GTEx Coexpression,146	[illegible]
163	PBX1	299.8	Literature ChIP-seq,123;ARCHS4 Coexpression,377;Enrichr Queries,309;GTEx Coexpression,390	[illegible]
164	SP5	302.3	ARCHS4 Coexpression,348;Enrichr Queries,473;GTEx Coexpression,86	[illegible]
165	XBP1	305.5	ARCHS4 Coexpression,143;Enrichr Queries,4;ReMap ChIP-seq,209;GTEx Coexpression,866	[illegible]
166	ZNF761	306	ARCHS4 Coexpression,347;Enrichr Queries,459;GTEx Coexpression,112	[illegible]
167	NR3C1	306.5	Literature ChIP-seq,100;ARCHS4 Coexpression,834;ENCODE ChIP-seq,8;Enrichr Queries,115;ReMap ChIP-seq,21;GTEx Coexpression,761	[illegible]
168	FOXS1	308	ARCHS4 Coexpression,434;Enrichr Queries,380;GTEx Coexpression,110	[illegible]
169	MBD2	308.3	ARCHS4 Coexpression,785;Enrichr Queries,28;ReMap ChIP-seq,290;GTEx Coexpression,130	[illegible]
170	TBP	308.4	Literature ChIP-seq,83;ARCHS4 Coexpression,109;ENCODE ChIP-seq,41;ReMap ChIP-seq,78;GTEx Coexpression,1231	[illegible]
171	BPTF	308.5	ARCHS4 Coexpression,80;GTEx Coexpression,537	[illegible]
172	ELK1	309	Literature ChIP-seq,26;ARCHS4 Coexpression,349;ENCODE ChIP-seq,68;ReMap ChIP-seq,221;GTEx Coexpression,881	[illegible]
173	ZNF189	310.3	ARCHS4 Coexpression,199;Enrichr Queries,518;GTEx Coexpression,214	[illegible]
174	FLI1	310.3	Literature ChIP-seq,17;ARCHS4 Coexpression,640;ENCODE ChIP-seq,109;Enrichr Queries,308;ReMap ChIP-seq,220;GTEx Coexpression,568	[illegible]

175	NFYB	310.6	ARCHS4 Coexpression,466;ENCODE ChIP-seq,47;Enrichr Queries,560;ReMap ChIP-seq,260;GTEx Coexpression,220	[illegible]
176	SREBF2	310.8	Literature ChIP-seq,141;ARCHS4 Coexpression,929;ENCODE ChIP-seq,70;Enrichr Queries,120;ReMap ChIP-seq,171;GTEx Coexpression,434	[illegible]
177	HMGN3	312	ARCHS4 Coexpression,708;ENCODE ChIP-seq,115;ReMap ChIP-seq,197;GTEx Coexpression,228	[illegible]
178	JAZF1	313.3	ARCHS4 Coexpression,330;Enrichr Queries,388;GTEx Coexpression,222	[illegible]
179	ZNF721	315.7	ARCHS4 Coexpression,17;Enrichr Queries,274;GTEx Coexpression,656	[illegible]
180	SP100	315.7	ARCHS4 Coexpression,457;Enrichr Queries,291;GTEx Coexpression,199	[illegible]
181	ZNF326	318.3	ARCHS4 Coexpression,130;Enrichr Queries,506;GTEx Coexpression,319	[illegible]
182	RELB	319	ARCHS4 Coexpression,662;Enrichr Queries,201;ReMap ChIP-seq,198;GTEx Coexpression,215	[illegible]
183	JDP2	319	ARCHS4 Coexpression,695;Enrichr Queries,60;GTEx Coexpression,202	[illegible]
184	KDM5B	319.8	Literature ChIP-seq,155;ARCHS4 Coexpression,206;ENCODE ChIP-seq,108;ReMap ChIP-seq,118;GTEx Coexpression,1012	[illegible]
185	NR0B1	320.5	Literature ChIP-seq,44;ARCHS4 Coexpression,488;Enrichr Queries,412;GTEx Coexpression,338	[illegible]
186	CEBPA	322.2	Literature ChIP-seq,117;ARCHS4 Coexpression,605;Enrichr Queries,64;ReMap ChIP-seq,181;GTEx Coexpression,644	[illegible]
187	MBD4	322.4	ARCHS4 Coexpression,249;ENCODE ChIP-seq,63;Enrichr Queries,366;ReMap ChIP-seq,169;GTEx Coexpression,765	[illegible]
188	CREBL2	324.7	ARCHS4 Coexpression,419;Enrichr Queries,176;GTEx Coexpression,379	[illegible]
189	HOXB6	325.7	ARCHS4 Coexpression,506;Enrichr Queries,141;GTEx Coexpression,330	[illegible]
190	ZNF263	326.7	Literature ChIP-seq,158;ARCHS4 Coexpression,814;ENCODE ChIP-seq,79;Enrichr Queries,295;ReMap ChIP-seq,216;GTEx Coexpression,398	[illegible]
191	GFI1B	327.6	Literature ChIP-seq,55;ARCHS4 Coexpression,221;Enrichr Queries,374;ReMap ChIP-seq,99;GTEx Coexpression,889	[illegible]
192	IKZF4	328	ARCHS4 Coexpression,148;Enrichr Queries,697;GTEx Coexpression,139	[illegible]
193	MEF2A	329.8	Literature ChIP-seq,149;ARCHS4 Coexpression,599;ENCODE ChIP-seq,38;Enrichr Queries,224;ReMap ChIP-seq,126;GTEx Coexpression,843	[illegible]
194	MAX	331.8	ARCHS4 Coexpression,166;ENCODE ChIP-seq,44;Enrichr Queries,135;ReMap ChIP-seq,116;GTEx Coexpression,1198	[illegible]
195	ZNF598	334.3	ARCHS4 Coexpression,389;Enrichr Queries,338;GTEx Coexpression,276	[illegible]
196	FOXF1	335	ARCHS4 Coexpression,83;Enrichr Queries,470;GTEx Coexpression,452	[illegible]
197	ZNF22	336.3	ARCHS4 Coexpression,345;Enrichr Queries,111;GTEx Coexpression,553	[illegible]
198	SKIL	337.7	ARCHS4 Coexpression,566;Enrichr Queries,85;GTEx Coexpression,362	[illegible]
199	ERG	337.8	Literature ChIP-seq,32;ARCHS4 Coexpression,742;Enrichr Queries,457;ReMap ChIP-seq,232;GTEx Coexpression,226	[illegible]
200	ZNF883	338	ARCHS4 Coexpression,168;GTEx Coexpression,508	[illegible]
201	SOX2	339.6	Literature ChIP-seq,24;ARCHS4 Coexpression,542;Enrichr Queries,419;ReMap ChIP-seq,217;GTEx Coexpression,496	[illegible]
202	ZNF561	339.7	ARCHS4 Coexpression,252;Enrichr Queries,741;GTEx Coexpression,26	[illegible]
203	HSF1	340	Literature ChIP-seq,60;ARCHS4 Coexpression,260;ENCODE ChIP-seq,74;ReMap ChIP-seq,211;GTEx Coexpression,1095	[illegible]
204	NANOG	341.2	Literature ChIP-seq,34;ARCHS4 Coexpression,622;ENCODE ChIP-seq,62;Enrichr Queries,557;ReMap ChIP-seq,242;GTEx Coexpression,530	[illegible]
205	MSX2	341.3	ARCHS4 Coexpression,386;Enrichr Queries,255;GTEx Coexpression,383	[illegible]
206	EBF1	341.4	ARCHS4 Coexpression,359;ENCODE ChIP-seq,102;Enrichr Queries,524;ReMap ChIP-seq,218;GTEx Coexpression,504	[illegible]
207	ATF2	343.2	ARCHS4 Coexpression,248;ENCODE ChIP-seq,61;Enrichr Queries,876;ReMap ChIP-seq,86;GTEx Coexpression,445	[illegible]
208	SMAD9	344	ARCHS4 Coexpression,67;Enrichr Queries,656;GTEx Coexpression,309	[illegible]
209	YY1	344	Literature ChIP-seq,108;ARCHS4 Coexpression,913;ENCODE ChIP-seq,85;Enrichr Queries,158;ReMap ChIP-seq,223;GTEx Coexpression,577	[illegible]
210	ZNF608	347.7	ARCHS4 Coexpression,272;Enrichr Queries,663;GTEx Coexpression,108	[illegible]
211	ZBTB33	349.4	ARCHS4 Coexpression,609;ENCODE ChIP-seq,56;Enrichr Queries,847;ReMap ChIP-seq,48;GTEx Coexpression,187	[illegible]
212	ZBTB42	349.5	ARCHS4 Coexpression,270;GTEx Coexpression,429	[illegible]
213	ZSCAN2	350	ARCHS4 Coexpression,174;Enrichr Queries,476;GTEx Coexpression,400	[illegible]
214	HNF4G	350.8	ARCHS4 Coexpression,669;ENCODE ChIP-seq,84;Enrichr Queries,685;ReMap ChIP-seq,133;GTEx Coexpression,183	[illegible]
215	PA2G4	351	ARCHS4 Coexpression,405;GTEx Coexpression,297	[illegible]
216	MAF	351.5	ARCHS4 Coexpression,936;Enrichr Queries,112;ReMap ChIP-seq,155;GTEx Coexpression,203	[illegible]
217	TGIF2	352	ARCHS4 Coexpression,282;Enrichr Queries,458;ReMap ChIP-seq,183;GTEx Coexpression,485	[illegible]
218	WT1	352.5	Literature ChIP-seq,40;ARCHS4 Coexpression,616;Enrichr Queries,165;GTEx Coexpression,589	[illegible]
219	SETBP1	352.7	ARCHS4 Coexpression,103;Enrichr Queries,790;GTEx Coexpression,165	[illegible]
220	IRX3	352.7	ARCHS4 Coexpression,675;Enrichr Queries,65;GTEx Coexpression,318	[illegible]
221	KLF2	353.5	Literature ChIP-seq,111;ARCHS4 Coexpression,1014;Enrichr Queries,41;GTEx Coexpression,248	[illegible]
222	ZBED2	353.7	ARCHS4 Coexpression,482;Enrichr Queries,275;GTEx Coexpression,304	[illegible]
223	AEBP2	354	ARCHS4 Coexpression,413;Enrichr Queries,526;GTEx Coexpression,123	[illegible]
224	STAT5A	355.7	Literature ChIP-seq,31;ARCHS4 Coexpression,1344;ENCODE ChIP-seq,106;Enrichr Queries,118;ReMap ChIP-seq,57;GTEx Coexpression,478	[illegible]
225	STAT1	357.3	Literature ChIP-seq,151;ARCHS4 Coexpression,900;ENCODE ChIP-seq,81;Enrichr Queries,52;ReMap ChIP-seq,31;GTEx Coexpression,929	[illegible]
226	ZNF212	357.7	ARCHS4 Coexpression,205;Enrichr Queries,815;GTEx Coexpression,53	[illegible]
227	ZBTB7A	358.2	ARCHS4 Coexpression,1147;ENCODE ChIP-seq,52;Enrichr Queries,290;ReMap ChIP-seq,214;GTEx Coexpression,88	[illegible]
228	SP1	360.8	ARCHS4 Coexpression,1082;ENCODE ChIP-seq,23;Enrichr Queries,334;ReMap ChIP-seq,66;GTEx Coexpression,299	[illegible]
229	MEF2C	361.6	ARCHS4 Coexpression,597;ENCODE ChIP-seq,51;Enrichr Queries,184;ReMap ChIP-seq,228;GTEx Coexpression,748	[illegible]
230	DACH1	363	Literature ChIP-seq,135;ARCHS4 Coexpression,289;Enrichr Queries,665	[illegible]
231	OSR2	363	ARCHS4 Coexpression,351;Enrichr Queries,233;GTEx Coexpression,505	[illegible]
232	CDX1	363.7	ARCHS4 Coexpression,689;Enrichr Queries,197;GTEx Coexpression,205	[illegible]
233	PLSCR1	364.5	ARCHS4 Coexpression,287;GTEx Coexpression,442	[illegible]
234	E2F4	364.7	Literature ChIP-seq,101;ARCHS4 Coexpression,1033;ENCODE ChIP-seq,71;Enrichr Queries,563;ReMap ChIP-seq,186;GTEx Coexpression,234	[illegible]
235	MITF	365	Literature ChIP-seq,102;ARCHS4 Coexpression,175;Enrichr Queries,185;ReMap ChIP-seq,212;GTEx Coexpression,1151	[illegible]
236	MAFG	365.5	ARCHS4 Coexpression,291;Enrichr Queries,166;ReMap ChIP-seq,175;GTEx Coexpression,830	[illegible]
237	HOXB1	367.7	ARCHS4 Coexpression,271;Enrichr Queries,831;GTEx Coexpression,1	[illegible]
238	TFE3	367.7	ARCHS4 Coexpression,366;Enrichr Queries,78;GTEx Coexpression,659	[illegible]
239	ZBED3	369.3	ARCHS4 Coexpression,415;Enrichr Queries,536;GTEx Coexpression,157	[illegible]
240	SATB1	371.3	ARCHS4 Coexpression,370;Enrichr Queries,336;ReMap ChIP-seq,288;GTEx Coexpression,491	[illegible]
241	MYCN	371.4	Literature ChIP-seq,145;ARCHS4 Coexpression,914;Enrichr Queries,354;ReMap ChIP-seq,182;GTEx Coexpression,262	[illegible]
242	NFIB	371.8	Literature ChIP-seq,114;ARCHS4 Coexpression,613;Enrichr Queries,189;GTEx Coexpression,571	[illegible]
243	POU3F1	372.5	Literature ChIP-seq,130;ARCHS4 Coexpression,396;Enrichr Queries,650;GTEx Coexpression,314	[illegible]
244	PBX3	373.2	ARCHS4 Coexpression,837;ENCODE ChIP-seq,33;Enrichr Queries,385;ReMap ChIP-seq,39;GTEx Coexpression,572	[illegible]
245	ZEB2	375.3	ARCHS4 Coexpression,856;Enrichr Queries,187;ReMap ChIP-seq,81;GTEx Coexpression,377	[illegible]
246	SOX17	376	Literature ChIP-seq,22;ARCHS4 Coexpression,940;Enrichr Queries,285;GTEx Coexpression,257	[illegible]
247	CUX1	377	Literature ChIP-seq,53;ARCHS4 Coexpression,852;ENCODE ChIP-seq,55;Enrichr Queries,259;ReMap ChIP-seq,72;GTEx Coexpression,971	[illegible]
248	SMAD4	378.4	Literature ChIP-seq,3;ARCHS4 Coexpression,635;Enrichr Queries,337;ReMap ChIP-seq,257;GTEx Coexpression,660	[illegible]
249	NCOA3	378.5	ARCHS4 Coexpression,469;Enrichr Queries,5;ReMap ChIP-seq,199;GTEx Coexpression,841	[illegible]
250	ZNF704	379.3	ARCHS4 Coexpression,203;Enrichr Queries,740;GTEx Coexpression,195	[illegible]
251	SOX13	379.5	ARCHS4 Coexpression,1066;Enrichr Queries,130;ReMap ChIP-seq,147;GTEx Coexpression,175	[illegible]
252	GATA1	380.7	Literature ChIP-seq,62;ARCHS4 Coexpression,657;ENCODE ChIP-seq,77;Enrichr Queries,454;ReMap ChIP-seq,113;GTEx Coexpression,921	[illegible]
253	ZNF322	381	ARCHS4 Coexpression,62;GTEx Coexpression,700	[illegible]
254	RORA	381.7	ARCHS4 Coexpression,317;Enrichr Queries,537;GTEx Coexpression,291	[illegible]
255	HES2	381.7	ARCHS4 Coexpression,907;Enrichr Queries,225;GTEx Coexpression,13	[illegible]
256	MNX1	382	Literature ChIP-seq,91;ARCHS4 Coexpression,490;Enrichr Queries,649;GTEx Coexpression,298	[illegible]
257	POU5F1	382.4	Literature ChIP-seq,36;ARCHS4 Coexpression,1088;Enrichr Queries,362;ReMap ChIP-seq,256;GTEx Coexpression,170	[illegible]
258	IRF3	382.4	ARCHS4 Coexpression,1225;ENCODE ChIP-seq,75;Enrichr Queries,134;ReMap ChIP-seq,237;GTEx Coexpression,241	[illegible]
259	HOXB8	382.7	ARCHS4 Coexpression,394;Enrichr Queries,394;GTEx Coexpression,360	[illegible]
260	HOXD8	387	ARCHS4 Coexpression,222;Enrichr Queries,414;GTEx Coexpression,525	[illegible]
261	NFE2	389.8	ARCHS4 Coexpression,658;ENCODE ChIP-seq,104;Enrichr Queries,180;ReMap ChIP-seq,59;GTEx Coexpression,948	[illegible]
262	BACH1	390.5	Literature ChIP-seq,76;ARCHS4 Coexpression,1228;ENCODE ChIP-seq,50;Enrichr Queries,216;ReMap ChIP-seq,44;GTEx Coexpression,729	[illegible]
263	HIF3A	390.7	ARCHS4 Coexpression,532;Enrichr Queries,387;GTEx Coexpression,253	[illegible]
264	HOXB2	391	ARCHS4 Coexpression,388;Enrichr Queries,313;GTEx Coexpression,472	[illegible]
265	ZNF550	391	ARCHS4 Coexpression,66;Enrichr Queries,1004;GTEx Coexpression,103	[illegible]
266	SPI1	391	Literature ChIP-seq,50;ARCHS4 Coexpression,1334;ENCODE ChIP-seq,26;Enrichr Queries,264;ReMap ChIP-seq,62;GTEx Coexpression,610	[illegible]
267	ZNF462	391.3	ARCHS4 Coexpression,177;Enrichr Queries,548;GTEx Coexpression,449	[illegible]
268	ZNF468	391.3	ARCHS4 Coexpression,560;Enrichr Queries,416;GTEx Coexpression,198	[illegible]
269	GLIS1	392.7	ARCHS4 Coexpression,42;Enrichr Queries,978;GTEx Coexpression,158	[illegible]
270	ZNF385A	393.7	ARCHS4 Coexpression,1010;Enrichr Queries,66;GTEx Coexpression,105	[illegible]
271	ZNF428	394.3	ARCHS4 Coexpression,420;Enrichr Queries,51;GTEx Coexpression,712	[illegible]
272	HOXC11	397.5	ARCHS4 Coexpression,106;Enrichr Queries,903;ReMap ChIP-seq,294;GTEx Coexpression,287	[illegible]
273	GLIS3	398.7	ARCHS4 Coexpression,128;Enrichr Queries,469;GTEx Coexpression,599	[illegible]
274	NR2F1	399.3	ARCHS4 Coexpression,453;Enrichr Queries,421;ReMap ChIP-seq,179;GTEx Coexpression,544	[illegible]
275	ZNF575	399.7	ARCHS4 Coexpression,223;Enrichr Queries,267;GTEx Coexpression,709	[illegible]
276	ZNF524	400.7	ARCHS4 Coexpression,178;Enrichr Queries,455;GTEx Coexpression,569	[illegible]
277	SOX18	401.3	ARCHS4 Coexpression,411;Enrichr Queries,401;GTEx Coexpression,392	[illegible]
278	ETV6	401.5	ARCHS4 Coexpression,841;Enrichr Queries,298;ReMap ChIP-seq,160;GTEx Coexpression,307	[illegible]
279	NR4A2	401.5	Literature ChIP-seq,148;ARCHS4 Coexpression,504;Enrichr Queries,154;GTEx Coexpression,800	[illegible]
280	ELK4	401.8	ARCHS4 Coexpression,1206;ENCODE ChIP-seq,65;Enrichr Queries,562;ReMap ChIP-seq,103;GTEx Coexpression,73	[illegible]
281	NACC2	403.5	ARCHS4 Coexpression,310;GTEx Coexpression,497	[illegible]
282	POU2F2	404.8	ARCHS4 Coexpression,1077;ENCODE ChIP-seq,93;Enrichr Queries,501;ReMap ChIP-seq,82;GTEx Coexpression,271	[illegible]
283	ZNF33B	405	ARCHS4 Coexpression,18;Enrichr Queries,756;GTEx Coexpression,441	[illegible]
284	FOXP1	405.2	Literature ChIP-seq,46;ARCHS4 Coexpression,1201;Enrichr Queries,439;ReMap ChIP-seq,203;GTEx Coexpression,137	[illegible]
285	HOXC6	405.3	ARCHS4 Coexpression,847;Enrichr Queries,203;GTEx Coexpression,166	[illegible]
286	ZKSCAN1	405.4	ARCHS4 Coexpression,76;ENCODE ChIP-seq,60;Enrichr Queries,567;ReMap ChIP-seq,132;GTEx Coexpression,1192	[illegible]
287	MEIS1	405.8	Literature ChIP-seq,110;ARCHS4 Coexpression,615;Enrichr Queries,516;ReMap ChIP-seq,163;GTEx Coexpression,625	[illegible]
288	CXXC4	406.7	ARCHS4 Coexpression,539;ReMap ChIP-seq,106;GTEx Coexpression,575	[illegible]
289	THAP11	408.4	Literature ChIP-seq,152;ARCHS4 Coexpression,123;Enrichr Queries,181;ReMap ChIP-seq,282;GTEx Coexpression,1304	[illegible]
290	HMGA1	409	ARCHS4 Coexpression,543;Enrichr Queries,15;GTEx Coexpression,669	[illegible]
291	RXRA	409	Literature ChIP-seq,156;ARCHS4 Coexpression,1400;ENCODE ChIP-seq,16;Enrichr Queries,55;ReMap ChIP-seq,74;GTEx Coexpression,753	[illegible]
292	HEY1	409	ARCHS4 Coexpression,565;Enrichr Queries,161;GTEx Coexpression,501	[illegible]
293	ADNP	409.3	ARCHS4 Coexpression,236;Enrichr Queries,626;ReMap ChIP-seq,264;GTEx Coexpression,511	[illegible]
294	TEAD2	410.3	ARCHS4 Coexpression,1073;Enrichr Queries,68;ReMap ChIP-seq,194;GTEx Coexpression,306	[illegible]
295	ZNF415	410.3	ARCHS4 Coexpression,147;Enrichr Queries,807;GTEx Coexpression,277	[illegible]
296	ZFP62	410.7	ARCHS4 Coexpression,86;Enrichr Queries,857;GTEx Coexpression,289	[illegible]
297	ZZZ3	413	ARCHS4 Coexpression,294;ENCODE ChIP-seq,49;Enrichr Queries,954;ReMap ChIP-seq,230;GTEx Coexpression,538	[illegible]
298	XPA	413.3	ARCHS4 Coexpression,500;Enrichr Queries,474;GTEx Coexpression,266	[illegible]
299	MEOX2	413.7	ARCHS4 Coexpression,407;Enrichr Queries,657;GTEx Coexpression,177	[illegible]
300	TBX18	414	ARCHS4 Coexpression,60;Enrichr Queries,829;GTEx Coexpression,353	[illegible]
301	NFE2L3	415.3	ARCHS4 Coexpression,507;Enrichr Queries,152;GTEx Coexpression,587	[illegible]
302	MEIS2	416.3	ARCHS4 Coexpression,262;Enrichr Queries,535;ReMap ChIP-seq,143;GTEx Coexpression,725	[illegible]
303	IRF1	419.2	Literature ChIP-seq,134;ARCHS4 Coexpression,1323;ENCODE ChIP-seq,58;Enrichr Queries,303;ReMap ChIP-seq,54;GTEx Coexpression,643	[illegible]
304	TCF21	420.8	Literature ChIP-seq,20;ARCHS4 Coexpression,442;Enrichr Queries,271;GTEx Coexpression,950	[illegible]
305	TCF3	420.8	Literature ChIP-seq,38;ARCHS4 Coexpression,874;ENCODE ChIP-seq,24;Enrichr Queries,319;ReMap ChIP-seq,68;GTEx Coexpression,1202	[illegible]
306	TSHZ2	422	ARCHS4 Coexpression,258;Enrichr Queries,764;GTEx Coexpression,244	[illegible]
307	DLX4	422.3	Literature ChIP-seq,164;ARCHS4 Coexpression,644;Enrichr Queries,580;GTEx Coexpression,301	[illegible]
308	REST	422.3	Literature ChIP-seq,58;ARCHS4 Coexpression,926;ENCODE ChIP-seq,15;Enrichr Queries,426;ReMap ChIP-seq,151;GTEx Coexpression,958	[illegible]
309	ARNT	422.6	Literature ChIP-seq,88;ARCHS4 Coexpression,639;Enrichr Queries,681;ReMap ChIP-seq,75;GTEx Coexpression,630	[illegible]
310	EGR3	423.7	ARCHS4 Coexpression,643;Enrichr Queries,151;GTEx Coexpression,477	[illegible]
311	GATAD2A	425.3	ARCHS4 Coexpression,631;Enrichr Queries,227;GTEx Coexpression,418	[illegible]
312	NKX21	427	ARCHS4 Coexpression,188;GTEx Coexpression,666	[illegible]
313	FOXO1	427.2	Literature ChIP-seq,61;ARCHS4 Coexpression,598;Enrichr Queries,195;ReMap ChIP-seq,177;GTEx Coexpression,1105	[illegible]
314	LYL1	427.4	Literature ChIP-seq,133;ARCHS4 Coexpression,638;Enrichr Queries,263;ReMap ChIP-seq,200;GTEx Coexpression,903	[illegible]
315	NFE2L1	427.5	ARCHS4 Coexpression,133;Enrichr Queries,8;ReMap ChIP-seq,168;GTEx Coexpression,1401	[illegible]
316	ELF4	427.7	ARCHS4 Coexpression,1096;Enrichr Queries,40;GTEx Coexpression,147	[illegible]
317	FOXP2	428	Literature ChIP-seq,75;ARCHS4 Coexpression,499;ENCODE ChIP-seq,99;Enrichr Queries,1292;ReMap ChIP-seq,164;GTEx Coexpression,439	[illegible]
318	ZNF827	429.7	ARCHS4 Coexpression,57;Enrichr Queries,669;GTEx Coexpression,563	[illegible]
319	ZBTB4	431.3	ARCHS4 Coexpression,429;Enrichr Queries,250;GTEx Coexpression,615	[illegible]
320	KCMF1	433	ARCHS4 Coexpression,502;Enrichr Queries,119;GTEx Coexpression,678	[illegible]
321	TEAD1	433.3	ARCHS4 Coexpression,41;Enrichr Queries,214;ReMap ChIP-seq,19;GTEx Coexpression,1459	[illegible]
322	GATA4	435	Literature ChIP-seq,21;ARCHS4 Coexpression,1140;Enrichr Queries,593;ReMap ChIP-seq,20;GTEx Coexpression,401	[illegible]
323	LHX6	436	ARCHS4 Coexpression,666;Enrichr Queries,417;GTEx Coexpression,225	[illegible]
324	KLF12	436.3	ARCHS4 Coexpression,131;Enrichr Queries,939;GTEx Coexpression,239	[illegible]
325	ESRRA	436.6	ARCHS4 Coexpression,456;ENCODE ChIP-seq,103;Enrichr Queries,346;ReMap ChIP-seq,38;GTEx Coexpression,1240	[illegible]
326	HEYL	437.3	ARCHS4 Coexpression,863;Enrichr Queries,331;GTEx Coexpression,118	[illegible]
327	PIN1	437.5	ARCHS4 Coexpression,266;GTEx Coexpression,609	[illegible]
328	ZNF823	438.3	ARCHS4 Coexpression,734;Enrichr Queries,570;GTEx Coexpression,11	[illegible]
329	BCL11B	438.8	Literature ChIP-seq,161;ARCHS4 Coexpression,783;Enrichr Queries,610;GTEx Coexpression,201	[illegible]
330	OVOL2	440	ARCHS4 Coexpression,1054;Enrichr Queries,269;ReMap ChIP-seq,295;GTEx Coexpression,142	[illegible]
331	VEZF1	441.3	ARCHS4 Coexpression,292;Enrichr Queries,108;GTEx Coexpression,924	[illegible]
332	HES4	441.7	ARCHS4 Coexpression,1021;Enrichr Queries,230;GTEx Coexpression,74	[illegible]
333	FOXA3	441.7	ARCHS4 Coexpression,510;Enrichr Queries,178;GTEx Coexpression,637	[illegible]
334	ZNF384	442	ARCHS4 Coexpression,1025;ENCODE ChIP-seq,53;Enrichr Queries,568;ReMap ChIP-seq,148;GTEx Coexpression,416	[illegible]
335	RARB	442.5	Literature ChIP-seq,129;ARCHS4 Coexpression,326;Enrichr Queries,582;GTEx Coexpression,733	[illegible]
336	SMAD5	442.5	ARCHS4 Coexpression,570;Enrichr Queries,758;ReMap ChIP-seq,43;GTEx Coexpression,399	[illegible]
337	PROX1	446	ARCHS4 Coexpression,637;Enrichr Queries,672;ReMap ChIP-seq,16;GTEx Coexpression,459	[illegible]
338	KLF1	446.6	Literature ChIP-seq,35;ARCHS4 Coexpression,672;Enrichr Queries,499;ReMap ChIP-seq,63;GTEx Coexpression,964	[illegible]
339	KLF10	448.3	ARCHS4 Coexpression,44;Enrichr Queries,54;GTEx Coexpression,1247	[illegible]
340	ZNF552	450.3	ARCHS4 Coexpression,151;Enrichr Queries,710;GTEx Coexpression,490	[illegible]
341	NR5A1	451	ARCHS4 Coexpression,757;Enrichr Queries,239;GTEx Coexpression,357	[illegible]
342	ZBTB5	451.3	ARCHS4 Coexpression,728;Enrichr Queries,597;GTEx Coexpression,29	[illegible]
343	NKX12	451.5	ARCHS4 Coexpression,891;GTEx Coexpression,12	[illegible]
344	SOX11	453.2	Literature ChIP-seq,43;ARCHS4 Coexpression,755;Enrichr Queries,674;ReMap ChIP-seq,287;GTEx Coexpression,507	[illegible]
345	ZNF175	453.3	ARCHS4 Coexpression,468;Enrichr Queries,398;ReMap ChIP-seq,108;GTEx Coexpression,839	[illegible]
346	NR5A2	453.8	ARCHS4 Coexpression,810;Enrichr Queries,463;ReMap ChIP-seq,91;GTEx Coexpression,451	[illegible]
347	USF2	454	ARCHS4 Coexpression,1204;ENCODE ChIP-seq,94;Enrichr Queries,74;ReMap ChIP-seq,204;GTEx Coexpression,694	[illegible]
348	ZNF177	454	ARCHS4 Coexpression,72;Enrichr Queries,739;GTEx Coexpression,551	[illegible]
349	MSGN1	454.3	ARCHS4 Coexpression,572;Enrichr Queries,783;GTEx Coexpression,8	[illegible]
350	ZNF799	454.7	ARCHS4 Coexpression,153;Enrichr Queries,670;GTEx Coexpression,541	[illegible]

351	NFATC1	455.8	ARCHS4 Coexpression,709;ENCODE ChIP-seq,83;Enrichr Queries,245;ReMap ChIP-seq,188;GTEx Coexpression,1054	[illegible]
352	SP4	456.2	ARCHS4 Coexpression,29;ENCODE ChIP-seq,88;Enrichr Queries,858;ReMap ChIP-seq,88;GTEx Coexpression,1218	[illegible]
353	MBD3	458.8	ARCHS4 Coexpression,581;Enrichr Queries,320;ReMap ChIP-seq,101;GTEx Coexpression,833	[illegible]
354	ZNF331	459	ARCHS4 Coexpression,652;Enrichr Queries,347;GTEx Coexpression,378	[illegible]
355	ZSCAN16	459.3	ARCHS4 Coexpression,119;Enrichr Queries,584;GTEx Coexpression,675	[illegible]
356	DRAP1	460	ARCHS4 Coexpression,6;Enrichr Queries,19;GTEx Coexpression,1355	[illegible]
357	MYBL1	461.5	Literature ChIP-seq,146;ARCHS4 Coexpression,114;Enrichr Queries,677;GTEx Coexpression,909	[illegible]
358	IRF5	462	ARCHS4 Coexpression,686;Enrichr Queries,376;GTEx Coexpression,324	[illegible]
359	GTF3A	462.3	ARCHS4 Coexpression,73;Enrichr Queries,77;GTEx Coexpression,1237	[illegible]
360	BCL6B	462.3	ARCHS4 Coexpression,295;Enrichr Queries,272;GTEx Coexpression,820	[illegible]
361	ZNF117	463.3	ARCHS4 Coexpression,141;Enrichr Queries,894;GTEx Coexpression,355	[illegible]
362	LTF	464	ARCHS4 Coexpression,683;GTEx Coexpression,245	[illegible]
363	MECOM	464.3	Literature ChIP-seq,56;ARCHS4 Coexpression,1094;GTEx Coexpression,243	[illegible]
364	ZNF888	465	GTEx Coexpression,465	[illegible]
365	IRX1	469	ARCHS4 Coexpression,688;Enrichr Queries,704;GTEx Coexpression,15	[illegible]
366	NFKB1	469.3	ARCHS4 Coexpression,1369;Enrichr Queries,175;ReMap ChIP-seq,189;GTEx Coexpression,144	[illegible]
367	NR3C2	469.3	ARCHS4 Coexpression,483;Enrichr Queries,734;GTEx Coexpression,191	[illegible]
368	ZFP90	470	ARCHS4 Coexpression,238;Enrichr Queries,370;GTEx Coexpression,802	[illegible]
369	ZNF652	471.5	Literature ChIP-seq,142;ARCHS4 Coexpression,511;Enrichr Queries,778;GTEx Coexpression,455	[illegible]
370	ZFP2	471.7	ARCHS4 Coexpression,337;Enrichr Queries,673;GTEx Coexpression,405	[illegible]
371	SPIB	471.8	ARCHS4 Coexpression,325;Enrichr Queries,640;ReMap ChIP-seq,258;GTEx Coexpression,664	[illegible]
372	SOX7	472.3	ARCHS4 Coexpression,1185;Enrichr Queries,215;GTEx Coexpression,17	[illegible]
373	ZNF354A	473.7	ARCHS4 Coexpression,157;Enrichr Queries,349;GTEx Coexpression,915	[illegible]
374	ZSCAN25	474.5	ARCHS4 Coexpression,674;GTEx Coexpression,275	[illegible]
375	NFIA	474.7	ARCHS4 Coexpression,620;Enrichr Queries,390;GTEx Coexpression,414	[illegible]
376	PCGF2	475.5	ARCHS4 Coexpression,660;ReMap ChIP-seq,291	[illegible]
377	HMG20A	476	ARCHS4 Coexpression,208;Enrichr Queries,198;GTEx Coexpression,1022	[illegible]
378	L3MBTL3	476	ARCHS4 Coexpression,213;GTEx Coexpression,739	[illegible]
379	PREB	476.7	ARCHS4 Coexpression,265;Enrichr Queries,182;GTEx Coexpression,983	[illegible]
380	ASCL2	478.7	ARCHS4 Coexpression,1051;Enrichr Queries,205;GTEx Coexpression,180	[illegible]
381	KLF11	479	ARCHS4 Coexpression,838;Enrichr Queries,311;GTEx Coexpression,288	[illegible]
382	ATF1	479.6	ARCHS4 Coexpression,625;ENCODE ChIP-seq,42;Enrichr Queries,344;ReMap ChIP-seq,161;GTEx Coexpression,1226	[illegible]
383	HLF	480	ARCHS4 Coexpression,335;Enrichr Queries,355;GTEx Coexpression,750	[illegible]
384	PLAGL1	480.3	ARCHS4 Coexpression,860;Enrichr Queries,193;GTEx Coexpression,388	[illegible]
385	ZNF430	480.3	ARCHS4 Coexpression,773;Enrichr Queries,581;GTEx Coexpression,87	[illegible]
386	ZFP57	481	ARCHS4 Coexpression,541;Enrichr Queries,114;GTEx Coexpression,788	[illegible]
387	ZSCAN26	481.5	ARCHS4 Coexpression,476;GTEx Coexpression,487	[illegible]
388	SP140L	483	ARCHS4 Coexpression,547;GTEx Coexpression,419	[illegible]
389	CENPT	483.7	ARCHS4 Coexpression,471;Enrichr Queries,266;GTEx Coexpression,714	[illegible]
390	HOXA2	484	Literature ChIP-seq,162;ARCHS4 Coexpression,784;Enrichr Queries,834;GTEx Coexpression,156	[illegible]
391	STAT6	485.8	Literature ChIP-seq,98;ARCHS4 Coexpression,1394;Enrichr Queries,13;GTEx Coexpression,438	[illegible]
392	ATF7	486.3	ARCHS4 Coexpression,795;Enrichr Queries,875;ReMap ChIP-seq,93;GTEx Coexpression,182	[illegible]
393	ZNF25	486.7	ARCHS4 Coexpression,513;Enrichr Queries,307;GTEx Coexpression,640	[illegible]
394	PRDM16	487.8	Literature ChIP-seq,144;ARCHS4 Coexpression,704;Enrichr Queries,986;GTEx Coexpression,117	[illegible]
395	E2F5	488.3	ARCHS4 Coexpression,255;Enrichr Queries,588;ReMap ChIP-seq,270;GTEx Coexpression,840	[illegible]
396	RBSN	489.5	ARCHS4 Coexpression,280;GTEx Coexpression,699	[illegible]
397	PRDM4	489.7	ARCHS4 Coexpression,564;Enrichr Queries,622;GTEx Coexpression,283	[illegible]
398	ERF	490	ARCHS4 Coexpression,395;Enrichr Queries,220;GTEx Coexpression,855	[illegible]
399	ZNF770	490.3	ARCHS4 Coexpression,324;Enrichr Queries,827;GTEx Coexpression,320	[illegible]
400	ZNF358	492.3	ARCHS4 Coexpression,745;Enrichr Queries,391;GTEx Coexpression,341	[illegible]
401	ZNF69	492.7	ARCHS4 Coexpression,430;Enrichr Queries,646;GTEx Coexpression,402	[illegible]
402	HEY2	495	ARCHS4 Coexpression,849;Enrichr Queries,396;GTEx Coexpression,240	[illegible]
403	AHDC1	496	ARCHS4 Coexpression,943;Enrichr Queries,477;GTEx Coexpression,68	[illegible]
404	SMAD1	497.6	Literature ChIP-seq,119;ARCHS4 Coexpression,970;Enrichr Queries,90;ReMap ChIP-seq,77;GTEx Coexpression,1232	[illegible]
405	MYBL2	498.5	Literature ChIP-seq,59;ARCHS4 Coexpression,400;ENCODE ChIP-seq,90;Enrichr Queries,1313;ReMap ChIP-seq,245;GTEx Coexpression,884	[illegible]
406	ELF2	498.7	ARCHS4 Coexpression,259;Enrichr Queries,630;GTEx Coexpression,607	[illegible]
407	ZNF280B	499.5	ARCHS4 Coexpression,554;Enrichr Queries,445	[illegible]
408	ZNF207	499.8	ARCHS4 Coexpression,1256;Enrichr Queries,229;ReMap ChIP-seq,125;GTEx Coexpression,389	[illegible]
409	ZNF251	500.7	ARCHS4 Coexpression,134;Enrichr Queries,545;GTEx Coexpression,823	[illegible]
410	ZNF512	501.3	ARCHS4 Coexpression,322;Enrichr Queries,364;GTEx Coexpression,818	[illegible]
411	ZNF860	501.5	ARCHS4 Coexpression,993;GTEx Coexpression,10	[illegible]
412	NFKB2	502	ARCHS4 Coexpression,1356;Enrichr Queries,93;ReMap ChIP-seq,109;GTEx Coexpression,450	[illegible]
413	ZBTB9	502	ARCHS4 Coexpression,132;Enrichr Queries,843;GTEx Coexpression,531	[illegible]
414	GLI2	502.7	ARCHS4 Coexpression,492;Enrichr Queries,655;GTEx Coexpression,361	[illegible]
415	FOXI3	503	ARCHS4 Coexpression,806;GTEx Coexpression,200	[illegible]
416	NR1H4	503	ARCHS4 Coexpression,881;Enrichr Queries,164;GTEx Coexpression,464	[illegible]
417	TCF7L1	503.7	ARCHS4 Coexpression,887;Enrichr Queries,472;GTEx Coexpression,152	[illegible]
418	NFATC4	504	ARCHS4 Coexpression,246;Enrichr Queries,437;GTEx Coexpression,829	[illegible]
419	ZNF532	504	ARCHS4 Coexpression,268;Enrichr Queries,357;GTEx Coexpression,887	[illegible]
420	TSHZ3	504.3	ARCHS4 Coexpression,253;Enrichr Queries,731;GTEx Coexpression,529	[illegible]
421	ZNF362	504.7	ARCHS4 Coexpression,1152;Enrichr Queries,241;GTEx Coexpression,121	[illegible]
422	SALL4	505	Literature ChIP-seq,47;ARCHS4 Coexpression,816;Enrichr Queries,384;GTEx Coexpression,773	[illegible]
423	POU2F1	505.5	ARCHS4 Coexpression,56;Enrichr Queries,804;ReMap ChIP-seq,280;GTEx Coexpression,882	[illegible]
424	BATF	507.2	ARCHS4 Coexpression,858;ENCODE ChIP-seq,111;Enrichr Queries,450;ReMap ChIP-seq,176;GTEx Coexpression,941	[illegible]
425	PDX1	507.4	Literature ChIP-seq,137;ARCHS4 Coexpression,831;Enrichr Queries,889;ReMap ChIP-seq,222;GTEx Coexpression,458	[illegible]
426	ZNF57	508	ARCHS4 Coexpression,808;Enrichr Queries,578;GTEx Coexpression,138	[illegible]
427	HMBOX1	509.3	ARCHS4 Coexpression,70;Enrichr Queries,1007;ReMap ChIP-seq,170;GTEx Coexpression,790	[illegible]
428	ZNF837	510.7	ARCHS4 Coexpression,525;Enrichr Queries,971;GTEx Coexpression,36	[illegible]
429	EBF2	511	ARCHS4 Coexpression,895;GTEx Coexpression,127	[illegible]
430	BCL6	512	Literature ChIP-seq,131;ARCHS4 Coexpression,1102;Enrichr Queries,81;ReMap ChIP-seq,134;GTEx Coexpression,1112	[illegible]
431	MEOX1	512.7	ARCHS4 Coexpression,717;Enrichr Queries,413;GTEx Coexpression,408	[illegible]
432	ZNF587B	513	ARCHS4 Coexpression,423;GTEx Coexpression,603	[illegible]
433	HOXB4	515.5	Literature ChIP-seq,52;ARCHS4 Coexpression,1286;Enrichr Queries,256;GTEx Coexpression,468	[illegible]
434	ETV1	515.8	ARCHS4 Coexpression,424;Enrichr Queries,480;ReMap ChIP-seq,135;GTEx Coexpression,1024	[illegible]
435	ARID3A	516	ARCHS4 Coexpression,1325;ENCODE ChIP-seq,43;Enrichr Queries,153;ReMap ChIP-seq,98;GTEx Coexpression,961	[illegible]
436	KLF8	516.7	ARCHS4 Coexpression,1207;Enrichr Queries,276;GTEx Coexpression,67	[illegible]
437	IRF8	517	Literature ChIP-seq,122;ARCHS4 Coexpression,1338;Enrichr Queries,305;GTEx Coexpression,303	[illegible]
438	PRDM8	518.3	ARCHS4 Coexpression,754;Enrichr Queries,466;GTEx Coexpression,335	[illegible]
439	SALL1	518.8	Literature ChIP-seq,82;ARCHS4 Coexpression,515;Enrichr Queries,601;GTEx Coexpression,877	[illegible]
440	MESP2	520.3	ARCHS4 Coexpression,968;Enrichr Queries,403;GTEx Coexpression,190	[illegible]
441	USF1	522.8	ARCHS4 Coexpression,1399;ENCODE ChIP-seq,80;Enrichr Queries,373;ReMap ChIP-seq,131;GTEx Coexpression,631	[illegible]
442	FOXN2	523.3	ARCHS4 Coexpression,436;Enrichr Queries,508;GTEx Coexpression,626	[illegible]
443	KMT2A	525.3	ARCHS4 Coexpression,536;ReMap ChIP-seq,269;GTEx Coexpression,771	[illegible]
444	GLI3	525.7	ARCHS4 Coexpression,673;Enrichr Queries,652;GTEx Coexpression,252	[illegible]
445	TET1	525.7	Literature ChIP-seq,126;ARCHS4 Coexpression,617;GTEx Coexpression,834	[illegible]
446	ZNF460	526	ARCHS4 Coexpression,235;Enrichr Queries,551;GTEx Coexpression,792	[illegible]
447	HOXD4	526.7	ARCHS4 Coexpression,251;Enrichr Queries,587;GTEx Coexpression,742	[illegible]
448	PAX6	527.4	Literature ChIP-seq,120;ARCHS4 Coexpression,649;Enrichr Queries,830;ReMap ChIP-seq,32;GTEx Coexpression,1006	[illegible]
449	TCF7	527.8	Literature ChIP-seq,74;ARCHS4 Coexpression,1193;Enrichr Queries,296;ReMap ChIP-seq,174;GTEx Coexpression,902	[illegible]
450	LCOR	530	ARCHS4 Coexpression,234;Enrichr Queries,1102;GTEx Coexpression,254	[illegible]
451	ZNF792	530.3	ARCHS4 Coexpression,632;Enrichr Queries,546;GTEx Coexpression,413	[illegible]
452	LHX2	530.5	ARCHS4 Coexpression,780;Enrichr Queries,520;ReMap ChIP-seq,124;GTEx Coexpression,698	[illegible]
453	IRX2	530.7	ARCHS4 Coexpression,1092;Enrichr Queries,306;GTEx Coexpression,194	[illegible]
454	E2F1	531.5	Literature ChIP-seq,4;ARCHS4 Coexpression,680;ENCODE ChIP-seq,105;Enrichr Queries,1257;ReMap ChIP-seq,205;GTEx Coexpression,938	[illegible]
455	IKZF1	531.5	Literature ChIP-seq,79;ARCHS4 Coexpression,1502;ENCODE ChIP-seq,30;Enrichr Queries,879;ReMap ChIP-seq,65;GTEx Coexpression,634	[illegible]
456	CDX4	531.7	ARCHS4 Coexpression,356;Enrichr Queries,912;GTEx Coexpression,327	[illegible]
457	TERF2	531.8	ARCHS4 Coexpression,972;Enrichr Queries,435;ReMap ChIP-seq,267;GTEx Coexpression,453	[illegible]
458	CREB5	532.7	ARCHS4 Coexpression,365;Enrichr Queries,159;GTEx Coexpression,1074	[illegible]
459	THAP2	533.7	ARCHS4 Coexpression,164;Enrichr Queries,690;GTEx Coexpression,747	[illegible]
460	MYRFL	534	ARCHS4 Coexpression,818;GTEx Coexpression,250	[illegible]
461	E2F7	534	Literature ChIP-seq,160;ARCHS4 Coexpression,129;Enrichr Queries,1294;ReMap ChIP-seq,46;GTEx Coexpression,1041	[illegible]
462	NR2C1	534	ARCHS4 Coexpression,171;Enrichr Queries,789;GTEx Coexpression,642	[illegible]
463	ATOH1	536	ARCHS4 Coexpression,373;Enrichr Queries,1051;GTEx Coexpression,184	[illegible]
464	BHLHE41	536	ARCHS4 Coexpression,527;GTEx Coexpression,545	[illegible]
465	DDIT3	536.3	ARCHS4 Coexpression,619;Enrichr Queries,133;GTEx Coexpression,857	[illegible]
466	FOXC1	537.3	ARCHS4 Coexpression,1329;Enrichr Queries,169;GTEx Coexpression,114	[illegible]
467	CREB3L3	538.3	ARCHS4 Coexpression,845;Enrichr Queries,324;GTEx Coexpression,446	[illegible]
468	HIC1	539.3	ARCHS4 Coexpression,461;Enrichr Queries,423;GTEx Coexpression,734	[illegible]
469	MSANTD4	539.5	ARCHS4 Coexpression,392;GTEx Coexpression,687	[illegible]
470	MAZ	539.6	ARCHS4 Coexpression,877;ENCODE ChIP-seq,91;Enrichr Queries,162;ReMap ChIP-seq,89;GTEx Coexpression,1479	[illegible]
471	ZNF394	540.7	ARCHS4 Coexpression,250;Enrichr Queries,409;GTEx Coexpression,963	[illegible]
472	DNTTIP1	541	ARCHS4 Coexpression,34;GTEx Coexpression,1048	[illegible]
473	ZNF658	541.5	ARCHS4 Coexpression,149;Enrichr Queries,934	[illegible]
474	ZNF91	541.7	ARCHS4 Coexpression,183;Enrichr Queries,186;GTEx Coexpression,1256	[illegible]
475	ZNF595	542.5	ARCHS4 Coexpression,185;Enrichr Queries,900	[illegible]
476	HIVEP3	542.7	ARCHS4 Coexpression,823;Enrichr Queries,558;GTEx Coexpression,247	[illegible]
477	HOXD13	543	Literature ChIP-seq,150;ARCHS4 Coexpression,659;Enrichr Queries,893;GTEx Coexpression,470	[illegible]
478	SP3	543.3	ARCHS4 Coexpression,984;Enrichr Queries,475;GTEx Coexpression,171	[illegible]
479	BATF3	543.7	ARCHS4 Coexpression,1048;Enrichr Queries,289;GTEx Coexpression,294	[illegible]
480	THAP8	544	ARCHS4 Coexpression,651;Enrichr Queries,636;GTEx Coexpression,345	[illegible]
481	ZNF426	544.3	ARCHS4 Coexpression,962;Enrichr Queries,625;GTEx Coexpression,46	[illegible]
482	NR4A3	544.3	ARCHS4 Coexpression,654;Enrichr Queries,322;GTEx Coexpression,657	[illegible]
483	ZFHX3	544.7	ARCHS4 Coexpression,1104;Enrichr Queries,284;GTEx Coexpression,246	[illegible]
484	NKX22	545	ARCHS4 Coexpression,538;GTEx Coexpression,552	[illegible]
485	ZNF24	545.3	ARCHS4 Coexpression,1006;Enrichr Queries,489;ReMap ChIP-seq,172;GTEx Coexpression,514	[illegible]
486	MTF1	545.5	ARCHS4 Coexpression,665;GTEx Coexpression,426	[illegible]
487	ZNF205	545.7	ARCHS4 Coexpression,668;Enrichr Queries,684;GTEx Coexpression,285	[illegible]
488	ZNF496	545.7	ARCHS4 Coexpression,908;Enrichr Queries,333;GTEx Coexpression,396	[illegible]
489	FOXI1	546	ARCHS4 Coexpression,1355;Enrichr Queries,238;GTEx Coexpression,45	[illegible]
490	ESR2	546.4	Literature ChIP-seq,69;ARCHS4 Coexpression,1586;Enrichr Queries,539;ReMap ChIP-seq,192;GTEx Coexpression,346	[illegible]
491	DLX5	547.3	ARCHS4 Coexpression,800;Enrichr Queries,525;GTEx Coexpression,317	[illegible]
492	IRX6	547.7	ARCHS4 Coexpression,557;Enrichr Queries,898;GTEx Coexpression,188	[illegible]
493	SLC2A4RG	547.7	ARCHS4 Coexpression,448;Enrichr Queries,411;GTEx Coexpression,784	[illegible]
494	HOXB13	548	ARCHS4 Coexpression,1034;Enrichr Queries,183;ReMap ChIP-seq,286;GTEx Coexpression,689	[illegible]
495	RARA	548.4	Literature ChIP-seq,124;ARCHS4 Coexpression,1318;Enrichr Queries,167;ReMap ChIP-seq,215;GTEx Coexpression,918	[illegible]
496	IRF9	549.3	ARCHS4 Coexpression,1295;Enrichr Queries,208;ReMap ChIP-seq,121;GTEx Coexpression,573	[illegible]
497	ZNF143	550	ARCHS4 Coexpression,736;ENCODE ChIP-seq,66;Enrichr Queries,532;ReMap ChIP-seq,187;GTEx Coexpression,1229	[illegible]
498	MTF2	550	Literature ChIP-seq,154;ARCHS4 Coexpression,112;GTEx Coexpression,1384	[illegible]
499	ARNTL	550.3	ARCHS4 Coexpression,439;Enrichr Queries,113;ReMap ChIP-seq,279;GTEx Coexpression,1370	[illegible]
500	ISL2	550.3	ARCHS4 Coexpression,593;Enrichr Queries,727;GTEx Coexpression,331	[illegible]
501	RBPJL	551	ARCHS4 Coexpression,802;Enrichr Queries,235;GTEx Coexpression,616	[illegible]
502	ARID5A	551.5	ARCHS4 Coexpression,491;GTEx Coexpression,612	[illegible]
503	ZBTB22	553	ARCHS4 Coexpression,772;Enrichr Queries,254;GTEx Coexpression,633	[illegible]
504	GLI1	553.3	Literature ChIP-seq,159;ARCHS4 Coexpression,1421;Enrichr Queries,464;GTEx Coexpression,169	[illegible]
505	ZBTB1	553.7	ARCHS4 Coexpression,608;Enrichr Queries,872;GTEx Coexpression,181	[illegible]
506	TEF	556	ARCHS4 Coexpression,561;Enrichr Queries,194;GTEx Coexpression,913	[illegible]
507	ZNF613	556.3	ARCHS4 Coexpression,304;Enrichr Queries,498;GTEx Coexpression,867	[illegible]
508	EOMES	556.8	Literature ChIP-seq,30;ARCHS4 Coexpression,1114;Enrichr Queries,433;ReMap ChIP-seq,250;GTEx Coexpression,957	[illegible]
509	NR1D2	558	ARCHS4 Coexpression,912;Enrichr Queries,100;GTEx Coexpression,662	[illegible]
510	ZNF121	558.3	ARCHS4 Coexpression,412;Enrichr Queries,1208;GTEx Coexpression,55	[illegible]
511	DMRTA2	558.7	ARCHS4 Coexpression,503;Enrichr Queries,1134;GTEx Coexpression,39	[illegible]
512	ZNF518B	561.7	ARCHS4 Coexpression,167;Enrichr Queries,786;GTEx Coexpression,732	[illegible]
513	HES6	565	ARCHS4 Coexpression,771;Enrichr Queries,110;GTEx Coexpression,814	[illegible]
514	BHLHE22	565.3	ARCHS4 Coexpression,623;ReMap ChIP-seq,193;GTEx Coexpression,880	[illegible]
515	IRX5	566.3	ARCHS4 Coexpression,864;Enrichr Queries,694;GTEx Coexpression,141	[illegible]
516	ARID5B	567.7	ARCHS4 Coexpression,451;Enrichr Queries,163;GTEx Coexpression,1089	[illegible]
517	ZNF275	568.3	ARCHS4 Coexpression,1199;Enrichr Queries,268;GTEx Coexpression,238	[illegible]
518	FOXP4	568.3	ARCHS4 Coexpression,796;Enrichr Queries,550;GTEx Coexpression,359	[illegible]
519	ZNF600	568.7	ARCHS4 Coexpression,685;Enrichr Queries,456;GTEx Coexpression,565	[illegible]
520	FOXE1	569.7	ARCHS4 Coexpression,207;Enrichr Queries,1069;GTEx Coexpression,433	[illegible]
521	ZNF296	569.7	ARCHS4 Coexpression,653;Enrichr Queries,382;GTEx Coexpression,674	[illegible]
522	GPBP1	570.5	ARCHS4 Coexpression,95;GTEx Coexpression,1046	[illegible]
523	ZBTB7B	571	ARCHS4 Coexpression,1466;Enrichr Queries,107;GTEx Coexpression,140	[illegible]
524	NFIX	571	ARCHS4 Coexpression,726;Enrichr Queries,45;GTEx Coexpression,942	[illegible]
525	ZBED6	571	ARCHS4 Coexpression,571	[illegible]
526	ZNF423	571.7	ARCHS4 Coexpression,346;Enrichr Queries,730;GTEx Coexpression,639	[illegible]

527	THAP12	573	ARCHS4 Coexpression,474;GTEx Coexpression,672	[illegible]
528	ZNF589	573.3	ARCHS4 Coexpression,363;Enrichr Queries,841;ReMap ChIP-seq,142;GTEx Coexpression,947	[illegible]
529	ZNF880	573.5	ARCHS4 Coexpression,454;GTEx Coexpression,693	[illegible]
530	GBX2	574.5	Literature ChIP-seq,157;ARCHS4 Coexpression,548;Enrichr Queries,902;GTEx Coexpression,691	[illegible]
531	ZNF219	575.3	ARCHS4 Coexpression,1039;Enrichr Queries,199;GTEx Coexpression,488	[illegible]
532	RUNX3	575.8	ARCHS4 Coexpression,1358;ENCODE ChIP-seq,113;Enrichr Queries,342;ReMap ChIP-seq,104;GTEx Coexpression,962	[illegible]
533	DZIP1	576.3	ARCHS4 Coexpression,139;Enrichr Queries,616;GTEx Coexpression,974	[illegible]
534	ZBTB38	577	ARCHS4 Coexpression,97;Enrichr Queries,146;GTEx Coexpression,1488	[illegible]
535	NR1I2	577.5	Literature ChIP-seq,128;ARCHS4 Coexpression,911;Enrichr Queries,623;GTEx Coexpression,648	[illegible]
536	CTCF	577.7	Literature ChIP-seq,106;ARCHS4 Coexpression,1449;ENCODE ChIP-seq,48;Enrichr Queries,693;ReMap ChIP-seq,190;GTEx Coexpression,980	[illegible]
537	ZNF804A	579	ARCHS4 Coexpression,274;Enrichr Queries,796;GTEx Coexpression,667	[illegible]
538	KLF15	579	ARCHS4 Coexpression,1273;Enrichr Queries,246;GTEx Coexpression,218	[illegible]
539	FOXO3	579.3	Literature ChIP-seq,72;ARCHS4 Coexpression,1084;Enrichr Queries,202;GTEx Coexpression,959	[illegible]
540	PEG3	580.5	ARCHS4 Coexpression,333;GTEx Coexpression,828	[illegible]
541	HOXB9	581.7	ARCHS4 Coexpression,1339;Enrichr Queries,213;GTEx Coexpression,193	[illegible]
542	TBX19	582	ARCHS4 Coexpression,336;Enrichr Queries,612;GTEx Coexpression,798	[illegible]
543	HDX	582.3	ARCHS4 Coexpression,417;Enrichr Queries,1170;GTEx Coexpression,160	[illegible]
544	FEV	582.3	ARCHS4 Coexpression,549;Enrichr Queries,822;GTEx Coexpression,376	[illegible]
545	HIVEP2	583.3	ARCHS4 Coexpression,767;Enrichr Queries,315;GTEx Coexpression,668	[illegible]
546	ZNF664	584	ARCHS4 Coexpression,1241;Enrichr Queries,137;GTEx Coexpression,374	[illegible]
547	ZFP28	585	ARCHS4 Coexpression,540;Enrichr Queries,554;GTEx Coexpression,661	[illegible]
548	ISX	585.7	ARCHS4 Coexpression,906;Enrichr Queries,718;GTEx Coexpression,133	[illegible]
549	IRF2	586.8	ARCHS4 Coexpression,1119;Enrichr Queries,443;ReMap ChIP-seq,114;GTEx Coexpression,671	[illegible]
550	ZNF133	588	ARCHS4 Coexpression,191;Enrichr Queries,851;GTEx Coexpression,722	[illegible]
551	ZNF181	588	ARCHS4 Coexpression,155;Enrichr Queries,1133;GTEx Coexpression,476	[illegible]
552	FOXM1	589.5	Literature ChIP-seq,37;ARCHS4 Coexpression,829;ENCODE ChIP-seq,9;Enrichr Queries,1379;ReMap ChIP-seq,30;GTEx Coexpression,1253	[illegible]
553	TRAFD1	592.7	ARCHS4 Coexpression,1032;Enrichr Queries,446;GTEx Coexpression,300	[illegible]
554	MYOD1	593	ARCHS4 Coexpression,642;ENCODE ChIP-seq,10;Enrichr Queries,736;ReMap ChIP-seq,129;GTEx Coexpression,1448	[illegible]
555	SOX10	593.3	ARCHS4 Coexpression,1160;Enrichr Queries,744;ReMap ChIP-seq,240;GTEx Coexpression,229	[illegible]
556	EBF3	593.3	ARCHS4 Coexpression,375;Enrichr Queries,996;GTEx Coexpression,409	[illegible]
557	TCF24	593.7	ARCHS4 Coexpression,319;Enrichr Queries,613;GTEx Coexpression,849	[illegible]
558	POU3F3	595.7	ARCHS4 Coexpression,342;Enrichr Queries,725;GTEx Coexpression,720	[illegible]
559	POU3F2	596.8	Literature ChIP-seq,140;ARCHS4 Coexpression,546;Enrichr Queries,1207;GTEx Coexpression,494	[illegible]
560	ZNF23	597	ARCHS4 Coexpression,189;Enrichr Queries,316;GTEx Coexpression,1286	[illegible]
561	ASCL3	597.3	ARCHS4 Coexpression,1036;Enrichr Queries,692;GTEx Coexpression,64	[illegible]
562	OLIG1	597.3	ARCHS4 Coexpression,921;Enrichr Queries,352;GTEx Coexpression,519	[illegible]
563	POGK	597.3	ARCHS4 Coexpression,996;Enrichr Queries,429;GTEx Coexpression,367	[illegible]
564	ZNF266	598	ARCHS4 Coexpression,935;Enrichr Queries,232;GTEx Coexpression,627	[illegible]
565	ZNF627	598.3	ARCHS4 Coexpression,316;Enrichr Queries,680;GTEx Coexpression,799	[illegible]
566	ZNF311	599	ARCHS4 Coexpression,410;Enrichr Queries,887;GTEx Coexpression,500	[illegible]
567	NFAT5	599.3	ARCHS4 Coexpression,636;Enrichr Queries,615;GTEx Coexpression,547	[illegible]
568	ZNF701	600.3	ARCHS4 Coexpression,296;Enrichr Queries,951;GTEx Coexpression,554	[illegible]
569	PATZ1	600.3	ARCHS4 Coexpression,277;Enrichr Queries,842;GTEx Coexpression,682	[illegible]
570	ZNF582	601	ARCHS4 Coexpression,398;Enrichr Queries,1020;GTEx Coexpression,385	[illegible]
571	SOX12	601.3	ARCHS4 Coexpression,633;Enrichr Queries,491;GTEx Coexpression,680	[illegible]
572	MNT	601.8	ARCHS4 Coexpression,1458;Enrichr Queries,496;ReMap ChIP-seq,28;GTEx Coexpression,425	[illegible]
573	SOX21	602.3	ARCHS4 Coexpression,1118;Enrichr Queries,605;GTEx Coexpression,84	[illegible]
574	SRY	604.3	Literature ChIP-seq,12;ARCHS4 Coexpression,1322;Enrichr Queries,981;GTEx Coexpression,102	[illegible]
575	ZNF680	604.3	ARCHS4 Coexpression,39;Enrichr Queries,1383;GTEx Coexpression,391	[illegible]
576	IRF4	604.6	ARCHS4 Coexpression,1100;ENCODE ChIP-seq,116;Enrichr Queries,811;ReMap ChIP-seq,152;GTEx Coexpression,844	[illegible]
577	NFIL3	608.3	ARCHS4 Coexpression,1310;Enrichr Queries,84;GTEx Coexpression,431	[illegible]
578	ZNF165	608.5	ARCHS4 Coexpression,355;Enrichr Queries,329;ReMap ChIP-seq,234;GTEx Coexpression,1516	[illegible]
579	MECP2	608.7	ARCHS4 Coexpression,941;Enrichr Queries,406;GTEx Coexpression,479	[illegible]
580	HNF1A	610.3	ARCHS4 Coexpression,893;Enrichr Queries,317;GTEx Coexpression,621	[illegible]
581	CENPBD1	611.5	ARCHS4 Coexpression,441;GTEx Coexpression,782	[illegible]
582	FOXB1	612	ARCHS4 Coexpression,531;Enrichr Queries,1046;GTEx Coexpression,259	[illegible]
583	GABPA	612	Literature ChIP-seq,29;ARCHS4 Coexpression,584;ENCODE ChIP-seq,87;Enrichr Queries,1162;ReMap ChIP-seq,206;GTEx Coexpression,1604	[illegible]
584	ZNF443	612.7	ARCHS4 Coexpression,299;Enrichr Queries,531;GTEx Coexpression,1008	[illegible]
585	E2F6	613	ARCHS4 Coexpression,781;ENCODE ChIP-seq,100;Enrichr Queries,432;ReMap ChIP-seq,207;GTEx Coexpression,1545	[illegible]
586	NCOA2	613.3	ARCHS4 Coexpression,1180;Enrichr Queries,1028;ReMap ChIP-seq,37;GTEx Coexpression,208	[illegible]
587	IKZF5	613.3	ARCHS4 Coexpression,124;Enrichr Queries,1105;GTEx Coexpression,611	[illegible]
588	AHRR	614	ARCHS4 Coexpression,81;GTEx Coexpression,1147	[illegible]
589	PURG	614.5	ARCHS4 Coexpression,578;GTEx Coexpression,651	[illegible]
590	PBX4	615.3	ARCHS4 Coexpression,545;Enrichr Queries,345;GTEx Coexpression,956	[illegible]
591	ZNF830	615.3	ARCHS4 Coexpression,184;Enrichr Queries,946;GTEx Coexpression,716	[illegible]
592	SOX8	616.7	ARCHS4 Coexpression,1139;Enrichr Queries,368;GTEx Coexpression,343	[illegible]
593	HAND1	617	ARCHS4 Coexpression,850;Enrichr Queries,732;GTEx Coexpression,269	[illegible]
594	ZBED5	622.5	ARCHS4 Coexpression,30;GTEx Coexpression,1215	[illegible]
595	ZSCAN21	622.7	ARCHS4 Coexpression,332;Enrichr Queries,485;GTEx Coexpression,1051	[illegible]
596	TBPL2	623.5	ARCHS4 Coexpression,951;GTEx Coexpression,296	[illegible]
597	PRDM5	623.5	Literature ChIP-seq,139;ARCHS4 Coexpression,945;Enrichr Queries,654;GTEx Coexpression,756	[illegible]
598	ZFP91	624.3	ARCHS4 Coexpression,99;Enrichr Queries,207;GTEx Coexpression,1567	[illegible]
599	AR	626.4	Literature ChIP-seq,95;ARCHS4 Coexpression,1158;Enrichr Queries,236;ReMap ChIP-seq,130;GTEx Coexpression,1513	[illegible]
600	ZNF442	626.7	ARCHS4 Coexpression,737;Enrichr Queries,422;GTEx Coexpression,721	[illegible]
601	LBX2	627.7	ARCHS4 Coexpression,677;Enrichr Queries,552;GTEx Coexpression,654	[illegible]
602	ZNF700	628.3	ARCHS4 Coexpression,225;Enrichr Queries,1245;GTEx Coexpression,415	[illegible]
603	THRA	629	Literature ChIP-seq,105;ARCHS4 Coexpression,718;Enrichr Queries,188;GTEx Coexpression,1505	[illegible]
604	CEBPG	629	ARCHS4 Coexpression,915;Enrichr Queries,210;ReMap ChIP-seq,235;GTEx Coexpression,1156	[illegible]
605	ATF6	630.7	ARCHS4 Coexpression,1052;Enrichr Queries,408;GTEx Coexpression,432	[illegible]
606	OLIG2	631.5	Literature ChIP-seq,115;ARCHS4 Coexpression,1151;Enrichr Queries,733;GTEx Coexpression,527	[illegible]
607	ZNF365	631.7	ARCHS4 Coexpression,853;Enrichr Queries,487;GTEx Coexpression,555	[illegible]
608	ZNF853	632	ARCHS4 Coexpression,729;GTEx Coexpression,535	[illegible]
609	TET3	632.7	ARCHS4 Coexpression,1274;ReMap ChIP-seq,296;GTEx Coexpression,328	[illegible]
610	RFX6	635.7	ARCHS4 Coexpression,380;Enrichr Queries,1005;GTEx Coexpression,522	[illegible]
611	HOXB3	636.3	ARCHS4 Coexpression,1097;Enrichr Queries,351;GTEx Coexpression,461	[illegible]
612	NFYC	636.3	ARCHS4 Coexpression,495;Enrichr Queries,156;GTEx Coexpression,1258	[illegible]
613	ETV3	636.7	ARCHS4 Coexpression,872;Enrichr Queries,716;GTEx Coexpression,322	[illegible]
614	TCF23	637.3	ARCHS4 Coexpression,1608;Enrichr Queries,88;GTEx Coexpression,216	[illegible]
615	ZNF184	637.3	ARCHS4 Coexpression,338;Enrichr Queries,870;GTEx Coexpression,704	[illegible]
616	CENPX	638	GTEx Coexpression,638	EIF6,SRD5A1
617	KLF13	639.3	ARCHS4 Coexpression,1171;Enrichr Queries,136;ReMap ChIP-seq,92;GTEx Coexpression,1158	[illegible]
618	SHOX2	639.7	ARCHS4 Coexpression,1031;Enrichr Queries,653;GTEx Coexpression,235	[illegible]
619	TFDP1	640.5	ARCHS4 Coexpression,830;Enrichr Queries,873;ReMap ChIP-seq,196;GTEx Coexpression,663	[illegible]
620	ASH1L	641	ARCHS4 Coexpression,354;Enrichr Queries,963;GTEx Coexpression,606	[illegible]
621	ZBTB16	641.8	ARCHS4 Coexpression,1302;Enrichr Queries,211;ReMap ChIP-seq,292;GTEx Coexpression,762	[illegible]
622	ZNF131	642	ARCHS4 Coexpression,425;Enrichr Queries,340;GTEx Coexpression,1161	[illegible]
623	FOXF2	642.3	ARCHS4 Coexpression,851;Enrichr Queries,602;GTEx Coexpression,474	[illegible]
624	BAZ2B	642.3	ARCHS4 Coexpression,290;Enrichr Queries,1097;GTEx Coexpression,540	[illegible]
625	CLOCK	642.4	Literature ChIP-seq,14;ARCHS4 Coexpression,318;Enrichr Queries,1119;ReMap ChIP-seq,213;GTEx Coexpression,1548	[illegible]
626	FOXH1	642.7	ARCHS4 Coexpression,1311;Enrichr Queries,492;GTEx Coexpression,125	[illegible]
627	MLXIPL	643	ARCHS4 Coexpression,714;Enrichr Queries,278;GTEx Coexpression,937	[illegible]
628	ZFX	643.4	Literature ChIP-seq,94;ARCHS4 Coexpression,408;Enrichr Queries,1323;ReMap ChIP-seq,239;GTEx Coexpression,1153	[illegible]
629	TBX10	643.7	ARCHS4 Coexpression,1086;Enrichr Queries,671;GTEx Coexpression,174	[illegible]
630	FOXK1	644.8	ARCHS4 Coexpression,1133;Enrichr Queries,500;ReMap ChIP-seq,293;GTEx Coexpression,653	[illegible]
631	TBX5	646	Literature ChIP-seq,109;ARCHS4 Coexpression,738;Enrichr Queries,1155;GTEx Coexpression,582	[illegible]
632	RREB1	648	ARCHS4 Coexpression,1312;Enrichr Queries,561;GTEx Coexpression,71	[illegible]
633	ZNF34	649	ARCHS4 Coexpression,276;Enrichr Queries,1077;GTEx Coexpression,594	[illegible]
634	TSC22D1	649	ARCHS4 Coexpression,607;Enrichr Queries,2;GTEx Coexpression,1338	[illegible]
635	THAP10	649.7	ARCHS4 Coexpression,904;Enrichr Queries,763;GTEx Coexpression,282	[illegible]
636	TCF20	649.7	ARCHS4 Coexpression,879;Enrichr Queries,1027;GTEx Coexpression,43	[illegible]
637	ZNF92	649.8	ARCHS4 Coexpression,244;Enrichr Queries,1302;ReMap ChIP-seq,157;GTEx Coexpression,896	[illegible]
638	DLX1	651	ARCHS4 Coexpression,762;Enrichr Queries,676;GTEx Coexpression,515	[illegible]
639	TRERF1	651	ARCHS4 Coexpression,1053;Enrichr Queries,801;GTEx Coexpression,99	[illegible]
640	NKRF	651.3	ARCHS4 Coexpression,700;Enrichr Queries,781;GTEx Coexpression,473	[illegible]
641	STAT4	651.5	Literature ChIP-seq,66;ARCHS4 Coexpression,901;Enrichr Queries,438;GTEx Coexpression,1201	[illegible]
642	SCMH1	652	ARCHS4 Coexpression,1074;GTEx Coexpression,230	[illegible]
643	ATF5	652.3	ARCHS4 Coexpression,1484;Enrichr Queries,30;GTEx Coexpression,443	[illegible]
644	SETDB1	652.7	Literature ChIP-seq,51;ARCHS4 Coexpression,1381;ENCODE ChIP-seq,114;Enrichr Queries,698;ReMap ChIP-seq,278;GTEx Coexpression,1394	[illegible]
645	ZNF254	652.7	ARCHS4 Coexpression,59;Enrichr Queries,1084;GTEx Coexpression,815	[illegible]
646	ARX	654	ARCHS4 Coexpression,521;Enrichr Queries,917;GTEx Coexpression,524	[illegible]
647	GATA5	654.7	ARCHS4 Coexpression,766;Enrichr Queries,512;GTEx Coexpression,686	[illegible]
648	CSRNP1	655	ARCHS4 Coexpression,634;GTEx Coexpression,676	[illegible]
649	CASZ1	655	ARCHS4 Coexpression,869;Enrichr Queries,806;GTEx Coexpression,290	[illegible]
650	ZNF48	656	ARCHS4 Coexpression,385;Enrichr Queries,852;GTEx Coexpression,731	[illegible]
651	LHX9	656.3	ARCHS4 Coexpression,360;Enrichr Queries,1076;GTEx Coexpression,533	[illegible]
652	ZNF558	656.3	ARCHS4 Coexpression,1245;Enrichr Queries,431;GTEx Coexpression,293	[illegible]
653	ZNF32	656.3	ARCHS4 Coexpression,288;Enrichr Queries,109;GTEx Coexpression,1572	[illegible]
654	PAX5	657.6	ARCHS4 Coexpression,1340;ENCODE ChIP-seq,76;Enrichr Queries,1088;ReMap ChIP-seq,137;GTEx Coexpression,647	[illegible]
655	DEAF1	657.8	ARCHS4 Coexpression,1137;Enrichr Queries,314;ReMap ChIP-seq,274;GTEx Coexpression,906	[illegible]
656	SP8	658.7	ARCHS4 Coexpression,715;Enrichr Queries,983;GTEx Coexpression,278	[illegible]
657	MYRF	660	ARCHS4 Coexpression,614;GTEx Coexpression,706	[illegible]
658	MTERF1	660.5	ARCHS4 Coexpression,553;GTEx Coexpression,768	[illegible]
659	ZNF83	660.8	ARCHS4 Coexpression,350;Enrichr Queries,586;ReMap ChIP-seq,263;GTEx Coexpression,1444	[illegible]
660	NKX61	661.5	ARCHS4 Coexpression,710;GTEx Coexpression,613	[illegible]
661	DLX2	661.7	ARCHS4 Coexpression,971;Enrichr Queries,547;GTEx Coexpression,467	[illegible]
662	AIRE	661.7	ARCHS4 Coexpression,1270;Enrichr Queries,350;GTEx Coexpression,365	[illegible]
663	HIVEP1	662	ARCHS4 Coexpression,989;Enrichr Queries,397;GTEx Coexpression,600	[illegible]
664	CTCFL	662	ARCHS4 Coexpression,1174;ENCODE ChIP-seq,98;Enrichr Queries,383;ReMap ChIP-seq,243;GTEx Coexpression,1412	[illegible]
665	NEUROG3	662.3	ARCHS4 Coexpression,811;Enrichr Queries,828;GTEx Coexpression,348	[illegible]
666	ZNF317	663	ARCHS4 Coexpression,842;Enrichr Queries,1011;GTEx Coexpression,136	[illegible]
667	TP73	664.3	ARCHS4 Coexpression,1297;Enrichr Queries,600;GTEx Coexpression,96	[illegible]
668	SATB2	664.3	ARCHS4 Coexpression,770;Enrichr Queries,907;GTEx Coexpression,316	[illegible]
669	ZNF706	664.7	ARCHS4 Coexpression,1002;Enrichr Queries,37;GTEx Coexpression,955	[illegible]
670	ZNF879	667	ARCHS4 Coexpression,111;GTEx Coexpression,1223	[illegible]
671	SP2	667.8	ARCHS4 Coexpression,1332;ENCODE ChIP-seq,57;Enrichr Queries,688;ReMap ChIP-seq,231;GTEx Coexpression,1031	[illegible]
672	ZNF14	668.3	ARCHS4 Coexpression,92;Enrichr Queries,410;GTEx Coexpression,1503	[illegible]
673	HOXA11	668.7	ARCHS4 Coexpression,889;Enrichr Queries,619;GTEx Coexpression,498	[illegible]
674	ZNF438	669	ARCHS4 Coexpression,302;Enrichr Queries,614;GTEx Coexpression,1091	[illegible]
675	OTX1	669.7	ARCHS4 Coexpression,702;Enrichr Queries,974;GTEx Coexpression,333	[illegible]
676	TBX6	670	ARCHS4 Coexpression,1342;Enrichr Queries,644;GTEx Coexpression,24	[illegible]
677	NKX23	670.5	ARCHS4 Coexpression,740;GTEx Coexpression,601	[illegible]
678	PAX2	671.3	ARCHS4 Coexpression,896;Enrichr Queries,1052;GTEx Coexpression,66	[illegible]
679	ZUP1	672.3	ARCHS4 Coexpression,427;Enrichr Queries,970;GTEx Coexpression,620	[illegible]
680	CBX2	672.6	ARCHS4 Coexpression,919;ENCODE ChIP-seq,92;Enrichr Queries,1032;ReMap ChIP-seq,271;GTEx Coexpression,1049	[illegible]
681	ZNF44	673	ARCHS4 Coexpression,444;Enrichr Queries,1018;GTEx Coexpression,557	[illegible]
682	CIC	674	ARCHS4 Coexpression,747;Enrichr Queries,343;GTEx Coexpression,932	[illegible]
683	SALL2	675.7	ARCHS4 Coexpression,815;Enrichr Queries,607;GTEx Coexpression,605	[illegible]
684	ZNF675	675.7	ARCHS4 Coexpression,589;Enrichr Queries,1034;GTEx Coexpression,404	[illegible]
685	ZNF764	675.7	ARCHS4 Coexpression,487;Enrichr Queries,921;GTEx Coexpression,619	[illegible]
686	ESRRG	676	ARCHS4 Coexpression,486;Enrichr Queries,1310;GTEx Coexpression,232	[illegible]
687	DMTF1	676	ARCHS4 Coexpression,137;Enrichr Queries,318;GTEx Coexpression,1573	[illegible]
688	CREM	676.2	Literature ChIP-seq,80;ARCHS4 Coexpression,1375;Enrichr Queries,270;ReMap ChIP-seq,146;GTEx Coexpression,1510	[illegible]
689	ZHX3	676.3	ARCHS4 Coexpression,443;Enrichr Queries,793;GTEx Coexpression,793	[illegible]
690	SP7	676.7	ARCHS4 Coexpression,942;Enrichr Queries,839;GTEx Coexpression,249	[illegible]
691	FOXK2	677	ARCHS4 Coexpression,1365;Enrichr Queries,507;ReMap ChIP-seq,85;GTEx Coexpression,751	[illegible]
692	HOXC9	677.3	Literature ChIP-seq,143;ARCHS4 Coexpression,1050;Enrichr Queries,310;GTEx Coexpression,1206	[illegible]
693	NFYA	677.6	ARCHS4 Coexpression,1439;ENCODE ChIP-seq,82;Enrichr Queries,482;ReMap ChIP-seq,266;GTEx Coexpression,1119	[illegible]
694	MSX1	677.7	ARCHS4 Coexpression,1376;Enrichr Queries,148;GTEx Coexpression,509	[illegible]
695	ZNF18	679	ARCHS4 Coexpression,65;Enrichr Queries,700;GTEx Coexpression,1272	[illegible]
696	ZIK1	679.7	ARCHS4 Coexpression,113;Enrichr Queries,840;GTEx Coexpression,1086	[illegible]
697	NKX28	680	ARCHS4 Coexpression,201;GTEx Coexpression,1159	[illegible]
698	NFATC2	680.3	ARCHS4 Coexpression,798;Enrichr Queries,393;GTEx Coexpression,850	[illegible]
699	SALL3	680.7	ARCHS4 Coexpression,237;Enrichr Queries,1064;GTEx Coexpression,741	[illegible]
700	TFAP4	681.3	ARCHS4 Coexpression,1524;Enrichr Queries,1031;ReMap ChIP-seq,87;GTEx Coexpression,83	[illegible]
701	MXD4	681.3	ARCHS4 Coexpression,934;Enrichr Queries,94;GTEx Coexpression,1016	[illegible]
702	ZNF334	682.7	ARCHS4 Coexpression,231;Enrichr Queries,1010;GTEx Coexpression,807	[illegible]

703	ZNF813	683	ARCHS4 Coexpression,939;GTEx Coexpression,427	CDC42,PKM,CAPNS1,CTR9,RPLP0
704	TFEC	683	ARCHS4 Coexpression,882;Enrichr Queries,569;GTEx Coexpression,598	CD74,CDKN1A,ANXA1,ARPC1B,SERPINE1,MYOF,LPL,TALDO1,CAPG,PTGS1,LGALS3,BASP1,CTSL,LCN2,IER3
705	SIX2	683	ARCHS4 Coexpression,327;Enrichr Queries,761;ReMap ChIP-seq,281;GTEx Coexpression,1363	CDKN1A,ANXA3,ARPC1B,SERPINE1,ATP1B1,WFDC2,FAM129B,COL1A1,PKM,ELF3,BASP1,MGP,MYH9,CYP1B1,TIMP3,ITGA6,MAP4,CALM1,SPTBN1,SOX4
706	SOX6	683.5	ARCHS4 Coexpression,69;Enrichr Queries,1012;ReMap ChIP-seq,100;GTEx Coexpression,1553	CLIC4,CDKN1A,CAPG,ACTB,RTN4,PRDX2,CCND2,CAPZB,CAPNS1,FUT9,FTH1,TMSB4X,CFL1,RACK1,TIMP3,JUNB,CLIC1,YWHAG,SOX4,S100A11,IER3,EIF5A,ANXA1,TNFRSF12A,PDZK1IP1,ATP1B1,RAB10,KLF6,PKM,GSTA4,MYH9,TAGLN2,ITGA6
707	PHOX2B	683.7	ARCHS4 Coexpression,505;Enrichr Queries,1279;GTEx Coexpression,267	COL1A1,UCHL1,CCND2,BASP1,ACTN1,RBP1,CYP1B1,TMSB10,PDLIM7,YWHAG,PTGS1
708	HBP1	684.5	ARCHS4 Coexpression,1309;Enrichr Queries,249;ReMap ChIP-seq,184;GTEx Coexpression,996	CLIC4,CDKN1A,SERPINE1,IQGAP1,MYL12A,PRDX2,LGALS3,FTH1,CFL1,BSG,RACK1,TIMP3,APOE,JUNB,CLIC1,SOX4,YWHAG,IER3,S100A10,EIF5A,HSPA8,ANXA1,GSN,JUP,TNFRSF12A,TSC22D1,ANXA2,RPS5,IDH1,EEF1G,KLF6,CLDN7,NUPR1,ITM2B
709	ZNF418	685	ARCHS4 Coexpression,22;Enrichr Queries,1206;GTEx Coexpression,827	EIF5A,CSTB,ACTN1,ARPC1B,FAM129B,COL1A1,CCND2,PKM,LAS1L,CAPNS1,FUT9,MYH9,TAGLN2,SOX4,S100A11,S100A10
710	ZKSCAN4	685.7	ARCHS4 Coexpression,362;Enrichr Queries,929;GTEx Coexpression,766	CSTB,CD74,CDKN1A,TSC22D1,CTR9,RPLP0,TALDO1,EEF1G,KLF6,RPL18A,CFL1,PMEPA1,SOX4,IER3
711	ZNF649	686.3	ARCHS4 Coexpression,422;Enrichr Queries,1216;GTEx Coexpression,421	LDHB,CLIC4,KLF6,CAPNS1,ACTN1,IDH1,DSTN,CYP1B1,TAGLN2,WFDC2,SOX4
712	TSHZ1	687.7	ARCHS4 Coexpression,575;Enrichr Queries,471;GTEx Coexpression,1017	CDKN1A,ANXA1,GSN,CADM4,TSC22D1,ATP1B1,KLF6,CCND2,EIF6,FXYD2,CYP1B1,PMEPA1,TIMP3,JUNB,CLIC1,SOX4,IER3
713	ZBTB21	688	ARCHS4 Coexpression,982;GTEx Coexpression,394	CDKN1A,KLF6,ARPC1B,CFL1,JUNB
714	NRF1	688	ARCHS4 Coexpression,1202;ENCODE ChIP-seq,72;Enrichr Queries,668;ReMap ChIP-seq,219;GTEx Coexpression,1279	ARF1,CDKN1A,CLIC4,MGST3,ACTB,MYL12A,RPL7,CDC42,PRDX2,CAPNS1,CTSL,CFL1,RACK1,RPL14,FLNA,PMEPA1,JUNB,YWHAG,PDLIM7,SOX4,RPS13,EIF5A,HSPA8,JUP,TNFRSF12A,TSC22D1,SRD5A1,RPS5,ADAM10,DSTN,EEF1G,RAB10,KLF6,PKM,ARPC2,CLDN7,MYH9
715	TFAP2B	688	ARCHS4 Coexpression,1089;Enrichr Queries,905;GTEx Coexpression,70	CERS3,JUP,LPL,KRT5,LRP2,RAB11A,COL1A1,UCHL1,CCND2,BASP1,KRT15,KRT14,MGP,LY6D,SDC1,CYP1B1,TIMP3,SFN
716	ZFP1	689.3	ARCHS4 Coexpression,587;Enrichr Queries,933;GTEx Coexpression,548	SLC12A2,HSPA8,CDKN1A,KLF6,TSC22D1,GSTA4,FUT9,ARPC1B,CALM1,JUNB,YWHAG,SOX4
717	ZNF74	693	ARCHS4 Coexpression,49;Enrichr Queries,1233;GTEx Coexpression,797	CDKN1A,MYOF,IQGAP1,MYL12A,FAM129B,UCHL1,CCND2,CAPNS1,GSTA4,MYH9,TAGLN2,CALM1,SOX4,S100A11,ITM2B
718	BACH2	694.8	ARCHS4 Coexpression,655;Enrichr Queries,752;ReMap ChIP-seq,238;GTEx Coexpression,1134	CSTB,CDKN1A,TPM4,ARPC1B,ACTN1,MYOF,ACTB,FAM129B,UCHL1,MMP14,KLF6,PKM,CCND2,TMSB4X,BSG,RACK1,MYH9,PMEPA1,TIMP3,ITGA6,JUNB,YWHAG,SOX4
719	CHCHD3	696	ARCHS4 Coexpression,284;GTEx Coexpression,1108	LDHB,RPL18A,RPS5,RPLP0,RPL11,RPS13,RPL7
720	ARNT2	697	ARCHS4 Coexpression,735;Enrichr Queries,353;GTEx Coexpression,1003	YWHAE,CDKN1A,CADM4,TSC22D1,SERPINE1,LPL,ATP1B1,CLU,COL1A1,UCHL1,CCND2,BASP1,CYP1B1,PMEPA1,TIMP3,APOE,CALM1,SOX4,YWHAG,IER3
721	ZFP30	697	ARCHS4 Coexpression,481;Enrichr Queries,696;GTEx Coexpression,914	CDKN1A,TSC22D1,SRD5A1,ATP1B1,KLF6,CCND2,LAS1L,CAPNS1,CAPZB,GSTA4,BSG,CYP1B1,ITGA6,JUNB,SOX4,IER3
722	LMX1B	700	ARCHS4 Coexpression,832;Enrichr Queries,1059;GTEx Coexpression,209	SLC12A2,CDKN1A,JUP,SERPINE1,KRT8,PRSS27,LRP2,EEF1G,COL1A1,BASP1,KRT15,KRT14,CYP1B1
723	ZNF235	700.3	ARCHS4 Coexpression,58;Enrichr Queries,950;GTEx Coexpression,1093	EIF5A,CDKN1A,TSC22D1,TPM4,ACTN1,FAM129B,CCND2,PKM,CAPNS1,BSG,PMEPA1,TIMP3,MYH9,FLNA,TAGLN2,APOE,SOX4
724	PURB	701	ARCHS4 Coexpression,193;GTEx Coexpression,1209	CLIC4,PKM,MYOF,MYH9,FLNA,IQGAP1,SPTBN1,YWHAG
725	ZSCAN9	702	ARCHS4 Coexpression,569;GTEx Coexpression,835	UCHL1,GSTA4,ARPC1B,RAC1,SOX4
726	SETDB2	704	ARCHS4 Coexpression,604;Enrichr Queries,813;GTEx Coexpression,695	YWHAE,CD74,CLIC4,CDKN1A,TSC22D1,RTN4,FAM129B,KLF6,CCND2,PKM,MGAT4A,BSG,NUPR1,JUNB
727	MESP1	704.3	ARCHS4 Coexpression,1251;Enrichr Queries,304;GTEx Coexpression,558	CSTB,CDKN1A,KRT8,KRT7,IQGAP1,WFDC2,COL1A1,UCHL1,KLF6,KRT19,ELF3,CLDN7,S100A6,CYP1B1,PMEPA1,APOE,JUNB,YWHAG,SOX4
728	ZNF639	704.8	ARCHS4 Coexpression,1534;Enrichr Queries,943;ReMap ChIP-seq,123;GTEx Coexpression,219	CLIC4,CDKN1A,SERPINE1,RTN4,ACTB,CDC42,FTH1,TMSB4X,CFL1,BSG,FLNA,SH3BGRL3,CLIC1,JUNB,YWHAG,IER3,EIF5A,HSPA8,RPS9,SLC6A19,ACTN1,RPS5,SLC34A1,DSTN,EEF1G,KLF6,CLDN7,S100A6,CTNNB1
729	POU2F3	705	ARCHS4 Coexpression,1615;Enrichr Queries,219;GTEx Coexpression,281	CSTB,CDKN1A,SERPINE1,LPL,WFDC2,PTGS1,CCND2,CYP1B1,CERS3,S100G,ANXA1,JUP,GPX3,PDZK1IP1,COL1A1,CLDN4,KLF6,KRT19,KRT18,ELF3,KRT15,KRT14,LCN2,SDC1
730	ZNF521	706.7	ARCHS4 Coexpression,195;Enrichr Queries,826;GTEx Coexpression,1099	YWHAE,ANXA1,ARPC1B,LPL,MYL12A,COL1A1,UCHL1,CCND2,GSTA4,MGP,CYP1B1,TIMP3,TAGLN2,SOX4,S100A11,S100A10
731	ZNF197	707.8	ARCHS4 Coexpression,61;Enrichr Queries,1284;ReMap ChIP-seq,248;GTEx Coexpression,1238	EIF5A,GSN,EGF,ACTN1,ARPC1B,RPL11,ADAM10,PDZK1IP1,RTN4,PRDX2,RPL18A,CAPNS1,CAPZB,GSTA4,TMSB4X,UCP2,BSG,PMEPA1,MYH9,TAGLN2,JUNB,PDLIM7,SOX4,S100A11
732	ZNF691	708	ARCHS4 Coexpression,526;Enrichr Queries,494;GTEx Coexpression,1104	HSPA8,CSTB,CLIC4,CDKN1A,ACTN1,RPS5,IDH1,ATP1B1,LDHB,KLF6,CCND2,EIF6,S100A6,PMEPA1,MYH9,SH3BGRL3,JUNB,IER3
733	ZNF737	708.5	ARCHS4 Coexpression,125;GTEx Coexpression,1292	EIF5A,PKM,CAPNS1,GSTA4,ACTN1,MYH9,FLNA,SOX4,FAM129B
734	THYN1	709	ARCHS4 Coexpression,535;GTEx Coexpression,883	LDHB,PRDX2,MYH9,FLNA,CALM1,RPL7
735	ZNF33A	709	ARCHS4 Coexpression,87;Enrichr Queries,1350;GTEx Coexpression,690	EIF5A,KLF6,PKM,CAPNS1,TPM4,TNFRSF12A,GSTA4,MGAT4A,ACTN1,CFL1,BSG,FAM129B
736	ESRRB	711	Literature ChIP-seq,121;ARCHS4 Coexpression,1112;Enrichr Queries,523;GTEx Coexpression,1088	S100G,CDKN1A,TSC22D1,ACTN1,MGST3,IDH1,SERPINE1,LPL,LRP2,ATP1B1,COL1A1,LGALS3,CCND2,KRT18,ELF3,BASP1,FUT9,FXYD2,CFL1,UCP2,CYP1B1,MYH9,ITM2B,YWHAG
737	ARID2	711.3	ARCHS4 Coexpression,219;Enrichr Queries,1029;ReMap ChIP-seq,111;GTEx Coexpression,1486	CDKN1A,CAPG,ACTB,MYL12A,PRDX2,CCND2,CAPNS1,CAPZB,FTH1,CFL1,BSG,RACK1,PMEPA1,S100A14,JUNB,SOX4,EIF5A,RPS9,TNFRSF12A,ANXA2,ADAM10,CP,EEF1G,KLF6,CLDN7,MYH9,CTNNB1,ITM2B
738	ONECUT1	712.5	ARCHS4 Coexpression,512;Enrichr Queries,908;ReMap ChIP-seq,246;GTEx Coexpression,1184	S100G,CDKN1A,MMP7,MGST3,MYOF,DSTN,ATP1B1,CLU,CP,FMO5,MYL12A,MMP14,KLF6,UCHL1,CCND2,KRT18,TMSB4X,RBP1,CYP1B1,MYH9,JUNB
739	ZNF852	712.5	ARCHS4 Coexpression,214;GTEx Coexpression,1211	PKM,CAPNS1,ACTN1,MAP4,YWHAG,RPL7,FAM129B
740	DMRT2	713.7	ARCHS4 Coexpression,1271;Enrichr Queries,772;GTEx Coexpression,98	MIOX,IL33,CDKN1A,ANXA1,GSN,GPX3,SLC34A1,LPL,LRP2,PDZK1IP1,COL1A1,CCND2,FXYD2,SPP1,CYP1B1
741	ZNF503	715	ARCHS4 Coexpression,1434;Enrichr Queries,144;GTEx Coexpression,567	CSTB,CDKN1A,SERPINE1,CLU,LGALS3,CCND2,RDH10,CYP1B1,PMEPA1,TIMP3,APOE,JUNB,PDLIM7,SOX4,IER3,S100A10,ANXA1,GSN,TNFRSF12A,TSC22D1,ACTN1,ATP1B1,COL1A1,KLF6,S100A6
742	ZNF248	715.3	ARCHS4 Coexpression,263;Enrichr Queries,993;GTEx Coexpression,890	CDKN1A,ATP1B1,KLF6,UCHL1,CCND2,LAS1L,GSTA4,CAPZB,FUT9,CYP1B1,TAGLN2,SOX4,S100A11
743	ZNF282	715.7	ARCHS4 Coexpression,857;Enrichr Queries,798;GTEx Coexpression,492	YWHAE,EIF5A,CDKN1A,RPS5,FAM129B,COL1A1,KLF6,CCND2,PKM,RPL18A,CAPNS1,CYP1B1,MYH9,JUNB,SOX4
744	ZNF624	716	ARCHS4 Coexpression,308;Enrichr Queries,1138;GTEx Coexpression,702	EIF5A,KLF6,GSTA4,CAPNS1,BSG,ADAM10,CYP1B1,MYH9,SOX4
745	ZNF711	717.7	ARCHS4 Coexpression,159;Enrichr Queries,666;GTEx Coexpression,1328	YWHAE,CDKN1A,ANXA1,ARPC1B,LPL,CLU,MYL12A,CCND2,CAPZB,GSTA4,BASP1,CYP1B1,TIMP3,ITGA6,CLIC1,SOX4,S100A11
746	EGR4	717.7	ARCHS4 Coexpression,381;Enrichr Queries,682;GTEx Coexpression,1090	CSTB,S100G,CDKN1A,TNFRSF12A,GPX3,TMEM176B,SERPINE1,TMEM176A,CLU,KLF6,CCND2,FXYD2,PMEPA1,CALM1,JUNB,IER3
747	TFEB	718.5	Literature ChIP-seq,97;ARCHS4 Coexpression,1478;Enrichr Queries,332;GTEx Coexpression,967	CSTB,CDKN1A,CAPG,MYL12A,FAM129B,LGALS3,FTH1,UCP2,CFL1,BSG,PMEPA1,APOE,JUNB,IER3,CD74,GSN,JUP,ADAM10,COL1A1,KLF6,ELF3,MGP,S100A6,MYH9,TAGLN2,MICU1
748	ATMIN	718.7	ARCHS4 Coexpression,1004;Enrichr Queries,326;GTEx Coexpression,826	YWHAE,HSPA8,CSTB,CLIC4,CDKN1A,TSC22D1,IDH1,SERPINE1,IQGAP1,ATP1B1,RTN4,KLF6,PKM,FTH1,PMEPA1,SH3BGRL3,CALM1,JUNB,SOX4,YWHAG
749	BRF2	719	ARCHS4 Coexpression,888;ReMap ChIP-seq,236;GTEx Coexpression,1033	CDKN1A,ARF1,RPS5,SERPINE1,CTR9,SLC12A1,KRT8,RAB10,KRT18,GSTA4,EIF6,CTSL,RACK1,SFN,MICU1,RPS13
750	HESX1	720.3	ARCHS4 Coexpression,712;Enrichr Queries,447;GTEx Coexpression,1002	CD74,S100G,CDKN1A,ANXA1,IDH1,SERPINE1,MYOF,LPL,RPL7,COL1A1,LDHB,KLF6,ELF3,RPL18A,TMSB4X,CYP1B1,NUPR1,APOE,IER3
751	TCF15	720.7	ARCHS4 Coexpression,1337;Enrichr Queries,330;GTEx Coexpression,495	CSTB,S100G,CDKN1A,TNFRSF12A,GPX3,MGST3,SERPINE1,LPL,MYL12A,WFDC2,COL1A1,CCND2,KRT18,S100A6,CYP1B1,TIMP3,NUPR1,APOE,JUNB,RPS13
752	ZBTB37	722.7	ARCHS4 Coexpression,331;Enrichr Queries,1021;GTEx Coexpression,816	EIF5A,CDKN1A,SRD5A1,ATP1B1,KLF6,CCND2,PKM,EIF6,CFL1,BSG,JUNB,ITM2B,SOX4
753	ZNF404	723	ARCHS4 Coexpression,245;Enrichr Queries,660;GTEx Coexpression,1264	CDKN1A,ANXA1,SERPINE1,IQGAP1,CLU,UCHL1,CAPNS1,GSTA4,FUT9,RDH10,CYP1B1,PMEPA1,MYH9,ITGA6,JUNB,SOX4,IER3
754	GZF1	723.3	ARCHS4 Coexpression,1324;Enrichr Queries,449;GTEx Coexpression,397	HSPA8,CSTB,CLIC4,CDKN1A,TSC22D1,RPL11,ATP1B1,RTN4,RPL7,KLF6,CCND2,PKM,RPL14,PMEPA1,JUNB,SOX4,YWHAG,IER3
755	CSRNP3	724	ARCHS4 Coexpression,537;GTEx Coexpression,911	UCHL1,GSTA4,FUT9,CALM1,RTN4
756	ZNF654	725.7	ARCHS4 Coexpression,211;Enrichr Queries,1217;GTEx Coexpression,749	EIF5A,CDC42,TMEM123,CDKN1A,KLF6,CAPNS1,GSTA4,CFL1,BSG,IQGAP1,JUNB
757	FOXJ2	726	ARCHS4 Coexpression,1009;Enrichr Queries,805;ReMap ChIP-seq,120;GTEx Coexpression,970	YWHAE,CDKN1A,SERPINE1,RPL11,IQGAP1,RTN4,ACTB,CCND2,FTH1,TMSB4X,BSG,JUNB,SOX4,HSPA8,RPS9,ANXA1,TSC22D1,GSTO1,RPS5,ACTN1,DSTN,ATP1B1,EEF1G,KLF6,KRT19,KRT15,MYH9,TAGLN2,NUPR1
758	THAP5	727	ARCHS4 Coexpression,774;Enrichr Queries,1276;GTEx Coexpression,131	LGALS3,CLIC4,RPL18A,JUP,EIF6,CAPNS1,BSG,PMEPA1,MYH9,CAPG,S100A10
759	THAP7	727	ARCHS4 Coexpression,656;Enrichr Queries,627;GTEx Coexpression,898	EIF5A,HSPA8,CSTB,RPS9,CDKN1A,ARF1,RPS5,RPLP0,ACTB,CDC42,RPL18A,EIF6,BSG,JUNB
760	GTF2IRD2B	727.3	ARCHS4 Coexpression,1015;Enrichr Queries,760;GTEx Coexpression,407	YWHAE,EIF5A,CDKN1A,ANXA1,GSN,SERPINE1,MYOF,PKM,CFL1,PMEPA1,NUPR1,JUNB,SOX4
761	SIM1	727.3	ARCHS4 Coexpression,681;Enrichr Queries,1280;GTEx Coexpression,221	COL1A1,MMP14,CDKN1A,ANXA2,GPX3,FXYD2,TMEM176B,MYOF,CFL1,CYP1B1
762	CSRNP2	728	ARCHS4 Coexpression,353;GTEx Coexpression,1103	UCHL1,CADM4,BASP1,MAP4,RTN4,YWHAG
763	AKAP8L	728.7	ARCHS4 Coexpression,1220;Enrichr Queries,261;GTEx Coexpression,705	HSPA8,CDKN1A,TSC22D1,ANXA2,ACTN1,RPS5,IQGAP1,ATP1B1,ACTB,KLF6,PKM,LAS1L,BSG,MYH9,FLNA,APOE,JUNB,SPTBN1,PDLIM7,IER3
764	ZNF335	730	ARCHS4 Coexpression,1186;Enrichr Queries,713;ReMap ChIP-seq,297;GTEx Coexpression,724	CDKN1A,KLF6,PKM,MGST3,FTH1,ACTN1,MYH9,MAP4,JUNB,SOX4,PDLIM7
765	ZNF562	730	ARCHS4 Coexpression,1196;Enrichr Queries,960;GTEx Coexpression,34	CSTB,CLIC4,ANXA1,TPM4,SRD5A1,PRSS27,PRSS22,ACTB,FAM129B,RAB10,KLF6,FTH1,LY6D,SFN,TRIM16,S100A14,SPRR1A,JUNB,IVL,S100A10,IER3
766	ZNF705E	731.5	ARCHS4 Coexpression,1359;GTEx Coexpression,104	KLF6,RPL18A,SRD5A1,RPL11,RACK1,RPL14,RPS13,RPL7
767	KMT2B	731.5	ARCHS4 Coexpression,1216;ReMap ChIP-seq,247	EIF5A,MIOX,VCP,ARF1,MMP7,RPS5,RPLP0,LRP2,MYL12A,CFL1,CLDN7,RACK1,MYH9
768	ZIC1	731.7	ARCHS4 Coexpression,421;Enrichr Queries,592;GTEx Coexpression,1182	YWHAE,CDKN1A,CADM4,LPL,CLU,CP,COL1A1,UCHL1,CCND2,BASP1,MGP,CYP1B1,TIMP3,APOE,YWHAG
769	ZNF114	732	ARCHS4 Coexpression,799;Enrichr Queries,780;GTEx Coexpression,617	CSTB,CDKN1A,ANXA3,SERPINE1,KRT7,LRP2,CLU,KLF6,CAPNS1,CYP1B1,PMEPA1,TIMP3,MYH9,FLNA
770	ASCL1	733	ARCHS4 Coexpression,866;Enrichr Queries,1128;ReMap ChIP-seq,162;GTEx Coexpression,776	YWHAE,CLIC4,CDKN1A,ACTB,FAM129B,PRDX2,CCND2,BSG,CYP1B1,JUNB,SOX4,IL33,SLC6A19,JUP,TNFRSF12A,KRT8,KRT7,KRT19,KRT18,ELF3,GSTA4,KRT15,S100A6,LY6D,SDC1
771	ZFP69B	733	ARCHS4 Coexpression,592;GTEx Coexpression,874	UCHL1,PKM,GSTA4,SOX4,ITM2B
772	ZNF195	733.3	ARCHS4 Coexpression,38;Enrichr Queries,1213;GTEx Coexpression,949	CDKN1A,ACTN1,IDH1,FAM129B,LDHB,PKM,CAPNS1,CAPZB,GSTA4,FUT9,MYH9,FLNA,TAGLN2,SOX4
773	ZNF71	733.7	ARCHS4 Coexpression,533;Enrichr Queries,1109;GTEx Coexpression,559	LGALS3,UCHL1,GSN,GSTA4,ACTN1,S100A6,TIMP3,IQGAP1,JUNB,PDLIM7,SOX4
774	PURA	734	ARCHS4 Coexpression,352;GTEx Coexpression,1116	FUT9,ATP1B1,CALM1,RTN4,SPTBN1,YWHAG
775	THAP4	734.3	ARCHS4 Coexpression,401;Enrichr Queries,251;GTEx Coexpression,1551	EIF5A,HSPA8,CSTB,CDKN1A,ANXA2,RPS5,IDH1,RPLP0,ATP1B1,ACTB,FAM129B,PRDX2,PKM,EIF6,CAPNS1,FTH1,BSG,RACK1,TAGLN2,JUNB
776	HOXC10	735	ARCHS4 Coexpression,902;Enrichr Queries,190;GTEx Coexpression,1113	EIF5A,CSTB,CDKN1A,ANXA1,GSN,GPX3,SERPINE1,KRT8,KRT7,LPL,COL1A1,KRT19,KRT18,MGP,S100A6,CYP1B1,SDC1,PMEPA1,TIMP3,NUPR1,RAC1,JUNB,IER3
777	ZNF529	735.3	ARCHS4 Coexpression,242;Enrichr Queries,1186;GTEx Coexpression,778	EIF5A,CDKN1A,CCND2,PKM,LAS1L,CAPNS1,CFL1,BSG,CYP1B1,MYH9,SOX4,FAM129B
778	HOXD3	735.3	ARCHS4 Coexpression,769;Enrichr Queries,1062;GTEx Coexpression,375	COL1A1,MMP14,CCND2,RBP1,SLC12A1,CYP1B1,LPL,TIMP3,LRP2,SOX4,RPL7
779	ZNF580	735.3	ARCHS4 Coexpression,452;Enrichr Queries,217;GTEx Coexpression,1537	CSTB,CDKN1A,RPS9,JUP,TNFRSF12A,TSC22D1,ACTB,COL1A1,KLF6,PKM,CAPNS1,FTH1,CFL1,BSG,S100A6,SH3BGRL3,TMSB10,JUNB,SOX4,PDLIM7,IER3,S100A10
780	ETV5	735.7	ARCHS4 Coexpression,1070;Enrichr Queries,128;GTEx Coexpression,1009	YWHAE,CSTB,CDKN1A,SERPINE1,LGALS3,CCND2,BASP1,CYP1B1,PMEPA1,TIMP3,APOE,JUNB,SOX4,IER3,ANXA1,GSN,TNFRSF12A,TSC22D1,ANXA2,ACTN1,ATP1B1,COL1A1,KLF6,S100A6,CTNNB1,ITGA6,TAGLN2
781	NCOA1	735.8	ARCHS4 Coexpression,828;Enrichr Queries,635;ReMap ChIP-seq,105;GTEx Coexpression,1375	CDKN1A,SERPINE1,PRSS27,IQGAP1,PRSS22,ACTB,MYL12A,PRDX2,CCND2,FTH1,TMSB4X,BSG,JUNB,S100A11,YWHAG,SOX4,SPTBN1,S100A10,EIF5A,HSPA8,JUP,TSC22D1,TNFRSF12A,ACTN1,KRT8,CP,CLDN4,KRT19,KLF6,KRT18,ELF3,EIF6,KRT15,MYH9,CTNNB1
782	ZNF572	736	ARCHS4 Coexpression,406;Enrichr Queries,1114;GTEx Coexpression,688	EEF1G,TMEM123,CLIC4,CDKN1A,ANXA1,KRT18,TSC22D1,ELF3,MGST3,RPLP0,KRT8,SOX4
783	ALX4	736	ARCHS4 Coexpression,969;Enrichr Queries,1199;GTEx Coexpression,40	SLC12A2,ANXA2,RPLP0,MYOF,RPL7,COL1A1,EEF1G,MMP14,CCND2,KRT14,RACK1,CYP1B1,RPS13
784	PRDM14	736.4	Literature ChIP-seq,41;ARCHS4 Coexpression,1005;Enrichr Queries,979;ReMap ChIP-seq,201;GTEx Coexpression,1456	ARF1,CDKN1A,RPLP0,WFDC2,ACTB,CCND2,RPL18A,CAPNS1,TMSB4X,CFL1,TIMP3,JUNB,TMSB10,SOX4,RPS13,SLC6A19,TNFRSF12A,TSC22D1,ANXA2,SLC34A1,MYOF,ATP1B1,EEF1G,CLDN4,ELF3,S100A6,TAGLN2,ITGA6,CSTB,LRP2,PTGS1,FAM129B,LDHB,FUT9,RDH10,RPL14,SPP1,FLNA,SFN,S100A14,PDLIM7,S100G,HSPA8,JUP,IDH1,PDZK1IP1,KLF6,EIF6,LAS1L,KRT14,CALM1
785	ZNF614	736.5	ARCHS4 Coexpression,108;GTEx Coexpression,1365	LDHB,CAPNS1,CAPZB,ACTN1,IDH1,ARPC1B,MYH9,TAGLN2,FAM129B
786	NKX62	736.5	ARCHS4 Coexpression,955;GTEx Coexpression,518	CADM4,MAP4,RTN4
787	TIGD2	736.7	ARCHS4 Coexpression,991;Enrichr Queries,360;GTEx Coexpression,859	CSTB,CDKN1A,ANXA1,ANXA3,IDH1,TMEM176A,RPLP0,RPSA,ATP1B1,KLF6,CCND2,BASP1,S100A6,LCN2,NUPR1,JUNB,SOX4,IER3,S100A10
788	GLYR1	737.3	ARCHS4 Coexpression,753;ReMap ChIP-seq,285;GTEx Coexpression,1174	EEF1B2,CD74,PKM,CAPNS1,SRD5A1,ARPC1B,RPLP0,MYH9,YWHAG
789	ZNF281	737.3	ARCHS4 Coexpression,1026;Enrichr Queries,218;GTEx Coexpression,968	HSPA8,CLIC4,CDKN1A,ANXA1,ACTN1,IDH1,SERPINE1,ADAM10,ATP1B1,ACTB,RTN4,LDHB,KLF6,PKM,BASP1,FTH1,S100A6,MYH9,CTNNB1,JUNB,SOX4,IER3,S100A10
790	YBX1	738	ARCHS4 Coexpression,705;Enrichr Queries,14;GTEx Coexpression,1495	YWHAE,CSTB,VCP,RPLP0,RPL11,ACTB,MYL12A,RTN4,RPL7,EEF1B2,LDHB,RPL18A,FTH1,CFL1,BSG,RACK1,RPL14,FLNA,TMSB10,CLIC1,RPS13,YWHAG,S100A10,EIF5A,HSPA8,RPS9,ANXA1,ANXA2,RPS5,RPSA,EEF1G,PKM,EIF6,ARPC2,S100A6,MYH9,TAGLN2,CALM1,ITM2B
791	ZNF585A	738.7	ARCHS4 Coexpression,241;Enrichr Queries,1065;GTEx Coexpression,910	EIF5A,CDKN1A,ANXA1,SRD5A1,SERPINE1,FAM129B,KLF6,PKM,LAS1L,CAPNS1,GSTA4,MYH9,JUNB
792	ZNF699	739	ARCHS4 Coexpression,1091;Enrichr Queries,754;GTEx Coexpression,372	CDKN1A,TPM4,MYOF,SERPINE1,IQGAP1,COL1A1,KLF6,CAPNS1,BSG,CYP1B1,TIMP3,MYH9,SOX4
793	STAT2	739.2	ARCHS4 Coexpression,1492;ENCODE ChIP-seq,118;Enrichr Queries,348;ReMap ChIP-seq,251;GTEx Coexpression,1487	CSTB,CLIC4,CDKN1A,SERPINE1,MYL12A,LGALS3,CCND2,TMSB4X,UCP2,JUNB,IER3,S100A10,CD74,RPS9,SLC6A19,ANXA1,ANXA2,MYOF,PDZK1IP1,CLDN4,KRT19,KLF6,PKM,KRT15,LCN2,MYH9,NUPR1
794	PHOX2A	739.3	ARCHS4 Coexpression,1044;Enrichr Queries,818;GTEx Coexpression,356	COL1A1,CSTB,S100G,CDKN1A,KRT19,CCND2,GSTA4,IDH1,RBP1,ISOC1,CYP1B1,JUNB
795	NEUROD1	739.5	ARCHS4 Coexpression,701;Enrichr Queries,1066;ReMap ChIP-seq,115;GTEx Coexpression,1076	SERPINE1,CAPG,ACTB,PRDX2,UCHL1,CCND2,FUT9,CFL1,BSG,RACK1,SH3BGRL3,APOE,JUNB,CLIC1,PDLIM7,SOX4,IER3,EIF5A,S100G,RPS9,TNFRSF12A,GPX3,TMEM176B,TMEM176A,EEF1G,KRT19,KRT15,FXYD2,CLDN7,TAGLN2
796	ETV3L	739.7	ARCHS4 Coexpression,661;Enrichr Queries,686;GTEx Coexpression,872	CDKN1A,MMP7,GSTO1,SERPINE1,MYOF,TALDO1,CTSL,TMSB4X,CYP1B1,PMEPA1,TIMP3,ITGA6,IER3,S100A10
797	MYPOP	740	ARCHS4 Coexpression,946;GTEx Coexpression,534	CAPNS1,CALM1,YWHAG
798	POU5F1B	741.5	ARCHS4 Coexpression,1405;GTEx Coexpression,78	EEF1G,RPS9,RPL18A,RPS5,RPLP0,RPL11,RACK1,RPL14
799	ZIC3	742	Literature ChIP-seq,49;ARCHS4 Coexpression,833;Enrichr Queries,926;GTEx Coexpression,1160	YWHAE,CDKN1A,ANXA1,JUP,IDH1,ACTN1,LPL,WFDC2,RTN4,COL1A1,LDHB,KLF6,PKM,BASP1,CYP1B1,SDC1,APOE
800	ZNF223	742.7	ARCHS4 Coexpression,224;Enrichr Queries,927;GTEx Coexpression,1077	CSTB,CDKN1A,IDH1,KLF6,PKM,CAPNS1,GSTA4,BASP1,CYP1B1,MYH9,FLNA,SOX4,IER3
801	TBX2	743	ARCHS4 Coexpression,1341;Enrichr Queries,478;GTEx Coexpression,410	CLIC4,CDKN1A,GSN,SERPINE1,KRT8,COL1A1,KRT18,MGP,TIMP3,MYH9,CYP1B1,PMEPA1,APOE,JUNB,SOX4,IER3
802	ZNF846	743.7	ARCHS4 Coexpression,478;Enrichr Queries,691;GTEx Coexpression,1062	EIF5A,CDKN1A,GSN,SERPINE1,CLU,COL1A1,LGALS3,KLF6,PKM,CAPNS1,BSG,CYP1B1,MYH9,JUNB,SOX4
803	ZNF134	744.3	ARCHS4 Coexpression,573;Enrichr Queries,596;GTEx Coexpression,1064	CDKN1A,TPM4,JUP,TSC22D1,ANXA3,IDH1,ARPC1B,LDHB,KLF6,BASP1,CYP1B1,JUNB,SOX4,IER3
804	ZNF264	745	ARCHS4 Coexpression,449;Enrichr Queries,1023;GTEx Coexpression,763	EIF5A,CDKN1A,KLF6,CCND2,PKM,SRD5A1,MGAT4A,CTR9,CFL1,BSG,SOX4,SPTBN1
805	ZNF644	745	ARCHS4 Coexpression,169;Enrichr Queries,1132;ReMap ChIP-seq,178;GTEx Coexpression,1501	CLIC4,ARPC1B,PRSS22,ACTB,RTN4,LGALS3,CAPNS1,CAPZB,CTSL,FTH1,TMSB4X,CFL1,UCP2,RPL14,TMSB10,YWHAG,SPTBN1,SOX4,SLC6A19,RPS9,SLC34A1,TMEM176B,TMEM176A,KLF6,GSTA4,CTNNB1,ITGA6,TAGLN2
806	ZNF226	745.3	ARCHS4 Coexpression,162;Enrichr Queries,712;GTEx Coexpression,1362	EIF5A,CDKN1A,ANXA1,GSN,TSC22D1,ACTN1,CLU,FAM129B,LGALS3,KLF6,PKM,CAPNS1,GSTA4,MYH9,FLNA,JUNB,SOX4,IER3
807	ZNF35	745.7	ARCHS4 Coexpression,580;Enrichr Queries,441;GTEx Coexpression,1216	CSTB,HSPA8,CDKN1A,TNFRSF12A,TSC22D1,IDH1,ATP1B1,COL1A1,LDHB,KLF6,CCND2,CYP1B1,TIMP3,ITGA6,JUNB,SOX4,ITM2B,IER3
808	ZFP14	746.3	ARCHS4 Coexpression,343;Enrichr Queries,699;GTEx Coexpression,1197	EIF5A,CDKN1A,TSC22D1,FAM129B,COL1A1,LGALS3,PKM,CAPNS1,BSG,CYP1B1,TIMP3,MYH9,ITGA6,JUNB,SOX4,IER3
809	ZGLP1	746.5	ARCHS4 Coexpression,232;GTEx Coexpression,1261	EGF,ARPC1B,TAGLN2,SH3BGRL3,PDZK1IP1,RAB11A,PTGS1
810	ZSCAN4	747.3	ARCHS4 Coexpression,1223;Enrichr Queries,735;GTEx Coexpression,284	CERS3,CDKN1A,JUP,SERPINE1,COL1A1,CCND2,ELF3,KRT14,SDC1,CYP1B1,JUNB,SOX4,RPS13,IER3
811	GSX2	748	ARCHS4 Coexpression,606;Enrichr Queries,1265;GTEx Coexpression,373	YWHAE,S100G,CADM4,CCND2,GSTA4,CLU,SPTBN1,SOX4
812	ZNF486	748.3	ARCHS4 Coexpression,1027;Enrichr Queries,302;GTEx Coexpression,916	CSTB,CDKN1A,ANXA1,ANXA2,MYOF,LRP2,ACTB,MYL12A,KLF6,CCND2,PKM,CAPNS1,BASP1,S100A6,CYP1B1,APOE,TMSB10,SOX4,IER3,S100A10
813	ZFP37	748.7	ARCHS4 Coexpression,721;Enrichr Queries,462;GTEx Coexpression,1063	CDKN1A,LPL,ATP1B1,CLU,COL1A1,UCHL1,KLF6,GSTA4,BASP1,CYP1B1,TIMP3,MYH9,ITGA6,SOX4,IER3
814	ZNF511	749	ARCHS4 Coexpression,706;Enrichr Queries,378;GTEx Coexpression,1163	EIF5A,HSPA8,CSTB,RPS9,TNFRSF12A,GSTO1,RPS5,ACTB,PKM,EIF6,CFL1,S100A6,RPL14,TAGLN2,CLIC1,IER3,S100A10
815	ZNF615	749.3	ARCHS4 Coexpression,182;Enrichr Queries,1087;GTEx Coexpression,979	EIF5A,ANXA1,FAM129B,KLF6,PKM,CAPNS1,CFL1,BSG,PMEPA1,CYP1B1,MYH9,FLNA,SOX4
816	HLX	749.3	ARCHS4 Coexpression,1111;Enrichr Queries,252;GTEx Coexpression,885	CD74,CDKN1A,ANXA1,GSN,ANXA2,ARPC1B,SERPINE1,MYOF,TALDO1,CAPG,PTGS1,COL1A1,LGALS3,MMP14,KLF6,CCND2,BASP1,S100A6,CYP1B1,PMEPA1,JUNB,IER3
817	ZNF107	749.3	ARCHS4 Coexpression,309;Enrichr Queries,1359;GTEx Coexpression,580	CDC42,CD74,CAPNS1,ACTN1,BSG,MAP4,SOX4,PDLIM7,FAM129B
818	NR2C2	749.6	ARCHS4 Coexpression,1298;ENCODE ChIP-seq,107;Enrichr Queries,1017;ReMap ChIP-seq,185;GTEx Coexpression,1141	EIF5A,HSPA8,VCP,CDKN1A,RPS5,IDH1,CTR9,RPLP0,RTN4,EEF1B2,PRDX2,KLF6,LAS1L,CAPZB,CFL1,CLDN7,RACK1,CTNNB1,APOE,JUNB,CLIC1,SPTBN1,IER3
819	THRB	750.3	ARCHS4 Coexpression,1250;Enrichr Queries,737;GTEx Coexpression,264	CDKN1A,ACTN1,SERPINE1,DSTN,LPL,ATP1B1,CLU,CCND2,PMEPA1,FLNA,CYP1B1,TIMP3,CALM1,SOX4
820	TET2	751.5	ARCHS4 Coexpression,1208;GTEx Coexpression,295	CDC42,ARPC2,IQGAP1,CLIC1
821	SCML4	752	ARCHS4 Coexpression,247;Enrichr Queries,884;GTEx Coexpression,1125	YWHAE,CD74,CDKN1A,GSN,ATP1B1,LGALS3,KLF6,CCND2,ARPC2,TMSB4X,MGAT4A,UCP2,PMEPA1,ITGA6,SH3BGRL3
822	ZNF146	752	ARCHS4 Coexpression,484;Enrichr Queries,430;GTEx Coexpression,1342	YWHAE,HSPA8,CLIC4,ANXA1,IDH1,ACTB,RTN4,LDHB,TMEM123,KLF6,KRT18,EIF6,BASP1,CFL1,RPL14,SOX4,ITM2B
823	HOXA7	753	ARCHS4 Coexpression,591;Enrichr Queries,420;GTEx Coexpression,1248	CSTB,CD74,CDKN1A,ANXA1,GSN,ANXA3,GPX3,RPS5,LPL,TALDO1,COL1A1,CCND2,RPL18A,CYP1B1,PMEPA1,TIMP3,SOX4,IER3
824	TBXT	754	ARCHS4 Coexpression,883;Enrichr Queries,1190;ReMap ChIP-seq,173;GTEx Coexpression,770	ARF1,CDKN1A,JUP,TNFRSF12A,KRT8,CP,ACTB,COL1A1,MMP14,KLF6,KRT19,UCHL1,KRT18,CCND2,CTSL,TMSB4X,FXYD2,RBP1,CYP1B1,PMEPA1,TRIM16,PDLIM7,S100A10
825	NANOGP8	754.5	ARCHS4 Coexpression,868;GTEx Coexpression,641	LDHB,HSPA8,BASP1,IDH1,CFL1
826	ZNF672	754.7	ARCHS4 Coexpression,523;Enrichr Queries,226;GTEx Coexpression,1515	CSTB,CDKN1A,MGST3,ARPC1B,ACTB,FAM129B,CAPZB,FTH1,CFL1,SH3BGRL3,JUNB,SOX4,IER3,S100A10,CD74,ANXA1,TNFRSF12A,TSC22D1,ACTN1,TALDO1,KLF6,PKM,S100A6,MYH9,TAGLN2
827	SIM2	755	ARCHS4 Coexpression,1556;Enrichr Queries,687;GTEx Coexpression,22	CSTB,CDKN1A,SERPINE1,PRSS27,PRSS22,FAM129B,CYP1B1,PMEPA1,SFN,S100A14,SOX4,S100A10,ANXA1,SRD5A1,KRT8,KRT7,KRT5,RAB10,COL1A1,KRT19,KRT15,LY6D,TRIM16,SPRR1A
828	ZBTB12	755.3	ARCHS4 Coexpression,817;Enrichr Queries,768;GTEx Coexpression,681	EIF5A,CDKN1A,COL1A1,EEF1G,LARS2,KLF6,CCND2,PKM,CYP1B1,NUPR1,JUNB,SOX4,IER3
829	ZNF229	756.5	ARCHS4 Coexpression,558;Enrichr Queries,955	COL1A1,CDKN1A,UCHL1,GSTA4,ARPC1B,CYP1B1,PMEPA1,APOE,SOX4
830	ZNF256	757	ARCHS4 Coexpression,556;Enrichr Queries,618;GTEx Coexpression,1097	CDKN1A,TNFRSF12A,IDH1,ATP1B1,FAM129B,LDHB,KLF6,CCND2,CAPNS1,BASP1,CYP1B1,TIMP3,JUNB,SOX4,IER3
831	HSF2	757.3	ARCHS4 Coexpression,28;Enrichr Queries,701;GTEx Coexpression,1543	HSPA8,CDKN1A,ANXA1,TSC22D1,ACTN1,ARPC1B,IQGAP1,ATP1B1,FAM129B,KLF6,UCHL1,CCND2,CAPNS1,CAPZB,GSTA4,MYH9,FLNA,TAGLN2,JUNB,SOX4,S100A11,IER3
832	ALX1	757.3	ARCHS4 Coexpression,1043;Enrichr Queries,1056;GTEx Coexpression,173	COL1A1,MMP14,CDKN1A,CCND2,KRT18,TPM4,ANXA2,EIF6,RPS5,MYOF,SLC12A1,CYP1B1
833	CAMTA1	757.7	ARCHS4 Coexpression,582;Enrichr Queries,706;GTEx Coexpression,985	CDKN1A,TSC22D1,ACTN1,IQGAP1,ATP1B1,ACTB,RTN4,KLF6,CCND2,FUT9,MYH9,CALM1,SOX4
834	TERF1	758.3	ARCHS4 Coexpression,1061;Enrichr Queries,918;ReMap ChIP-seq,229;GTEx Coexpression,825	HSPA8,CLIC4,CDKN1A,RPS5,MGST3,IDH1,RPL11,ADAM10,ATP1B1,ACTB,EEF1G,CDC42,LGALS3,LDHB,KLF6,CAPZB,SDC1,MYH9,TAGLN2,MAP4,CALM1,TMSB10,SOX4
835	FOXJ1	759	ARCHS4 Coexpression,1087;Enrichr Queries,69;GTEx Coexpression,1121	CSTB,CDKN1A,SERPINE1,CLU,WFDC2,LGALS3,UCP2,CYP1B1,TIMP3,APOE,JUNB,SOX4,IER3,IL33,CD74,S100G,ANXA1,GSN,TNFRSF12A,KRT8,CP,COL1A1,CLDN4,MMP14,KRT19,KRT18,ELF3,S100A6,LCN2
836	ZFP3	759.7	ARCHS4 Coexpression,489;Enrichr Queries,436;GTEx Coexpression,1354	EIF5A,CSTB,CD74,CDKN1A,ANXA1,LPL,RPSA,ATP1B1,CLU,PTGS1,COL1A1,KLF6,PKM,CFL1,BSG,CYP1B1,TIMP3,NUPR1,JUNB
837	ZNF710	760	ARCHS4 Coexpression,1397;Enrichr Queries,490;GTEx Coexpression,393	CSTB,CDKN1A,GSN,JUP,EGF,ACTN1,EEF1B2,KLF6,CCND2,MGAT4A,PMEPA1,MYH9,JUNB,SOX4,SPTBN1,IER3
838	ZIC2	763	ARCHS4 Coexpression,826;Enrichr Queries,442;GTEx Coexpression,1021	YWHAE,CDKN1A,ANXA1,IDH1,SERPINE1,LPL,CLU,COL1A1,LDHB,UCHL1,KLF6,CCND2,KRT18,CYP1B1,APOE,JUNB,SOX4
839	ZNF366	763.7	ARCHS4 Coexpression,1443;Enrichr Queries,461;GTEx Coexpression,387	IL33,CDKN1A,GSN,SERPINE1,MYOF,LPL,ATP1B1,KLF6,MGP,TIMP3,CYP1B1,SPTBN1,IER3
840	CEBPZ	764.2	ARCHS4 Coexpression,1072;ENCODE ChIP-seq,86;Enrichr Queries,1103;ReMap ChIP-seq,272;GTEx Coexpression,1288	CD74,HSPA8,VCP,JUP,RPS5,IDH1,RPLP0,RTN4,RPL7,LDHB,KLF6,RPL18A,EIF6,MYH9,SFN,APOE,CLIC1
841	HOXD1	765	ARCHS4 Coexpression,1362;Enrichr Queries,513;GTEx Coexpression,420	CDKN1A,RPS5,SERPINE1,COL1A1,UCHL1,KRT19,CCND2,KRT18,SPP1,CYP1B1,TIMP3,MAP4,RAC1,JUNB,SOX4,IER3
842	LEF1	765.8	ARCHS4 Coexpression,1236;Enrichr Queries,209;ReMap ChIP-seq,265;GTEx Coexpression,1353	CDKN1A,SERPINE1,LPL,CCND2,BASP1,CYP1B1,PMEPA1,TIMP3,APOE,TMSB10,JUNB,SOX4,IER3,CD74,ANXA1,TNFRSF12A,ANXA2,GSTO1,RPS5,ACTN1,SLC12A1,ATP1B1,COL1A1,KLF6,MGAT4A,RBP1,S100A6,ITGA6
843	RFX2	765.8	ARCHS4 Coexpression,1060;Enrichr Queries,221;ReMap ChIP-seq,283;GTEx Coexpression,1499	CDKN1A,SERPINE1,CLU,WFDC2,PTGS1,LGALS3,CCND2,SPP1,CYP1B1,PMEPA1,TIMP3,JUNB,PDLIM7,YWHAG,IER3,ANXA1,GSN,ANXA2,RPS5,SLC34A1,ACTN1,MYOF,KRT8,COL1A1,KLF6,KRT18,TAGLN2,CALM1
844	HSFY1	766	ARCHS4 Coexpression,1404;GTEx Coexpression,128	ANXA1,FUT9,PRSS27,ATP1B1,PRSS22,SPRR1A
845	SIX1	766	ARCHS4 Coexpression,859;Enrichr Queries,222;GTEx Coexpression,1217	CSTB,CDKN1A,ANXA1,GSN,ANXA2,SERPINE1,LPL,ATP1B1,CLU,COL1A1,LGALS3,KLF6,CCND2,KRT18,MGP,CYP1B1,TIMP3,NUPR1,MAP4,JUNB,SOX4,PDLIM7,IER3
846	ZNF775	766.7	ARCHS4 Coexpression,966;Enrichr Queries,530;GTEx Coexpression,804	CSTB,VCP,CDKN1A,RPS9,JUP,CAPG,ACTB,FAM129B,COL1A1,CCND2,RACK1,PMEPA1,JUNB,SOX4,IER3
847	MEF2B	766.8	ARCHS4 Coexpression,1265;Enrichr Queries,542;ReMap ChIP-seq,261;GTEx Coexpression,999	MIOX,CD74,CSTB,CDKN1A,RPS9,ARPC1B,KRT7,MYL12A,COL1A1,LGALS3,KLF6,CAPNS1,ARPC2,ELF3,TMSB4X,UCP2,PMEPA1,APOE,NUPR1,JUNB,IER3
848	HOXA5	767	ARCHS4 Coexpression,1514;Enrichr Queries,288;GTEx Coexpression,499	CDKN1A,ANXA1,GPX3,IDH1,SERPINE1,LPL,ATP1B1,COL1A1,KLF6,CCND2,KRT18,GSTA4,MGP,CYP1B1,TIMP3,APOE,JUNB,SOX4,IER3,S100A10
849	TBX21	767	ARCHS4 Coexpression,1226;Enrichr Queries,1043;ReMap ChIP-seq,150;GTEx Coexpression,649	CD74,CDKN1A,TPM4,MYL12A,EEF1G,PRDX2,TMEM123,LGALS3,KLF6,CCND2,CAPZB,TMSB4X,UCP2,CFL1,S100A6,RACK1,MYH9,FLNA,SH3BGRL3,TMSB10,JUNB,IER3
850	HAND2	767.3	ARCHS4 Coexpression,1058;Enrichr Queries,832;GTEx Coexpression,412	ANXA1,ACTN1,SERPINE1,LPL,COL1A1,CCND2,MGP,RBP1,ISOC1,FLNA,CYP1B1,TIMP3,PDLIM7
851	ZC3H8	767.8	ARCHS4 Coexpression,1013;Enrichr Queries,1072;ReMap ChIP-seq,53;GTEx Coexpression,933	CDKN1A,MGST3,PRSS22,FAM129B,CAPNS1,FTH1,BSG,RACK1,SFN,SH3BGRL3,S100A14,TMSB10,JUNB,S100A11,S100A10,ANXA2,TNFRSF12A,KRT8,COL1A1,KLF6,KRT19,KRT18,EIF6,ELF3,KRT15,RBP1,CLDN7,KRT14,S100A6,MYH9,ITGA6
852	ZMAT1	768.7	ARCHS4 Coexpression,75;Enrichr Queries,791;GTEx Coexpression,1440	EIF5A,CD74,CDKN1A,VCP,ARF1,ANXA1,GSN,TPM4,ACTN1,CLU,FAM129B,CCND2,PKM,EIF6,FUT9,CFL1,BSG,CYP1B1,TIMP3,ITGA6
853	HOXC4	769	ARCHS4 Coexpression,1294;Enrichr Queries,862;GTEx Coexpression,151	EIF5A,CDKN1A,TPM4,ANXA2,MYOF,COL1A1,MMP14,MGP,CYP1B1,PMEPA1,TIMP3,SOX4,IER3
854	ZBTB45	770	ARCHS4 Coexpression,508;Enrichr Queries,481;GTEx Coexpression,1321	EIF5A,CDKN1A,JUP,TNFRSF12A,TSC22D1,ACTB,KLF6,CAPNS1,CFL1,BSG,PMEPA1,MYH9,APOE,JUNB,SOX4,PDLIM7,IER3
855	DPF1	770	ARCHS4 Coexpression,626;Enrichr Queries,765;GTEx Coexpression,919	COL1A1,S100G,CDKN1A,UCHL1,CCND2,CADM4,BASP1,SERPINE1,PMEPA1,IQGAP1,CALM1,SOX4
856	STAT5B	770.5	ARCHS4 Coexpression,1571;Enrichr Queries,179;ReMap ChIP-seq,138;GTEx Coexpression,1194	VCP,CDKN1A,RPLP0,IQGAP1,CLU,ACTB,MYL12A,PRDX2,CCND2,TMSB4X,FTH1,CFL1,UCP2,RACK1,APOE,TMSB10,JUNB,YWHAG,SOX4,S100A10,HSPA8,ANXA1,GSN,JUP,ANXA2,RPS5,ACTN1,EEF1G,COL1A1,KLF6,PKM,ARPC2,MGAT4A,MYH9,CTNNB1,ITGA6,CALM1
857	ZNF816	771	ARCHS4 Coexpression,150;GTEx Coexpression,1392	PKM,CAPNS1,BSG,MYH9,FLNA,MAP4,YWHAG,FAM129B
858	ZHX2	771	ARCHS4 Coexpression,1485;Enrichr Queries,339;ReMap ChIP-seq,128;GTEx Coexpression,1132	CSTB,CDKN1A,CLIC4,SERPINE1,PRDX2,CCND2,BASP1,FTH1,CFL1,BSG,RACK1,PMEPA1,APOE,JUNB,CLIC1,SOX4,IER3,GSN,JUP,TNFRSF12A,TSC22D1,ANXA2,RPS5,SLC34A1,ACTN1,TALDO1,ATP1B1,EEF1G,COL1A1,KLF6,SDC1,ITGA6,NUPR1
859	ZBTB20	771.3	ARCHS4 Coexpression,341;Enrichr Queries,386;GTEx Coexpression,1587	EIF5A,CDKN1A,ANXA1,GSN,TSC22D1,LPL,ATP1B1,CLU,COL1A1,EEF1G,KLF6,CCND2,PKM,CAPZB,CFL1,BSG,PMEPA1,TIMP3,ITGA6,SOX4,SPTBN1
860	ZNF536	772.7	ARCHS4 Coexpression,393;Enrichr Queries,1361;GTEx Coexpression,564	UCHL1,CADM4,GSTA4,BASP1,FUT9,MAP4,LRP2,SOX4
861	ZXDB	772.7	ARCHS4 Coexpression,1150;Enrichr Queries,784;GTEx Coexpression,384	CDKN1A,TSC22D1,SERPINE1,IQGAP1,CP,KLF6,CCND2,CAPNS1,BSG,CYP1B1,PMEPA1,JUNB,SOX4
862	ZSCAN1	773	ARCHS4 Coexpression,358;Enrichr Queries,1370;GTEx Coexpression,591	YWHAE,UCHL1,GSTA4,TMEM176B,RBP1,CYP1B1,MYH9,IQGAP1
863	MLXIP	773	ARCHS4 Coexpression,1177;Enrichr Queries,294;GTEx Coexpression,848	HSPA8,CLIC4,CDKN1A,GSN,JUP,TSC22D1,EGF,ACTN1,LDHB,KLF6,CCND2,PKM,PMEPA1,MYH9,FLNA,ITGA6,JUNB,SOX4,SPTBN1,IER3
864	SOX3	775.3	ARCHS4 Coexpression,890;Enrichr Queries,923;GTEx Coexpression,513	YWHAE,COL1A1,UCHL1,CDKN1A,RPS9,ANXA1,KRT18,RACK1,CYP1B1,APOE,LRP2
865	ZNF75D	775.3	ARCHS4 Coexpression,1222;Enrichr Queries,514;GTEx Coexpression,590	YWHAE,CDKN1A,ANXA1,TSC22D1,IDH1,IQGAP1,CLU,KLF6,CCND2,TMSB4X,CYP1B1,PMEPA1,FLNA,SOX4,IER3
866	ZBTB2	775.3	ARCHS4 Coexpression,756;Enrichr Queries,556;GTEx Coexpression,1014	HSPA8,CSTB,CDKN1A,TNFRSF12A,ACTN1,ACTB,EEF1G,TMEM123,KLF6,PMEPA1,MYH9,JUNB,SOX4,IER3
867	THAP1	776	ARCHS4 Coexpression,1364;ENCODE ChIP-seq,112;Enrichr Queries,924;ReMap ChIP-seq,276;GTEx Coexpression,1204	CSTB,CLIC4,CDKN1A,ARF1,MGST3,MYL12A,PRDX2,RPL18A,CAPNS1,FTH1,BSG,JUNB,PDLIM7,SOX4,RPS13,HSPA8,TNFRSF12A,TSC22D1,SRD5A1,ACTN1,RPS5,TALDO1,LARS2,KLF6,ARPC2,LCN2,CTNNB1
868	MYOG	776.5	ARCHS4 Coexpression,1363;ENCODE ChIP-seq,39;Enrichr Queries,540;GTEx Coexpression,1164	CSTB,HSPA8,CDKN1A,CLIC4,ANXA1,SERPINE1,LPL,RTN4,FAM129B,COL1A1,LGALS3,KRT18,ELF3,MGP,PMEPA1,MYH9,APOE,CALM1,JUNB
869	IRF7	776.7	ARCHS4 Coexpression,1291;Enrichr Queries,369;GTEx Coexpression,670	CSTB,CD74,CDKN1A,ANXA1,GSN,ANXA2,ARPC1B,SERPINE1,CAPG,LGALS3,KLF6,UCP2,S100A6,LCN2,NUPR1,APOE,JUNB,IER3
870	ZNF98	777.7	ARCHS4 Coexpression,618;Enrichr Queries,1244;GTEx Coexpression,471	PKM,CAPNS1,EGF,FUT9,MGAT4A,RBP1,CYP1B1,TIMP3,MYH9,FLNA
871	ZNF174	778.7	ARCHS4 Coexpression,494;Enrichr Queries,853;GTEx Coexpression,989	CDKN1A,ARF1,JUP,TSC22D1,RPS5,ACTB,EEF1G,LGALS3,KLF6,CAPZB,CFL1,SDC1,SOX4,IER3
872	HOXA6	780.7	ARCHS4 Coexpression,976;Enrichr Queries,904;GTEx Coexpression,462	EEF1G,LGALS3,CSTB,CD74,MMP14,CDKN1A,MYOF,CYP1B1,PMEPA1,JUNB,SOX4,S100A10
873	ZNF124	781	ARCHS4 Coexpression,261;Enrichr Queries,949;GTEx Coexpression,1133	EIF5A,CDKN1A,ANXA1,TSC22D1,SRD5A1,CLU,PKM,CAPNS1,MGAT4A,CFL1,BSG,CYP1B1,MYH9,SOX4
874	LIN54	781	ARCHS4 Coexpression,741;GTEx Coexpression,821	RAB10,CAPNS1,CFL1,PDLIM7
875	ZNF208	781.3	ARCHS4 Coexpression,812;Enrichr Queries,1174;GTEx Coexpression,358	COL1A1,LDHB,CCND2,PKM,CAPNS1,GPX3,MGST3,CYP1B1,MYH9,LRP2
876	DACH2	781.3	ARCHS4 Coexpression,498;Enrichr Queries,1258;GTEx Coexpression,588	S100G,UCHL1,GSTA4,FUT9,LPL,MYH9,CALM1,YWHAG
877	ZNF616	781.7	ARCHS4 Coexpression,956;Enrichr Queries,661;GTEx Coexpression,728	CDKN1A,ANXA1,COL1A1,KLF6,CAPNS1,CLDN7,RACK1,CYP1B1,MYH9,APOE,JUNB,SOX4,IER3
878	DUX4	784	ARCHS4 Coexpression,1284;ReMap ChIP-seq,284	LARS2,CLDN4,ANXA3,SRD5A1,RPL11,PRSS22,MYL12A

879	FOXE3	784.7	ARCHS4 Coexpression,687;Enrichr Queries,1404;GTEx Coexpression,263	YWHAE,EIF5A,LDHB,CADM4,LAS1L,ITGA6,SPTBN1
880	ZNF280D	785.7	ARCHS4 Coexpression,752;Enrichr Queries,1079;GTEx Coexpression,526	EIF5A,CDKN1A,GSN,ARPC2,TSC22D1,SRD5A1,CFL1,ITGA6,SPTBN1
881	RAX	785.7	ARCHS4 Coexpression,749;Enrichr Queries,1335;GTEx Coexpression,273	YWHAE,CCND2,CYP1B1,CTNNB1,RPSA,LRP2,CP
882	ZNF708	787.7	ARCHS4 Coexpression,88;Enrichr Queries,1353;GTEx Coexpression,922	EIF5A,CADM4,PKM,CAPNS1,GSTA4,MGAT4A,ACTN1,BSG,MYH9,FLNA,SOX4,FAM129B
883	ZNF821	787.7	ARCHS4 Coexpression,497;Enrichr Queries,641;GTEx Coexpression,1225	CDKN1A,GSN,JUP,TSC22D1,ACTN1,IQGAP1,KLF6,UCHL1,PKM,GSTA4,ITGA6,JUNB,SOX4,IER3
884	FEZF1	788	ARCHS4 Coexpression,663;Enrichr Queries,1140;GTEx Coexpression,561	YWHAE,COL1A1,S100G,PRDX2,UCHL1,GSTA4,CYP1B1,LRP2
885	ZNF260	788.3	ARCHS4 Coexpression,156;Enrichr Queries,695;GTEx Coexpression,1514	SLC12A2,CDKN1A,ANXA1,TSC22D1,IDH1,ARPC1B,ATP1B1,LDHB,KLF6,CCND2,CAPNS1,CAPZB,GSTA4,MYH9,ITGA6,TAGLN2,SOX4
886	NEUROD6	788.7	ARCHS4 Coexpression,624;Enrichr Queries,1222;GTEx Coexpression,520	S100G,UCHL1,GSTA4,BASP1,FUT9,CALM1,YWHAG,SOX4
887	SON	789	ARCHS4 Coexpression,865;GTEx Coexpression,713	EIF6,CFL1,CTNNB1,TALDO1
888	ZBTB11	789	ARCHS4 Coexpression,788;Enrichr Queries,1187;ReMap ChIP-seq,273;GTEx Coexpression,908	HSPA8,VCP,TNFRSF12A,RPS5,SLC34A1,PRSS27,IQGAP1,MYL12A,KLF6,CAPNS1,EIF6,CFL1,RACK1,JUNB,YWHAG,PDLIM7
889	ZNF576	789.3	ARCHS4 Coexpression,875;Enrichr Queries,931;GTEx Coexpression,562	EIF5A,HSPA8,PKM,BSG,MYH9,FLNA,IQGAP1,CALM1,MICU1,JUNB,SOX4,IER3
890	CDC5L	792	ARCHS4 Coexpression,954;Enrichr Queries,282;GTEx Coexpression,1140	YWHAE,HSPA8,RPS9,VCP,RPS5,RPLP0,IQGAP1,ACTB,RPL7,PKM,EIF6,CAPZB,RACK1,RPL14,MYH9,CTNNB1,RPS13,SPTBN1,YWHAG,ITM2B
891	HOXC5	792.3	ARCHS4 Coexpression,886;Enrichr Queries,985;GTEx Coexpression,506	COL1A1,EIF5A,MMP14,KRT19,PKM,TNFRSF12A,MYOF,SERPINE1,KRT8,CYP1B1,TIMP3
892	TAL2	792.3	ARCHS4 Coexpression,594;Enrichr Queries,621;GTEx Coexpression,1162	IL33,S100G,CDKN1A,IDH1,LPL,LRP2,COL1A1,LGALS3,KRT18,GSTA4,CYP1B1,TIMP3,MYH9,SOX4
893	KLF16	792.7	ARCHS4 Coexpression,472;Enrichr Queries,497;GTEx Coexpression,1409	EIF5A,HSPA8,CSTB,CDKN1A,TNFRSF12A,ACTN1,ACTB,FAM129B,KLF6,PKM,CAPNS1,BSG,MYH9,JUNB,PDLIM7,IER3
894	HOXA4	793	ARCHS4 Coexpression,1328;Enrichr Queries,515;GTEx Coexpression,536	CDKN1A,ANXA1,GPX3,ACTN1,COL1A1,CCND2,RBP1,CYP1B1,PMEPA1,TIMP3,NUPR1,JUNB,PDLIM7,SOX4,IER3
895	SOX1	793	ARCHS4 Coexpression,518;Enrichr Queries,1266;GTEx Coexpression,595	YWHAE,UCHL1,CCND2,CADM4,GSTA4,BASP1,RACK1,SOX4
896	SPIC	793.3	ARCHS4 Coexpression,1491;Enrichr Queries,405;GTEx Coexpression,484	CD74,CDKN1A,ANXA1,ARPC1B,SERPINE1,LPL,ATP1B1,CP,LGALS3,MMP14,ELF3,UCP2,LCN2,NUPR1,APOE,JUNB,IER3
897	ZNF594	793.7	ARCHS4 Coexpression,152;Enrichr Queries,1120;GTEx Coexpression,1109	CDKN1A,SERPINE1,FAM129B,KLF6,CAPNS1,CAPZB,GSTA4,BSG,PMEPA1,MYH9,TAGLN2,SOX4,S100A11
898	TFDP2	793.7	ARCHS4 Coexpression,27;Enrichr Queries,771;GTEx Coexpression,1583	CDKN1A,ARF1,GSN,TSC22D1,ANXA2,TNFRSF12A,ACTN1,MYOF,IQGAP1,FAM129B,KLF6,CCND2,PKM,CAPNS1,CAPZB,MYH9,ITGA6,TAGLN2,JUNB,SOX4,S100A11,S100A10
899	ZNF112	794.5	ARCHS4 Coexpression,314;GTEx Coexpression,1275	UCHL1,GSTA4,ARPC1B,TAGLN2,SOX4,RTN4
900	ZNF420	796.7	ARCHS4 Coexpression,218;Enrichr Queries,1054;GTEx Coexpression,1118	CDKN1A,CCND2,ANXA1,CAPNS1,GSTA4,ACTN1,CYP1B1,MYH9,FLNA,JUNB,SOX4,FAM129B
901	ZNF841	797	ARCHS4 Coexpression,180;Enrichr Queries,766;GTEx Coexpression,1445	EIF5A,CSTB,CDKN1A,RPS9,ANXA1,SERPINE1,RPSA,COL1A1,KLF6,CAPNS1,CAPZB,CFL1,BSG,FLNA,RAC1,JUNB,IER3
902	ZNF346	797.7	ARCHS4 Coexpression,186;Enrichr Queries,797;GTEx Coexpression,1410	HSPA8,CSTB,CLIC4,CDKN1A,MYOF,IQGAP1,FAM129B,LGALS3,KLF6,MMP14,CCND2,CAPNS1,MYH9,JUNB,PDLIM7,YWHAG
903	ZNF682	798	ARCHS4 Coexpression,110;Enrichr Queries,891;GTEx Coexpression,1393	CDKN1A,ANXA3,ACTN1,FAM129B,KLF6,CCND2,PKM,CAPNS1,GSTA4,BASP1,CYP1B1,MYH9,FLNA,TAGLN2,SOX4
904	ZNF286A	798	ARCHS4 Coexpression,154;Enrichr Queries,836;GTEx Coexpression,1404	YWHAE,IDH1,LDHB,UCHL1,KLF6,CCND2,CAPNS1,GSTA4,CTNNB1,MYH9,ITGA6,TRIM16,SOX4,IER3
905	ZNF565	799.7	ARCHS4 Coexpression,321;Enrichr Queries,997;GTEx Coexpression,1081	KLF6,ANXA1,CAPNS1,GSTA4,CYP1B1,PMEPA1,MYH9,FLNA,JUNB,SOX4,IER3
906	PAX1	800	ARCHS4 Coexpression,458;Enrichr Queries,1334;GTEx Coexpression,608	TSC22D1,KRT15,CYP1B1,CAPG,NUPR1,LRP2,ATP1B1
907	ZNF350	800	ARCHS4 Coexpression,707;Enrichr Queries,517;GTEx Coexpression,1176	CSTB,CDKN1A,ANXA1,TSC22D1,ANXA3,LGALS3,KLF6,PKM,BSG,CYP1B1,PMEPA1,MAP4,JUNB,SOX4,YWHAG,IER3
908	PBX2	800.5	ARCHS4 Coexpression,1429;Enrichr Queries,335;ReMap ChIP-seq,136;GTEx Coexpression,1302	CSTB,CDKN1A,CAPG,ACTB,RTN4,PRDX2,CCND2,FTH1,TMSB4X,CFL1,BSG,UCP2,JUNB,CLIC1,YWHAG,SOX4,PDLIM7,IER3,HSPA8,JUP,TNFRSF12A,TSC22D1,ACTN1,EEF1G,KLF6,KRT19,MMP14,PKM,KRT15,MYH9
909	OTP	802.7	ARCHS4 Coexpression,493;Enrichr Queries,1339;GTEx Coexpression,576	UCHL1,CDKN1A,GSTA4,BASP1,TMSB10,YWHAG,SOX4
910	ETV2	802.8	Literature ChIP-seq,84;ARCHS4 Coexpression,1464;Enrichr Queries,212;GTEx Coexpression,1451	CSTB,SPRR2F,CDKN1A,SERPINE1,RPL7,CDC42,LGALS3,TMEM123,CCND2,RDH10,CYP1B1,APOE,JUNB,SOX4,CERS3,S100G,CD74,TMEM176B,TMEM176A,KRT8,KRT7,COL1A1,CLDN4,KLF6,KRT19,KRT18,ELF3,CLDN7,S100A6,SDC1,IVL
911	ZSCAN18	803	ARCHS4 Coexpression,944;Enrichr Queries,262;GTEx Coexpression,1203	CSTB,CDKN1A,GSN,CADM4,TSC22D1,GPX3,ATP1B1,CLU,COL1A1,UCHL1,KLF6,CCND2,BASP1,CYP1B1,PMEPA1,TIMP3,APOE,JUNB,SOX4,IER3
912	GSC	803	ARCHS4 Coexpression,813;Enrichr Queries,1193;GTEx Coexpression,403	GSN,KRT18,IDH1,KRT8,LPL,TIMP3,CYP1B1,JUNB
913	CRX	805.3	Literature ChIP-seq,93;ARCHS4 Coexpression,1603;Enrichr Queries,590;GTEx Coexpression,935	EIF5A,SLC12A2,CSTB,S100G,CDKN1A,CADM4,SRD5A1,MGST3,TMEM176A,SERPINE1,LPL,CP,ACTB,COL1A1,LARS2,KRT18,APOE,CALM1,JUNB,SOX4
914	ZNF180	805.3	ARCHS4 Coexpression,212;Enrichr Queries,1220;GTEx Coexpression,984	LDHB,CDKN1A,CCND2,CAPNS1,CAPZB,CTR9,BSG,MYH9,MAP4,JUNB,FAM129B
915	ZFP64	805.7	ARCHS4 Coexpression,1607;Enrichr Queries,634;GTEx Coexpression,176	CERS3,CDKN1A,JUP,TSC22D1,ACTN1,RPLP0,ACTB,KLF6,PKM,EIF6,KRT14,PMEPA1,MYH9,ITGA6,JUNB,SOX4
916	ZNF286B	807	ARCHS4 Coexpression,586;GTEx Coexpression,1028	CAPNS1,BSG,MYH9,SOX4
917	WIZ	807	ARCHS4 Coexpression,600;Enrichr Queries,488;GTEx Coexpression,1333	HSPA8,CDKN1A,JUP,ACTN1,ACTB,FAM129B,CAPNS1,BSG,MYH9,FLNA,MAP4,JUNB,SPTBN1,PDLIM7
918	ZFP92	807.3	ARCHS4 Coexpression,1521;Enrichr Queries,495;GTEx Coexpression,406	CSTB,CDKN1A,JUP,GPX3,LPL,LGALS3,CCND2,ELF3,TMSB4X,CLDN7,LCN2,SDC1,CYP1B1,TIMP3,NUPR1,JUNB
919	ZNF669	808.3	ARCHS4 Coexpression,574;Enrichr Queries,998;GTEx Coexpression,853	CDC42,CDKN1A,KLF6,PKM,TSC22D1,CAPNS1,MYH9,ATP1B1,JUNB,IER3,FAM129B
920	REL	809.7	ARCHS4 Coexpression,1017;Enrichr Queries,808;GTEx Coexpression,604	YWHAE,CD74,CDKN1A,SRD5A1,SERPINE1,CDC42,LGALS3,KLF6,CCND2,ARPC2,JUNB,SOX4,IER3
921	ZNF467	810	ARCHS4 Coexpression,1320;Enrichr Queries,122;GTEx Coexpression,988	CSTB,CDKN1A,CLU,PTGS1,LGALS3,CCND2,BASP1,UCP2,CYP1B1,PMEPA1,TIMP3,APOE,JUNB,SOX4,IER3,CD74,GSN,JUP,TSC22D1,TALDO1,ATP1B1,COL1A1,KLF6,S100A6,NUPR1,ITM2B
922	C11ORF95	810	GTEx Coexpression,810	ARPC2
923	ZBTB18	811.5	ARCHS4 Coexpression,432;GTEx Coexpression,1191	CADM4,FUT9,CALM1,RTN4,YWHAG
924	ZNF385D	811.7	ARCHS4 Coexpression,975;Enrichr Queries,1325;GTEx Coexpression,135	CLIC4,BASP1,SRD5A1,ACTN1,MGP,PMEPA1,MYH9,FLNA,CYP1B1,PTGS1
925	ZKSCAN7	812	ARCHS4 Coexpression,855;GTEx Coexpression,769	PKM,CAPNS1,MYH9,RAC1
926	ETV7	812.7	ARCHS4 Coexpression,1402;Enrichr Queries,888;GTEx Coexpression,148	CERS3,CSTB,CD74,CDKN1A,JUP,SERPINE1,KLF6,RPL18A,EIF6,KRT14,SDC1,NUPR1,IER3
927	HES5	813	ARCHS4 Coexpression,1076;Enrichr Queries,617;GTEx Coexpression,746	YWHAE,CSTB,CDKN1A,COL1A1,CCND2,RACK1,CYP1B1,TIMP3,NUPR1,APOE,JUNB,SOX4,RPS13,S100A10
928	MEIS3	813	ARCHS4 Coexpression,576;Enrichr Queries,367;GTEx Coexpression,1496	CDKN1A,GSN,CADM4,SERPINE1,CLU,COL1A1,KLF6,UCHL1,CCND2,BASP1,CYP1B1,PMEPA1,TIMP3,APOE,JUNB,SOX4,PDLIM7,YWHAG,IER3
929	TIGD6	813.3	ARCHS4 Coexpression,922;Enrichr Queries,800;GTEx Coexpression,718	CDKN1A,KLF6,ANXA2,CTR9,SERPINE1,MYOF,MYH9,PDLIM7,SPTBN1,IER3,PTGS1
930	ZNF140	815	ARCHS4 Coexpression,84;Enrichr Queries,773;GTEx Coexpression,1588	EIF5A,CLIC4,CDKN1A,RPS9,TSC22D1,ACTN1,FAM129B,KLF6,CCND2,PKM,CAPNS1,GSTA4,MYH9,FLNA,ITGA6,JUNB,SOX4,YWHAG,IER3
931	ZNF273	815.7	ARCHS4 Coexpression,791;Enrichr Queries,1377;GTEx Coexpression,279	CERS3,PKM,JUP,CAPNS1,KRT14,SDC1,MYH9
932	ZNF655	817.3	ARCHS4 Coexpression,621;Enrichr Queries,425;GTEx Coexpression,1406	YWHAE,HSPA8,CDKN1A,ANXA1,GSN,ANXA2,SERPINE1,ACTB,RTN4,KLF6,PKM,MGAT4A,PMEPA1,CALM1,JUNB,IER3,S100A10
933	TOPORS	819	ARCHS4 Coexpression,931;GTEx Coexpression,707	CAPNS1,CTR9,CFL1
934	ONECUT2	820	ARCHS4 Coexpression,520;Enrichr Queries,838;GTEx Coexpression,1102	COL1A1,CDKN1A,UCHL1,CCND2,BASP1,RBP1,CYP1B1,LPL,PMEPA1,ATP1B1,TMSB10,SOX4
935	BBX	821	ARCHS4 Coexpression,551;Enrichr Queries,418;GTEx Coexpression,1494	HSPA8,CLIC4,CDKN1A,ANXA1,TSC22D1,ANXA2,ARPC1B,TALDO1,IQGAP1,RTN4,TMEM123,KLF6,CCND2,CAPZB,CFL1,MYH9,SOX4,SPTBN1
936	HOXA13	821.3	ARCHS4 Coexpression,1075;Enrichr Queries,823;GTEx Coexpression,566	COL1A1,SLC12A2,CDKN1A,CCND2,CLDN7,CYP1B1,PMEPA1,TIMP3,JUNB,PDLIM7,SOX4
937	ZNF586	821.3	ARCHS4 Coexpression,116;Enrichr Queries,1107;GTEx Coexpression,1241	CDKN1A,RPS5,ACTN1,MYOF,ADAM10,IQGAP1,RTN4,FAM129B,PKM,CAPNS1,MAP4,RAC1,YWHAG,IER3
938	MXD1	821.3	ARCHS4 Coexpression,1209;Enrichr Queries,328;GTEx Coexpression,927	CSTB,CD74,CDKN1A,ANXA1,ANXA2,SERPINE1,LGALS3,KLF6,CCND2,BASP1,FTH1,S100A6,LCN2,CYP1B1,SH3BGRL3,NUPR1,JUNB,ITM2B,IER3
939	DR1	822.3	ARCHS4 Coexpression,1626;Enrichr Queries,206;GTEx Coexpression,635	HSPA8,CLIC4,CDKN1A,TPM4,ANXA2,CTR9,ADAM10,IQGAP1,ACTB,RTN4,CDC42,RAB10,TMEM123,KLF6,PKM,ARPC2,EIF6,FTH1,MYH9,CTNNB1,TAGLN2,JUNB,YWHAG
940	HOXC12	822.7	ARCHS4 Coexpression,803;Enrichr Queries,1333;GTEx Coexpression,332	CERS3,COL1A1,MMP14,JUP,CAPNS1,KRT14,MYOF
941	ZNF772	823.7	ARCHS4 Coexpression,120;Enrichr Queries,1112;GTEx Coexpression,1239	EIF5A,CDKN1A,GSN,TSC22D1,ARPC1B,CCND2,CAPNS1,CAPZB,GSTA4,BSG,MYH9,TAGLN2,SOX4,S100A11
942	ZFP69	824	ARCHS4 Coexpression,1301;GTEx Coexpression,347	LDHB,TMSB4X,ADAM10,ACTB
943	POU6F2	824.3	ARCHS4 Coexpression,378;Enrichr Queries,1372;GTEx Coexpression,723	S100G,PKM,CAPNS1,MYH9,FLNA,IQGAP1,YWHAG,FAM129B
944	OLIG3	824.7	ARCHS4 Coexpression,1090;Enrichr Queries,1275;GTEx Coexpression,109	EEF1B2,CERS3,CDKN1A,RPL18A,EIF6,SRD5A1,BASP1,RPS5,RBP1,SDC1,SOX4
945	ZGPAT	825	ARCHS4 Coexpression,819;Enrichr Queries,814;GTEx Coexpression,842	CSTB,CDKN1A,ARPC1B,TMEM176A,IQGAP1,EIF6,FTH1,CFL1,TAGLN2,SOX4,S100A11,PDLIM7,S100A10
946	ZNF584	827.8	ARCHS4 Coexpression,1317;Enrichr Queries,715;ReMap ChIP-seq,226;GTEx Coexpression,1053	CDKN1A,ANXA1,TSC22D1,ANXA2,SERPINE1,DSTN,CAPG,PDZK1IP1,ACTB,RTN4,RAB11A,CDC42,KLF6,PKM,FTH1,TMSB4X,S100A6,MYH9,SH3BGRL3,JUNB,SOX4,IER3
947	ZBED1	828	ARCHS4 Coexpression,1585;Enrichr Queries,98;ReMap ChIP-seq,60;GTEx Coexpression,1569	CSTB,CDKN1A,RPLP0,SERPINE1,ACTB,MYL12A,PRDX2,LGALS3,BASP1,CTSL,TMSB4X,FTH1,CFL1,BSG,RACK1,FLNA,CYP1B1,TIMP3,TMSB10,JUNB,CLIC1,PDLIM7,SOX4,IER3,S100A10,HSPA8,CD74,RPS9,ANXA1,TNFRSF12A,JUP,ANXA2,RPS5,ACTN1,MYOF,RPSA,ATP1B1,EEF1G,KLF6,PKM,S100A6,MYH9,TAGLN2,ITM2B
948	ADNP2	828.7	ARCHS4 Coexpression,1307;Enrichr Queries,371;GTEx Coexpression,808	CERS3,HSPA8,CLIC4,CDKN1A,TSC22D1,ACTN1,RPLP0,IQGAP1,ACTB,RTN4,KLF6,CCND2,MYH9,ITGA6,JUNB,SOX4,SPTBN1,IER3
949	ZSCAN29	829	ARCHS4 Coexpression,240;Enrichr Queries,1040;GTEx Coexpression,1207	HSPA8,CDKN1A,ARF1,EIF6,GSTO1,CFL1,ITGA6,JUNB,SOX4,MYL12A,S100A11,S100A10
950	ZNF787	830.7	ARCHS4 Coexpression,698;Enrichr Queries,679;GTEx Coexpression,1115	EIF5A,CSTB,RPS9,CDKN1A,RPL18A,TNFRSF12A,CAPNS1,RPS5,MYH9,JUNB,PDLIM7,FAM129B
951	YBX2	831.7	ARCHS4 Coexpression,978;Enrichr Queries,174;GTEx Coexpression,1343	CSTB,CDKN1A,ANXA1,GSN,GPX3,KRT8,KRT7,LPL,ATP1B1,CLU,WFDC2,COL1A1,KRT19,KRT18,ELF3,CLDN7,S100A6,LCN2,CYP1B1,APOE,JUNB,IER3
952	ZNF433	832	ARCHS4 Coexpression,217;Enrichr Queries,748;GTEx Coexpression,1531	EIF5A,CDKN1A,JUP,GPX3,ATP1B1,KLF6,PKM,CAPNS1,GSTA4,CYP1B1,PMEPA1,MYH9,FLNA,JUNB,SOX4,IER3
953	ZFP42	833	Literature ChIP-seq,127;ARCHS4 Coexpression,1018;Enrichr Queries,702;GTEx Coexpression,1485	YWHAE,S100G,RPS9,IDH1,KRT8,LRP2,ATP1B1,WFDC2,EEF1B2,LDHB,LARS2,CLDN4,KRT18,PKM,ELF3,GSTA4,BASP1,CLDN7,UCP2,LCN2,CYP1B1,APOE,ITM2B
954	NKX26	833	ARCHS4 Coexpression,229;GTEx Coexpression,1437	COL1A1,CLIC4,MMP14,TNFRSF12A,ACTN1,PDLIM7,FAM129B
955	NR1I3	833.7	ARCHS4 Coexpression,1105;Enrichr Queries,782;GTEx Coexpression,614	CSTB,CDKN1A,TMEM176B,TMEM176A,LPL,CP,FMO5,KRT18,PKM,LCN2,MYH9,APOE,JUNB
956	BARX1	833.7	ARCHS4 Coexpression,963;Enrichr Queries,1189;GTEx Coexpression,349	COL1A1,BASP1,ACTN1,RDH10,BSG,RPLP0,CYP1B1,PDLIM7
957	ZNF610	833.7	ARCHS4 Coexpression,257;Enrichr Queries,1121;GTEx Coexpression,1123	CDKN1A,CCND2,PKM,CAPNS1,GSTA4,SERPINE1,CYP1B1,MYH9,FLNA,IQGAP1,SOX4
958	HOXC8	834.3	ARCHS4 Coexpression,1455;Enrichr Queries,356;GTEx Coexpression,692	CSTB,CDKN1A,ANXA1,GSN,SERPINE1,COL1A1,KLF6,KRT19,CCND2,KRT18,MGP,CYP1B1,PMEPA1,TIMP3,NUPR1,SOX4
959	ZNF665	834.7	ARCHS4 Coexpression,1560;Enrichr Queries,243;GTEx Coexpression,701	CSTB,CLIC4,CDKN1A,ANXA1,ANXA3,GPX3,ATP1B1,ACTB,PTGS1,COL1A1,LGALS3,KLF6,CCND2,BASP1,CYP1B1,TIMP3,JUNB,SOX4,IER3
960	ZNF451	835.3	ARCHS4 Coexpression,298;Enrichr Queries,849;GTEx Coexpression,1359	EIF5A,CLIC4,CDKN1A,TSC22D1,IQGAP1,KLF6,CAPNS1,CAPZB,CFL1,BSG,CTNNB1,RAC1,SOX4,SPTBN1
961	ZNF391	835.7	ARCHS4 Coexpression,340;Enrichr Queries,1149;GTEx Coexpression,1018	CCND2,GSTA4,CAPNS1,CAPZB,ARPC1B,CYP1B1,MYH9,TAGLN2,SOX4,FAM129B
962	TBX20	835.7	ARCHS4 Coexpression,724;Enrichr Queries,817;GTEx Coexpression,966	COL1A1,CDKN1A,KLF6,CLIC4,MGST3,SERPINE1,CYP1B1,LPL,TIMP3,MAP4,SOX4,SPTBN1
963	ZNF483	836.3	ARCHS4 Coexpression,496;Enrichr Queries,1101;GTEx Coexpression,912	CDKN1A,UCHL1,CCND2,LAS1L,CAPNS1,GPX3,FUT9,CFL1,MYH9,CALM1,CLU
964	ZNF514	836.3	ARCHS4 Coexpression,102;Enrichr Queries,1058;GTEx Coexpression,1349	EIF5A,CDKN1A,ARF1,GSTO1,ATP1B1,ACTB,KLF6,PKM,CAPZB,MGAT4A,CFL1,BSG,TAGLN2,S100A11,IER3
965	ZNF16	836.7	ARCHS4 Coexpression,1130;Enrichr Queries,751;GTEx Coexpression,629	EEF1G,COL1A1,CDKN1A,KLF6,PKM,JUP,TSC22D1,CAPNS1,CYP1B1,PMEPA1,MYH9,RPS13
966	GPBP1L1	837.5	ARCHS4 Coexpression,703;GTEx Coexpression,972	EEF1G,TMEM123,UCHL1,IQGAP1,YWHAG
967	ZNF3	838.3	ARCHS4 Coexpression,1123;Enrichr Queries,846;GTEx Coexpression,546	HSPA8,CDKN1A,KLF6,CCND2,TSC22D1,RACK1,RPL14,JUNB,ACTB,SOX4,RPS13
968	ZNF385B	838.7	ARCHS4 Coexpression,1124;Enrichr Queries,1165;GTEx Coexpression,227	FUT9,SERPINE1,LPL,IQGAP1,CALM1,MICU1,ATP1B1,CLU,YWHAG
969	ZNF416	838.7	ARCHS4 Coexpression,1545;Enrichr Queries,428;GTEx Coexpression,543	CERS3,CSTB,CDKN1A,JUP,TNFRSF12A,ANXA3,GPX3,SERPINE1,COL1A1,KLF6,CCND2,PMEPA1,JUNB,SOX4,IER3
970	ZNF253	839.3	ARCHS4 Coexpression,63;Enrichr Queries,1168;GTEx Coexpression,1287	ACTN1,MYOF,FAM129B,TMEM123,PKM,GSTA4,CAPNS1,MGAT4A,BSG,MYH9,FLNA,TAGLN2,SOX4
971	BHLHA15	839.5	ARCHS4 Coexpression,1387;GTEx Coexpression,292	EEF1B2,EGF,MGAT4A,RBP1
972	SNAI3	839.7	ARCHS4 Coexpression,1262;Enrichr Queries,173;GTEx Coexpression,1084	CSTB,CD74,CDKN1A,ANXA1,GSN,TNFRSF12A,GPX3,SERPINE1,LPL,CAPG,ATP1B1,CLU,COL1A1,LGALS3,CCND2,UCP2,S100A6,CYP1B1,APOE,JUNB,PDLIM7,ITM2B,IER3
973	ZBTB47	839.7	ARCHS4 Coexpression,1170;Enrichr Queries,604;GTEx Coexpression,745	CDKN1A,GSN,COL1A1,KLF6,CCND2,CYP1B1,PMEPA1,TIMP3,MAP4,JUNB,SPTBN1,PDLIM7
974	RORC	841	ARCHS4 Coexpression,1382;Enrichr Queries,155;GTEx Coexpression,986	CSTB,CDKN1A,SERPINE1,LPL,CLU,LGALS3,TIMP3,JUNB,SOX4,IER3,S100A10,CD74,ANXA1,GSN,EGF,GPX3,KRT8,ATP1B1,FMO5,COL1A1,KRT18,ELF3,LCN2,NUPR1
975	ZNF540	841	ARCHS4 Coexpression,403;Enrichr Queries,1249;GTEx Coexpression,871	PKM,CAPNS1,GSTA4,GPX3,FUT9,MGAT4A,CYP1B1,MYH9,CALM1
976	ST18	841.3	ARCHS4 Coexpression,397;Enrichr Queries,1090;GTEx Coexpression,1037	EIF5A,CDKN1A,CCND2,GSN,GSTA4,BASP1,TMEM176B,RBP1,LPL,MYH9,SOX4
977	ZNF444	842	ARCHS4 Coexpression,1598;Enrichr Queries,603;GTEx Coexpression,325	CSTB,CDKN1A,JUP,TNFRSF12A,IQGAP1,ACTB,COL1A1,KLF6,ARPC2,BSG,S100A6,MYH9,JUNB,PDLIM7,S100A10
978	ZNF12	842.3	ARCHS4 Coexpression,462;Enrichr Queries,1285;GTEx Coexpression,780	YWHAE,KLF6,ARF1,CAPZB,ARPC1B,CFL1,SH3BGRL3,SOX4
979	ZSCAN31	843	ARCHS4 Coexpression,1232;GTEx Coexpression,454	ARPC1B,CFL1,SH3BGRL3,CLIC1
980	ZNF280A	843.7	ARCHS4 Coexpression,1500;Enrichr Queries,667;GTEx Coexpression,364	CSTB,CDKN1A,JUP,IDH1,SERPINE1,COL1A1,CCND2,GSTA4,TIMP3,APOE,SOX4,IER3
981	ZNF668	843.7	ARCHS4 Coexpression,843;Enrichr Queries,944;GTEx Coexpression,744	EIF5A,CSTB,CDKN1A,TNFRSF12A,CFL1,SH3BGRL3,JUNB,PDLIM7,IER3
982	FOXG1	843.7	ARCHS4 Coexpression,585;Enrichr Queries,747;GTEx Coexpression,1199	UCHL1,CCND2,GSTA4,BASP1,FUT9,CYP1B1,LPL,TIMP3,ATP1B1,CLU,SOX4
983	ZNF43	844.3	ARCHS4 Coexpression,559;Enrichr Queries,583;GTEx Coexpression,1391	SLC12A2,CDKN1A,ANXA1,LPL,ATP1B1,CLU,FAM129B,CCND2,PKM,CAPNS1,CYP1B1,TIMP3,MYH9,ITGA6,SOX4
984	MYNN	845.3	ARCHS4 Coexpression,744;Enrichr Queries,1256;ReMap ChIP-seq,145;GTEx Coexpression,1236	CSTB,VCP,ARPC1B,RPLP0,ACTB,FAM129B,PRDX2,CAPNS1,CFL1,BSG,RACK1,FLNA,PMEPA1,SH3BGRL3,JUNB,CLIC1,PDLIM7,RPS9,TNFRSF12A,TALDO1,ATP1B1,EEF1G,KLF6,MYH9,TAGLN2
985	LHX1	845.5	ARCHS4 Coexpression,641;Enrichr Queries,1050	COL1A1,YWHAE,CDKN1A,UCHL1,CCND2,BASP1,CYP1B1,TMSB10,SOX4
986	FOXL2	845.7	ARCHS4 Coexpression,1277;Enrichr Queries,892;GTEx Coexpression,368	COL1A1,CSTB,CDKN1A,CCND2,RBP1,RACK1,CYP1B1,CLU,SOX4
987	ZNF703	845.7	ARCHS4 Coexpression,1261;Enrichr Queries,91;GTEx Coexpression,1185	CSTB,CDKN1A,SERPINE1,CLU,LGALS3,CCND2,CAPNS1,BASP1,CYP1B1,PMEPA1,TIMP3,FLNA,APOE,JUNB,SOX4,IER3,S100A10,ANXA1,GSN,JUP,ANXA2,MYOF,KRT8,ATP1B1,COL1A1,KLF6,KRT19,KRT18
988	ZBTB6	845.7	ARCHS4 Coexpression,650;Enrichr Queries,1075;GTEx Coexpression,812	RAB10,TMEM123,HSPA8,CDKN1A,KLF6,CTR9,ADAM10,IQGAP1,JUNB,SOX4
989	EBF4	846.7	ARCHS4 Coexpression,1153;Enrichr Queries,606;GTEx Coexpression,781	CDKN1A,GSN,CADM4,SERPINE1,CLU,COL1A1,CCND2,CYP1B1,PMEPA1,TIMP3,APOE,PDLIM7,SOX4
990	PPARA	847	ARCHS4 Coexpression,1544;Enrichr Queries,427;GTEx Coexpression,570	CDKN1A,GSN,TMEM176B,TMEM176A,SERPINE1,LPL,ATP1B1,CLU,COL1A1,LGALS3,KLF6,CCND2,TIMP3,ITGA6,APOE,JUNB
991	LCORL	847	ARCHS4 Coexpression,215;Enrichr Queries,1261;GTEx Coexpression,1065	EIF5A,CDKN1A,KLF6,CAPNS1,CAPZB,GSTA4,MYH9,SOX4,S100A11
992	MBD1	847.3	ARCHS4 Coexpression,1195;Enrichr Queries,327;GTEx Coexpression,1020	HSPA8,CDKN1A,GSN,TSC22D1,ANXA2,ACTN1,SERPINE1,ACTB,RTN4,KLF6,CCND2,PKM,MYH9,FLNA,CTNNB1,JUNB,PDLIM7
993	ZNF596	847.3	ARCHS4 Coexpression,670;Enrichr Queries,952;GTEx Coexpression,920	CDKN1A,ANXA1,GSN,PKM,LAS1L,GSTA4,CAPNS1,CYP1B1,PMEPA1,MYH9,FLNA,CLU
994	MYF5	847.7	ARCHS4 Coexpression,379;Enrichr Queries,746;GTEx Coexpression,1418	CSTB,CDKN1A,CLIC4,ANXA2,SERPINE1,LPL,MYL12A,COL1A1,LGALS3,S100A6,CYP1B1,JUNB,S100A11,IER3
995	ZNF222	848.3	ARCHS4 Coexpression,563;Enrichr Queries,770;GTEx Coexpression,1212	CDKN1A,KLF6,ANXA1,CAPNS1,GSTA4,CYP1B1,MYH9,ATP1B1,CLU,JUNB,SOX4,IER3
996	ZNF432	848.3	ARCHS4 Coexpression,630;Enrichr Queries,845;GTEx Coexpression,1070	EIF5A,CDKN1A,KLF6,ANXA1,PKM,CAPNS1,SERPINE1,BSG,PMEPA1,JUNB,SOX4,IER3
997	LHX8	848.7	ARCHS4 Coexpression,967;Enrichr Queries,1210;GTEx Coexpression,369	YWHAE,COL1A1,CCND2,GSTA4,KRT7,CYP1B1,WFDC2,SOX4
998	ZFY	849	ARCHS4 Coexpression,522;Enrichr Queries,576;GTEx Coexpression,1449	EIF5A,CDKN1A,ANXA1,ANXA3,SERPINE1,CLU,CCND2,PKM,CFL1,BSG,CYP1B1,PMEPA1,ITGA6,RAC1,JUNB,IER3
999	PKNOX1	849.3	ARCHS4 Coexpression,1493;Enrichr Queries,486;ReMap ChIP-seq,158;GTEx Coexpression,1260	CLIC4,CDKN1A,CAPG,ACTB,RTN4,CDC42,PRDX2,CCND2,FTH1,TMSB4X,PMEPA1,SH3BGRL3,JUNB,CLIC1,YWHAG,PDLIM7,SOX4,IER3,HSPA8,TNFRSF12A,TSC22D1,ACTN1,KLF6,KRT19,EIF6,KRT15,MYH9,ITGA6
1000	ZNF845	849.3	ARCHS4 Coexpression,719;Enrichr Queries,1347;GTEx Coexpression,482	CDC42,CAPNS1,RPLP0,PMEPA1,IQGAP1,MAP4,PDLIM7
1001	ZNF431	849.7	ARCHS4 Coexpression,1205;Enrichr Queries,1314;GTEx Coexpression,30	CSTB,ANXA1,PRSS27,PRSS22,RAB11A,FAM129B,RAB10,PKM,MGAT4A,LY6D,SFN,TRIM16,S100A14,SPRR1A,IVL,SOX4,S100A10
1002	ZNF417	849.7	ARCHS4 Coexpression,267;Enrichr Queries,1239;GTEx Coexpression,1043	EIF5A,CDKN1A,PKM,CAPNS1,ACTN1,CFL1,BSG,RAC1,SOX4,IER3
1003	ZNF549	849.7	ARCHS4 Coexpression,920;Enrichr Queries,509;GTEx Coexpression,1120	EIF5A,HSPA8,CSTB,RPS9,CDKN1A,ANXA1,ACTB,MYL12A,KLF6,CCND2,PKM,CAPNS1,BASP1,CYP1B1,IER3
1004	DMRTA1	850	ARCHS4 Coexpression,985;Enrichr Queries,776;GTEx Coexpression,789	COL1A1,PRDX2,CDKN1A,ANXA1,TMEM176A,SERPINE1,RACK1,CYP1B1,LPL,PMEPA1,TIMP3,SOX4
1005	NFXL1	850.3	ARCHS4 Coexpression,777;Enrichr Queries,961;GTEx Coexpression,813	CLIC4,CDKN1A,KLF6,ANXA1,CCND2,CAPNS1,CTR9,ITGA6,S100A14,RTN4,IER3
1006	CXXC1	850.7	ARCHS4 Coexpression,1576;Enrichr Queries,297;GTEx Coexpression,679	HSPA8,CSTB,RPS9,CDKN1A,ANXA2,RPS5,ACTN1,RPLP0,ACTB,KLF6,PKM,FTH1,CFL1,MYH9,FLNA,SH3BGRL3,JUNB,PDLIM7
1007	ZNF202	851	ARCHS4 Coexpression,1047;Enrichr Queries,742;GTEx Coexpression,764	HSPA8,CDKN1A,KLF6,CCND2,RPS5,SERPINE1,PMEPA1,RPSA,JUNB,SOX4,IER3
1008	FOXD4L5	851	ARCHS4 Coexpression,1276;Enrichr Queries,1124;GTEx Coexpression,153	CDKN1A,CCND2,ANXA1,GPX3,BASP1,SRD5A1,RDH10,LPL,RTN4,SPTBN1
1009	ZNF749	852.3	ARCHS4 Coexpression,440;Enrichr Queries,1363;GTEx Coexpression,754	EIF5A,LDHB,CAPNS1,EIF6,RPS5,SOX4,ITM2B
1010	ZNF10	853.3	ARCHS4 Coexpression,8;Enrichr Queries,994;GTEx Coexpression,1558	EIF5A,CDKN1A,TPM4,ACTN1,ARPC1B,ATP1B1,FAM129B,KLF6,PKM,CAPNS1,CAPZB,GSTA4,CFL1,BSG,PMEPA1,MYH9,FLNA,TAGLN2,JUNB,CLIC1,SOX4,S100A11
1011	FIZ1	854.3	ARCHS4 Coexpression,1240;Enrichr Queries,639;GTEx Coexpression,684	EIF5A,HSPA8,CDKN1A,ACTB,KLF6,PKM,CAPNS1,NUPR1,APOE,JUNB,SOX4,PDLIM7
1012	ZBTB24	854.7	ARCHS4 Coexpression,414;Enrichr Queries,762;GTEx Coexpression,1388	CDKN1A,CADM4,TSC22D1,ATP1B1,FAM129B,KLF6,CCND2,MGAT4A,BSG,PMEPA1,MAP4,JUNB,PDLIM7,IER3
1013	ZNF354C	855	ARCHS4 Coexpression,181;Enrichr Queries,938;GTEx Coexpression,1446	CLIC4,ANXA1,ARPC1B,COL1A1,UCHL1,CCND2,EIF6,GSTA4,FUT9,CYP1B1,PMEPA1,ITGA6,CLIC1,SOX4,S100A11
1014	ZNF579	855.3	ARCHS4 Coexpression,716;Enrichr Queries,511;GTEx Coexpression,1339	CSTB,CDKN1A,RPS9,TNFRSF12A,ACTB,UCHL1,BASP1,FTH1,S100A6,RACK1,NUPR1,APOE,JUNB,SOX4,PDLIM7,IER3
1015	AKAP8	855.7	ARCHS4 Coexpression,1093;Enrichr Queries,573;GTEx Coexpression,901	YWHAE,HSPA8,CDKN1A,ANXA2,ACTN1,RPS5,IQGAP1,ACTB,KLF6,EIF6,MYH9,FLNA,APOE,JUNB
1016	NR2E1	857.3	ARCHS4 Coexpression,727;Enrichr Queries,1130;GTEx Coexpression,715	YWHAE,CDKN1A,CCND2,GSTA4,FUT9,CYP1B1,LRP2,CLU
1017	FOXD2	857.7	ARCHS4 Coexpression,1460;Enrichr Queries,620;GTEx Coexpression,493	CSTB,CLIC4,CDKN1A,COL1A1,KLF6,CCND2,ELF3,S100A6,CYP1B1,PMEPA1,ITGA6,JUNB,SOX4,IER3
1018	GCM1	858	ARCHS4 Coexpression,1281;Enrichr Queries,234;GTEx Coexpression,1059	S100G,CDKN1A,ANXA1,ANXA2,SERPINE1,KRT8,KRT7,COL1A1,LGALS3,CLDN4,KLF6,CCND2,KRT18,ELF3,BASP1,CTSL,CYP1B1,TIMP3,JUNB,SOX4
1019	ZNF302	859	ARCHS4 Coexpression,1347;Enrichr Queries,1148;GTEx Coexpression,82	ANXA1,TSC22D1,CAPG,IQGAP1,CDC42,UCHL1,EIF6,ARPC2,TAGLN2,SH3BGRL3,ITGA6,CLIC1,SOX4
1020	ZNF90	859.7	ARCHS4 Coexpression,1035;Enrichr Queries,1283;GTEx Coexpression,261	ANXA1,CAPNS1,RPLP0,RPL11,RACK1,MYH9,RPSA
1021	ZNF662	859.7	ARCHS4 Coexpression,1156;Enrichr Queries,577;GTEx Coexpression,846	CSTB,ANXA1,GPX3,SERPINE1,RPL7,COL1A1,CCND2,PKM,MGP,CYP1B1,PMEPA1,TIMP3,ITGA6
1022	ZNF808	861	ARCHS4 Coexpression,477;Enrichr Queries,1289;GTEx Coexpression,817	YWHAE,EIF5A,CDKN1A,PKM,CAPNS1,MGAT4A,CFL1,BSG
1023	ZSCAN12	862.3	ARCHS4 Coexpression,172;Enrichr Queries,874;GTEx Coexpression,1541	CD74,CDKN1A,TSC22D1,ACTN1,FAM129B,KLF6,CCND2,CAPNS1,CAPZB,BSG,MYH9,JUNB,SOX4,S100A11,IER3
1024	HINFP	862.3	ARCHS4 Coexpression,1575;ReMap ChIP-seq,225;GTEx Coexpression,787	JUP,SLC34A1,SERPINE1,CTR9,RPLP0,RTN4,KLF6,KRT19,LAS1L,CTSL,FTH1,FXYD2,TMSB4X,PDLIM7,IER3
1025	ZNF784	863.3	ARCHS4 Coexpression,782;Enrichr Queries,721;GTEx Coexpression,1087	CSTB,CDKN1A,KLF6,RPS9,TNFRSF12A,CAPNS1,PMEPA1,JUNB,SOX4,PDLIM7,IER3
1026	DBP	863.3	ARCHS4 Coexpression,1368;Enrichr Queries,67;GTEx Coexpression,1155	CSTB,CDKN1A,SERPINE1,LPL,CLU,ACTB,LGALS3,CCND2,FTH1,UCP2,PMEPA1,TIMP3,APOE,JUNB,SOX4,IER3,S100A10,HSPA8,CD74,GSN,TSC22D1,TPM4,GPX3,ATP1B1,COL1A1,KLF6,MGP,S100A6,LCN2,NUPR1
1027	POU6F1	864	ARCHS4 Coexpression,1162;Enrichr Queries,555;GTEx Coexpression,875	CDKN1A,GSN,TSC22D1,LPL,CLU,COL1A1,KLF6,CCND2,PMEPA1,TIMP3,CALM1,JUNB,SOX4
1028	ZNF138	864.3	ARCHS4 Coexpression,64;Enrichr Queries,1385;GTEx Coexpression,1144	LDHB,CAPNS1,CAPZB,GSTA4,ACTN1,IDH1,MYH9,FLNA,TAGLN2,SOX4,FAM129B
1029	BORCS8MEF2B	867	ARCHS4 Coexpression,867	CAPZB,TALDO1,TAGLN2
1030	ZNF777	867.7	ARCHS4 Coexpression,629;Enrichr Queries,882;GTEx Coexpression,1092	COL1A1,EIF5A,CSTB,CDKN1A,KLF6,PKM,JUP,CAPNS1,MYH9,JUNB,IER3
1031	HOXD10	868.7	ARCHS4 Coexpression,1591;Enrichr Queries,465;GTEx Coexpression,550	CSTB,CDKN1A,ANXA1,GPX3,SERPINE1,LPL,CLU,COL1A1,TMEM123,LGALS3,CCND2,MGP,CYP1B1,TIMP3,PDLIM7
1032	DBX2	869.7	ARCHS4 Coexpression,723;Enrichr Queries,1156;GTEx Coexpression,730	YWHAE,IL33,TSC22D1,FUT9,LPL,TIMP3,APOE,CLU
1033	OTX2	872.3	ARCHS4 Coexpression,1246;Enrichr Queries,723;ReMap ChIP-seq,249;GTEx Coexpression,1271	IL33,CDKN1A,ANXA1,ANXA2,ANXA3,IDH1,MYOF,KRT8,KRT7,LRP2,RTN4,WFDC2,COL1A1,KRT18,MGP,S100A6,CYP1B1,APOE,SOX4,S100A10
1034	ZNF789	872.7	ARCHS4 Coexpression,794;Enrichr Queries,1081;GTEx Coexpression,743	CDKN1A,KLF6,PKM,LAS1L,CAPNS1,PMEPA1,JUNB,IER3,FAM129B
1035	ZNF2	873	ARCHS4 Coexpression,1038;Enrichr Queries,708	CDKN1A,KLF6,CCND2,TSC22D1,CAPNS1,PMEPA1,MYH9,ITGA6,ATP1B1,JUNB,SOX4,IER3
1036	ZFP82	874.3	ARCHS4 Coexpression,437;Enrichr Queries,913;GTEx Coexpression,1273	CDKN1A,PKM,CAPNS1,SERPINE1,CYP1B1,PMEPA1,TIMP3,MYH9,FLNA,SOX4,IER3,FAM129B
1037	ZNF227	875	ARCHS4 Coexpression,220;Enrichr Queries,977;GTEx Coexpression,1428	CDKN1A,ANXA1,ACTN1,FAM129B,LDHB,KLF6,CAPNS1,CAPZB,PMEPA1,MYH9,JUNB,SOX4,S100A11
1038	EMX1	875	ARCHS4 Coexpression,1214;Enrichr Queries,1057;GTEx Coexpression,354	MIOX,S100G,CDKN1A,UCHL1,CCND2,SLC34A1,FXYD2,CYP1B1,LRP2
1039	ZNF345	875.3	ARCHS4 Coexpression,983;Enrichr Queries,1232;GTEx Coexpression,411	YWHAE,LARS2,CDKN1A,PKM,CAPNS1,TIMP3,YWHAG,SOX4
1040	CXXC5	875.5	ARCHS4 Coexpression,555;GTEx Coexpression,1196	CADM4,MAP4,CLU,YWHAG
1041	DUXA	876	ARCHS4 Coexpression,1238;Enrichr Queries,1300;GTEx Coexpression,90	COL1A1,CLIC4,CCND2,MGP,DSTN,PMEPA1,MYH9,FLNA,ACTB,RPS13
1042	ZNF678	876.7	ARCHS4 Coexpression,307;Enrichr Queries,1250;GTEx Coexpression,1073	CLIC4,KLF6,CAPNS1,CAPZB,ARPC1B,CFL1,SPTBN1,SOX4,S100A11
1043	RFX4	877	ARCHS4 Coexpression,357;Enrichr Queries,1144;GTEx Coexpression,1130	S100G,CDKN1A,CCND2,FUT9,TIMP3,MYH9,IQGAP1,CLU,S100A11
1044	FOXO4	877	ARCHS4 Coexpression,1561;Enrichr Queries,359;GTEx Coexpression,711	CDKN1A,GSN,JUP,TSC22D1,SERPINE1,ATP1B1,CLU,COL1A1,KLF6,CCND2,TIMP3,MAP4,NUPR1,APOE,JUNB,SOX4,IER3
1045	ZXDA	877.7	ARCHS4 Coexpression,1037;Enrichr Queries,541;GTEx Coexpression,1055	CDKN1A,ANXA1,ANXA3,IQGAP1,KLF6,CCND2,BASP1,CYP1B1,PMEPA1,TIMP3,JUNB,SOX4,IER3
1046	ZNF440	878	ARCHS4 Coexpression,1546;Enrichr Queries,999;GTEx Coexpression,89	CERS3,CDKN1A,JUP,SERPINE1,KRT5,RAB11A,CLDN4,KLF6,EIF6,MGAT4A,LY6D,SDC1,JUNB,IER3
1047	ZNF620	878.3	ARCHS4 Coexpression,1016;Enrichr Queries,1182;GTEx Coexpression,437	CERS3,CDKN1A,CCND2,JUP,CAPNS1,MGAT4A,MYH9,RAB11A,SOX4
1048	ZNF441	878.3	ARCHS4 Coexpression,387;Enrichr Queries,1015;GTEx Coexpression,1233	EIF5A,CDKN1A,RPS9,ANXA1,GSTA4,CAPNS1,SRD5A1,MGAT4A,CFL1,CYP1B1,JUNB
1049	POU4F2	879	ARCHS4 Coexpression,822;Enrichr Queries,1196;ReMap ChIP-seq,253;GTEx Coexpression,1245	CSTB,VCP,CLIC4,RPS5,DSTN,LRP2,EEF1G,COL1A1,UCHL1,GSTA4,FUT9,BASP1,CLDN7,CYP1B1,CALM1,TMSB10,SOX4
1050	REPIN1	880	ARCHS4 Coexpression,1203;Enrichr Queries,125;GTEx Coexpression,1312	CSTB,CDKN1A,CLU,ACTB,RTN4,LDHB,CCND2,CAPNS1,FTH1,JUNB,SOX4,IER3,S100A10,HSPA8,GSN,JUP,TSC22D1,ANXA2,ACTN1,ATP1B1,KLF6,PKM,KRT18,MYH9,TAGLN2
1051	ZNF625	880.7	ARCHS4 Coexpression,239;Enrichr Queries,1078;GTEx Coexpression,1325	CSTB,CCND2,PKM,TSC22D1,CAPNS1,MYH9,FLNA,TAGLN2,IQGAP1,JUNB,SOX4
1052	NHLH2	880.7	ARCHS4 Coexpression,283;Enrichr Queries,1287;GTEx Coexpression,1072	CDKN1A,UCHL1,GSTA4,BASP1,RBP1,LPL,TMSB10,SOX4,RTN4
1053	KDM2B	881	ARCHS4 Coexpression,1315;GTEx Coexpression,447	CERS3,JUP,SDC1,DSTN
1054	RLF	881.3	ARCHS4 Coexpression,722;Enrichr Queries,969;GTEx Coexpression,953	KLF6,CLIC4,CDKN1A,EIF6,CAPNS1,ARPC1B,CFL1,MYH9,ATP1B1,JUNB

1055	TFAP2E	882.3	ARCHS4 Coexpression,748;Enrichr Queries,1026;GTEx Coexpression,873	CSTB,CD74,CDKN1A,GSN,LAS1L,ELF3,TMSB4X,UCP2,CYP1B1,JUNB
1056	ZNF696	884	ARCHS4 Coexpression,894;Enrichr Queries,452;GTEx Coexpression,1306	CDKN1A,TPM4,TSC22D1,IQGAP1,ACTB,MYL12A,FAM129B,KLF6,CCND2,FTH1,PMEPA1,MYH9,FLNA,JUNB,SOX4,ITM2B,IER3
1057	ZNF606	884.3	ARCHS4 Coexpression,45;Enrichr Queries,1181;GTEx Coexpression,1427	EIF5A,CDKN1A,ANXA1,ACTN1,IQGAP1,FAM129B,PKM,CAPNS1,CAPZB,GSTA4,PMEPA1,MYH9,FLNA,TAGLN2,SOX4
1058	ZNF684	885	ARCHS4 Coexpression,981;Enrichr Queries,1091;GTEx Coexpression,583	CLIC4,CDKN1A,KLF6,PKM,TSC22D1,TMEM176B,TMEM176A,CYP1B1,MYH9
1059	PHF20	887.7	ARCHS4 Coexpression,562;Enrichr Queries,643;GTEx Coexpression,1458	HSPA8,CDKN1A,IQGAP1,ATP1B1,RTN4,FAM129B,KLF6,PKM,EIF6,CAPNS1,CFL1,BSG,MYH9,CALM1,SPTBN1
1060	ZBTB3	887.7	ARCHS4 Coexpression,1333;Enrichr Queries,468;GTEx Coexpression,862	CDKN1A,JUP,TNFRSF12A,TSC22D1,TALDO1,COL1A1,KLF6,CCND2,CYP1B1,PMEPA1,MYH9,JUNB,SOX4,IER3
1061	ZNF592	888.3	ARCHS4 Coexpression,1292;Enrichr Queries,632;ReMap ChIP-seq,224;GTEx Coexpression,1405	HSPA8,SLC6A19,CDKN1A,ANXA2,ACTB,KRT19,KLF6,PKM,ELF3,KRT15,CFL1,LY6D,MYH9,FLNA,MAP4,JUNB,SPTBN1
1062	ZNF239	888.3	ARCHS4 Coexpression,676;Enrichr Queries,440;GTEx Coexpression,1549	HSPA8,CDKN1A,ANXA1,TNFRSF12A,ANXA3,IDH1,SERPINE1,RPLP0,LPL,ATP1B1,LDHB,UCHL1,KLF6,CYP1B1,PMEPA1,JUNB,SOX4,IER3
1063	ZNF101	889.7	ARCHS4 Coexpression,759;Enrichr Queries,1047;GTEx Coexpression,863	KLF6,PKM,ARPC1B,MGAT4A,MYH9,MAP4,JUNB,YWHAG,IER3
1064	SNAPC4	890.8	ARCHS4 Coexpression,1134;Enrichr Queries,1070;ReMap ChIP-seq,259;GTEx Coexpression,1100	SLC12A2,EIF5A,SPRR2F,CDKN1A,SRD5A1,IDH1,KRT5,CAPNS1,ELF3,GSTA4,UCP2,S100A6,FLNA,MYH9,TMSB10,JUNB,SOX4,PDLIM7
1065	ZBTB14	891.5	ARCHS4 Coexpression,923;GTEx Coexpression,860	ARF1,EGF,PTGS1
1066	ZBTB41	891.7	ARCHS4 Coexpression,311;Enrichr Queries,1184;GTEx Coexpression,1180	TMEM123,CLIC4,CDKN1A,KLF6,RPS9,RPL18A,CAPZB,GSTA4,ARPC1B,CFL1
1067	ZNF84	892	ARCHS4 Coexpression,46;Enrichr Queries,1205;GTEx Coexpression,1425	EIF5A,TSC22D1,ACTN1,ARPC1B,CCND2,CAPNS1,CAPZB,GSTA4,FUT9,MYH9,TAGLN2,SOX4,S100A11
1068	FOXP3	892.3	Literature ChIP-seq,99;ARCHS4 Coexpression,1510;Enrichr Queries,968;GTEx Coexpression,992	COL1A1,CSTB,CD74,CDKN1A,KLF6,ANXA1,SERPINE1,SH3BGRL3,APOE,JUNB,ITM2B
1069	HSF4	893.7	ARCHS4 Coexpression,1614;Enrichr Queries,564;GTEx Coexpression,503	CSTB,CD74,CDKN1A,GSN,CLU,CDC42,COL1A1,LGALS3,ARPC2,NUPR1,APOE,JUNB,SOX4,IER3
1070	ZNF300	894.7	ARCHS4 Coexpression,459;Enrichr Queries,678;GTEx Coexpression,1547	CDKN1A,ANXA1,GPX3,ARPC1B,SERPINE1,CLU,UCHL1,CCND2,CAPZB,GSTA4,CYP1B1,PMEPA1,NUPR1,SOX4
1071	ZNF574	894.7	ARCHS4 Coexpression,509;Enrichr Queries,920;GTEx Coexpression,1255	RPS9,CDKN1A,ARF1,PKM,JUP,RPS5,CFL1,JUNB,ACTB
1072	EN1	895.3	ARCHS4 Coexpression,530;Enrichr Queries,973;GTEx Coexpression,1183	COL1A1,UCHL1,CCND2,CADM4,BASP1,CYP1B1,TIMP3,TMSB10,SOX4,RTN4
1073	FOXD3	896.3	ARCHS4 Coexpression,1303;Enrichr Queries,1126;GTEx Coexpression,260	LDHB,CADM4,RDH10,CYP1B1,LPL,ITGA6,APOE,SPTBN1,SOX4
1074	ZNF30	896.7	ARCHS4 Coexpression,460;Enrichr Queries,1041;GTEx Coexpression,1189	KLF6,CCND2,CAPNS1,GSTA4,IDH1,PMEPA1,MYH9,ITGA6,ATP1B1,SOX4
1075	TCFL5	897	ARCHS4 Coexpression,1142;Enrichr Queries,279;GTEx Coexpression,1270	CSTB,CLIC4,CDKN1A,ANXA1,TNFRSF12A,GPX3,SERPINE1,ATP1B1,COL1A1,KLF6,CCND2,PKM,BASP1,CYP1B1,PMEPA1,APOE,JUNB,SOX4,IER3
1076	ZNF557	897.7	ARCHS4 Coexpression,1175;Enrichr Queries,962;GTEx Coexpression,556	HSPA8,CDKN1A,KLF6,CCND2,CAPNS1,ARPC1B,MGAT4A,CTR9,JUNB,SOX4
1077	FERD3L	898.3	ARCHS4 Coexpression,1063;Enrichr Queries,1269;GTEx Coexpression,363	S100G,CDKN1A,MMP14,BASP1,MYOF,CYP1B1,TMSB10
1078	FOXD4L4	898.5	ARCHS4 Coexpression,1411;GTEx Coexpression,386	LPL,RTN4,YWHAG
1079	MEF2D	898.7	ARCHS4 Coexpression,998;Enrichr Queries,228;GTEx Coexpression,1470	HSPA8,CLIC4,CDKN1A,GSN,JUP,TSC22D1,ACTN1,ATP1B1,ACTB,RTN4,FAM129B,KLF6,CCND2,PKM,CAPNS1,FTH1,MYH9,JUNB,SOX4,SPTBN1,YWHAG
1080	POU1F1	901	ARCHS4 Coexpression,1157;Enrichr Queries,755;GTEx Coexpression,791	S100G,ARF1,CDKN1A,KLF6,FUT9,SERPINE1,LCN2,CYP1B1,JUNB,CP,IER3
1081	NPAS4	901.3	ARCHS4 Coexpression,1122;Enrichr Queries,683;GTEx Coexpression,899	HSPA8,CSTB,CDKN1A,GPX3,SERPINE1,LPL,COL1A1,KLF6,CCND2,PMEPA1,CALM1,JUNB,IER3
1082	ZNF77	902	ARCHS4 Coexpression,739;Enrichr Queries,794;GTEx Coexpression,1173	CDKN1A,KLF6,CCND2,TNFRSF12A,CAPNS1,UCP2,ISOC1,PMEPA1,MYH9,JUNB,ACTB,SOX4
1083	NKX31	902.5	ARCHS4 Coexpression,1065;GTEx Coexpression,740	PMEPA1,NUPR1
1084	ZNF292	903	ARCHS4 Coexpression,54;Enrichr Queries,1240;GTEx Coexpression,1415	EIF5A,CDKN1A,ACTN1,FAM129B,KLF6,PKM,CAPNS1,CAPZB,GSTA4,CFL1,BSG,TAGLN2,SOX4,S100A11
1085	ASCL4	903	ARCHS4 Coexpression,1191;Enrichr Queries,1197;GTEx Coexpression,321	SLC12A2,S100G,CDKN1A,JUP,BASP1,SRD5A1,KRT14,SDC1
1086	SCRT2	903.7	ARCHS4 Coexpression,446;Enrichr Queries,1164;GTEx Coexpression,1101	S100G,CDKN1A,UCHL1,CCND2,CADM4,GSTA4,BASP1,SOX4
1087	PRDM6	904	ARCHS4 Coexpression,1590;Enrichr Queries,1061;GTEx Coexpression,61	CLIC4,ACTN1,MYOF,DSTN,ACTB,COL1A1,MGP,PMEPA1,MYH9,FLNA,CYP1B1,TIMP3,PDLIM7
1088	EN2	904.3	ARCHS4 Coexpression,690;Enrichr Queries,899;GTEx Coexpression,1124	YWHAE,CDKN1A,KLF6,CCND2,CADM4,BASP1,CYP1B1,LPL,PMEPA1,TMSB10,SOX4
1089	DMRT1	904.5	Literature ChIP-seq,77;ARCHS4 Coexpression,1424;Enrichr Queries,820;GTEx Coexpression,1297	YWHAE,CDKN1A,CLIC4,CAPG,LRP2,ACTB,WFDC2,RPL7,CAPZB,BASP1,RACK1,CYP1B1,PMEPA1,FLNA,JUNB,TMSB10,SOX4,EIF5A,S100G,TSC22D1,ACTN1,KRT8,KRT7,ATP1B1,COL1A1,KLF6,KRT18,MGAT4A,CTNNB1,CALM1
1090	SEBOX	905	ARCHS4 Coexpression,1518;Enrichr Queries,292	IL33,S100G,CDKN1A,JUP,GPX3,LPL,CLU,CP,COL1A1,LGALS3,CCND2,KRT18,S100A6,LCN2,NUPR1,APOE,JUNB,IER3
1091	ZNF318	905	ARCHS4 Coexpression,1385;Enrichr Queries,883;ReMap ChIP-seq,52;GTEx Coexpression,1300	YWHAE,CLIC4,CDKN1A,SERPINE1,PRSS22,MYL12A,RTN4,RPL7,CTSL,FTH1,RDH10,TMSB4X,TMSB10,CLIC1,JUNB,YWHAG,SOX4,SPTBN1,RPS9,ANXA1,ACTN1,PDZK1IP1,ATP1B1,EEF1G,KLF6,PKM,EIF6,ELF3,S100A6,MYH9,TAGLN2
1092	ZNF618	905.3	ARCHS4 Coexpression,361;Enrichr Queries,835;GTEx Coexpression,1520	SLC12A2,YWHAE,JUP,ARPC1B,MYOF,MYL12A,COL1A1,KLF6,UCHL1,BASP1,RACK1,CYP1B1,PMEPA1,SOX4
1093	USF3	905.5	ARCHS4 Coexpression,470;GTEx Coexpression,1341	EIF5A,PKM,CFL1,BSG,RPLP0
1094	ZNF445	906.3	ARCHS4 Coexpression,1189;Enrichr Queries,880;GTEx Coexpression,650	HSPA8,CDKN1A,KLF6,LAS1L,TSC22D1,SRD5A1,MGAT4A,RAB11A,ACTB,SOX4,SPTBN1
1095	ZNF471	906.7	ARCHS4 Coexpression,1188;Enrichr Queries,1224;GTEx Coexpression,308	EIF5A,CDC42,CCND2,LAS1L,ARPC2,SRD5A1,CYP1B1,LPL
1096	INSM2	907	ARCHS4 Coexpression,854;Enrichr Queries,1243;GTEx Coexpression,624	S100G,UCHL1,CDKN1A,BASP1,RBP1,LRP2,YWHAG
1097	ZNF564	908	ARCHS4 Coexpression,1108;Enrichr Queries,864;GTEx Coexpression,752	YWHAE,CSTB,CDKN1A,KLF6,PKM,TSC22D1,CAPNS1,MGAT4A,CYP1B1,JUNB,SOX4
1098	ZNF667	909.3	ARCHS4 Coexpression,1194;Enrichr Queries,728;GTEx Coexpression,806	UCHL1,CDKN1A,CCND2,BASP1,SRD5A1,CYP1B1,PMEPA1,TIMP3,CLU,SOX4
1099	ZNF747	909.7	ARCHS4 Coexpression,467;Enrichr Queries,901;GTEx Coexpression,1361	CSTB,CLIC4,PKM,TPM4,FTH1,MYOF,MYH9,NUPR1,SOX4,S100A11,PDLIM7,S100A10
1100	TFCP2	910.3	ARCHS4 Coexpression,897;Enrichr Queries,448;GTEx Coexpression,1386	HSPA8,CDKN1A,GSN,TSC22D1,ACTN1,ACTB,RPL7,EEF1G,LDHB,KLF6,CCND2,EIF6,BASP1,MYH9,JUNB,SOX4
1101	ZNF211	910.3	ARCHS4 Coexpression,300;Enrichr Queries,877;GTEx Coexpression,1554	EIF5A,CDKN1A,TSC22D1,ATP1B1,FAM129B,KLF6,PKM,CAPNS1,CYP1B1,JUNB,SOX4,YWHAG,IER3
1102	ZBTB43	911	ARCHS4 Coexpression,301;Enrichr Queries,850;GTEx Coexpression,1582	EIF5A,CLIC4,CDKN1A,TSC22D1,ACTN1,ATP1B1,KLF6,PKM,CAPNS1,CFL1,BSG,JUNB,SOX4,IER3
1103	ZNF559	911.3	ARCHS4 Coexpression,200;Enrichr Queries,1082;GTEx Coexpression,1452	EIF5A,JUP,ATP1B1,FAM129B,KLF6,PKM,CAPNS1,BSG,CTNNB1,MYH9,FLNA,ITGA6,SOX4
1104	NPAS3	911.3	ARCHS4 Coexpression,699;Enrichr Queries,1299;GTEx Coexpression,736	YWHAE,CCND2,CADM4,BASP1,FUT9,TIMP3,MYH9
1105	ZNF836	912.3	ARCHS4 Coexpression,916;Enrichr Queries,1298;GTEx Coexpression,523	YWHAE,PKM,GSTA4,CAPNS1,BSG,TIMP3,SOX4
1106	BNC2	912.7	ARCHS4 Coexpression,447;Enrichr Queries,816;GTEx Coexpression,1475	COL1A1,CLIC4,ANXA1,GSN,TPM4,ANXA2,SERPINE1,MYOF,MGP,CYP1B1,TIMP3,NUPR1
1107	ZNF583	912.7	ARCHS4 Coexpression,1306;Enrichr Queries,988;GTEx Coexpression,444	CDKN1A,KLF6,CCND2,ANXA1,JUP,SERPINE1,SDC1,CYP1B1,MYH9,CAPG
1108	ZNF878	913	ARCHS4 Coexpression,835;GTEx Coexpression,991	LDHB,HSPA8,TMSB4X,IDH1
1109	ZNF267	914.7	ARCHS4 Coexpression,1564;Enrichr Queries,757;GTEx Coexpression,423	CLIC4,CDKN1A,ANXA1,ARPC1B,KLF6,CCND2,ARPC2,BASP1,TMSB4X,CYP1B1,JUNB,IER3
1110	ZNF681	914.7	ARCHS4 Coexpression,293;Enrichr Queries,1366;GTEx Coexpression,1085	CCND2,PKM,CAPNS1,ACTN1,BSG,MYH9,FLNA,FAM129B
1111	ZBTB46	916.3	ARCHS4 Coexpression,1030;Enrichr Queries,404;GTEx Coexpression,1315	CDKN1A,GSN,JUP,ACTN1,SERPINE1,COL1A1,KLF6,UCHL1,CCND2,BASP1,PMEPA1,TIMP3,APOE,JUNB,SOX4,IER3,S100A10
1112	ZNF141	916.7	ARCHS4 Coexpression,216;Enrichr Queries,1315;GTEx Coexpression,1219	EIF5A,CCND2,PKM,CAPNS1,CFL1,BSG,CYP1B1,MYH9,FAM129B
1113	ATF6B	918	ARCHS4 Coexpression,1580;GTEx Coexpression,256	RPS9,RPL18A,RACK1,MAP4
1114	TLX2	920.3	ARCHS4 Coexpression,1476;Enrichr Queries,890;GTEx Coexpression,395	COL1A1,S100G,CDKN1A,UCHL1,CCND2,GSTA4,IDH1,SERPINE1,CYP1B1,JUNB
1115	ZNF623	920.3	ARCHS4 Coexpression,516;Enrichr Queries,711;GTEx Coexpression,1534	CSTB,CLIC4,CDKN1A,SRD5A1,GSTO1,MYOF,TALDO1,ACTB,KLF6,EIF6,CAPZB,PMEPA1,JUNB,SOX4,IER3
1116	VAX2	921	ARCHS4 Coexpression,790;Enrichr Queries,724;GTEx Coexpression,1249	COL1A1,LGALS3,CSTB,CDKN1A,KRT18,SERPINE1,KRT8,CYP1B1,KRT7,LPL,RAC1,JUNB
1117	ZNF786	921	ARCHS4 Coexpression,899;Enrichr Queries,972;GTEx Coexpression,892	CDKN1A,KLF6,CAPNS1,TPM4,SRD5A1,BASP1,MYH9,JUNB,ACTB,SOX4
1118	ZNF408	921.7	ARCHS4 Coexpression,196;Enrichr Queries,1092;GTEx Coexpression,1477	EIF5A,CDKN1A,ARF1,RPS5,TALDO1,ACTB,PKM,EIF6,CFL1,JUNB,CLIC1,PDLIM7,IER3
1119	ZNF285	922.5	ARCHS4 Coexpression,952;GTEx Coexpression,893	SRD5A1,MYH9,CALM1
1120	ZNF488	923	ARCHS4 Coexpression,950;Enrichr Queries,896	CDKN1A,SLC6A19,CADM4,ANXA3,SERPINE1,CYP1B1,KRT7,PMEPA1,TIMP3
1121	ZNF692	923.3	ARCHS4 Coexpression,1579;Enrichr Queries,483;GTEx Coexpression,708	HSPA8,CSTB,CDKN1A,TSC22D1,ACTN1,ACTB,KLF6,PKM,LAS1L,MYH9,JUNB,SOX4,PDLIM7,IER3
1122	ZNF573	923.7	ARCHS4 Coexpression,233;Enrichr Queries,1202;GTEx Coexpression,1336	PKM,CAPNS1,GSTA4,BSG,CYP1B1,MYH9,FLNA,SOX4,IER3,FAM129B
1123	HOXD9	923.7	ARCHS4 Coexpression,1259;Enrichr Queries,980;GTEx Coexpression,532	COL1A1,CDKN1A,CCND2,SLC34A1,CYP1B1,LPL,TIMP3,PDLIM7,RPL7
1124	SIX6	923.7	ARCHS4 Coexpression,694;Enrichr Queries,1272;GTEx Coexpression,805	YWHAE,S100G,LDHB,ARF1,UCHL1,IDH1,CYP1B1
1125	PKNOX2	924	ARCHS4 Coexpression,1354;Enrichr Queries,906;GTEx Coexpression,512	COL1A1,CCND2,GSN,CYP1B1,LPL,PMEPA1,TIMP3,NUPR1,CALM1,YWHAG
1126	ZNF569	924.3	ARCHS4 Coexpression,160;Enrichr Queries,1146;GTEx Coexpression,1467	CDKN1A,ANXA1,CAPNS1,GSTA4,ACTN1,SERPINE1,MYH9,TAGLN2,JUNB,SOX4,S100A11,FAM129B
1127	EVX1	925	ARCHS4 Coexpression,1391;Enrichr Queries,1045;GTEx Coexpression,339	S100G,CDKN1A,CCND2,ELF3,GSTA4,IDH1,CYP1B1,SOX4
1128	NKX32	926.5	ARCHS4 Coexpression,1418;GTEx Coexpression,435	ACTN1,PDLIM7,PTGS1
1129	POU4F1	928.7	ARCHS4 Coexpression,264;Enrichr Queries,1137;GTEx Coexpression,1385	YWHAE,CDKN1A,UCHL1,CCND2,CADM4,BASP1,TMSB10,SOX4,YWHAG
1130	SAFB	929.5	ARCHS4 Coexpression,1028;GTEx Coexpression,831	EIF5A,PKM,MGST3
1131	ZBED4	930	ARCHS4 Coexpression,1132;Enrichr Queries,1036;GTEx Coexpression,622	EIF5A,EEF1G,CDKN1A,KLF6,TSC22D1,CTR9,MYH9,ITGA6,RPSA,SOX4
1132	ZKSCAN2	930.7	ARCHS4 Coexpression,138;Enrichr Queries,1118;GTEx Coexpression,1536	YWHAE,CDKN1A,ARPC1B,MYL12A,KLF6,CCND2,GSTA4,TAGLN2,SH3BGRL3,JUNB,SOX4,S100A11,ITM2B,S100A10
1133	ZNF148	931	ARCHS4 Coexpression,431;Enrichr Queries,869;GTEx Coexpression,1493	EIF5A,KLF6,RPS9,CFL1,BSG,ADAM10,PMEPA1,MYH9,CTNNB1,IQGAP1,RTN4,SPTBN1
1134	ZNF93	932.3	ARCHS4 Coexpression,438;Enrichr Queries,1172;GTEx Coexpression,1187	LDHB,CCND2,CAPNS1,IDH1,CYP1B1,TIMP3,MYH9,SOX4,FAM129B
1135	ZMAT4	932.7	ARCHS4 Coexpression,384;Enrichr Queries,1286;GTEx Coexpression,1128	S100G,UCHL1,GSTA4,FUT9,LPL,MYH9,TAGLN2,CALM1
1136	FOXI2	932.7	ARCHS4 Coexpression,1567;Enrichr Queries,1200;GTEx Coexpression,31	CSTB,ANXA1,PRSS27,PRSS22,RAB11A,WFDC2,FAM129B,RAB10,COL1A1,LY6D,CYP1B1,SFN,TRIM16,S100A14,SPRR1A,IVL,S100A10
1137	E2F2	933.3	ARCHS4 Coexpression,1346;Enrichr Queries,1248;GTEx Coexpression,206	PRDX2,CDKN1A,CCND2,ARPC2,TALDO1,SH3BGRL3,CLIC1,S100A11,IER3
1138	ZNF714	933.3	ARCHS4 Coexpression,409;Enrichr Queries,1356;GTEx Coexpression,1035	CAPNS1,CAPZB,MYH9,SOX4,FAM129B
1139	CEBPE	936	ARCHS4 Coexpression,1459;Enrichr Queries,504;GTEx Coexpression,845	CSTB,CDKN1A,ANXA1,ANXA2,ANXA3,SERPINE1,CAPG,CLU,LGALS3,KLF6,LCN2,SH3BGRL3,JUNB,IER3
1140	RFX1	937.3	ARCHS4 Coexpression,1413;Enrichr Queries,792;ReMap ChIP-seq,180;GTEx Coexpression,1364	VCP,CDKN1A,SERPINE1,PRSS22,ACTB,LGALS3,PRDX2,UCP2,JUNB,CLIC1,YWHAG,PDLIM7,SOX4,JUP,RPS5,ACTN1,KRT8,DSTN,ATP1B1,EEF1G,KLF6,KRT18,MYH9,TAGLN2
1141	DRGX	938.3	ARCHS4 Coexpression,1113;Enrichr Queries,1358;GTEx Coexpression,344	S100G,UCHL1,RTN4,YWHAG
1142	ZNF230	940.3	ARCHS4 Coexpression,524;Enrichr Queries,1111;GTEx Coexpression,1186	CDKN1A,KLF6,ANXA1,PKM,CAPNS1,CYP1B1,MAP4,JUNB,YWHAG,FAM129B
1143	ZNF337	940.3	ARCHS4 Coexpression,862;Enrichr Queries,1042;GTEx Coexpression,917	CDKN1A,KLF6,CCND2,LAS1L,FUT9,GSTO1,PMEPA1,JUNB,SOX4,IER3
1144	ISL1	942.3	ARCHS4 Coexpression,667;Enrichr Queries,595;GTEx Coexpression,1565	CDKN1A,GPX3,TMEM176B,TMEM176A,ATP1B1,COL1A1,UCHL1,KLF6,CCND2,BASP1,FXYD2,CYP1B1,TIMP3,SOX4
1145	ZNF534	944	ARCHS4 Coexpression,1408;GTEx Coexpression,480	LAS1L,FUT9,CALM1
1146	ZNF224	944.7	ARCHS4 Coexpression,463;Enrichr Queries,1229;GTEx Coexpression,1142	EIF5A,KLF6,PKM,CAPNS1,SRD5A1,CFL1,CYP1B1,JUNB
1147	ZNF577	945.3	ARCHS4 Coexpression,320;Enrichr Queries,1223;GTEx Coexpression,1293	EIF5A,CD74,CCND2,PKM,CAPNS1,BSG,CYP1B1,MYH9,FAM129B
1148	PAX4	945.3	ARCHS4 Coexpression,1517;Enrichr Queries,1008;GTEx Coexpression,311	LGALS3,CSTB,S100G,KRT19,CLDN4,CDKN1A,CLDN7,SERPINE1,KRT8
1149	ZNF804B	945.7	ARCHS4 Coexpression,1049;Enrichr Queries,1352;GTEx Coexpression,436	FUT9,KRT7,NUPR1,LRP2
1150	ZNF136	946.3	ARCHS4 Coexpression,273;Enrichr Queries,1060;GTEx Coexpression,1506	EIF5A,ANXA1,PKM,CAPNS1,ACTN1,BSG,PMEPA1,MYH9,JUNB,SOX4,IER3,FAM129B
1151	ZNF768	946.3	ARCHS4 Coexpression,892;Enrichr Queries,377;GTEx Coexpression,1570	CSTB,CDKN1A,JUP,ACTN1,EEF1G,EIF6,CAPNS1,FTH1,CFL1,BSG,NUPR1,JUNB,SOX4,S100A11,PDLIM7,IER3,S100A10
1152	GCM2	947.7	ARCHS4 Coexpression,1266;Enrichr Queries,1195;GTEx Coexpression,382	S100G,ARF1,CYP1B1,KRT7,CAPG,ATP1B1
1153	ZNF160	948	ARCHS4 Coexpression,645;Enrichr Queries,749;GTEx Coexpression,1450	EIF5A,CDKN1A,ANXA1,GSN,TSC22D1,CLU,KLF6,CAPNS1,CFL1,BSG,CTNNB1,JUNB,SOX4
1154	IKZF2	948.7	ARCHS4 Coexpression,1396;Enrichr Queries,572;GTEx Coexpression,878	CD74,CDKN1A,ANXA1,ATP1B1,KLF6,CCND2,TMSB4X,MGAT4A,CYP1B1,PMEPA1,TIMP3,ITGA6,SOX4
1155	ZNF790	949	ARCHS4 Coexpression,909;Enrichr Queries,878;GTEx Coexpression,1060	CDKN1A,UCHL1,KLF6,PKM,CAPNS1,BASP1,MYH9,ITGA6,CLU,JUNB,S100A10
1156	ZNF566	949.3	ARCHS4 Coexpression,792;Enrichr Queries,1006;GTEx Coexpression,1050	CDKN1A,CCND2,ANXA1,PKM,CAPNS1,PMEPA1,MYH9,ITGA6,IER3
1157	REXO4	950	ARCHS4 Coexpression,367;GTEx Coexpression,1533	EIF5A,EEF1G,RPS9,PKM,RPS5,RPSA
1158	NRL	950.3	ARCHS4 Coexpression,1168;Enrichr Queries,897;GTEx Coexpression,786	COL1A1,S100G,CDKN1A,KLF6,CCND2,GPX3,MYOF,JUNB,SOX4
1159	GTF2IRD2	951	ARCHS4 Coexpression,1515;Enrichr Queries,571;GTEx Coexpression,767	CDKN1A,GSN,TSC22D1,ACTB,LGALS3,KLF6,CCND2,CFL1,PMEPA1,NUPR1,JUNB,SOX4,IER3
1160	L3MBTL1	952	ARCHS4 Coexpression,1000;GTEx Coexpression,904	EIF5A,LAS1L,SRD5A1
1161	ZBTB40	953.7	ARCHS4 Coexpression,1085;Enrichr Queries,848;GTEx Coexpression,928	YWHAE,HSPA8,CLIC4,CDKN1A,KLF6,ACTN1,MYH9,IQGAP1,SOX4,S100A10
1162	ZNF763	954	ARCHS4 Coexpression,279;Enrichr Queries,1180;GTEx Coexpression,1403	EIF5A,CDKN1A,KLF6,PKM,GSTA4,CAPNS1,MYH9,JUNB,YWHAG,FAM129B
1163	MYCL	955.5	ARCHS4 Coexpression,1448;GTEx Coexpression,463	CERS3,JUP,SDC1
1164	ZNF621	956	ARCHS4 Coexpression,1024;Enrichr Queries,1260;GTEx Coexpression,584	MMP14,CDKN1A,KLF6,SRD5A1,CFL1,ADAM10,IQGAP1
1165	HOXD12	957	ARCHS4 Coexpression,1145;Enrichr Queries,1400;GTEx Coexpression,326	ANXA1,CAPZB,ACTN1,PDLIM7,PTGS1
1166	RAG1	957.3	ARCHS4 Coexpression,1384;Enrichr Queries,493;GTEx Coexpression,995	CD74,CDKN1A,ANXA1,GSN,ANXA2,IDH1,SERPINE1,COL1A1,LGALS3,CCND2,TIMP3,ITGA6,JUNB,SOX4
1167	ZNF28	957.7	ARCHS4 Coexpression,937;Enrichr Queries,1251;GTEx Coexpression,685	CDKN1A,CAPNS1,MGAT4A,CTR9,MYOF,MYH9
1168	ZNF671	958.3	ARCHS4 Coexpression,884;Enrichr Queries,860;GTEx Coexpression,1131	CDKN1A,KLF6,CCND2,ANXA1,MYOF,CYP1B1,PMEPA1,RAC1,JUNB,SOX4,FAM129B
1169	PLAGL2	959	ARCHS4 Coexpression,1425;Enrichr Queries,341;GTEx Coexpression,1111	CSTB,CLIC4,CDKN1A,ACTN1,IQGAP1,ATP1B1,ACTB,KLF6,CCND2,PKM,FTH1,MYH9,NUPR1,JUNB,SOX4,IER3,S100A10
1170	ZNF396	959.7	ARCHS4 Coexpression,1555;Enrichr Queries,855;GTEx Coexpression,469	CSTB,ELF3,GSTA4,ANXA3,FTH1,MGAT4A,TALDO1,NUPR1,FMO5,ITM2B,SOX4
1171	ZFHX4	959.7	ARCHS4 Coexpression,372;Enrichr Queries,914;GTEx Coexpression,1593	COL1A1,CDKN1A,UCHL1,CCND2,GSN,GSTA4,BASP1,FUT9,CYP1B1,TIMP3,TAGLN2,SOX4
1172	SAFB2	960	ARCHS4 Coexpression,1210;GTEx Coexpression,710	LAS1L,DSTN
1173	TBR1	961	ARCHS4 Coexpression,997;Enrichr Queries,1160;GTEx Coexpression,726	S100G,UCHL1,CCND2,BASP1,CTNNB1,ATP1B1,YWHAG
1174	NEUROG2	961.3	ARCHS4 Coexpression,979;Enrichr Queries,975;GTEx Coexpression,930	COL1A1,CDKN1A,CCND2,BASP1,TMSB4X,RBP1,CYP1B1,LRP2,SOX4
1175	SIX4	962.7	ARCHS4 Coexpression,1083;Enrichr Queries,358;GTEx Coexpression,1447	CDKN1A,TNFRSF12A,TSC22D1,SERPINE1,MYOF,ATP1B1,CLU,COL1A1,KLF6,CCND2,CYP1B1,PMEPA1,TIMP3,APOE,JUNB,SOX4,ITM2B,IER3
1176	ZNF605	963	ARCHS4 Coexpression,1011;Enrichr Queries,1161;GTEx Coexpression,717	EIF5A,CDKN1A,CCND2,PKM,ARPC2,MGAT4A,PMEPA1
1177	ZNF528	963	ARCHS4 Coexpression,1129;Enrichr Queries,936;GTEx Coexpression,824	EIF5A,CDKN1A,CCND2,PKM,LAS1L,CYP1B1,PMEPA1,TIMP3,CLU,SOX4
1178	MIXL1	964.7	ARCHS4 Coexpression,1372;Enrichr Queries,591;GTEx Coexpression,931	CDKN1A,SERPINE1,KRT8,COL1A1,KLF6,KRT19,CCND2,KRT18,ELF3,TMSB4X,MYH9,JUNB,IER3
1179	ZNF66	965.5	ARCHS4 Coexpression,1474;GTEx Coexpression,457	RPLP0,RPL11,RACK1
1180	ZNF517	966.3	ARCHS4 Coexpression,1166;Enrichr Queries,937;GTEx Coexpression,796	CDKN1A,GSN,LAS1L,JUP,TSC22D1,TPM4,JUNB,SOX4,PDLIM7
1181	PLAG1	966.7	ARCHS4 Coexpression,1573;Enrichr Queries,662;GTEx Coexpression,665	CDKN1A,KLF6,CCND2,GSN,SERPINE1,ADAM10,CYP1B1,PMEPA1,TIMP3,ITGA6,SOX4
1182	ZNF284	967.3	ARCHS4 Coexpression,733;Enrichr Queries,1230;GTEx Coexpression,939	CDKN1A,CAPNS1,BSG,RACK1,MYH9,CLU,SOX4
1183	GMEB2	968	ARCHS4 Coexpression,994;Enrichr Queries,566;GTEx Coexpression,1344	CSTB,CDKN1A,ACTN1,IQGAP1,KLF6,FTH1,MYH9,FLNA,NUPR1,JUNB,SOX4,PDLIM7,S100A10
1184	CENPA	968	ARCHS4 Coexpression,861;GTEx Coexpression,1075	EIF5A,RPLP0,RPSA
1185	NFATC3	969	ARCHS4 Coexpression,961;Enrichr Queries,628;GTEx Coexpression,1318	YWHAE,HSPA8,CLIC4,CDKN1A,ADAM10,IQGAP1,KLF6,UCHL1,CCND2,MYH9,ITGA6,JUNB,SPTBN1
1186	ZNF280C	969.7	ARCHS4 Coexpression,303;Enrichr Queries,1094;GTEx Coexpression,1512	SLC12A2,LDHB,CDKN1A,CCND2,CAPNS1,CAPZB,ARPC1B,FLNA,MYH9,TAGLN2,CLU
1187	CREBZF	971.7	ARCHS4 Coexpression,1296;Enrichr Queries,502;GTEx Coexpression,1117	HSPA8,CDKN1A,ACTN1,ATP1B1,ACTB,RTN4,KLF6,CCND2,MYH9,CTNNB1,JUNB,SOX4,SPTBN1
1188	ZNF513	971.7	ARCHS4 Coexpression,1419;Enrichr Queries,759;GTEx Coexpression,737	CSTB,ARF1,CDKN1A,FTH1,NUPR1,JUNB,SOX4,S100A11,PDLIM7,S100A10
1189	HOXD11	972.3	ARCHS4 Coexpression,1349;Enrichr Queries,895;GTEx Coexpression,673	COL1A1,EIF5A,TMEM123,CDKN1A,CCND2,SERPINE1,CYP1B1,KRT7,JUNB,PDLIM7
1190	ZNF304	972.7	ARCHS4 Coexpression,1106;Enrichr Queries,743;GTEx Coexpression,1069	CLIC4,JUP,GSTA4,BASP1,CYP1B1,TIMP3,SH3BGRL3,CLU,JUNB,SOX4,IER3
1191	ZSCAN10	972.7	ARCHS4 Coexpression,1319;Enrichr Queries,609;GTEx Coexpression,990	S100G,CDKN1A,TNFRSF12A,KRT8,WFDC2,COL1A1,LDHB,CLDN4,KRT18,ELF3,CLDN7,APOE,MICU1
1192	ZNF547	973	ARCHS4 Coexpression,776;Enrichr Queries,964;GTEx Coexpression,1179	CDKN1A,KLF6,ANXA1,ANXA2,CAPNS1,SERPINE1,MYH9,FLNA,CLU,SOX4
1193	ZNF670	973	ARCHS4 Coexpression,805;Enrichr Queries,1141	LDHB,CDKN1A,EIF6,IDH1,ATP1B1,SOX4,IER3
1194	ZNF257	973.7	ARCHS4 Coexpression,980;Enrichr Queries,1183;GTEx Coexpression,758	ANXA1,PKM,TMSB4X,CYP1B1,TIMP3,MYH9,APOE
1195	DMBX1	974	ARCHS4 Coexpression,1243;Enrichr Queries,976;GTEx Coexpression,703	COL1A1,ARF1,KLF6,SERPINE1,CLDN7,CYP1B1,MYH9,LRP2
1196	SMYD3	974.5	ARCHS4 Coexpression,1519;GTEx Coexpression,430	PKM,CALM1,MICU1
1197	HES7	974.7	ARCHS4 Coexpression,1279;Enrichr Queries,395;GTEx Coexpression,1250	CSTB,CDKN1A,TNFRSF12A,SERPINE1,CLU,COL1A1,KLF6,CCND2,ELF3,S100A6,RACK1,CYP1B1,NUPR1,JUNB,SOX4,IER3
1198	NHLH1	974.7	ARCHS4 Coexpression,825;Enrichr Queries,1067;GTEx Coexpression,1032	S100G,CDKN1A,UCHL1,CCND2,ELF3,BASP1,RBP1,JUNB
1199	NFE4	975	GTEx Coexpression,975	TALDO1
1200	ZKSCAN3	975.7	ARCHS4 Coexpression,1605;Enrichr Queries,453;GTEx Coexpression,869	YWHAE,HSPA8,CSTB,CDKN1A,TSC22D1,ATP1B1,KLF6,CCND2,TIMP3,MYH9,ITGA6,APOE,JUNB,SOX4,S100A10
1201	L3MBTL4	976.5	ARCHS4 Coexpression,1619;GTEx Coexpression,334	CERS3,JUP,KRT14,SDC1
1202	TIGD4	976.7	ARCHS4 Coexpression,418;Enrichr Queries,909;GTEx Coexpression,1603	COL1A1,IL33,S100G,LDHB,CAPNS1,GSTA4,IDH1,LPL,TIMP3,MYH9,CAPG,IER3
1203	CC2D1A	977	ARCHS4 Coexpression,1612;GTEx Coexpression,342	CLDN4,CTSL,CLDN7
1204	ZNF791	978.3	ARCHS4 Coexpression,1527;Enrichr Queries,750;GTEx Coexpression,658	YWHAE,CLIC4,CDKN1A,KLF6,ANXA1,FTH1,BSG,ADAM10,JUNB,SOX4,IER3
1205	FOXJ3	979	ARCHS4 Coexpression,1447;Enrichr Queries,565;GTEx Coexpression,925	HSPA8,CLIC4,CDKN1A,ADAM10,IQGAP1,ATP1B1,RTN4,KLF6,EIF6,CFL1,MYH9,SOX4,IER3
1206	NEUROD2	980	Literature ChIP-seq,132;ARCHS4 Coexpression,1494;Enrichr Queries,1151;GTEx Coexpression,1143	YWHAE,S100G,CADM4,KRT7,CDC42,KLF6,KRT19,CCND2,ARPC2,CAPZB,BASP1,CFL1,LY6D,RPL14,ITGA6,TRIM16,SOX4,YWHAG
1207	PRDM2	980.3	ARCHS4 Coexpression,1062;Enrichr Queries,1025;GTEx Coexpression,854	CDKN1A,KLF6,CCND2,EIF6,GSTO1,CFL1,MYH9,RTN4,SPTBN1
1208	ARID3C	981	ARCHS4 Coexpression,1101;GTEx Coexpression,861	TMEM176A,FMO5
1209	TIGD1	983	ARCHS4 Coexpression,1172;GTEx Coexpression,794	LAS1L,SRD5A1
1210	ZBTB8A	983.5	ARCHS4 Coexpression,1617;GTEx Coexpression,350	CLIC4,GSN,TIMP3
1211	ZSCAN23	983.7	ARCHS4 Coexpression,312;Enrichr Queries,1309;GTEx Coexpression,1330	CAPNS1,CAPZB,GSTA4,ARPC1B,TAGLN2,SOX4,PTGS1
1212	ZNF17	984.7	ARCHS4 Coexpression,313;Enrichr Queries,1227;GTEx Coexpression,1414	CCND2,TSC22D1,CAPNS1,GSTA4,ACTN1,CYP1B1,MYH9,SOX4,FAM129B
1213	PAX3	985.3	ARCHS4 Coexpression,648;Enrichr Queries,1074;GTEx Coexpression,1234	COL1A1,YWHAE,CDKN1A,CCND2,GSTA4,BASP1,CYP1B1,CTNNB1,LRP2
1214	NFX1	987.3	ARCHS4 Coexpression,1178;Enrichr Queries,717;GTEx Coexpression,1067	HSPA8,CDKN1A,KLF6,PKM,EIF6,SRD5A1,MYH9,IQGAP1,ACTB,RTN4,SPTBN1
1215	VAX1	987.7	ARCHS4 Coexpression,870;Enrichr Queries,1396;GTEx Coexpression,697	YWHAE,GSTA4,RACK1
1216	PRDM9	988.3	ARCHS4 Coexpression,610;Enrichr Queries,956;GTEx Coexpression,1399	YWHAE,EEF1G,CSTB,S100G,CDKN1A,ARF1,BASP1,LPL,ITGA6,RPSA,JUNB
1217	NKX25	988.5	ARCHS4 Coexpression,1625;GTEx Coexpression,352	LDHB,MGST3,MYL12A
1218	FIGLA	990	ARCHS4 Coexpression,1535;Enrichr Queries,1338;GTEx Coexpression,97	EEF1B2,S100G,RPS9,PKM,RPS5,RBP1,RPL14,RPS13
1219	ZNF571	992	ARCHS4 Coexpression,445;Enrichr Queries,1093;GTEx Coexpression,1438	ANXA1,CAPNS1,IDH1,CYP1B1,PMEPA1,MYH9,JUNB,FMO5,SOX4,FAM129B
1220	POU4F3	993	ARCHS4 Coexpression,1462;Enrichr Queries,1397;GTEx Coexpression,120	CLIC4,DSTN,PMEPA1,MYH9,FLNA,ACTB
1221	ATOH7	993.3	ARCHS4 Coexpression,840;Enrichr Queries,1139;GTEx Coexpression,1001	S100G,UCHL1,CCND2,IQGAP1,CALM1,JUNB,SOX4
1222	KDM2A	994.5	ARCHS4 Coexpression,974;GTEx Coexpression,1015	MYH9,IQGAP1
1223	ZNF746	994.7	ARCHS4 Coexpression,1388;Enrichr Queries,312;GTEx Coexpression,1284	HSPA8,CSTB,CDKN1A,ANXA1,TNFRSF12A,ACTN1,SERPINE1,ACTB,KLF6,CCND2,BASP1,FTH1,S100A6,CYP1B1,MYH9,JUNB,IER3
1224	ZNF26	994.7	ARCHS4 Coexpression,1183;Enrichr Queries,915;GTEx Coexpression,886	CDKN1A,KLF6,TSC22D1,SRD5A1,RPLP0,SERPINE1,JUNB,SOX4,IER3
1225	HOXA10	995.3	ARCHS4 Coexpression,1008;Enrichr Queries,549;GTEx Coexpression,1429	EIF5A,CDKN1A,ANXA1,GPX3,SERPINE1,COL1A1,EEF1G,CCND2,CYP1B1,TIMP3,NUPR1,APOE,SOX4,IER3
1226	THAP6	995.7	ARCHS4 Coexpression,603;Enrichr Queries,1157;GTEx Coexpression,1227	HSPA8,CDKN1A,KLF6,ANXA1,PKM,CAPNS1,BSG,MYH9
1227	ZNF597	996	ARCHS4 Coexpression,1562;Enrichr Queries,579;GTEx Coexpression,847	CSTB,CDKN1A,ANXA1,TNFRSF12A,CTR9,SERPINE1,KLF6,CCND2,LCN2,CYP1B1,JUNB,IER3
1228	FOXN3	996	ARCHS4 Coexpression,1079;Enrichr Queries,629;GTEx Coexpression,1280	CLIC4,CDKN1A,GSN,TSC22D1,TNFRSF12A,ACTN1,KLF6,CCND2,EIF6,TIMP3,MYH9,SOX4,SPTBN1
1229	ZSCAN5C	996.3	ARCHS4 Coexpression,1472;Enrichr Queries,1095;GTEx Coexpression,422	LARS2,PKM,GSTO1,BASP1,FUT9,MYOF,ITGA6,MAP4
1230	ZNF766	996.5	ARCHS4 Coexpression,1198;Enrichr Queries,1369;ReMap ChIP-seq,262;GTEx Coexpression,1157	PRDX2,RPS9,PKM,ANXA1,ANXA2,CAPNS1,FTH1,PDZK1IP1,JUNB

1231	ZNF135	996.7	ARCHS4 Coexpression,697;Enrichr Queries,863;GTEx Coexpression,1430	EIF5A,CSTB,CDKN1A,CCND2,PKM,BASP1,PMEPA1,TIMP3,MYH9,APOE,SOX4
1232	ZNF491	997.3	ARCHS4 Coexpression,534;Enrichr Queries,1312;GTEx Coexpression,1146	LGALS3,CDKN1A,GSTA4,CAPNS1,S100A6,SOX4
1233	GATAD2B	997.7	ARCHS4 Coexpression,1067;Enrichr Queries,886;GTEx Coexpression,1040	HSPA8,CDKN1A,KLF6,CCND2,EIF6,GSTO1,MYH9,ACTB,RTN4,SPTBN1
1234	ZNF502	998.3	ARCHS4 Coexpression,1457;Enrichr Queries,753;GTEx Coexpression,785	CDKN1A,CCND2,ANXA1,ANXA3,CFL1,CYP1B1,PMEPA1,ITGA6,SOX4,IER3
1235	THAP9	998.7	ARCHS4 Coexpression,117;Enrichr Queries,1355;GTEx Coexpression,1524	CLIC4,PKM,CAPNS1,ACTN1,CFL1,MYH9,FLNA,TAGLN2,SOX4,FAM129B
1236	ZNF439	999	ARCHS4 Coexpression,775;Enrichr Queries,658;GTEx Coexpression,1564	EIF5A,CDKN1A,ANXA1,SERPINE1,ATP1B1,CCND2,PKM,CAPNS1,MGAT4A,CYP1B1,TIMP3,JUNB,SOX4
1237	CARF	999	ARCHS4 Coexpression,596;GTEx Coexpression,1402	EIF5A,PKM,CFL1,BSG
1238	TBX4	1000	ARCHS4 Coexpression,1445;Enrichr Queries,982;GTEx Coexpression,574	COL1A1,CDKN1A,TIMP3,CYP1B1,KRT7,TAGLN2,SOX4
1239	MGA	1000	ARCHS4 Coexpression,228;Enrichr Queries,1231;GTEx Coexpression,1542	ARF1,EIF6,CAPNS1,CAPZB,CFL1,TAGLN2,IQGAP1,RAC1,SPTBN1,PDLIM7
1240	ZNF653	1000	ARCHS4 Coexpression,1219;Enrichr Queries,856;GTEx Coexpression,926	PRDX2,CSTB,CDKN1A,PKM,LAS1L,JUP,CAPNS1,BSG,JUNB,PDLIM7
1241	ZNF607	1001	ARCHS4 Coexpression,399;Enrichr Queries,1296;GTEx Coexpression,1307	YWHAE,UCHL1,CCND2,ARPC1B,CYP1B1,SH3BGRL3,MYL12A,S100A11
1242	PITX2	1002	ARCHS4 Coexpression,1366;Enrichr Queries,365;GTEx Coexpression,1274	CDKN1A,ANXA1,ANXA3,GPX3,IDH1,SERPINE1,KRT8,CLU,COL1A1,KLF6,KRT19,CCND2,KRT18,CYP1B1,PMEPA1,TIMP3,SOX4
1243	ZNF713	1002	ARCHS4 Coexpression,1597;Enrichr Queries,935;GTEx Coexpression,475	YWHAE,KLF6,MGAT4A,SERPINE1,CYP1B1,MAP4,LRP2,CLIC1,JUNB,SOX4
1244	POU3F4	1003	ARCHS4 Coexpression,374;Enrichr Queries,1238;GTEx Coexpression,1397	CCND2,CADM4,GSTA4,BASP1,LPL,IQGAP1,JUNB,TMSB10,SOX4
1245	RXRG	1004	ARCHS4 Coexpression,1452;Enrichr Queries,392;GTEx Coexpression,1168	IL33,CD74,CDKN1A,GPX3,SERPINE1,LPL,CLU,COL1A1,LGALS3,CCND2,MGP,PMEPA1,TIMP3,APOE,JUNB
1246	RFX3	1005	ARCHS4 Coexpression,876;Enrichr Queries,1104;GTEx Coexpression,1034	EIF5A,CDKN1A,KLF6,TSC22D1,GSTA4,FUT9,ITGA6,SOX4
1247	GMEB1	1005	ARCHS4 Coexpression,1235;Enrichr Queries,785;GTEx Coexpression,994	CSTB,CLIC4,CDKN1A,KLF6,ACTN1,BSG,PMEPA1,SH3BGRL3,JUNB,ACTB,SOX4
1248	PROP1	1005	ARCHS4 Coexpression,959;Enrichr Queries,722;GTEx Coexpression,1334	CSTB,S100G,CDKN1A,KLF6,KRT19,SERPINE1,TMEM176A,LCN2,KRT7,JUNB,RTN4
1249	ZNF611	1006	ARCHS4 Coexpression,987;Enrichr Queries,1024;GTEx Coexpression,1007	CDKN1A,KLF6,PKM,ANXA2,CAPNS1,ADAM10,CYP1B1,ACTB,SOX4
1250	BATF2	1006	ARCHS4 Coexpression,1290;Enrichr Queries,910;GTEx Coexpression,819	CSTB,CD74,CDKN1A,ARPC1B,SERPINE1,LCN2,NUPR1,S100A11,IER3
1251	ZNF780B	1007	ARCHS4 Coexpression,176;Enrichr Queries,1321;GTEx Coexpression,1523	EIF5A,ARF1,CAPNS1,CAPZB,SRD5A1,MGAT4A,CFL1,BSG,CLIC1,SOX4
1252	ZNF709	1008	ARCHS4 Coexpression,450;Enrichr Queries,1262;GTEx Coexpression,1311	KLF6,CCND2,CAPNS1,GSTA4,ACTN1,MYH9,SOX4
1253	SP140	1008	ARCHS4 Coexpression,1239;Enrichr Queries,1328;GTEx Coexpression,456	CD74,ARPC2,TMSB4X,UCP2,MYOF
1254	ZNF525	1008	ARCHS4 Coexpression,595;GTEx Coexpression,1421	PKM,CAPNS1,BSG,PDLIM7
1255	ZNF765	1008	ARCHS4 Coexpression,1173;Enrichr Queries,1368;GTEx Coexpression,483	CAPNS1,CTR9,RPLP0,RACK1,IQGAP1
1256	ZFP41	1009	ARCHS4 Coexpression,1190;Enrichr Queries,885;GTEx Coexpression,951	COL1A1,UCHL1,CDKN1A,CCND2,TSC22D1,PMEPA1,FLNA,ITGA6,APOE,SOX4
1257	HELT	1009	ARCHS4 Coexpression,1431;Enrichr Queries,1267;GTEx Coexpression,329	CERS3,S100G,CCND2,JUP,KRT14,SDC1
1258	ZNF732	1009	ARCHS4 Coexpression,1121;GTEx Coexpression,897	LDHB,LAS1L,IDH1
1259	TIGD7	1009	ARCHS4 Coexpression,731;Enrichr Queries,1127;GTEx Coexpression,1170	CDKN1A,KLF6,CAPNS1,GSTA4,CYP1B1,PMEPA1,MYH9
1260	THAP3	1010	ARCHS4 Coexpression,848;Enrichr Queries,611;GTEx Coexpression,1571	CSTB,RPS9,CDKN1A,GSN,JUP,RPL11,IQGAP1,KLF6,EIF6,S100A6,MYH9,SH3BGRL3,APOE,JUNB
1261	ZNF587	1010	ARCHS4 Coexpression,1213;Enrichr Queries,1221;GTEx Coexpression,597	CDKN1A,KLF6,KRT18,CFL1,KRT8,SOX4
1262	LHX5	1011	ARCHS4 Coexpression,903;Enrichr Queries,1277;GTEx Coexpression,852	S100G,CDKN1A,UCHL1,FUT9,BASP1,RBP1
1263	TTF1	1012	ARCHS4 Coexpression,789;GTEx Coexpression,1235	CAPNS1,MYH9,FAM129B
1264	LIN28B	1012	ARCHS4 Coexpression,827;Enrichr Queries,916;GTEx Coexpression,1294	COL1A1,YWHAE,LDHB,CDKN1A,CCND2,IDH1,TIMP3,ITGA6,LRP2,SOX4
1265	ZNF484	1013	ARCHS4 Coexpression,552;Enrichr Queries,1305;GTEx Coexpression,1181	CDKN1A,ANXA1,CAPNS1,CAPZB,GSTA4,FUT9
1266	ZNF548	1013	ARCHS4 Coexpression,1221;Enrichr Queries,1236;GTEx Coexpression,581	YWHAE,KLF6,CAPNS1,JUNB,SOX4
1267	ZNF329	1014	ARCHS4 Coexpression,1621;Enrichr Queries,484;GTEx Coexpression,936	CDKN1A,ANXA1,ANXA3,ATP1B1,KLF6,CCND2,BASP1,CYP1B1,TIMP3,ITGA6,CALM1,JUNB,SOX4,IER3
1268	ZKSCAN5	1015	ARCHS4 Coexpression,947;Enrichr Queries,745;GTEx Coexpression,1352	HSPA8,CDKN1A,KLF6,TSC22D1,CAPNS1,PMEPA1,TAGLN2,JUNB,ACTB,SOX4,IER3
1269	ZNF316	1015	ARCHS4 Coexpression,1164;Enrichr Queries,1098;GTEx Coexpression,783	YWHAE,CDKN1A,MYH9,JUNB,PDLIM7,IER3
1270	ZNF560	1015	ARCHS4 Coexpression,786;Enrichr Queries,1115;GTEx Coexpression,1145	COL1A1,LDHB,CDKN1A,UCHL1,IDH1,CYP1B1,LPL,SOX4
1271	AKNA	1017	ARCHS4 Coexpression,1473;Enrichr Queries,637;GTEx Coexpression,940	CD74,CDKN1A,KLF6,CCND2,ANXA1,BASP1,UCP2,MYH9,FLNA,CAPG,JUNB
1272	SOX14	1017	ARCHS4 Coexpression,1057;Enrichr Queries,1401;GTEx Coexpression,593	UCHL1,TALDO1,YWHAG
1273	ZNF319	1018	ARCHS4 Coexpression,1522;Enrichr Queries,503;GTEx Coexpression,1030	CDKN1A,JUP,TSC22D1,ACTN1,COL1A1,KLF6,CCND2,PMEPA1,MYH9,FLNA,JUNB,SOX4,PDLIM7
1274	PTF1A	1020	ARCHS4 Coexpression,1022;Enrichr Queries,1391;GTEx Coexpression,646	EGF,MGAT4A,RBP1
1275	SKI	1021	ARCHS4 Coexpression,568;GTEx Coexpression,1474	MYH9,FLNA,MAP4,YWHAG
1276	ZNF518A	1021	ARCHS4 Coexpression,173;Enrichr Queries,1290;GTEx Coexpression,1600	EIF5A,KLF6,ARF1,ANXA1,CAPNS1,CAPZB,ARPC1B,CFL1,TAGLN2,SH3BGRL3
1277	SP110	1022	ARCHS4 Coexpression,1538;Enrichr Queries,648;GTEx Coexpression,879	LGALS3,CD74,CDKN1A,KLF6,ANXA1,ARPC1B,SERPINE1,MYOF,NUPR1,JUNB,IER3
1278	CAMTA2	1022	ARCHS4 Coexpression,1604;Enrichr Queries,574;GTEx Coexpression,888	CDKN1A,GSN,ACTN1,ATP1B1,ACTB,KLF6,CCND2,PKM,FTH1,MYH9,APOE,CALM1,JUNB
1279	NPAS1	1023	ARCHS4 Coexpression,977;Enrichr Queries,594;GTEx Coexpression,1498	EIF5A,CSTB,S100G,CDKN1A,SERPINE1,WFDC2,COL1A1,KLF6,CCND2,BSG,CYP1B1,APOE,JUNB
1280	DOT1L	1024	ARCHS4 Coexpression,1321;Enrichr Queries,505;GTEx Coexpression,1246	CLIC4,CDKN1A,JUP,ACTN1,ATP1B1,FAM129B,COL1A1,KLF6,CCND2,MYH9,FLNA,CTNNB1,JUNB,IER3
1281	ZNF343	1025	ARCHS4 Coexpression,1098;Enrichr Queries,967;GTEx Coexpression,1010	YWHAE,HSPA8,CDKN1A,KLF6,PKM,MGP,MYH9,ACTB,ITM2B,PTGS1
1282	BAZ2A	1026	ARCHS4 Coexpression,1272;Enrichr Queries,451;GTEx Coexpression,1356	HSPA8,CDKN1A,JUP,ACTB,RTN4,KLF6,PKM,EIF6,MYH9,FLNA,CTNNB1,MAP4,JUNB,SPTBN1
1283	ZNF510	1026	ARCHS4 Coexpression,873;Enrichr Queries,1341;GTEx Coexpression,865	TSC22D1,SH3BGRL3,CALM1,SPTBN1,YWHAG
1284	ZNF771	1029	ARCHS4 Coexpression,1056;Enrichr Queries,651;GTEx Coexpression,1381	CSTB,FTH1,ACTN1,CFL1,MYH9,NUPR1,IQGAP1,JUNB,SOX4,S100A11,PDLIM7,S100A10
1285	ZNF730	1032	ARCHS4 Coexpression,953;GTEx Coexpression,1110	LDHB,IDH1
1286	ZNF341	1032	ARCHS4 Coexpression,1314;Enrichr Queries,645;GTEx Coexpression,1136	HSPA8,CSTB,CDKN1A,KLF6,PKM,ELF3,PMEPA1,JUNB,ACTB,PDLIM7,YWHAG,FAM129B
1287	ZNF782	1034	ARCHS4 Coexpression,590;Enrichr Queries,1071;GTEx Coexpression,1441	CDKN1A,ARF1,PKM,CAPZB,CFL1,CYP1B1,PMEPA1,CLU,PDLIM7
1288	ANKZF1	1034	ARCHS4 Coexpression,933;Enrichr Queries,575;GTEx Coexpression,1594	HSPA8,CDKN1A,CLIC4,GSN,JUP,TSC22D1,ANXA2,DSTN,KLF6,PKM,ITGA6,JUNB,SOX4,IER3
1289	ANHX	1034	ARCHS4 Coexpression,1042;GTEx Coexpression,1026	RPLP0,RPSA
1290	ZNF19	1034	ARCHS4 Coexpression,964;Enrichr Queries,1073;GTEx Coexpression,1066	CSTB,CDKN1A,KLF6,TSC22D1,SRD5A1,NUPR1,SOX4
1291	SCRT1	1035	ARCHS4 Coexpression,746;Enrichr Queries,1324	S100G,CDKN1A,CADM4,BASP1,YWHAG
1292	ZNF100	1035	ARCHS4 Coexpression,306;Enrichr Queries,1388;GTEx Coexpression,1411	PKM,CAPNS1,BSG,MYH9,FLNA,FAM129B
1293	MTERF3	1035	ARCHS4 Coexpression,804;GTEx Coexpression,1266	LDHB,IDH1,MYH9
1294	ZBTB34	1035	ARCHS4 Coexpression,1109;Enrichr Queries,871;GTEx Coexpression,1126	SLC12A2,CDKN1A,KLF6,CCND2,TSC22D1,PMEPA1,IQGAP1,JUNB,SOX4,PTGS1
1295	ZNF648	1037	ARCHS4 Coexpression,763;Enrichr Queries,1322;GTEx Coexpression,1027	KLF6,MYOF,PRSS22,CP,WFDC2
1296	ZNF80	1038	ARCHS4 Coexpression,1116;Enrichr Queries,1143;GTEx Coexpression,856	COL1A1,YWHAE,LGALS3,ANXA1,UCP2,MAP4,JUNB
1297	LEUTX	1039	ARCHS4 Coexpression,1019;Enrichr Queries,1295;GTEx Coexpression,803	CCND2,TMSB4X,RPS5,RPS13,IER3
1298	ZNF512B	1041	ARCHS4 Coexpression,1187;Enrichr Queries,375;GTEx Coexpression,1560	CSTB,CDKN1A,ACTN1,IQGAP1,ATP1B1,COL1A1,CCND2,PKM,FTH1,MYH9,FLNA,NUPR1,APOE,JUNB,SOX4,PDLIM7,S100A10
1299	ZFPM1	1041	ARCHS4 Coexpression,1541;Enrichr Queries,407;GTEx Coexpression,1175	CDKN1A,GSN,JUP,ACTN1,ATP1B1,ACTB,FAM129B,COL1A1,KLF6,CCND2,PMEPA1,MYH9,APOE,JUNB,SOX4
1300	BHLHA9	1041	ARCHS4 Coexpression,1565;GTEx Coexpression,517	UCHL1,YWHAG
1301	MYT1	1042	ARCHS4 Coexpression,787;Enrichr Queries,1259;GTEx Coexpression,1079	S100G,CDKN1A,UCHL1,CADM4,BASP1,SOX4
1302	ZNF324	1042	ARCHS4 Coexpression,1543;Enrichr Queries,638;GTEx Coexpression,944	CDKN1A,JUP,TNFRSF12A,SRD5A1,COL1A1,KLF6,PKM,PMEPA1,MYH9,JUNB,SOX4,IER3
1303	ZSCAN30	1042	ARCHS4 Coexpression,1233;GTEx Coexpression,851	EIF6,SRD5A1
1304	ZIC4	1042	ARCHS4 Coexpression,647;Enrichr Queries,1274;GTEx Coexpression,1205	COL1A1,YWHAE,UCHL1,CADM4,GSTA4,CYP1B1
1305	ZNF544	1043	ARCHS4 Coexpression,1007;Enrichr Queries,703;GTEx Coexpression,1420	CDKN1A,KLF6,CCND2,TSC22D1,CAPNS1,BASP1,MYOF,CYP1B1,MYH9,ITGA6,SOX4,IER3
1306	PRDM12	1045	ARCHS4 Coexpression,1353;Enrichr Queries,1354;GTEx Coexpression,428	UCHL1,CYP1B1,CALM1,YWHAG
1307	ZNF555	1046	ARCHS4 Coexpression,711;Enrichr Queries,965;GTEx Coexpression,1463	EIF5A,CDKN1A,KLF6,PKM,JUP,TNFRSF12A,TSC22D1,CAPNS1,MGAT4A,MYH9,SOX4
1308	ZNF570	1047	ARCHS4 Coexpression,567;Enrichr Queries,1380;GTEx Coexpression,1193	PKM,CAPNS1,BSG,MYH9
1309	ZSCAN5B	1047	ARCHS4 Coexpression,1224;Enrichr Queries,788;GTEx Coexpression,1129	S100G,CDKN1A,SERPINE1,CLDN7,MYOF,KRT7,NUPR1,APOE,RPS13,IER3
1310	ZNF740	1048	ARCHS4 Coexpression,1566;Enrichr Queries,866;ReMap ChIP-seq,208;GTEx Coexpression,1552	EIF5A,CDKN1A,TPM4,JUP,TSC22D1,ARPC1B,ACTB,FAM129B,EEF1G,CDC42,PRDX2,KLF6,CCND2,BSG,CLDN7,RPL14,FLNA,MYH9,JUNB,YWHAG,SOX4,IER3
1311	ZNF629	1048	ARCHS4 Coexpression,1440;Enrichr Queries,244;GTEx Coexpression,1461	CSTB,CDKN1A,JUP,TSC22D1,ACTN1,SERPINE1,MYOF,ATP1B1,CLU,ACTB,COL1A1,KLF6,CCND2,CYP1B1,SDC1,MYH9,ITGA6,JUNB,SOX4
1312	ZNF449	1049	ARCHS4 Coexpression,720;Enrichr Queries,945;GTEx Coexpression,1483	CDKN1A,CCND2,ANXA1,GSTA4,FUT9,ARPC1B,NUPR1,JUNB,SOX4,IER3
1313	CCDC17	1050	ARCHS4 Coexpression,1103;GTEx Coexpression,997	ELF3,TAGLN2,WFDC2
1314	ZNF429	1051	ARCHS4 Coexpression,797;Enrichr Queries,1317;GTEx Coexpression,1039	CDKN1A,CCND2,PKM,CAPNS1,SRD5A1
1315	RORB	1051	ARCHS4 Coexpression,885;Enrichr Queries,777;GTEx Coexpression,1492	CDKN1A,CCND2,ANXA1,BASP1,FUT9,MGP,LPL,TIMP3,ATP1B1,CALM1,RTN4
1316	PHF21A	1052	ARCHS4 Coexpression,1454;Enrichr Queries,633;GTEx Coexpression,1068	CDKN1A,KLF6,CCND2,JUP,TSC22D1,MYH9,CTNNB1,ITGA6,ATP1B1,JUNB,SOX4
1317	SRCAP	1052	ARCHS4 Coexpression,1280;Enrichr Queries,301;GTEx Coexpression,1576	HSPA8,CDKN1A,JUP,ACTN1,IQGAP1,ATP1B1,ACTB,KLF6,PKM,FTH1,MYH9,FLNA,CTNNB1,JUNB,SOX4,SPTBN1,YWHAG,IER3
1318	ZNF778	1053	ARCHS4 Coexpression,1587;Enrichr Queries,559;GTEx Coexpression,1013	HSPA8,CDKN1A,TSC22D1,ACTB,RTN4,KLF6,PKM,FTH1,SDC1,JUNB,SPTBN1,IER3
1319	ZNF215	1055	ARCHS4 Coexpression,995;Enrichr Queries,1171;GTEx Coexpression,998	UCHL1,CCND2,CAPNS1,CYP1B1,MYH9,RPSA,ITGA6
1320	RBAK	1055	ARCHS4 Coexpression,928;Enrichr Queries,1166;GTEx Coexpression,1071	CDKN1A,CCND2,SH3BGRL3,IQGAP1,JUNB,IER3
1321	MYSM1	1055	ARCHS4 Coexpression,1229;Enrichr Queries,1304;GTEx Coexpression,632	KLF6,EIF6,RPL11,CFL1,RPL7
1322	ZNF835	1055	ARCHS4 Coexpression,1161;Enrichr Queries,1225;GTEx Coexpression,779	UCHL1,CYP1B1,TIMP3,MYH9,CALM1
1323	ZNF554	1055	ARCHS4 Coexpression,1511;Enrichr Queries,1019;GTEx Coexpression,636	CDKN1A,GSN,TSC22D1,NUPR1,CALM1,MICU1,CLU,SOX4
1324	ZBTB32	1057	ARCHS4 Coexpression,1283;Enrichr Queries,809;GTEx Coexpression,1080	LGALS3,CSTB,CD74,CDKN1A,CCND2,SERPINE1,CAPG,JUNB,IER3
1325	ZNF398	1057	ARCHS4 Coexpression,1446;Enrichr Queries,1108;GTEx Coexpression,618	CERS3,KLF6,ARPC2,CYP1B1,JUNB,SOX4,YWHAG
1326	EMX2	1059	ARCHS4 Coexpression,1390;Enrichr Queries,1009;GTEx Coexpression,777	COL1A1,CCND2,TSC22D1,CYP1B1,LPL,TIMP3,CLU
1327	ZNF551	1059	ARCHS4 Coexpression,612;Enrichr Queries,1147;GTEx Coexpression,1417	CDKN1A,CAPNS1,BSG,ITGA6,RAC1,JUNB,IER3,FAM129B
1328	NKX63	1059	ARCHS4 Coexpression,1141;GTEx Coexpression,977	FUT9,MGAT4A
1329	VENTX	1059	ARCHS4 Coexpression,1215;Enrichr Queries,984;GTEx Coexpression,978	CSTB,KLF6,PKM,TNFRSF12A,BASP1,ARPC1B,CYP1B1,APOE
1330	ZIC5	1060	ARCHS4 Coexpression,1041;Enrichr Queries,919;GTEx Coexpression,1221	COL1A1,YWHAE,LDHB,CDKN1A,KLF6,CCND2,LPL,LRP2,SOX4
1331	E2F3	1061	ARCHS4 Coexpression,1401;Enrichr Queries,675;GTEx Coexpression,1107	HSPA8,CDKN1A,KLF6,CCND2,PKM,BASP1,ACTN1,MYH9,JUNB,SOX4
1332	SGSM2	1064	ARCHS4 Coexpression,1154;GTEx Coexpression,973	UCHL1,CADM4
1333	PROX2	1064	ARCHS4 Coexpression,1249;Enrichr Queries,1106;GTEx Coexpression,836	EIF5A,S100G,CDKN1A,SERPINE1,RACK1,PMEPA1,SOX4
1334	ZNF641	1064	ARCHS4 Coexpression,1288;Enrichr Queries,585;GTEx Coexpression,1319	CDKN1A,KLF6,CCND2,GSN,ANXA2,SERPINE1,CYP1B1,ATP1B1,JUNB,ITM2B,IER3,S100A10
1335	NEUROD4	1064	ARCHS4 Coexpression,1163;Enrichr Queries,1234;GTEx Coexpression,795	S100G,ARF1,CDKN1A,SERPINE1,SOX4
1336	RAX2	1064	ARCHS4 Coexpression,1378;Enrichr Queries,519;GTEx Coexpression,1295	CSTB,CDKN1A,JUP,ACTB,PKM,ELF3,FTH1,S100A6,CYP1B1,MYH9,JUNB,SOX4,IER3
1337	UNCX	1065	ARCHS4 Coexpression,760;Enrichr Queries,1392;GTEx Coexpression,1042	UCHL1,BASP1,RBP1
1338	BARHL2	1065	ARCHS4 Coexpression,758;Enrichr Queries,1398;GTEx Coexpression,1038	UCHL1,BASP1,TMSB10
1339	ZNF480	1066	ARCHS4 Coexpression,932;Enrichr Queries,1013;GTEx Coexpression,1252	ANXA1,CAPNS1,BASP1,SRD5A1,CYP1B1,ITGA6,SOX4,IER3
1340	FBXL19	1068	ARCHS4 Coexpression,958;GTEx Coexpression,1177	PKM,YWHAG
1341	FOXR1	1068	ARCHS4 Coexpression,1003;Enrichr Queries,1049;GTEx Coexpression,1152	RPS5,NUPR1,IQGAP1,S100A11,PDLIM7,RPS13,S100A10
1342	ZNF705A	1068	ARCHS4 Coexpression,1469;Enrichr Queries,231;GTEx Coexpression,1504	CD74,CDKN1A,ANXA3,GPX3,IDH1,TMEM176B,TMEM176A,LPL,CP,COL1A1,LGALS3,MMP14,KLF6,CCND2,MGP,LCN2,CYP1B1,JUNB,IER3
1343	ZNF446	1068	ARCHS4 Coexpression,1428;Enrichr Queries,1002;GTEx Coexpression,775	CDKN1A,KLF6,LAS1L,MYH9,JUNB,PDLIM7,IER3
1344	GTF2IRD1	1069	ARCHS4 Coexpression,839;GTEx Coexpression,1298	ARPC1B,TAGLN2,ITM2B
1345	ZBTB17	1069	ARCHS4 Coexpression,1380;Enrichr Queries,300;GTEx Coexpression,1527	CSTB,CLIC4,CDKN1A,GSN,TSC22D1,ACTN1,CAPG,ACTB,FAM129B,KLF6,CCND2,PKM,CAPZB,MYH9,CTNNB1,SH3BGRL3,JUNB,SOX4,IER3
1346	NR2E3	1071	ARCHS4 Coexpression,1149;Enrichr Queries,992	CSTB,S100G,CDKN1A,KLF6,ELF3,SRD5A1,SOX4
1347	E4F1	1071	Literature ChIP-seq,163;ARCHS4 Coexpression,1496;Enrichr Queries,1039;GTEx Coexpression,1585	CDKN1A,KLF6,MYH9,APOE,JUNB,PDLIM7
1348	ZBTB8B	1072	ARCHS4 Coexpression,1558;GTEx Coexpression,585	YWHAE,UCHL1
1349	FOXD4	1072	ARCHS4 Coexpression,1351;Enrichr Queries,819;GTEx Coexpression,1045	S100G,CDKN1A,UCHL1,CCND2,KRT18,SERPINE1,CYP1B1,APOE,JUNB
1350	ARHGAP35	1072	ARCHS4 Coexpression,583;GTEx Coexpression,1561	CADM4,MAP4,SPTBN1,YWHAG
1351	ZNF250	1072	ARCHS4 Coexpression,1020;Enrichr Queries,689;GTEx Coexpression,1507	COL1A1,CDKN1A,KLF6,CCND2,TSC22D1,CAPNS1,PMEPA1,TIMP3,MYH9,ITGA6,JUNB,SOX4
1352	ZFPM2	1072	ARCHS4 Coexpression,1352;Enrichr Queries,738;GTEx Coexpression,1127	COL1A1,CCND2,BASP1,SRD5A1,SERPINE1,MGP,CYP1B1,LPL,TIMP3,CLU
1353	IKZF3	1073	ARCHS4 Coexpression,1169;Enrichr Queries,1212;GTEx Coexpression,837	YWHAE,CD74,CDKN1A,CCND2,TMSB4X
1354	ZNF85	1073	ARCHS4 Coexpression,435;Enrichr Queries,1330;GTEx Coexpression,1455	PKM,CAPNS1,CYP1B1,MYH9,FLNA,SOX4,FAM129B
1355	RXRB	1075	ARCHS4 Coexpression,1227;Enrichr Queries,402;GTEx Coexpression,1596	HSPA8,CSTB,CDKN1A,GSN,RPLP0,ACTB,KLF6,PKM,CAPNS1,FTH1,CFL1,MYH9,CTNNB1,APOE,JUNB,IER3
1356	PRR12	1075	ARCHS4 Coexpression,986;Enrichr Queries,868;GTEx Coexpression,1371	CDKN1A,JUP,MYH9,FLNA,JUNB,ACTB,SOX4,SPTBN1
1357	YY2	1076	ARCHS4 Coexpression,1627;Enrichr Queries,769;GTEx Coexpression,832	CDKN1A,KLF6,CCND2,TPM4,PMEPA1,ITGA6,JUNB,SOX4,IER3,PTGS1
1358	ZNF718	1077	ARCHS4 Coexpression,297;Enrichr Queries,1376;GTEx Coexpression,1557	PKM,CAPNS1,CYP1B1,MYH9,MAP4,YWHAG,FAM129B
1359	GTF2I	1078	ARCHS4 Coexpression,1078	CTNNB1,YWHAG
1360	NR6A1	1078	ARCHS4 Coexpression,917;Enrichr Queries,991;GTEx Coexpression,1326	YWHAE,LDHB,CDKN1A,KLF6,CCND2,IDH1,TIMP3,ITGA6,SOX4
1361	ZNF674	1079	ARCHS4 Coexpression,1589;Enrichr Queries,510;GTEx Coexpression,1137	HSPA8,CDKN1A,ANXA1,ANXA2,RPS5,ACTB,KLF6,BASP1,TMSB4X,CYP1B1,MYH9,IER3
1362	FOXB2	1084	ARCHS4 Coexpression,1393;Enrichr Queries,1331;GTEx Coexpression,528	UCHL1,CYP1B1,YWHAG
1363	ZNF500	1085	ARCHS4 Coexpression,1623;Enrichr Queries,1003;GTEx Coexpression,628	YWHAE,RPS9,CLIC4,KLF6,RPL14,JUNB,PDLIM7,SOX4
1364	ZNF526	1087	ARCHS4 Coexpression,1144;Enrichr Queries,812;GTEx Coexpression,1305	CDKN1A,CCND2,PKM,JUP,ACTN1,BSG,FLNA,JUNB,YWHAG,IER3
1365	CHAMP1	1088	ARCHS4 Coexpression,1367;GTEx Coexpression,809	EIF5A,CTR9
1366	ZBTB44	1088	ARCHS4 Coexpression,475;Enrichr Queries,1319;GTEx Coexpression,1471	KLF6,ARF1,TNFRSF12A,FTH1,CFL1,RTN4,PDLIM7
1367	HOMEZ	1090	ARCHS4 Coexpression,1120;Enrichr Queries,631;GTEx Coexpression,1518	CSTB,CDKN1A,CLIC4,GSN,TSC22D1,CLU,KLF6,CCND2,RACK1,MYH9,JUNB,SOX4,IER3
1368	ZBTB39	1090	ARCHS4 Coexpression,1293;Enrichr Queries,1030;GTEx Coexpression,946	CLIC4,CDKN1A,KLF6,CCND2,TSC22D1,CTR9,RPSA,SOX4
1369	ZNF773	1090	ARCHS4 Coexpression,1023;Enrichr Queries,1185;GTEx Coexpression,1061	CSTB,CDKN1A,TNFRSF12A,CAPNS1,SRD5A1,JUNB
1370	KIN	1090	ARCHS4 Coexpression,957;Enrichr Queries,1038;GTEx Coexpression,1276	LGALS3,HSPA8,CDKN1A,KLF6,EIF6,CAPNS1,PRSS27,JUNB
1371	VSX2	1090	ARCHS4 Coexpression,1414;Enrichr Queries,1336;GTEx Coexpression,521	YWHAE,CYP1B1,RTN4
1372	PCGF6	1091	ARCHS4 Coexpression,793;Enrichr Queries,1255;GTEx Coexpression,1224	LDHB,CDKN1A,CCND2,CAPNS1,MYH9,ITGA6
1373	ZNF800	1091	ARCHS4 Coexpression,730;Enrichr Queries,1110;GTEx Coexpression,1432	YWHAE,CDKN1A,KLF6,PMEPA1,JUNB,SPTBN1
1374	POU2AF1	1091	ARCHS4 Coexpression,1237;GTEx Coexpression,945	TMSB4X,NUPR1
1375	ZNF425	1092	ARCHS4 Coexpression,1167;Enrichr Queries,941;GTEx Coexpression,1169	CDKN1A,TSC22D1,FTH1,SERPINE1,MYH9,NUPR1,JUNB,SOX4
1376	LMX1A	1095	ARCHS4 Coexpression,693;Enrichr Queries,1215;GTEx Coexpression,1377	COL1A1,KRT18,BASP1,LRP2,TMSB10,SOX4,RPL7
1377	SP9	1097	ARCHS4 Coexpression,880;GTEx Coexpression,1313	TSC22D1,GSTA4,SOX4
1378	ZNF333	1098	ARCHS4 Coexpression,1531;Enrichr Queries,799;GTEx Coexpression,965	LARS2,LGALS3,CDKN1A,KLF6,GSN,TSC22D1,FTH1,JUNB,SOX4,SPTBN1
1379	MXD3	1099	ARCHS4 Coexpression,1479;Enrichr Queries,867;GTEx Coexpression,952	CSTB,CDKN1A,TALDO1,JUNB,SOX4,S100A11,PDLIM7,IER3,S100A10
1380	ZNF516	1099	ARCHS4 Coexpression,1165;Enrichr Queries,787;GTEx Coexpression,1346	CDKN1A,KLF6,UCHL1,CCND2,MYOF,PMEPA1,ITGA6,JUNB,SOX4,IER3
1381	BSX	1099	ARCHS4 Coexpression,1383;Enrichr Queries,1337;GTEx Coexpression,578	S100G,UCHL1,YWHAG,MYL12A
1382	ZNF780A	1100	ARCHS4 Coexpression,1192;Enrichr Queries,1348;GTEx Coexpression,759	CDKN1A,SRD5A1,ARPC1B
1383	MYF6	1100	ARCHS4 Coexpression,1330;Enrichr Queries,598;GTEx Coexpression,1372	COL1A1,LGALS3,CSTB,CDKN1A,GPX3,SERPINE1,MGP,LCN2,LPL,MAP4,ATP1B1,JUNB
1384	ZNF519	1101	ARCHS4 Coexpression,965;Enrichr Queries,1345;GTEx Coexpression,993	CDKN1A,LAS1L,CAPNS1,SRD5A1
1385	FLYWCH1	1102	ARCHS4 Coexpression,1624;GTEx Coexpression,579	LAS1L,CTR9
1386	DMRTC2	1103	ARCHS4 Coexpression,1415;Enrichr Queries,664;GTEx Coexpression,1230	COL1A1,CSTB,S100G,CLDN4,CDKN1A,TNFRSF12A,SERPINE1,KRT8,LCN2,LPL
1387	ZNF367	1103	ARCHS4 Coexpression,751;Enrichr Queries,1346;GTEx Coexpression,1213	CDKN1A,EGF,TAGLN2,PTGS1
1388	GFI1	1104	ARCHS4 Coexpression,1403;Enrichr Queries,642;GTEx Coexpression,1268	LGALS3,CD74,CDKN1A,KLF6,CCND2,ANXA1,ANXA2,CYP1B1,ATP1B1,JUNB,IER3
1389	TBX15	1105	ARCHS4 Coexpression,1300;Enrichr Queries,599;GTEx Coexpression,1416	COL1A1,CDKN1A,ANXA1,GSN,GPX3,SERPINE1,MGP,CYP1B1,LPL,PMEPA1,TIMP3,MAP4
1390	ZNF213	1106	ARCHS4 Coexpression,1389;Enrichr Queries,795;GTEx Coexpression,1135	CDKN1A,KLF6,PKM,JUP,BSG,MYH9,JUNB,SOX4,PDLIM7
1391	ZNF493	1107	ARCHS4 Coexpression,1212;Enrichr Queries,1214;GTEx Coexpression,895	LAS1L,TSC22D1,SRD5A1,MGAT4A,SOX4
1392	HIC2	1107	ARCHS4 Coexpression,1117;Enrichr Queries,940;GTEx Coexpression,1265	CDKN1A,KLF6,CCND2,FTH1,RPL11,RPSA,APOE,JUNB,SOX4
1393	ZNF382	1108	ARCHS4 Coexpression,1247;Enrichr Queries,911;GTEx Coexpression,1165	CDKN1A,CCND2,SRD5A1,MGAT4A,CYP1B1,LPL,CLU,SOX4
1394	JRK	1109	ARCHS4 Coexpression,1407;GTEx Coexpression,811	CTR9
1395	TBPL1	1109	ARCHS4 Coexpression,692;GTEx Coexpression,1526	CAPNS1,MYH9,RAB11A,FAM129B
1396	ZFHX2	1111	ARCHS4 Coexpression,1068;Enrichr Queries,942;GTEx Coexpression,1324	CDKN1A,UCHL1,CCND2,TSC22D1,BASP1,PMEPA1,ATP1B1,JUNB,SOX4
1397	ZNF45	1112	ARCHS4 Coexpression,1040;Enrichr Queries,1145;GTEx Coexpression,1150	LDHB,HSPA8,ANXA1,CYP1B1,PMEPA1,SOX4
1398	ZNF407	1112	ARCHS4 Coexpression,1138;Enrichr Queries,1178;GTEx Coexpression,1019	MYOF,PMEPA1,MYH9,IQGAP1,SPTBN1
1399	ZNF157	1112	ARCHS4 Coexpression,1267;Enrichr Queries,1022;GTEx Coexpression,1047	CSTB,S100G,JUP,ELF3,FUT9,PRSS27,SOX4
1400	BHLHE23	1114	ARCHS4 Coexpression,1304;GTEx Coexpression,923	TMSB4X,RACK1
1401	ASCL5	1115	ARCHS4 Coexpression,1371;GTEx Coexpression,858	EGF,RACK1
1402	ZNF626	1116	ARCHS4 Coexpression,646;Enrichr Queries,1135;GTEx Coexpression,1568	EIF5A,CCND2,PKM,CAPNS1,BASP1,MGAT4A,CYP1B1,MYH9
1403	ZNF397	1118	ARCHS4 Coexpression,779;GTEx Coexpression,1457	PKM,CAPNS1,BSG
1404	ZNF717	1119	ARCHS4 Coexpression,1159;Enrichr Queries,1192;GTEx Coexpression,1005	ANXA1,SRD5A1,RPLP0,MYOF,ITGA6
1405	DLX6	1120	ARCHS4 Coexpression,764;Enrichr Queries,1198;GTEx Coexpression,1398	GSTA4,CYP1B1,LPL,MYH9,SOX4
1406	ZNF683	1121	ARCHS4 Coexpression,678;Enrichr Queries,1122;GTEx Coexpression,1562	LGALS3,CD74,ARPC2,UCP2,CAPG,SH3BGRL3,JUNB,MYL12A

1407	ZNF688	1121	ARCHS4 Coexpression,1489;Enrichr Queries,647;GTEx Coexpression,1228	CSTB,CDKN1A,FTH1,CFL1,NUPR1,JUNB,SOX4,S100A11,PDLIM7,S100A10
1408	ZNF724	1122	ARCHS4 Coexpression,1095;GTEx Coexpression,1148	CAPNS1,MYH9
1409	ZNF831	1123	ARCHS4 Coexpression,1230;Enrichr Queries,1270;GTEx Coexpression,870	CCND2,UCP2,JUNB,S100A10
1410	ZNF695	1124	ARCHS4 Coexpression,732;Enrichr Queries,1387;GTEx Coexpression,1254	LDHB,CAPNS1,MYH9
1411	ZNF7	1125	ARCHS4 Coexpression,1501;Enrichr Queries,775;GTEx Coexpression,1098	HSPA8,CSTB,CDKN1A,KLF6,PMEPA1,MYH9,JUNB,SOX4,IER3
1412	TBX22	1125	ARCHS4 Coexpression,824;Enrichr Queries,1204;GTEx Coexpression,1347	MIOX,IL33,S100G,LDHB,RPL18A,IDH1
1413	TFAP2D	1126	ARCHS4 Coexpression,1361;Enrichr Queries,1278;GTEx Coexpression,738	YWHAE,S100G,FUT9,TMSB10
1414	TBX1	1127	ARCHS4 Coexpression,1482;Enrichr Queries,589;GTEx Coexpression,1309	COL1A1,CSTB,CDKN1A,CCND2,GSN,MGP,CYP1B1,PMEPA1,TIMP3,JUNB,SOX4
1415	ZNF660	1129	ARCHS4 Coexpression,910;Enrichr Queries,1154;GTEx Coexpression,1322	CCND2,GSN,PKM,CAPNS1,MYH9,SOX4,PTGS1
1416	RHOXF2	1130	ARCHS4 Coexpression,1374;Enrichr Queries,538;GTEx Coexpression,1478	S100G,CDKN1A,ANXA2,SERPINE1,RPLP0,KRT8,CLU,COL1A1,KRT18,MYH9,FLNA,S100A11,PDLIM7
1417	ZNF543	1133	ARCHS4 Coexpression,1135;Enrichr Queries,1228;GTEx Coexpression,1036	KLF6,CCND2,MYH9,JUNB
1418	TLX3	1133	ARCHS4 Coexpression,844;Enrichr Queries,1271;GTEx Coexpression,1285	EIF5A,UCHL1,CCND2,BASP1,CYP1B1
1419	PRDM13	1133	ARCHS4 Coexpression,1069;Enrichr Queries,1349;GTEx Coexpression,982	CCND2,TMSB4X,RACK1,RPSA
1420	NOTO	1133	ARCHS4 Coexpression,1481;Enrichr Queries,1403;GTEx Coexpression,516	FUT9,KRT8
1421	ZNF865	1135	ARCHS4 Coexpression,988;GTEx Coexpression,1282	CAPNS1,MYH9
1422	TIGD3	1135	ARCHS4 Coexpression,1305;Enrichr Queries,802;GTEx Coexpression,1299	COL1A1,CSTB,CDKN1A,KLF6,TNFRSF12A,ANXA3,GPX3,JUNB,ITM2B,IER3
1423	HMX2	1136	ARCHS4 Coexpression,1373;Enrichr Queries,1048;GTEx Coexpression,987	CSTB,S100G,CDKN1A,TMSB4X,CYP1B1,JUNB,ACTB
1424	ZNF385C	1136	ARCHS4 Coexpression,1509;Enrichr Queries,803;GTEx Coexpression,1096	COL1A1,CD74,CDKN1A,JUP,ELF3,SERPINE1,KRT8,IER3,PTGS1
1425	NOBOX	1138	ARCHS4 Coexpression,1442;Enrichr Queries,833;GTEx Coexpression,1138	LGALS3,S100G,KLF6,KRT18,SERPINE1,KRT8,KRT5,LRP2
1426	RHOXF1	1138	ARCHS4 Coexpression,1471;Enrichr Queries,1035;GTEx Coexpression,907	COL1A1,KRT19,LAS1L,ELF3,ANXA3,GPX3,KRT14
1427	NAIF1	1140	ARCHS4 Coexpression,1379;GTEx Coexpression,900	LGALS3,TALDO1
1428	HSFY2	1140	ARCHS4 Coexpression,1386;GTEx Coexpression,894	CALM1
1429	FOXR2	1140	ARCHS4 Coexpression,1242;Enrichr Queries,726;GTEx Coexpression,1453	IL33,S100G,CDKN1A,CCND2,PKM,BASP1,FUT9,GPX3,LRP2,IER3
1430	ZNF689	1141	ARCHS4 Coexpression,1080;Enrichr Queries,953;GTEx Coexpression,1389	CSTB,LDHB,KLF6,ACTN1,IDH1,SOX4,PDLIM7,IER3,S100A10
1431	KLF14	1142	ARCHS4 Coexpression,1064;Enrichr Queries,1219	CDKN1A,RACK1,CYP1B1,RPSA,SOX4
1432	ZNF169	1142	ARCHS4 Coexpression,1497;Enrichr Queries,987;GTEx Coexpression,943	CSTB,CDKN1A,PKM,BASP1,MYH9,IER3,PTGS1
1433	ZNF20	1144	ARCHS4 Coexpression,1287;Enrichr Queries,709;GTEx Coexpression,1435	CDKN1A,KLF6,CCND2,ANXA1,TPM4,TSC22D1,SERPINE1,MYH9,CALM1,SOX4,S100A10
1434	HES3	1144	ARCHS4 Coexpression,1406;Enrichr Queries,1150;GTEx Coexpression,876	S100G,CDKN1A,KRT19,CALM1,JUNB
1435	FAM200B	1145	ARCHS4 Coexpression,846;GTEx Coexpression,1443	PKM,MYH9,FLNA
1436	TMF1	1145	ARCHS4 Coexpression,684;GTEx Coexpression,1605	EIF5A,RPS5,CFL1,BSG
1437	ZNF232	1146	ARCHS4 Coexpression,1099;Enrichr Queries,1055;GTEx Coexpression,1283	CSTB,LDHB,CYP1B1,MYH9,ITGA6,JUNB,SOX4
1438	MYT1L	1146	ARCHS4 Coexpression,1059;Enrichr Queries,1367;GTEx Coexpression,1011	FUT9,CALM1,ATP1B1
1439	ZNF501	1146	ARCHS4 Coexpression,938;Enrichr Queries,1167;GTEx Coexpression,1332	CSTB,ANXA1,CAPNS1,CYP1B1,TIMP3,MYH9
1440	ZNF781	1146	ARCHS4 Coexpression,1231;Enrichr Queries,1316;GTEx Coexpression,891	CCND2,LAS1L,SRD5A1,CYP1B1
1441	ZNF677	1147	ARCHS4 Coexpression,820;Enrichr Queries,1113;GTEx Coexpression,1508	UCHL1,CCND2,PKM,CAPNS1,GPX3,CYP1B1,TIMP3,MYH9
1442	PRMT3	1149	ARCHS4 Coexpression,761;Enrichr Queries,1320;GTEx Coexpression,1367	HSPA8,LDHB,ANXA2,LAS1L
1443	ZBTB48	1150	ARCHS4 Coexpression,1348;Enrichr Queries,719;GTEx Coexpression,1383	CDKN1A,KLF6,PKM,TNFRSF12A,RPL11,UCP2,TALDO1,SH3BGRL3,ATP1B1,JUNB,PDLIM7
1444	ZNF154	1151	ARCHS4 Coexpression,778;Enrichr Queries,1100;GTEx Coexpression,1574	EIF5A,LGALS3,CCND2,PKM,CAPNS1,CYP1B1,TIMP3,ITGA6
1445	TLX1	1151	ARCHS4 Coexpression,1435;Enrichr Queries,1395;GTEx Coexpression,623	CD74,UCP2
1446	ZNF414	1153	ARCHS4 Coexpression,1398;Enrichr Queries,553;GTEx Coexpression,1509	CSTB,CDKN1A,GSN,TSC22D1,ACTB,PKM,FTH1,S100A6,SH3BGRL3,APOE,JUNB,PDLIM7
1447	ZNF234	1159	ARCHS4 Coexpression,973;Enrichr Queries,1241;GTEx Coexpression,1263	KLF6,CAPNS1,BSG,SOX4,IER3
1448	ZBTB25	1160	ARCHS4 Coexpression,1601;Enrichr Queries,774;GTEx Coexpression,1106	CDKN1A,KLF6,CCND2,PKM,TSC22D1,ATP1B1,JUNB,SOX4,IER3
1449	GSX1	1161	ARCHS4 Coexpression,1525;Enrichr Queries,1399;GTEx Coexpression,560	YWHAE,UCHL1
1450	ZNF568	1162	ARCHS4 Coexpression,1046;Enrichr Queries,1382;GTEx Coexpression,1058	CAPNS1,MYH9
1451	ZNF506	1162	ARCHS4 Coexpression,1569;Enrichr Queries,1158;GTEx Coexpression,760	CD74,VCP,CCND2,MGAT4A,SOX4
1452	AHCTF1	1165	ARCHS4 Coexpression,1248;Enrichr Queries,957;GTEx Coexpression,1291	HSPA8,KLF6,EIF6,MYH9,FLNA,IQGAP1,ACTB,RTN4
1453	ARID3B	1166	ARCHS4 Coexpression,871;GTEx Coexpression,1460	MYOF,DSTN,FAM129B
1454	ZNF287	1166	ARCHS4 Coexpression,992;Enrichr Queries,1085;GTEx Coexpression,1422	COL1A1,CDKN1A,CAPNS1,GSTA4,PMEPA1,SOX4,IER3
1455	ZNF776	1166	ARCHS4 Coexpression,1029;Enrichr Queries,1226;GTEx Coexpression,1244	TSC22D1,CAPNS1,SRD5A1,MGAT4A,SOX4
1456	BARHL1	1167	ARCHS4 Coexpression,948;Enrichr Queries,1342;GTEx Coexpression,1210	S100G,CADM4,YWHAG
1457	INSM1	1167	ARCHS4 Coexpression,1127;Enrichr Queries,932;GTEx Coexpression,1442	S100G,CDKN1A,UCHL1,CCND2,BASP1,CYP1B1,LPL,SOX4
1458	SPEN	1168	ARCHS4 Coexpression,1513;GTEx Coexpression,822	CTNNB1
1459	TPRX1	1169	ARCHS4 Coexpression,1217;Enrichr Queries,1000;GTEx Coexpression,1290	CD74,S100G,CDKN1A,CCND2,KRT8,RPS13,IER3
1460	ZNF728	1169	ARCHS4 Coexpression,990;GTEx Coexpression,1348	CAPNS1,MYH9
1461	ZNF527	1169	ARCHS4 Coexpression,1155;Enrichr Queries,1384;GTEx Coexpression,969	PRSS27,CAPG
1462	HMX1	1171	ARCHS4 Coexpression,1568;Enrichr Queries,1263;GTEx Coexpression,683	YWHAE,S100G,CYP1B1
1463	CPEB1	1172	ARCHS4 Coexpression,1299;GTEx Coexpression,1044	MYH9
1464	HMX3	1172	ARCHS4 Coexpression,1409;Enrichr Queries,1268;GTEx Coexpression,838	CCND2,TMSB4X,CYP1B1
1465	DBX1	1172	ARCHS4 Coexpression,1370;Enrichr Queries,1389;GTEx Coexpression,757	ARF1,CCND2
1466	ZNF236	1175	ARCHS4 Coexpression,836;Enrichr Queries,1378;GTEx Coexpression,1310	EIF6,CFL1,CLIC1
1467	LHX3	1175	ARCHS4 Coexpression,1430;Enrichr Queries,1340;GTEx Coexpression,755	ARF1,SOX4
1468	PRDM15	1176	ARCHS4 Coexpression,1532;Enrichr Queries,966;GTEx Coexpression,1029	CDKN1A,KLF6,CCND2,PMEPA1,MYH9,ITGA6,JUNB
1469	MSANTD1	1177	ARCHS4 Coexpression,927;GTEx Coexpression,1426	CAPNS1,ARPC1B
1470	ZNF735	1177	ARCHS4 Coexpression,1181;GTEx Coexpression,1172	RPS13
1471	SOX5	1177	ARCHS4 Coexpression,627;Enrichr Queries,1297;GTEx Coexpression,1606	CCND2,GSTA4,FUT9,TIMP3,SOX4
1472	ZNF79	1178	ARCHS4 Coexpression,1264;Enrichr Queries,861;GTEx Coexpression,1408	CSTB,CDKN1A,KLF6,TNFRSF12A,SERPINE1,JUNB,SOX4,RTN4,IER3
1473	CENPS	1179	ARCHS4 Coexpression,1275;GTEx Coexpression,1082	EIF6
1474	ZNF76	1179	ARCHS4 Coexpression,1253;Enrichr Queries,705;GTEx Coexpression,1579	CSTB,CDKN1A,PKM,TSC22D1,APOE,JUNB,ACTB,SOX4,PDLIM7,IER3
1475	FOXD4L1	1181	ARCHS4 Coexpression,1182;Enrichr Queries,1211;GTEx Coexpression,1149	KLF6,CAPNS1,CYP1B1,SOX4
1476	ZNF320	1182	ARCHS4 Coexpression,1176;Enrichr Queries,1014;GTEx Coexpression,1357	CDKN1A,KLF6,JUP,MGAT4A,PMEPA1,PRSS27,SOX4
1477	ZNF324B	1183	ARCHS4 Coexpression,1282;GTEx Coexpression,1083	PKM
1478	DMRT3	1183	ARCHS4 Coexpression,1012;Enrichr Queries,1123;GTEx Coexpression,1413	YWHAE,S100G,CCND2,CYP1B1,LRP2
1479	ZNF793	1183	ARCHS4 Coexpression,1600;Enrichr Queries,1175;GTEx Coexpression,774	CCND2,LAS1L,BASP1,CYP1B1,SOX4
1480	SCX	1184	ARCHS4 Coexpression,1184	UCHL1
1481	ZNF850	1185	ARCHS4 Coexpression,1506;GTEx Coexpression,864	TMSB4X
1482	ZNF276	1188	ARCHS4 Coexpression,1548;Enrichr Queries,714;GTEx Coexpression,1303	LGALS3,CDKN1A,KLF6,GSN,JUP,MYH9,ITGA6,JUNB,PDLIM7,IER3
1483	ZNF805	1195	ARCHS4 Coexpression,1331;Enrichr Queries,1083;GTEx Coexpression,1171	CDKN1A,KLF6,TIMP3,IQGAP1,JUNB,SPTBN1
1484	DMRTB1	1197	ARCHS4 Coexpression,1488;Enrichr Queries,825;GTEx Coexpression,1278	COL1A1,S100G,CDKN1A,CCND2,KRT18,GPX3,LPL,PMEPA1
1485	ZNF727	1198	ARCHS4 Coexpression,1594;GTEx Coexpression,801	ARPC2
1486	ZNF578	1199	ARCHS4 Coexpression,1616;Enrichr Queries,1253;GTEx Coexpression,727	CDKN1A,CCND2,TPM4,IER3
1487	ZNF461	1199	ARCHS4 Coexpression,1045;Enrichr Queries,1293;GTEx Coexpression,1259	CDKN1A,CAPNS1,BASP1,MYH9
1488	ZSCAN5A	1203	ARCHS4 Coexpression,1211;Enrichr Queries,925;GTEx Coexpression,1473	CDKN1A,GSN,TSC22D1,BASP1,PMEPA1,MYH9,JUNB,IER3
1489	SIX3	1205	ARCHS4 Coexpression,1146;Enrichr Queries,1131;GTEx Coexpression,1337	EIF5A,CDKN1A,CCND2,CYP1B1,SOX4
1490	ZNF646	1206	ARCHS4 Coexpression,1326;Enrichr Queries,1152;GTEx Coexpression,1139	LGALS3,CDKN1A,KLF6,MYH9,JUNB
1491	ZIM3	1206	ARCHS4 Coexpression,1416;Enrichr Queries,1089;GTEx Coexpression,1114	IL33,S100G,LRP2,IVL,IER3
1492	ZNF419	1208	ARCHS4 Coexpression,1197;Enrichr Queries,1068;GTEx Coexpression,1358	CDKN1A,KLF6,PKM,TNFRSF12A,JUNB,SOX4
1493	ZNF221	1209	ARCHS4 Coexpression,949;Enrichr Queries,1381;GTEx Coexpression,1296	CAPNS1,MYH9
1494	ZNF200	1210	ARCHS4 Coexpression,1218;Enrichr Queries,1033;GTEx Coexpression,1380	HSPA8,CDKN1A,KLF6,TSC22D1,SRD5A1,SOX4,IER3
1495	ZNF473	1213	ARCHS4 Coexpression,1463;Enrichr Queries,824;GTEx Coexpression,1351	HSPA8,CDKN1A,KLF6,TNFRSF12A,SERPINE1,MYH9,JUNB,IER3
1496	ZNF37A	1213	ARCHS4 Coexpression,1234;Enrichr Queries,1252;GTEx Coexpression,1154	CDKN1A,KLF6,SRD5A1,MGAT4A
1497	ZNF75A	1214	ARCHS4 Coexpression,1278;Enrichr Queries,995;GTEx Coexpression,1368	CDKN1A,KLF6,RACK1,CYP1B1,ITGA6,JUNB,SOX4
1498	ONECUT3	1215	ARCHS4 Coexpression,1257;Enrichr Queries,1209;GTEx Coexpression,1178	CDKN1A,CCND2,MYH9,SOX4
1499	PITX3	1215	ARCHS4 Coexpression,1395;Enrichr Queries,729;GTEx Coexpression,1522	COL1A1,CSTB,CDKN1A,CCND2,SERPINE1,CYP1B1,LPL,JUNB,SOX4
1500	FOXN4	1215	ARCHS4 Coexpression,1461;Enrichr Queries,854;GTEx Coexpression,1331	COL1A1,S100G,CDKN1A,CYP1B1,LPL,TIMP3,LRP2,CLU
1501	ZNF454	1216	ARCHS4 Coexpression,898;Enrichr Queries,1371;GTEx Coexpression,1378	CAPNS1,GSTA4,CYP1B1,MYH9
1502	RFX7	1216	ARCHS4 Coexpression,1128;Enrichr Queries,1242;GTEx Coexpression,1277	CDKN1A,EIF6,GSTO1,CTNNB1,ATP1B1
1503	DPRX	1216	ARCHS4 Coexpression,930;Enrichr Queries,1136;GTEx Coexpression,1581	KLF6,ELF3,RPS5,SOX4,RPS13,IER3
1504	ZNF585B	1216	ARCHS4 Coexpression,1115;Enrichr Queries,1159;GTEx Coexpression,1374	CDKN1A,KLF6,JUP,SRD5A1,FTH1,NUPR1
1505	SNAPC5	1217	ARCHS4 Coexpression,878;GTEx Coexpression,1556	EIF6,MYH9,FLNA
1506	ZNF783	1218	ARCHS4 Coexpression,1483;Enrichr Queries,928;GTEx Coexpression,1243	KLF6,CCND2,ANXA1,TSC22D1,PMEPA1,MYH9,SOX4
1507	ZNF268	1219	ARCHS4 Coexpression,1001;Enrichr Queries,1063;GTEx Coexpression,1592	KLF6,ANXA1,CAPNS1,SRD5A1,CYP1B1,PMEPA1,SOX4
1508	ZNF628	1219	ARCHS4 Coexpression,1468;Enrichr Queries,881;GTEx Coexpression,1308	COL1A1,CDKN1A,JUP,MYH9,APOE,JUNB,ACTB,PDLIM7
1509	ZNF541	1222	ARCHS4 Coexpression,1486;Enrichr Queries,1086;GTEx Coexpression,1094	COL1A1,S100G,CDKN1A,SERPINE1,LCN2
1510	ZNF687	1224	ARCHS4 Coexpression,1475;Enrichr Queries,720;GTEx Coexpression,1476	HSPA8,CDKN1A,KLF6,PKM,MYH9,FLNA,JUNB,RTN4,SPTBN1,FAM129B
1511	ZNF490	1225	ARCHS4 Coexpression,1602;Enrichr Queries,1096;GTEx Coexpression,976	JUP,ARPC1B,MGAT4A,CTNNB1,ATP1B1,JUNB
1512	ZNF814	1226	ARCHS4 Coexpression,924;GTEx Coexpression,1528	PKM,BSG
1513	FOXO6	1229	ARCHS4 Coexpression,1477;Enrichr Queries,859;GTEx Coexpression,1350	COL1A1,S100G,CDKN1A,CYP1B1,PMEPA1,CLU,JUNB,SOX4
1514	CUX2	1234	ARCHS4 Coexpression,1071;Enrichr Queries,1080;GTEx Coexpression,1550	S100G,CDKN1A,BASP1,LPL,PMEPA1,YWHAG
1515	DPF3	1236	ARCHS4 Coexpression,1316;Enrichr Queries,922;GTEx Coexpression,1469	CDKN1A,CCND2,MYOF,PMEPA1,TIMP3,ITGA6,MAP4,ATP1B1
1516	ZNF875	1238	ARCHS4 Coexpression,1360;Enrichr Queries,1037;GTEx Coexpression,1317	LGALS3,CDKN1A,CCND2,GSN,PKM,JUP,SOX4
1517	ZNF354B	1238	ARCHS4 Coexpression,1260;Enrichr Queries,989;GTEx Coexpression,1466	COL1A1,CDKN1A,KLF6,JUNB,SOX4,SPTBN1,IER3
1518	PRDM10	1240	ARCHS4 Coexpression,1539;Enrichr Queries,1177;GTEx Coexpression,1004	HSPA8,CDKN1A,KLF6,MICU1,SPTBN1
1519	HSFX1	1244	ARCHS4 Coexpression,1436;GTEx Coexpression,1052	
1520	ZNF679	1244	ARCHS4 Coexpression,1528;Enrichr Queries,659;GTEx Coexpression,1546	CLIC4,CDKN1A,TNFRSF12A,BASP1,CYP1B1,LRP2,JUNB,ACTB,RPL7,IER3
1521	ZNF844	1249	ARCHS4 Coexpression,1537;GTEx Coexpression,960	CFL1
1522	CGGBP1	1249	ARCHS4 Coexpression,918;GTEx Coexpression,1580	EEF1G,TMEM123,PTGS1
1523	ZNF132	1252	ARCHS4 Coexpression,1530;Enrichr Queries,958;GTEx Coexpression,1269	CSTB,CDKN1A,CCND2,SERPINE1,CYP1B1,PMEPA1,TIMP3
1524	MZF1	1253	ARCHS4 Coexpression,1551;Enrichr Queries,608;GTEx Coexpression,1599	CSTB,CDKN1A,KLF6,JUP,TSC22D1,ELF3,UCP2,APOE,JUNB,SOX4,PDLIM7
1525	VSX1	1253	ARCHS4 Coexpression,1345;Enrichr Queries,1390;GTEx Coexpression,1023	MYH9
1526	ZBTB26	1255	ARCHS4 Coexpression,999;Enrichr Queries,1246;GTEx Coexpression,1519	EEF1G,CDKN1A,TSC22D1,SOX4,S100A11
1527	ZNF485	1255	ARCHS4 Coexpression,1254;Enrichr Queries,1142;GTEx Coexpression,1369	CDKN1A,KLF6,CYP1B1,MYH9,IER3
1528	E2F8	1255	ARCHS4 Coexpression,1335;Enrichr Queries,1308;GTEx Coexpression,1122	CDKN1A,SERPINE1,ITM2B
1529	ZNF487	1257	ARCHS4 Coexpression,1252;Enrichr Queries,930;GTEx Coexpression,1590	CDKN1A,KLF6,GSN,PKM,CYP1B1,SH3BGRL3,JUNB,IER3
1530	ZSCAN22	1258	ARCHS4 Coexpression,1540;Enrichr Queries,767;GTEx Coexpression,1468	HSPA8,CDKN1A,KLF6,CCND2,TNFRSF12A,TSC22D1,JUNB,ACTB,SOX4
1531	SKOR2	1259	ARCHS4 Coexpression,1583;GTEx Coexpression,934	LAS1L
1532	ZNF556	1259	ARCHS4 Coexpression,1593;Enrichr Queries,990;GTEx Coexpression,1195	CSTB,CDKN1A,CCND2,PMEPA1,IER3,PTGS1
1533	MAFA	1260	ARCHS4 Coexpression,1308;Enrichr Queries,1001;GTEx Coexpression,1472	COL1A1,CDKN1A,FXYD2,CYP1B1,CLU,JUNB,IER3
1534	ZNF530	1263	ARCHS4 Coexpression,1136;Enrichr Queries,1386;GTEx Coexpression,1267	CAPNS1
1535	FOXD4L3	1269	ARCHS4 Coexpression,1412;Enrichr Queries,1394;GTEx Coexpression,1000	CALM1
1536	SOHLH2	1269	ARCHS4 Coexpression,1498;Enrichr Queries,779;GTEx Coexpression,1530	CDKN1A,UCHL1,KRT18,SERPINE1,MYOF,CYP1B1,TIMP3,LRP2,CLU
1537	ZNF726	1275	ARCHS4 Coexpression,1595;GTEx Coexpression,954	SRD5A1
1538	ZNF214	1275	ARCHS4 Coexpression,1148;Enrichr Queries,1288;GTEx Coexpression,1390	GSTA4,LRP2,SOX4
1539	ESX1	1277	ARCHS4 Coexpression,1628;Enrichr Queries,821;GTEx Coexpression,1382	S100G,CLDN4,CDKN1A,CCND2,KRT18,ELF3,KRT8,KRT7
1540	TIGD5	1278	ARCHS4 Coexpression,1508;Enrichr Queries,947;GTEx Coexpression,1379	CSTB,CDKN1A,TNFRSF12A,PMEPA1,JUNB,PDLIM7,IER3
1541	RBCK1	1279	ARCHS4 Coexpression,960;GTEx Coexpression,1597	CAPNS1,TAGLN2
1542	JRKL	1280	ARCHS4 Coexpression,1441;Enrichr Queries,810;GTEx Coexpression,1589	CDKN1A,KLF6,ANXA1,TSC22D1,ACTN1,ITGA6,NUPR1,IQGAP1,SOX4
1543	ZNF70	1282	ARCHS4 Coexpression,1611;Enrichr Queries,1179;GTEx Coexpression,1056	CDKN1A,KLF6,CCND2,JUNB
1544	SOHLH1	1283	ARCHS4 Coexpression,1578;Enrichr Queries,707;GTEx Coexpression,1563	CSTB,S100G,CDKN1A,UCHL1,KRT19,KRT14,MGP,LY6D,JUNB,WFDC2
1545	ZNF155	1283	ARCHS4 Coexpression,1606;Enrichr Queries,1053;GTEx Coexpression,1190	GSTA4,CYP1B1,JUNB,SOX4,IER3
1546	POU5F2	1283	ARCHS4 Coexpression,1200;GTEx Coexpression,1366	SRD5A1
1547	ZNF609	1288	ARCHS4 Coexpression,1268;Enrichr Queries,1116;GTEx Coexpression,1481	MYH9,IQGAP1,MAP4,RTN4,SPTBN1
1548	ZXDC	1292	ARCHS4 Coexpression,1495;Enrichr Queries,948;GTEx Coexpression,1434	CSTB,CDKN1A,KLF6,ACTN1,MYH9,JUNB,SOX4
1549	KLF17	1295	ARCHS4 Coexpression,1505;Enrichr Queries,837;GTEx Coexpression,1544	COL1A1,KLF6,ELF3,SERPINE1,CYP1B1,LPL,JUNB,IER3
1550	ZNF563	1301	ARCHS4 Coexpression,1456;Enrichr Queries,844;GTEx Coexpression,1602	CDKN1A,KLF6,BASP1,GPX3,MGAT4A,SERPINE1,CLU,JUNB
1551	ZNF567	1301	ARCHS4 Coexpression,1081;Enrichr Queries,1282;GTEx Coexpression,1539	CDKN1A,CAPNS1,MYH9,SOX4
1552	ZNF707	1305	ARCHS4 Coexpression,1420;Enrichr Queries,959;GTEx Coexpression,1535	CSTB,CDKN1A,KLF6,TNFRSF12A,JUNB,ACTB,IER3
1553	ZNF497	1307	ARCHS4 Coexpression,1263;Enrichr Queries,1281;GTEx Coexpression,1376	PRSS27,IQGAP1,SOX4
1554	ZBED9	1307	ARCHS4 Coexpression,1357;GTEx Coexpression,1257	RACK1
1555	NEUROG1	1308	ARCHS4 Coexpression,1255;Enrichr Queries,1273;GTEx Coexpression,1395	CDKN1A,CCND2,BASP1
1556	ZNF347	1308	ARCHS4 Coexpression,1570;Enrichr Queries,1329;GTEx Coexpression,1025	CDKN1A,CCND2
1557	ZKSCAN8	1310	ARCHS4 Coexpression,1377;GTEx Coexpression,1242	SH3BGRL3
1558	CBLL2	1312	ARCHS4 Coexpression,1470;Enrichr Queries,865;GTEx Coexpression,1601	S100G,CLIC4,KLF6,FUT9,FTH1,MYOF,CYP1B1,TIMP3
1559	PAX7	1313	ARCHS4 Coexpression,1131;Enrichr Queries,1343;GTEx Coexpression,1464	YWHAE,CCND2,RACK1
1560	ZNF383	1313	ARCHS4 Coexpression,1516;Enrichr Queries,1016;GTEx Coexpression,1407	CDKN1A,KLF6,TSC22D1,CYP1B1,JUNB,IER3
1561	GBX1	1316	ARCHS4 Coexpression,1427;Enrichr Queries,1194;GTEx Coexpression,1327	S100G,CDKN1A,LRP2
1562	KCNIP3	1316	ARCHS4 Coexpression,1143;GTEx Coexpression,1489	CADM4
1563	FEZF2	1316	ARCHS4 Coexpression,1258;Enrichr Queries,1201;GTEx Coexpression,1490	S100G,RACK1,CYP1B1,LPL
1564	ZNF630	1318	ARCHS4 Coexpression,1107;GTEx Coexpression,1529	PKM,MYH9
1565	ZNF788P	1321	ARCHS4 Coexpression,1553;Enrichr Queries,1203;GTEx Coexpression,1208	CCND2,BASP1,CYP1B1
1566	ZNF470	1326	ARCHS4 Coexpression,1179;Enrichr Queries,1301;GTEx Coexpression,1497	BASP1,SRD5A1,SERPINE1
1567	ZNF785	1327	ARCHS4 Coexpression,1596;GTEx Coexpression,1057	
1568	LHX4	1329	ARCHS4 Coexpression,1529;Enrichr Queries,1291;GTEx Coexpression,1166	S100G,CYP1B1
1569	LIN28A	1333	ARCHS4 Coexpression,1269;GTEx Coexpression,1396	LDHB
1570	ZNF736	1333	ARCHS4 Coexpression,1126;GTEx Coexpression,1540	SRD5A1,CFL1
1571	SKOR1	1334	ARCHS4 Coexpression,1480;GTEx Coexpression,1188	
1572	GSC2	1334	ARCHS4 Coexpression,1422;Enrichr Queries,1264;GTEx Coexpression,1316	CSTB,S100G
1573	ZNF474	1334	ARCHS4 Coexpression,1289;Enrichr Queries,1129;GTEx Coexpression,1584	COL1A1,CDKN1A,SERPINE1,KRT7,WFDC2
1574	MTERF4	1335	ARCHS4 Coexpression,1110;GTEx Coexpression,1559	MYOF,FAM129B
1575	ZNF233	1335	ARCHS4 Coexpression,1244;Enrichr Queries,1237;GTEx Coexpression,1525	SRD5A1,CYP1B1,LRP2,FMO5
1576	ZNF546	1335	ARCHS4 Coexpression,1549;Enrichr Queries,1117;GTEx Coexpression,1340	CDKN1A,KLF6,TSC22D1,GSTA4,MGAT4A
1577	ZNF8	1338	ARCHS4 Coexpression,1577;Enrichr Queries,1099	CDKN1A,KLF6,CCND2,JUNB,SOX4
1578	DNMT1	1342	ARCHS4 Coexpression,1433;GTEx Coexpression,1251	
1579	ZSCAN20	1343	ARCHS4 Coexpression,1423;Enrichr Queries,1188;GTEx Coexpression,1419	CLIC4,CDKN1A,KLF6,MYH9
1580	YBX3	1347	ARCHS4 Coexpression,1392;GTEx Coexpression,1301	
1581	ZFAT	1347	ARCHS4 Coexpression,1426;Enrichr Queries,1254;GTEx Coexpression,1360	CDKN1A,KLF6,PMEPA1
1582	ZNF599	1350	ARCHS4 Coexpression,1336;Enrichr Queries,1176;GTEx Coexpression,1538	COL1A1,PMEPA1,MYH9,SOX4,IER3

1583	ZNF843	1354	ARCHS4 Coexpression,1503;Enrichr Queries,1235;GTEx Coexpression,1323	CDKN1A,CYP1B1,JUNB
1584	ZNF99	1354	ARCHS4 Coexpression,1609;Enrichr Queries,1374;GTEx Coexpression,1078	CCND2
1585	TFDP3	1355	ARCHS4 Coexpression,1432;Enrichr Queries,1318;GTEx Coexpression,1314	CSTB,S100G
1586	SOX30	1355	ARCHS4 Coexpression,1450;Enrichr Queries,1125;GTEx Coexpression,1491	CSTB,CDKN1A,CYP1B1,LRP2
1587	ZNF619	1361	ARCHS4 Coexpression,1554;Enrichr Queries,1362;GTEx Coexpression,1167	KLF6
1588	NANOGNB	1364	ARCHS4 Coexpression,1327;GTEx Coexpression,1400	MYH9
1589	RHOXF2B	1375	ARCHS4 Coexpression,1465;Enrichr Queries,1326;GTEx Coexpression,1335	S100G,CDKN1A
1590	ZNF41	1377	ARCHS4 Coexpression,1313;Enrichr Queries,1365;GTEx Coexpression,1454	TIMP3,IQGAP1
1591	ZNF891	1378	ARCHS4 Coexpression,1536;GTEx Coexpression,1220	
1592	NKX24	1379	ARCHS4 Coexpression,1438;GTEx Coexpression,1320	
1593	TGIF2LY	1383	ARCHS4 Coexpression,1574;Enrichr Queries,1044;GTEx Coexpression,1532	S100G,KLF6,KRT14,LCN2,IER3
1594	ZNF142	1384	ARCHS4 Coexpression,1552;Enrichr Queries,1311;GTEx Coexpression,1289	MYH9,PDLIM7
1595	ARGFX	1387	ARCHS4 Coexpression,1526;Enrichr Queries,1247;GTEx Coexpression,1387	KLF6,FUT9,PTGS1
1596	ALX3	1388	ARCHS4 Coexpression,1490;Enrichr Queries,1191;GTEx Coexpression,1482	COL1A1,S100G,CYP1B1
1597	OVOL3	1393	ARCHS4 Coexpression,1523;GTEx Coexpression,1262	
1598	ZBTB49	1394	ARCHS4 Coexpression,1507;GTEx Coexpression,1281	
1599	ZNF283	1395	ARCHS4 Coexpression,1599;Enrichr Queries,1153;GTEx Coexpression,1433	CDKN1A,TSC22D1,ANXA3,MYOF
1600	ZNF81	1401	ARCHS4 Coexpression,1618;Enrichr Queries,1364;GTEx Coexpression,1222	PMEPA1
1601	LBX1	1408	ARCHS4 Coexpression,1453;Enrichr Queries,1332;GTEx Coexpression,1439	CCND2
1602	TERB1	1410	ARCHS4 Coexpression,1620;GTEx Coexpression,1200	
1603	HSF5	1410	ARCHS4 Coexpression,1437;Enrichr Queries,1218;GTEx Coexpression,1575	S100G,TIMP3,LRP2
1604	ZNF507	1410	ARCHS4 Coexpression,1557;Enrichr Queries,1344;GTEx Coexpression,1329	SPTBN1
1605	ZNF225	1415	ARCHS4 Coexpression,1350;GTEx Coexpression,1480	CAPNS1
1606	ZNF774	1416	ARCHS4 Coexpression,1610;Enrichr Queries,1173;GTEx Coexpression,1465	COL1A1,ATP1B1,JUNB,FMO5
1607	ZNF492	1418	ARCHS4 Coexpression,1622;GTEx Coexpression,1214	
1608	ZNF705B	1420	ARCHS4 Coexpression,1285;GTEx Coexpression,1555	SRD5A1
1609	ZNF676	1431	ARCHS4 Coexpression,1613;Enrichr Queries,1163;GTEx Coexpression,1517	COL1A1,MGP,LPL,CP
1610	CPXCR1	1441	ARCHS4 Coexpression,1417;Enrichr Queries,1327;GTEx Coexpression,1578	S100G,FUT9
1611	SHOX	1443	ARCHS4 Coexpression,1550;Enrichr Queries,1357;GTEx Coexpression,1423	CYP1B1
1612	ZNF705D	1446	ARCHS4 Coexpression,1547;Enrichr Queries,1306;GTEx Coexpression,1484	JUNB,IER3
1613	ZNF182	1447	ARCHS4 Coexpression,1581;Enrichr Queries,1169;GTEx Coexpression,1591	CDKN1A,TSC22D1,JUNB,SOX4
1614	FOXD4L6	1449	ARCHS4 Coexpression,1504;Enrichr Queries,1393	
1615	ZNF729	1452	ARCHS4 Coexpression,1559;GTEx Coexpression,1345	
1616	TGIF2LX	1452	ARCHS4 Coexpression,1451;Enrichr Queries,1307;GTEx Coexpression,1598	S100G,SOX4
1617	ZNF705G	1455	ARCHS4 Coexpression,1343;GTEx Coexpression,1566	SRD5A1
1618	SPZ1	1456	ARCHS4 Coexpression,1410;GTEx Coexpression,1502	
1619	ZNF829	1457	ARCHS4 Coexpression,1499;Enrichr Queries,1360;GTEx Coexpression,1511	CDKN1A
1620	ZNF716	1466	ARCHS4 Coexpression,1588;Enrichr Queries,1375;GTEx Coexpression,1436	ANXA1
1621	HSFX2	1467	ARCHS4 Coexpression,1467	
1622	FAM170A	1469	ARCHS4 Coexpression,1584;Enrichr Queries,1303;GTEx Coexpression,1521	CERS3,S100G
1623	ZIM2	1475	ARCHS4 Coexpression,1444;Enrichr Queries,1373;GTEx Coexpression,1607	UCHL1
1624	PHF1	1476	ARCHS4 Coexpression,1520;GTEx Coexpression,1431	
1625	ZSCAN32	1478	ARCHS4 Coexpression,1582;GTEx Coexpression,1373	
1626	CCDC169SOHLH2	1487	ARCHS4 Coexpression,1487	
1627	NKX11	1494	ARCHS4 Coexpression,1563;GTEx Coexpression,1424	
1628	EVX2	1500	ARCHS4 Coexpression,1512;Enrichr Queries,1402;GTEx Coexpression,1586	
1629	KAT7	1502	ARCHS4 Coexpression,1542;GTEx Coexpression,1462	
1630	ZNF479	1507	ARCHS4 Coexpression,1592;Enrichr Queries,1351;GTEx Coexpression,1577	IER3
1631	MBD6	1517	ARCHS4 Coexpression,1533;GTEx Coexpression,1500	
1632	GLI4	1584	ARCHS4 Coexpression,1572;GTEx Coexpression,1595	
